# Associations between inclusivity norms and tolerance, contact, and cooperation amid polarization: Evidence from 12 European countries

**DOI:** 10.1093/pnasnexus/pgag087

**Published:** 2026-03-26

**Authors:** Laura F Schäfer, Nicole Tausch, Marcin Bukowski, Eva Jaspers, Miranda J Lubbers, Maarten van Zalk, Alejandro Ciordia, Anna Potoczek, Lucía Estevan-Reina, Maor Shani, Jan-Willem Simons, Maria-Therese Friehs, Dominika Gurbisz, Wilma Middendorf, Sarina J Schäfer, Jil Ullenboom, Sylvie Graf, Mikael Hjerm, Chloé Lavest, Inga Jasinskaja-Lahti, Anna Kende, Katerina Petkanopoulou, Francesca Prati, Oliver Christ

**Affiliations:** Faculty of Psychology, FernUniversität in Hagen, 58097 Hagen, Germany; School of Psychology and Neuroscience, University of St Andrews, St Andrews KY16 9AJ, United Kingdom; Institute of Psychology, Jagiellonian University, 30-348 Kraków, Poland; Department of Sociology, Utrecht University, 3584 CH Utrecht, The Netherlands; Department of Social and Cultural Anthropology, Autonomous University of Barcelona, 08193 Bellaterra, Spain; Department of Psychology, University of Osnabrück, 49076 Osnabrück, Germany; Maastricht Sustainability Institute, Maastricht University, Maastricht, 6211 ME, The Netherlands; Interdisciplinary Centre for Mathematical and Computational Modelling, University of Warsaw, 02106 Warszawa, Poland; Institute of Psychology, Jagiellonian University, 30-348 Kraków, Poland; Department of Social Psychology, University of Granada, 18071 Granada, Spain; Department of Psychology, University of Osnabrück, 49076 Osnabrück, Germany; Department of Sociology, Utrecht University, 3584 CH Utrecht, The Netherlands; Faculty of Psychology, FernUniversität in Hagen, 58097 Hagen, Germany; Institute of Psychology, Jagiellonian University, 30-348 Kraków, Poland; Department of Psychology, University of Osnabrück, 49076 Osnabrück, Germany; Faculty of Psychology, FernUniversität in Hagen, 58097 Hagen, Germany; Faculty of Psychology, FernUniversität in Hagen, 58097 Hagen, Germany; Institute of Psychology, Academy of Sciences of the Czech Republic, 602 00 Brno, Czech Republic; Department of Sociology, Umeå University, 901 87 Umeå, Sweden; Department of Sociology, Utrecht University, 3584 CH Utrecht, The Netherlands; Faculty of Social Sciences, University of Helsinki, 00014 Helsinki, Finland; Institute of Psychology, ELTE Eötvös Loránd University, 1064 Budapest, Hungary; Department of Psychology, University of Crete, 74150 Rethymno, Greece; Department of Psychology, University of Bologna, 33 - 40126 Bologna, Italy; Faculty of Psychology, FernUniversität in Hagen, 58097 Hagen, Germany

**Keywords:** social norms, polarization, intergroup relations

## Abstract

Sharp disagreements about social issues have raised concerns about increasing societal polarization in democracies worldwide. While diversity of opinion is vital for democratic engagement and can promote innovative solutions, social science research shows that such disagreements can undermine social cohesion and erode social trust if they turn into identity-based conflicts. The present research examines the potential importance of social norms that promote equality-based respect, dialogue, and unity (inclusivity norms) in mitigating these negative outcomes. In a preregistered, large-scale survey of 12,041 individuals in 12 European countries, we find that perceived inclusivity norms are associated with increased tolerance, greater willingness to collaborate, and lower tendencies to avoid people who hold opposing opinions. In most countries studied, this pattern holds among respondents who strongly disapprove of the opposing viewpoint, endorse antipluralist opinions, hold anti-egalitarian preferences, and identify more strongly with their opinion-based group relative to society. These findings support the potential of inclusivity norms to preserve social trust and cohesion amid opinion diversity.

Significance statement Perceived social norms shape intergroup relations, but how do they relate to the societal challenges that arise in polarized contexts? We examine the potential importance of inclusivity norms—a combination of equality-based respect, dialogue, and unity—as a counterweight to the potential negative consequences of polarization. Using data from 12 European countries, we find associations of perceived inclusivity norms with greater tolerance toward, more cooperation with, and less avoidance of those with opposed opinions. We evaluate the robustness of these findings among groups most prone to the negative effects of opinion polarization. Our findings have implications for the development of scalable approaches to address the negative consequences of polarization.

## Introduction

From heated debates over climate change and migration policies to intense discussions about gender equality and transgender rights—societies around the world are facing numerous challenges that spark polarized debates (1, 2). Differences in views on important societal issues are not necessarily harmful. On the contrary, scholars emphasize that some level of disagreement is integral to a healthy democracy (3, 4). However, divergence in opinion can become pernicious (4) when it is accompanied by the formation of opinion-based groups (5, 6). Opinion-based groups, like other social categories such as age, gender, or nationality, can function as genuine psychological groups, providing a social identity grounded in shared opinions. According to self-categorization theory, as individuals identify with these groups, they become divided into either the ingroup—those who share an opinion—or the outgroup—those with opposing views (7–9). Consequently, opinion-driven conflicts are likely to shift into identity-driven ones (10–12). Social psychological theories of intergroup conflict explain the conditions that produce negative attitudinal and behavioral outcomes of social categorization and identification with distinct social groups (9, 13, 14). Indeed, in many societies, the divide into opinion-based groups has been linked to dislike and distrust of those with opposing views (i.e. affective polarization) (11, 15, 16), low levels of tolerance (17, 18), reduced willingness to cooperate and compromise (19), and increased tendencies to avoid outgroup members (11, 18, 20). These individual-level tendencies may ultimately undermine social cohesion and social trust at the societal level, posing a threat to the very fabric of democracy (4, 12, 21, 22). It is therefore crucial to explore strategies that hold the potential to promote tolerance, contact, and cooperation among people with differing viewpoints and may thereby help to protect social trust and cohesion at the societal level.

Previous research has examined the effectiveness of several approaches to improve intergroup outcomes in polarized contexts. For instance, scholars have investigated the impact of intergroup contact interventions that brought together members of opposing opinion or partisan groups, demonstrating that such contact can mitigate negative intergroup attitudes (23–26) and foster more positive inter-partisan affect (27, 28). Likewise, interventions that reduce the overestimation of outgroup negativity toward the ingroup, thus correcting exaggerated negative meta-perceptions, were shown to reduce affective polarization (29). However, these interventions are limited in their scope, often fall short in engaging highly biased individuals, and are likely to only produce relatively short-lived effects on intergroup outcomes (23, 25, 26). Moreover, interventions that primarily focus on improving intergroup affect do not always mitigate anti-democratic attitudes and thus might be less consequential for direct democratic outcomes (30). Together, this may limit the effectiveness of individual-centered approaches in reducing aversive behaviors and their broader societal consequences.

In this paper, we focus on perceived social norms as a complementary factor that may be relevant for understanding when and why polarized opinion contexts are more or less likely to be accompanied by negative intergroup outcomes. To examine this, we conducted a preregistered large-scale survey with representative samples from 12 European countries (Czechia, Finland, France, Germany, Greece, Hungary, Italy, The Netherlands, Poland, Spain, Sweden, and the United Kingdom). To situate our research within the context of opinion polarization, we identified controversial issues that are salient across all countries surveyed (i.e. climate change, migration and refugee policies, gender equality, transgender rights, COVID-19 vaccination, and meat consumption) (15, 31). Specifically, we test whether perceptions of social norms that emphasize equality-based respect, dialogue, and unity (inclusivity norms) are associated with more constructive intergroup orientations. Identifying such associations can shed light on the broader societal conditions that help sustain trust and cohesion amid opinion diversity and provide insight into whether inclusivity norms represent a promising avenue for future large-scale, sustainable intervention efforts.

A vast literature has shown that perceived social norms guide individuals' behaviors—they are the informal rules that we live by (32–34). Merely knowing what others approve of (injunctive norms) or do (descriptive norms) can encourage individuals to do the same (35, 36), especially when both types of norms align (37–39). The Theory of Planned Behavior (40) emphasizes that perceived norms contribute to behavioral intentions over and above individual attitudes, highlighting that behavior is shaped not only by what one personally believes but also by perceptions of what others believe and do. A substantial body of research supports this idea. Perceived social norms have been shown to strongly influence individual behavior such as climate-related behavior (41), health behavior (42), cooperation between individuals (43) and within groups (44), but also attitudes and behaviors that are more directly linked to intergroup relations such as prejudice and appreciation of diversity (45, 46), willingness to engage in contact with outgroup members (47), and social cohesion among diverse groups (48, 49). Initial empirical evidence also suggests that perceived social norms might help to foster political tolerance (50). Moreover, experimental and field research shows that shifts in perceived norms can influence behavior even when personal attitudes remain stable. For example, a field study in Rwanda demonstrated that changes in perceived norms alone were sufficient to produce behavioral change (51).

Rather than attempting to reduce disapproval of opposing beliefs or to enhance liking of those with opposing views—factors that are likely to be less amenable to change (52)—we focus on the association between perceived inclusivity norms and individual orientations that support democratic exchange. In particular, we examine individuals' tolerance of opposing opinions, their willingness to actively approach (rather than avoid) those who disagree on an issue, and their readiness to cooperate across opinion divides, even in contexts where there is strong disapproval of opposing views (see Table 1 for an overview of constructs and measures). Tolerance has been highlighted as an indispensable foundation for engaging with diverse viewpoints, beliefs, and convictions. It extends beyond mere indifference as it implies accepting different opinions or practices without expecting others to refrain from them and granting everyone the same rights of civic participation despite one's own disapproval (53–55). Consequently, it forms a fundamental basis of democracy by allowing diverse perspectives to coexist without suppression. However, tolerance alone may not be enough to encourage people to actively connect or cooperate across differing viewpoints, which is essential for working toward the common good (56). Research suggests that a passive form of tolerance—i.e. not interfering with the disapproved conduct of others—is more common than an active form, where individuals engage with, support, or even defend the rights of others even if these others hold opposing viewpoints (57). In polarized settings, preserving social cohesion and social trust requires more than mere passive tolerance of differing opinions. The stability and functioning of societies often hinge on people's willingness to approach rather than avoid those with opposing views and their ability to compromise and cooperate across social divides (12, 21, 58).

**Table 1:** Overview of constructs, measures, definitions, and example items.

Construct	Descriptive inclusivity norms	
Measures	Definition	Example items
Equality-based respect norms	The perception that the majority of people in a given country “treat” others as equals, recognizing their equal standing in society.	Most people in [country] always treat everyone as a human being of equal worth.
Dialogue norms	The perception that the majority of people in a given country “actively listen” to others, receptive to their experiences and perspectives.	Most people in [country] really listen to everyone to better understand differences.
Unity norms	The perception that the majority of people in a given country “view” others as members of the same superordinate group (i.e. society) and recognize shared challenges.	Most people in [country] support the view that despite the differences between the people in [country], we are all part of a single community.

Construct	Injunctive inclusivity norms	
Measures	Definition	Example items
Equality-based respect norms	The perception that the majority of people in a given country “believe everyone should” treat others as equals and recognize their equal standing in society.	Most people in the [country] believe that we should always treat everyone as a human being of equal worth.
Dialogue norms	The perception that the majority of people in a given country “believe everyone should” actively listen to others and be receptive to their experiences and perspectives.	Most people in the [country] believe that we should really listen to everyone so that differences can be better understood.
Unity norms	The perception that the majority of people in a given country “believe everyone should” view others as members of the same superordinate group (i.e. society) and recognize shared challenges.	Most people in the [country] believe that we should support the view that despite the differences between the people in [country], we are all part of a single community.

Construct	Individual-level orientations	
Measures	Definition	Example items
Tolerance	The acceptance of other viewpoints, beliefs, and practices despite personal disapproval.	These people shall be given the chance to pursue their interests just like others.
Avoidance tendencies	Tendencies to withdraw from interactions with individuals holding disapproved views or opinions, as opposed to seeking intergroup contact.	I avoid contact with these people if I can.
Cooperation willingness	Readiness to work together with individuals holding disapproved views or opinions toward common goals.	I would cooperate with these people to solve problems that affect all members of society.

**Figure 1:**
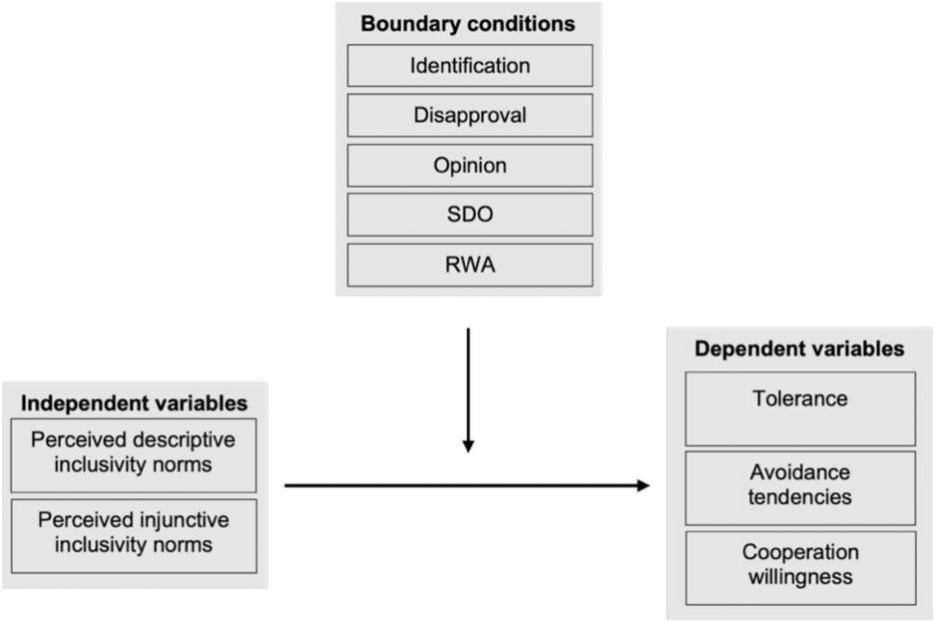
Conceptual model showing perceived descriptive and injunctive inclusivity norms predicting tolerance, avoidance tendencies, and cooperation willingness, with moderation by identification, disapproval, opinion, social dominance orientation, and right-wing authoritarianism.

Norms emphasizing equality-based respect, dialogue, and unity describe complementary ways in which societies signal that disagreement can occur within a framework of mutual recognition, open communication, and shared belonging, and guide individuals not only to accept opposing views but also to engage in contact and cooperation with those who hold different views (see Table 1 for an overview). For example, respecting others as equals may lead individuals to tolerate divergent opinions (53, 59), and engaging in dialogue may reduce tendencies to avoid them (53, 59); however, willingness to cooperate with those holding divergent beliefs may still depend on recognizing shared belonging and common challenges (52, 60, 61). As these elements are mutually reinforcing and frequently co-articulated in core frameworks of pluralistic democracy and diversity ideologies (52, 62, 63), we conceptualize them as facets of a single overarching construct—inclusivity norms—that captures a broader societal orientation relevant for constructive democratic exchange. While past research has examined these aspects as individual beliefs, our focus lies on their potential when perceived as social norms, which we refer to as inclusivity norms. Specifically, we test whether inclusivity norms are associated with individual orientations and country-level indicators critical to a functioning democracy. On the country level, we first descriptively explore the associations between the aggregated measure of perceived descriptive inclusivity norms (i.e. individuals' view of the extent to which people in their society treat others as equals, engage in open dialogue, and show support for unity) as a proxy of actual behavior in each country and aggregated measures of perceived social cohesion (i.e. individuals' sense of connectedness within society) and social trust (i.e. individuals' belief in the reliability of others within society). Both social cohesion and social trust, in their aggregated form, represent societal-level indicators that provide insight into how people live together within a country and how people relate to one another and manage diversity. Therefore, we examined these constructs at the country level rather than at the individual level. Additionally, we include an index of affective polarization developed by previous research (15) to explore the relationship between country-level polarization and aggregated perceptions of descriptive inclusivity norms. On the individual level, we assess the association between both perceived descriptive and injunctive (i.e. what people are perceived to approve of) inclusivity norms and the three indicators of constructive engagement, namely outgroup tolerance and tendencies to avoid and cooperate with opposing others. Further, we test whether the strength of associations between inclusivity norms and these individual orientations is stronger when perceptions of perceived descriptive and injunctive norms align. Importantly, we also examine potential boundary conditions (i.e. factors that may constrain the assumptions of a theoretical model) (64, 65) of the link between perceived inclusivity norms and the individual orientations (Figure 1). These boundary conditions include respondents' strength of disapproval of the opposing opinion, their identification with their opinion-based ingroup relative to society, their position on the controversial issue, and their levels of social-dominance orientation (SDO) and right-wing authoritarianism (RWA). SDO and RWA are well-established individual-difference variables. SDO captures the extent to which people favor group-based dominance and social hierarchy over democratic pluralism, whereas RWA reflects individuals' tendency to obey authority and conform to traditional norms (66). Both variables are strongly associated with anti-democratic tendencies and intolerant attitudes (67, 68). To better understand the potential reach of inclusivity norms, we raise the question: Do the associations between perceived descriptive and injunctive inclusivity norms and tolerance, (lack of) avoidance, and cooperation willingness hold even among individuals who strongly disapprove of the opposing viewpoint; who identify more strongly with their opinion-based ingroup than with society as a whole; who endorse antipluralist opinions; who favor group-based dominance over democratic pluralism; and who tend to adhere to authority and conform to traditional norms?

## Results

We analyze a large and unique survey dataset comprising 12,041 respondents from 12 European countries, each characterized by a varying degree of affective polarization (15). We recruited respondents from high-quality panels and collected data online between November 9 and December 6, 2023, using a quota sampling method based on age, gender, and education to approximate the demographic proportions in all countries (see Table S1 for details).

Our main goal is to examine the associations between perceived inclusivity norms and the individual orientations (tolerance, avoidance tendencies, and cooperation willingness) across 12 countries, assessing their robustness across diverse contexts and contested topics. Due to the small number of countries, we refrained from using multilevel analysis to combine individual- and country-level data. Instead, we conducted separate analyses: (i) descriptive country-level analyses to provide preliminary insights into bivariate correlations between aggregated individual-level measures of descriptive inclusivity norms and the aggregated individual-level measures of perceived social cohesion and social trust, both key indicators of a healthy democracy; and (ii) inferential individual-level analyses to explore the relationship between perceived inclusivity norms and self-reported individual behaviors and behavioral intentions in greater depth.

Note that the present research is part of a broader project that also considers the formation, transmission, and local operation of inclusivity norms. For this reason, the current dataset also includes measures of personal-network norms and several additional constructs capturing network-specific processes. We report additional analyses assessing the predictive role of network norms in the Supplementary Material (Tables S127 and S128). A detailed comparison of the predictive role of societal and network norms is, however, beyond the theoretical scope of the present paper, which focuses on the perceived societal normative climate surrounding democratic exchange rather than on norm dynamics within small-scale social networks.

### Country-level analyses

In some countries (e.g. Greece), there were deviations from the demographic proportions of the samples, particularly in terms of education. To correct for these imbalances, we applied sampling weights in all descriptive analyses. These weights adjusted for over- or underrepresentation of age, gender, and educational groups, ensuring that the aggregated data more accurately reflected the population proportions.

**Figure 2:**
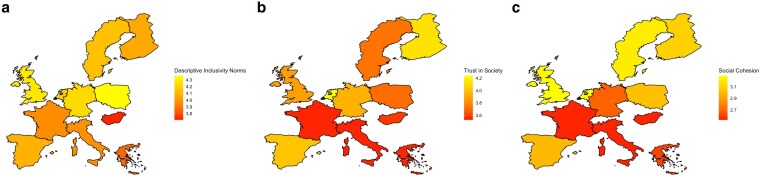
Three heat maps of Europe labelled a to c, illustrating aggregated descriptive inclusivity norms (a), social trust (b), and social cohesion (c), with lighter colors indicating higher values and darker colors indicating lower values across countries.

To investigate the role of perceived inclusivity norms in the context of opinion polarization and their relationship with social cohesion and trust at the country level, we calculated bivariate correlations between aggregated measures of these variables (Table S3). For these descriptive analyses, we used perceived descriptive inclusivity norms as a proxy for the perception of actual behavior of members of society in each country. As depicted in Figure 2, on a descriptive level, there are variations in these aggregated measures between the analyzed countries (the lighter the color, the higher the aggregated value). Moreover, the aggregated individual-level measure of perceived descriptive inclusivity norms is positively related with the aggregated individual-level measures of social trust, *r* = 0.50, and social cohesion, *r* = 0.68.

In addition, we included an index of affective polarization between opinion-based groups, derived from prior research (15) in nearly all countries studied (except the United Kingdom). The index measures affective polarization by quantifying the emotional distance between respondents' feelings toward opposing opinion-based groups related to seven topics (i.e. immigration, the war in Ukraine, pandemic control, climate change, social benefits and their financing, gender equality in society, and policy toward sexual minorities). Respondents rated each group on a feeling thermometer scale, and the absolute difference between these ratings for each issue represents the polarization score, with 0 indicating identical feelings and 10 reflecting maximum divergence. Summing these scores across all issues yields an individual index value ranging from 0 (no polarization) to 70 (maximum polarization). For each country, the national index is calculated by averaging the individual scores of all respondents, providing a measure of overall affective polarization (15). Affective polarization correlates negatively with the aggregated individual-level measures of perceived descriptive inclusivity norms, *r* = −0.32, social trust, *r* = −0.44, and social cohesion, *r* = −0.59.

Taken together, although the correlations reported above, also due to the small sample size (*n* = 12), should be regarded as descriptive and preliminary evidence, these findings point to perceived descriptive inclusivity norms covarying with perceived social cohesion and social trust, as well as with an external affective polarization index derived from independent previous research. In other words, in countries where people are perceived to respect others, engage in dialogue, and feel a sense of shared belonging, perceptions of social cohesion and social trust tend to be higher, whereas affective polarization tends to be lower.

### Individual-level analyses

In this section, we report the individual-level analyses in four steps. First, we describe the measurement strategy used to assess individuals' behavioral orientations, based on an adapted version of the least-liked group approach targeting opinion-based groups. Second, we outline the analytical strategy. Third, we present results from Bayesian hierarchical regression analyses examining associations between perceived inclusivity norms and individual orientations within each country, as well as tests of normative alignment through interactions between injunctive and descriptive inclusivity norms. Fourth, we report several robustness checks assessing the stability of these findings.

#### Measurement strategy

**Figure 3:**
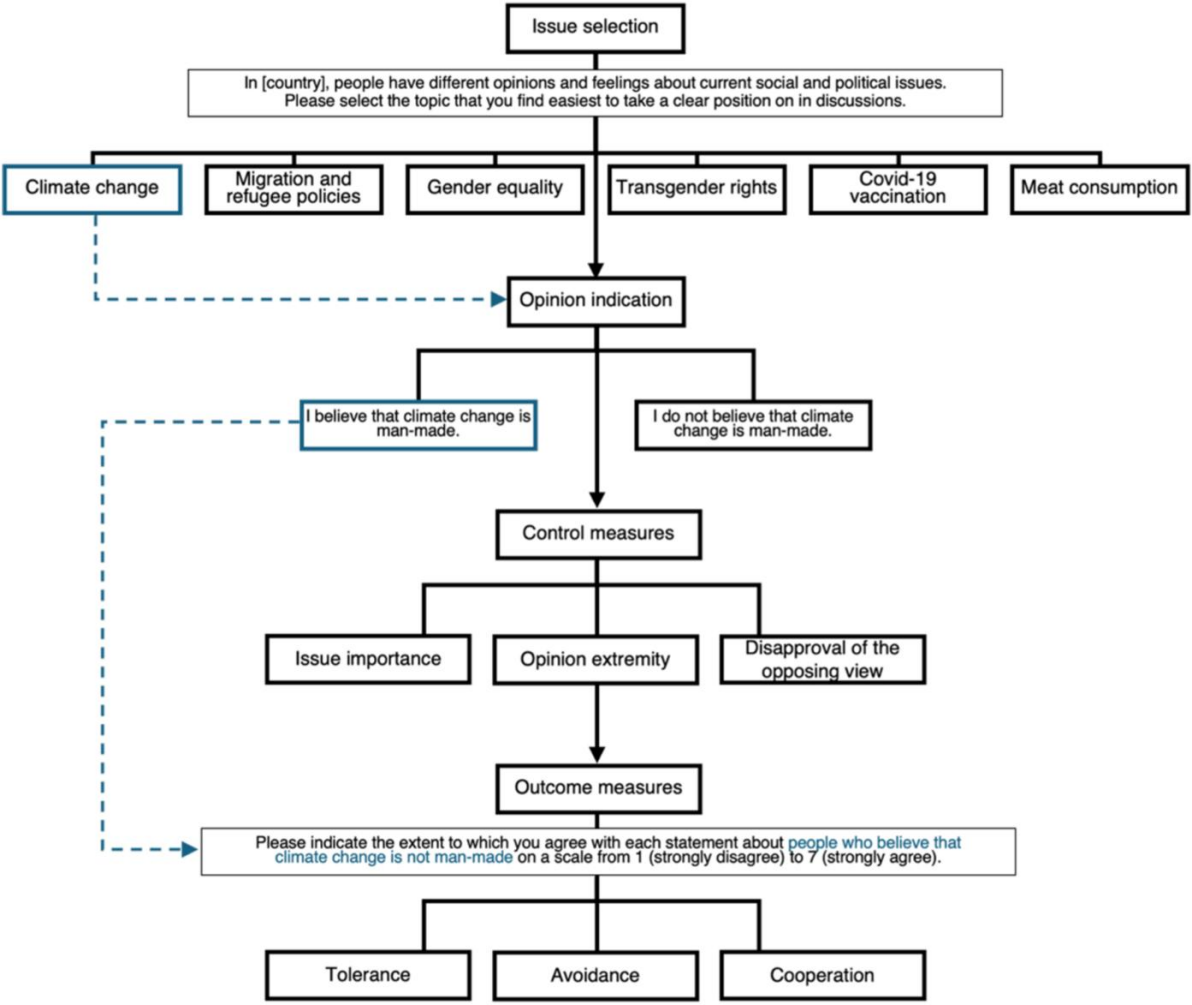
Flowchart illustrating all steps of the adapted version of the least-liked group approach focusing on opinion-based groups, from issue selection and opinion indication to responses toward an opposing opinion-based group, including control measures and outcomes of tolerance, avoidance, and cooperation.

We assessed all individual orientations using an adapted version of the least-liked group approach, focusing on opinion-based groups (Figure 3) (69, 70). Respondents first selected an issue on which they had the firmest stance from a list of six contested issues (i.e. climate change, migration and refugee policies, gender equality, transgender rights, COVID-19 vaccination, and meat consumption). We preselected the issues based on previous research on opinion-based groups and polarization in Europe (15, 31). After selecting an issue (e.g. climate change), respondents indicated their opinion on a specific controversy related to that issue (e.g. human causes of climate change) and then answered the items assessing the dependent variables with reference to the opposing opinion-based group. For instance, if respondents believed climate change was man-made, we asked them for their attitudes and behaviors toward individuals holding the opposite view (i.e. people who do not believe that climate change is man-made). Respondents then answered questions on their chosen issue, including measures of attitude strength, identification with like-minded people, and the strength of disapproval toward the opposite opinion. This approach ensures that respondents have a clear opinion, identify to some extent with a group linked to the issue, and hold disapproval toward the opposite opinion, although with a varying degree in strength.

#### Analytical strategy

We conducted Bayesian hierarchical regression analyses separately for each country using a noninformative (improper flat) prior as the default in R's brms package (RStudio version 2024.09.1 + 394) and model fitting was performed with the NUTS (no U-turn sampler) algorithm in Stan with the default settings in all analyses; the NUTS algorithm showed satisfactory convergence (rhat = 1). We report the mean and corresponding 95% credibility interval for all coefficients of interest in each analysis. In Bayesian terms, an association is considered “credible” (i.e. significant) when its 95% credible interval does not include zero, that is, when the posterior distribution provides sufficient probability for a nonzero effect. For the robustness checks, we additionally conducted hypothesis tests by calculating Bayes factors (BFs) for predictors with credible intervals including zero to quantify the evidence in support of the null hypothesis (BF_01_). Following Jeffreys' (1961) (71) evidence categories for interpreting BFs, a BF = 1 reflects equal support for *H*_0_ and *H*_1_, while BF_01_ > 3 and BF_01_ > 10 indicate substantial and strong evidence in favor of *H*_0_, respectively.

#### Associations between inclusivity norms and individual orientations

First, we regressed tolerance, cooperation willingness, and avoidance tendencies on perceived injunctive and descriptive inclusivity norms. Consistent with previous research using the least-liked group approach, we controlled for opinion extremity (i.e. the strength of one's own opinion on the issue) and issue importance (i.e. the personal importance of the issue), both well-established indicators of attitude strength without reference to the outgroup (70, 72). To align with previous research on intergroup tolerance, we included disapproval as a control variable (59).

**Figure 4:**
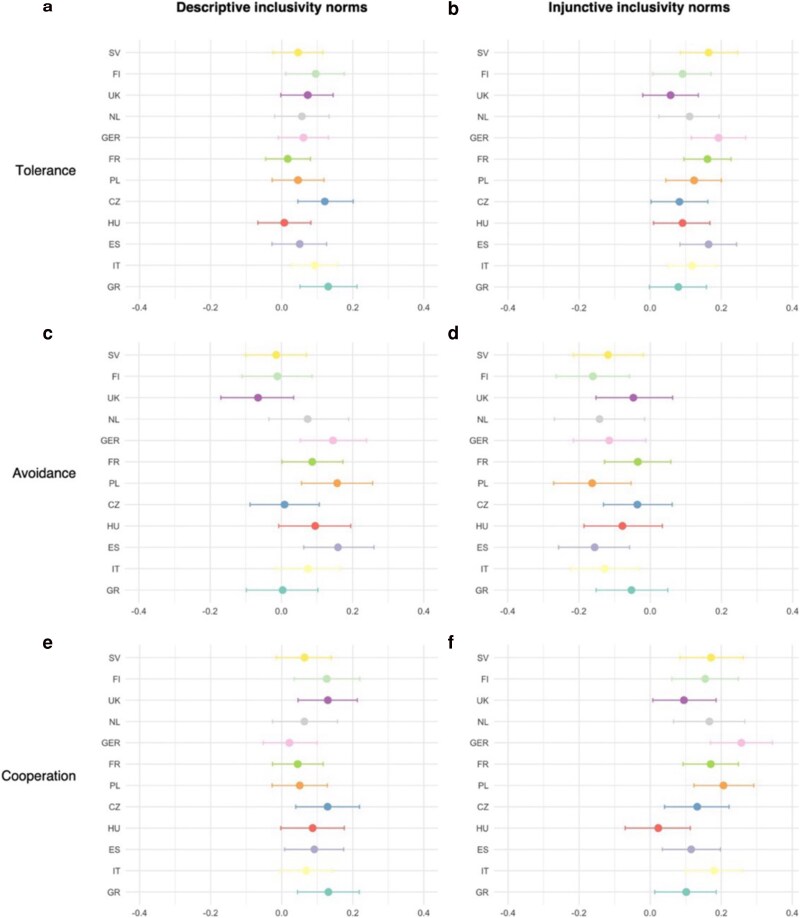
Six panels labelled a to f illustrating posterior regression estimates with credible intervals across countries, showing associations between descriptive and injunctive inclusivity norms and outcomes of tolerance, avoidance, and cooperation. a) Posterior estimates with credible intervals for tolerance regressed on perceived descriptive inclusivity norms for each country (*b* = 0.01 to *b* = 0.13; 95% CI [−0.07, 0.8] to 95% CI [0.05, 0.21]). b) Posterior estimates with credible intervals for tolerance regressed on perceived injunctive inclusivity norms for each country (*b* = 0.06 to *b* = 019; 95% CI [−0.03, 014] to 95% CI [0.11, 0.27]). c) Posterior estimates with credible intervals for avoidance tendencies regressed on perceived descriptive inclusivity for each country (*b* = 0.00 to *b* = 0.16; 95% CI [−0.10, 0.11] to 95% CI [0.06, 0.26]). d) Posterior estimates with credible intervals for avoidance tendencies regressed on perceived injunctive inclusivity norms for each country (*b* = −0.04 to *b* = −0.16; 95% CI [−0.13, 0.06] to 95% CI [−0.26, −0.06]). e) Posterior estimates with credible intervals for cooperation willingness regressed on perceived descriptive inclusivity norms for each country (*b* = 0.02 to *b* = 0.13; 95% CI [−0.06, 0.10] to 95% CI [0.04, 0.22]). f) Posterior estimates with credible intervals for cooperation willingness regressed on perceived injunctive inclusivity norms for each country (*b* = 0.02 to *b* = 0.25; 95% CI [−0.07, 0.11] to 95% CI [0.17, 0.34]).

Overall, the results provide evidence for an association between both perceptions of descriptive and injunctive inclusivity norms and tolerance, avoidance tendencies, and cooperation willingness in most countries. Figure 4 shows the results of the Bayesian regression analyses for each dependent variable across the 12 countries (Tables S16–S27). The associations of perceived injunctive inclusivity norms with the outcome variables are generally larger and more frequently significant than those of perceived descriptive inclusivity norms. However, in most cases, the difference in the strength of association between both types of social norms is not significant (Table S28). In nine out of the 12 countries (Finland, France, Germany, Hungary, Italy, The Netherlands, Poland, Spain, and Sweden), perceived injunctive inclusivity norms showed significant positive associations with tolerance, with posterior estimates ranging from *b* = 0.09 (95% CI [0.01, 0.17]) in Finland and Hungary to *b* = 0.19 (95% CI [0.11, 0.27]) in Germany. Descriptive inclusivity norms were significantly positively associated with tolerance in four out of the 12 countries (Czechia, Finland, Greece, and Italy), with posterior estimates ranging from *b* = 0.09 (95% CI [0.03, 0.16]) in Italy to *b* = 0.13 (95% CI [0.05, 0.21]) in Greece. For cooperation willingness, we found significant and positive associations with perceived injunctive inclusivity norms in 11 out of the 12 countries (all except Hungary), with posterior estimates ranging from *b* = 0.10 (95% CI [0.01, 0.19]) in Greece to *b* = 0.25 (95% CI [0.17, 0.34]) in Germany. Descriptive inclusivity norms showed significant positive associations with cooperation willingness in five out of the 12 countries (Czechia, Finland, Greece, Spain, and the United Kingdom), with posterior estimates ranging from *b* = 0.09 (95% CI [0.01, 0.17]) in Spain to *b* = 0.13 (95% CI [0.04, 0.22]) in Czechia, *b* = 0.13 (95% CI [0.03, 0.22]) in Finland, and *b* = 0.13 (95% CI [0.05, 0.22]) in the United Kingdom. In contrast, avoidance tendencies were significantly negatively related to perceived injunctive inclusivity norms in six out of 12 countries (Germany, Italy, The Netherlands, Poland, Spain, and Sweden), with posterior estimates ranging from *b* = −0.12 (95% CI [−0.22, −0.01]) in Germany and *b* = −0.12 (95% CI [−0.22, −0.02]) in Sweden to *b* = −0.17 (95% CI [−0.27, −0.06]) in Poland, *b* = −0.15 (95% CI [−0.26, −0.05]) in Spain, and *b* = −0.16 (95% CI [−0.26, −0.06]) in Finland. Only in two out of the 12 countries (Poland and Spain), associations between perceived descriptive inclusivity norms and avoidance tendencies were significant, and contrary to associations found for perceived injunctive inclusivity norms, positive, with *b* = 0.16 (95% CI [0.06, 0.25]) in Poland and *b* = 0.16 (95% CI [0.06, 0.26]) in Spain.

Notably, perceived injunctive and descriptive inclusivity norms are highly correlated in all country samples, with bivariate correlations ranging from *r* = 0.56 in Finland to *r* = 0.70 in Hungary and the United Kingdom (for bivariate correlations in all countries, see Tables S4–S15). These correlations demonstrate high overlap between the two types of norms with the likely consequence of underestimating their associations with the outcome variables when analyzed together. When examined separately (with all control variables included as before), the pattern of results is more consistent across all countries: both injunctive and descriptive inclusivity norms are significantly associated with tolerance and willingness to cooperate in all countries. For avoidance tendencies, the pattern remains less clear, with significant associations for injunctive norms only observed in Finland, Sweden, and the United Kingdom and for descriptive norms in Finland, Germany, and the United Kingdom (for all separate analyses, see Tables S79–S102).

Taken together, these results indicate that perceived inclusivity norms are associated with more tolerant and cooperative orientations across countries and, in most countries, lower avoidance tendencies. This pattern is particularly consistent for injunctive norms.

#### Normative alignment

To assess the strength of the combined influence of descriptive and injunctive inclusivity norms, we added their interaction to the model (Tables S16–S27). These analyses revealed that perceived descriptive norms interact with perceived injunctive norms only in some of the countries. For tolerance, we found a significant interaction between perceived descriptive and perceived injunctive norms in four out of the 12 countries (Finland, France, Hungary, and Italy), with posterior estimates ranging from *b* = 0.04 (95% CI [0.01, 0.08]) in Hungary to *b* = 0.07 (95% CI [0.02, 0.12]) in Finland. A similar pattern emerged for cooperation willingness, with significant interactions in four out of the 12 countries (Finland, France, The Netherlands, and Poland), ranging from *b* = 0.05 (95% CI [0.01, 0.09]) in Poland to *b* = 0.13 (95% CI [0.08, 0.18]) in The Netherlands. However, we found no credible evidence for the interaction between injunctive and descriptive inclusivity norms when avoidance was regressed on inclusivity norms, except for the Greek sample (*b* = 0.07; 95% CI [0.02, 0.12]). Overall, we found little evidence for stronger associations between both types of perceived inclusivity norms and the three outcome measures when both norms align.

#### Robustness checks

We explored the strength of disapproval of opposing opinions, respondents' opinion on the topic, levels of SDO and RWA, as well as relative group identification (i.e. difference score between identification with society and identification with the opinion-based ingroup) as potential boundary conditions of the relationship between the two types of social norms and the behavioral orientations.

First, we examined whether results varied depending on the degree of disapproval. The overall pattern for all outcome variables suggests no credible evidence that the strength of disapproval affects the associations between both types of inclusivity norms and the outcome measures (BF_01_ > 10) with few exceptions (Tables S29–S40). In case of significant interactions (five out of 36 cases), simple-slope analyses revealed that the relationship between perceived inclusivity norms and the outcome measures was particularly strong for people who indicated a “stronger” disapproval of the opposite opinion (for simple-slope plots, see Figures S2–S6).

Next, we tested whether the results varied depending on individuals' identification with their opinion-based ingroup relative to their identification with the superordinate societal group. Previous research consistently indicates that identification with the source of the normative information amplifies the impact of perceived social norms on individual behavior (73–76). However, stronger identification with the opinion-based ingroup is likely to weaken this association. This should particularly be the case when the contrast in strength of identification with society and the opinion-based ingroup is substantial. In other words, the association between perceived inclusivity norms and the outcome variables may be weakest among respondents who are more invested in (i.e. identified with) their opinion-based ingroup compared to society as a whole. To explore this possibility, we calculated a difference score between identification with the opinion-based group and identification with society. Higher values on this score reflect stronger relative identification with the opinion-based ingroup, allowing us to capture these nuanced dynamics of social identification.

The analyses revealed that there is no credible evidence for an interaction effect in most cases (Tables S41–S52). This suggests that the strength of the associations is independent of individuals' contrast in the strength of identification with the opinion-based group vs. society, providing evidence for the robustness of the importance of perceived inclusivity norms (BF_01_ > 10). The interaction was only significant in four out of 36 cases (for simple-slope plots, see Figures S7–S10). In these few cases, the associations between perceptions of inclusivity norms and the outcome variables were stronger for respondents who identified less with the opinion-based outgroup relative to society. The overall pattern of results remains the same when including identification with society and ingroup identification as separate moderators in the model instead of using the difference score (Tables S103–S126).

Finally, to better understand whether perceiving inclusivity norms is specific to individuals who already hold relatively egalitarian and pluralistic attitudes, we also tested whether results differed between respondents low versus high in SDO and RWA, as well as between those holding pluralist versus antipluralist opinions.

Including the interaction between each type of perceived inclusivity norms, SDO, and tolerance did not yield credible evidence in most countries (BF_01_ = 0.56–340.37), suggesting that the associations between perceiving inclusivity norms and being tolerant of opinion differences do not seem to differ between individuals holding rather egalitarian versus anti-egalitarian attitudes. A similar pattern emerged for cooperation willingness (BF_01_ = 0.01–295.31). In the few cases where interaction did emerge, simple-slope analyses suggest that the associations with both tolerance and cooperation willingness are even stronger among individuals high in SDO (for simple-slope plots, see Figures S11 and S20–S21). For avoidance tendencies the pattern is less consistent (BF_01_ = 0.01–67.83). Specifically, we found significant interactions with SDO in seven of the 12 countries (Germany, Greece, Hungary, Italy, Spain, Sweden, and the United Kingdom), and simple-slope analyses indicated that the association between inclusivity norms and avoidance tended to be stronger among individuals high in SDO (for simple-slope plots, see Figures S13–S19), pointing to a possible backlash effect among these individuals when it comes to avoiding those thinking differently from them (Tables S53–S64).

Analyses involving RWA produced a broadly comparable picture: mostly no interactions and where interactions did occur, the direction resembled the pattern observed for SDO (Tables S65–S76; for simple slopes, see Figures S22–S26).

Given that respondents differed in their position on the chosen issues and such differences may influence the observed associations, we repeated the analyses separately for those holding a more conservative (including participants from all countries given the small sample sizes for each opinion group) and those holding a more progressive stance on the topics of migration policies, gender equality, and transgender rights. These issues were selected because opinions on them can be meaningfully distinguished into rather pluralist or progressive and antipluralist or conservative orientations. Thus, we tested whether participants' opinion on the topic moderated the associations between inclusivity norms and the outcome variables. We found no credible evidence for the interaction effect for the topic of migration policies and transgender rights (BF_01_ > 3), while the data provide weak (BF_01_ = 1.70) to substantial (BF_01_ > 3) evidence for the absence of an interaction effect in case of the issue of gender equality. Overall, these results indicate that the associations not only hold among participants already aligned with pluralist attitudes but also among those with antipluralist attitudes, hence underscoring the robustness of our findings (Tables S76–S78).

Taken together, these analyses indicate that associations between perceived inclusivity norms and individual orientations are largely robust across levels of disapproval, social identification, RWA, SDO, and issue positions, with only inconsistent evidence for interactions.

## Discussion

In the present research, we examined the potential of perceived societal inclusivity norms—social norms that underline the importance of equality-based respect, dialogue, and unity—to counter negative consequences of societal polarization both on the country level (i.e. lower social trust and lower perceived social cohesion) and on the individual level (i.e. less tolerance, avoidance, and unwillingness to cooperate with opposing others). Our results speak to this potential. The descriptive and exploratory analyses indicate that, on the country level, higher inclusivity norms are associated with greater social trust and cohesion. At the individual level, analyzing survey data from more than 12,000 individuals residing in 12 European countries (Czechia, Finland, France, Germany, Greece, Hungary, Italy, The Netherlands, Poland, Spain, Sweden, and the United Kingdom) and multiple societal topics, we find a generally consistent pattern of associations between perceived inclusivity norms and the considered individual orientations. Across most replications, stronger perceptions of inclusivity norms were linked to higher levels of tolerance, greater willingness to collaborate with individuals holding opposing or disapproved opinions, and lower tendencies to avoid them. Importantly, these associations emerged even among individuals who strongly disapprove of the opposite opinion, who identify more strongly with like-minded others than with society as a whole, who endorse rather antipluralist opinions or who favor group-based dominance over democratic pluralism, and who tend to adhere to authority and conform to traditional norms. While the strength and consistency of these associations partly varied across contexts and samples, this pattern highlights the robustness and the potential reach of inclusivity norms across wide segments of the population. Together, these findings give reasons to believe that social norms hold promise to offer a realistic and scalable avenue for future large-scale intervention efforts on fostering important civic attitudes and behaviors in polarized societal contexts.

Prior studies have proven effective in reducing affective polarization at the individual level through interventions such as direct intergroup contact and misperception corrections. Our research complements these approaches by demonstrating the potential of assessing and promoting perceptions of inclusivity norms as a supplementary strategy with assumingly long-lasting effects while directly targeting important attitudes and behaviors that are important for the functioning of democracies (77). Indeed, the results highlight that individuals' perceptions of what others do (i.e. descriptive norms) or expect them to do (i.e. injunctive norms) are related to their level of tolerance of and their willingness to cooperate with those individuals with opposing and disapproved-of views on important societal issues. We also found evidence, albeit limited, that the relationship between inclusivity norms and the individual orientations is strongest when perceptions of what others expect (i.e. injunctive norms) align with perceptions of what others actually do (i.e. descriptive norms). These results not only confirm prior findings on the potential of social norms (37–39) but also add to our understanding of how perceptions of specific social norms may work in polarized contexts, thereby providing important insights for future research, interventions, and policies.

We used an adapted version of the least-liked group approach to ensure that the topic of reference was genuinely relevant to respondents, that they had an established opinion on the issue, and that they had some level of disapproval for the opposing view. This approach offers several advantages over others for assessing outcome variables such as tolerance, avoidance tendencies, and cooperation willingness toward disliked opinion-based outgroups. Notably, it avoids assumptions about which issues matter to respondents, ensuring that opposing opinions are in fact met with disapproval.

We acknowledge that our research has several limitations. First, our results are based on cross-sectional survey data, which do not permit causal inferences. However, in a series of three experimental studies in another line of our project, we found experimental evidence that manipulating perceptions of equality-based respect can increase tolerance in polarized contexts. In line with the present study, these experiments used opinion-based groups defined by opposing views on societal topics relevant to participants, allowing us to examine tolerance toward those with disapproved opinions in a similarly polarized context (78). These findings align with prior evidence on the effect of perceived social norms on conciliatory behaviors in conflictual intergroup settings (47, 50). Nevertheless, more research that employs longitudinal or experimental designs to test the causal effect of perceived inclusivity norms on tolerance, contact willingness, and cooperation is needed.

Second, our individual orientations measured behavioral intentions instead of actual behavior. Self-reported measures may hold the risk of overestimating levels of tolerance, non-avoidance, and cooperation due to social desirability biases or the relative ease of indicating such attitudes in hypothetical scenarios. As a result, the correlations with actual behavior may be weaker than those found here. While it is worth noting that a meta-analysis found substantial associations between intentions and behavior (79), we encourage future research to measure actual behavior rather than relying on self-reports to provide a more accurate understanding of the relationships between inclusivity norms and tolerance, avoidance, and cooperation.

Third, our study is limited to a European WEIRD (i.e. western, educated, industrialized, rich, and democratic) context, which constrains the generalizability of the findings to countries with different political systems or levels of democratization. However, the inclusion of European countries from the North, East, South, and West adds robustness to our results and gives reason to believe that they may hold across various political and cultural settings within this region. Future research would benefit from expanding the scope of this study to test and compare the associations between inclusivity norms and attitudes and behaviors that support the functioning of democracy outside of Europe and contribute to a better understanding of their applicability in other global contexts.

Despite these potential limitations, our study shows consistency across 12 European countries, with robustness tests indicating that findings hold even for individuals who are typically hard to reach in polarized contexts (e.g. those with strong disapproval, those holding antipluralist opinions, and those with a strong ingroup identification), while also addressing key aspects of opinion-based polarization and individual orientations supporting democracy (tolerance, avoidance, and cooperation with disapproved others) through an adapted version of the least-liked group approach. In summary, our findings provide initial insights into an alternative and potentially scalable approach to address the negative consequences of societal polarization. This not only provides a foundation for future research but also points to the policy potential of inclusivity norms, because they can be promoted in many contexts such as schools, communities, and society at large (80). The results suggest that perceptions of social norms emphasizing the treatment of others as equals, engagement in open dialogue, and a shared superordinate identity may play an important role in sustaining democracy, as they are linked to social cohesion at the societal level and tolerance, less avoidance, and higher willingness to cooperate at the individual level—patterns consistent with the idea that social norms act as a social “glue” in democratic societies.

## Materials and methods

### Design, sample, and data collection

We used cross-sectional survey data collected as part of a larger research project (INCLUSIVITY) that was preregistered along with other subprojects on the Open Science Framework (OSF) on November 1, 2023 (https://osf.io/n7c4y/). For the cross-sectional survey, we aimed to sample ∼1,000 respondents aged 16–69 years from each of 12 European countries (Czechia, Finland, France, Germany, Greece, Hungary, Italy, The Netherlands, Poland, Spain, Sweden, and the United Kingdom) to include countries from North, West, South, and East Europe. Respondents were recruited by the German market research company INFO GmbH using online-access panels from the panel provider dynata. Participation in the survey was voluntary. All respondents provided informed consent prior to participation and received compensation for completing the survey. Data collection took place between November 9 and December 6 in 2023. While full representativeness cannot be guaranteed when using online-access panels, we used quota sampling to approximate the demographic composition based on Census data of all 12 European countries with regard to age, education, and gender (see Table S1 for more details of the sample compositions). In some countries, respondents with higher educational levels were overrepresented, and younger respondents from the age cohort of 16–29 years were underrepresented. To correct for deviations from the population proportions, the market research company INFO GmbH provided us with sampling weights based on census data from the different countries to account for these deviations.

### Data quality

To ensure high data quality, we conducted both cognitive and quantitative pretests for all study measures. The cognitive pretest included 18 interviews with German respondents to assess the comprehensibility of item formulation, whether the respondents were able to differentiate between the different components of perceived inclusivity norms, and whether respondents' understanding of the item content was in line with the theoretical conceptualization. Following these, we conducted quantitative pretests. Results of all the pretests show the psychometric reliability and validity of all measures. The survey was professionally translated into all relevant languages, and native-speaking scholars checked the translations to ensure accuracy (for more details of all validation steps, see Figure S1). The interview script and documentation of the cognitive pretest, psychometrics report of the quantitative pretest, and R scripts are provided on OSF (https://osf.io/pb3ar/). Notably, to enhance response pattern reliability, we implemented various forms of randomization throughout the survey, including item randomization and randomization of display order of questions with randomized item order (see Supplementary material section “Survey Questions and Order” for more details on specific randomized question sets and items).

### Ethical review

We obtained ethical approval for the study from the ethics committee of the University of Hagen (approval number EA_480_2022).

### Analytic procedure

All included measures are reported in detail in Table S2 and are available for closer examination in a separate document available on OSF (https://osf.io/pb3ar/). We performed confirmatory factor analyses (CFAs) for all scales with three or more items across all samples. Measurement invariance testing established scalar invariance for perceived inclusivity norms and metric invariance for the primary outcome variables. Before conducting multiple regression analyses, we checked assumptions for regression analysis thoroughly. We preregistered the analytic procedure on OSF, with minor deviations that do not affect the overall pattern of results. First, contrary to the preregistration, we did not differentiate between the three components of perceived inclusivity norms in our analysis, as CFAs indicated that they were inseparable and highly correlated. Consequently, we treated them as a single construct. Second, rather than assessing contact willingness using both approach and avoidance subscales, we excluded the items measuring approach tendencies from our analyses. We based this decision on their inseparability and high correlations with cooperation willingness, as indicated by CFAs. The results of all CFAs can be reviewed in detail in a separate document reporting psychometrics for all items and scale analyses on OSF (https://osf.io/pb3ar/). Third, we included the interaction between both types of inclusivity norms in our model as an additional exploratory analysis to test whether the associations between perceptions of inclusivity norms and the outcome variables are stronger when both types of inclusivity norms align. Due to the large number of indicators for both types of norms (in both cases, nine indicators) and the challenges of conducting moderation analyses within structural equation modeling (SEM) (81), we used multiple regression instead. However, the pattern of results using SEM with latent variables produced are comparable results. All analytic steps are fully reproducible, with the corresponding R script available on OSF.

## Supplementary Material

pgag087_Supplementary_Data

## Data Availability

All data, materials, and R scripts needed to replicate the results reported in this paper can be found at https://osf.io/pb3ar/.

## Preprints

This manuscript was posted as a preprint at https://osf.io/preprints/psyarxiv/9gh6k and https://egreta.uab.cat/ws/files/516278821/assbetincnortolcontcoppol.pdf.
