# Supplementary material for: Associations between inclusivity norms and tolerance, contact, and cooperation amid polarization: Evidence from 12 European countries
Source: PNAS Nexus. 2026 Mar 26;5(4):pgag087. doi: 10.1093/pnasnexus/pgag087 (PMC13069891; doi:10.1093/pnasnexus/pgag087)
Supplement: pgag087_Supplementary_Data [file pgag087_supplementary_data.pdf]

Supplementary Information for

Associations between inclusivity norms and tolerance, contact, and cooperation amid polarization: Evidence from 12 European countries

Laura F. Schäfer, Nicole Tausch, Marcin Bukowski, Eva Jaspers, Miranda J. Lubbers, Maarten van Zalk, Alejandro Ciordia, Anna Potoczek, Lucía Estevan-Reina, Maor Shani, Jan-Willem Simons, Maria-Therese Friehs, Dominika Gurbisz, Wilma M. Middendorf, Sarina J. Schäfer, Jil Ullenboom, Sylvie Graf, Mikael Hjerm, Chloé Lavest, Inga Jasinskaja-Lahti, Anna Kende, Katerina Petkanopoulou, Francesca Prati, Oliver Christ

Corresponding authors: Laura F. Schäfer and Oliver Christ

Email: laura.schaefer@fernuni-hagen.de; oliver.christ@fernuni-hagen.de

This PDF file includes:

Supplementary text

Figures S1 to S26

Tables S1 to S128

Table of Contents

Supplementary Information Text **7**
Data and Materials Availability 7
Data Cleaning 7
Data Weighting 7
Deviations from the Preregistration 7
Measures 7
Survey Questions and Order 8
Figures **27**
Data Validation Process 27
Fig. S1 27
Boundary conditions: Simple slopes 28
Fig. S2 28
Fig. S3 29
Fig. S4 30
Fig. S5 31
Fig. S6 32
Fig. S7 33
Fig. S8 34
Fig. S9 35
Fig. S10 36
Fig. S11 37
Fig. S12 38
Fig. S13 39
Fig. S14 40
Fig. S15 41
Fig. S16 42
Fig. S17 43
Fig. S18 44
Fig. S19 45
Fig. S20 46
Fig. S21 47
Fig. S22 48
Fig. S23 49
Fig. S24 50
Fig. S25 51
Fig. S26 52
Tables **53**
Sample Description 53

- Table S1 53
- Table S1A 55
- Table S1B 56

Constructs 57

- Table S2 57

Descriptives and Correlations across Countries 59

- Table S3 59

Descriptives and correlations by country 60

- Table S4 60
- Table S5 61
- Table S6 62
- Table S7 63
- Table S8 64
- Table S9 65
- Table S10 66
- Table S11 67
- Table S12 68
- Table S13 69
- Table S14 70
- Table S15 71

Individual-level analyses: Main analyses by country 72

- Table S16 72
- Table S17 73
- Table S18 74
- Table S19 75
- Table S20 76
- Table S21 77
- Table S22 78
- Table S23 79
- Table S24 80
- Table S25 81
- Table S26 82
- Table S27 83
- Table S28 84

Boundary Conditions 86

- Strength of Disapproval 86
- Table S29 86
- Table S30 88
- Table S31 90
- Table S32 92

Table S33 94
Table S34 96
Table S35 98
Table S36 100
Table S37 102
Table S38 104
Table S39 106
Table S40 108
Identification Difference Score 110
Table S41 110
Table S42 112
Table S43 114
Table S44 116
Table S45 118
Table S46 120
Table S47 122
Table S48 124
Table S49 126
Table S50 128
Table S51 130
Table S52 132
Social Dominance Orientation 134
Table S53 134
Table S54 136
Table S55 138
Table S56 140
Table S57 142
Table S58 144
Table S59 146
Table S60 148
Table S61 150
Table S62 152
Table S63 154
Table S64 156
Right-Wing Authoritarianism 158
Table S65 158
Table S66 160
Table S67 162
Table S68 164
Table S69 166

Table S70 168
Table S71 170
Table S72 172
Table S73 174
Table S74 176
Table S75 178
Table S76 180
Opinion on Polarized Issue 182
Table S76 182
Table S77 184
Table S78 186
Additional Analyses 188
Injunctive Inclusivity Norms 188
Table S79 188
Table S80 189
Table S81 190
Table S82 191
Table S83 192
Table S84 193
Table S85 194
Table S86 195
Table S87 196
Table S88 197
Table S89 198
Table S90 199
Descriptive Inclusivity Norms 200
Table S91 200
Table S92 201
Table S93 202
Table S94 203
Table S95 204
Table S96 205
Table S97 206
Table S98 207
Table S99 208
Table S100 209
Table S101 210
Table S102 211
Identification with Society 212
Table S103 212

Table S104	214
Table S105	216
Table S106	218
Table S107	220
Table S108	222
Table S109	224
Table S110	226
Table S111	228
Table S112	230
Table S113	232
Table S114	234
Identification with the Opinion-Based Group	236
Table S115	236
Table S116	238
Table S117	240
Table S118	242
Table S119	244
Table S120	246
Table S121	248
Table S122	250
Table S123	252
Table S124	254
Table S125	256
Table S126	258
Network Norms	260
Table S127	260
Table S128	261

Supplementary Information Text

Data and Materials Availability. The data and materials related to this study are available on the Open Science Framework (OSF) under the following link https://osf.io/pb3ar/.

Data Cleaning. To ensure data quality, manual and automated checks for data cleaning were combined. Initially, the dataset underwent a preliminary review to identify and exclude missing or incorrect entries. Outliers were removed based on a Speed Index threshold of 2.85 and response variance criteria to detect straightlining. Control questions assessed respondent attention, with incorrect answers leading to exclusion. Additionally, filter logic and response validity were examined, flagging implausible responses for potential exclusion. Automated quality checks were conducted using the ReDem-Ki tool, which evaluated responses based on time spent (Time-Score), response patterns in matrix questions (Item-Battery-Score), and plausibility of open-ended answers (Open-Ended-Score). These scores were weighted to generate an overall quality assessment, ensuring that only high-quality data were included in the final data set.

Data Weighting. Weights were calculated for each country by crossing age and gender as well as by level of education (primary, secondary, and tertiary level).The dataset includes two weighting variables: disproportional weight (adjustment of imbalances in the sample by applying weights to correct over-or under-represented groups, aiming to reflect actual population proportions accurately) and proportional weight (adjustment for the weights of each group directly according to their proportions in the population, ensuring that the sample reflects the population’s true distribution without additional correction for disproportionality).

Deviations from the Preregistration. The main differences between the analysis plan as filed in the preregistration (see https://osf.io/gda5u) and the publication are as follows. First, contrary to the preregistration, we did not differentiate between the three components of perceived inclusivity norms in our analysis, as Confirmatory Factor Analyses (CFAs) indicated that they were inseparable and highly correlated. Consequently, we treated them as a single construct. Second, rather than assessing contact willingness using both approach and avoidance subscales, we excluded the items measuring approach tendencies from our analyses. This decision was based on their inseparability and high correlations with cooperation willingness, as indicated by CFAs. All results of the CFAs can be reviewed in the psychometrics and scale analyses report and reproduced with the corresponding r-file provided on OSF (https://osf.io/pb3ar/). Third, we included the interaction between both types of inclusivity norms in our model as an additional exploratory analysis in order to test whether the associations between perceptions of inclusivity norms and the outcome variables is stronger when both types of inclusivity norms align. Due to the large number of indicators for both types of norms (in both cases 9 indicators) and the challenges of conducting moderation analyses within Structural Equation Modelling (SEM), we used multiple regression instead. However, pattern of results using SEM with latent variables produced comparable results. All analytic steps are fully reproducible, with the corresponding R script available on OSF (https://osf.io/pb3ar/).

Measures. The questionnaire was initially developed in English and reviewed by professional native English-speaking translators. It was then translated into the other languages by professional native-speaking translators, with subsequent revisions made by native-speaking scholars with expertise in this field of research. The questionnaire included additional measures not reported in the main article or supplementary material (a detailed documentation of all measures can be found here: (https://osf.io/pb3ar/).

Survey Questions and Order. Below, we provide all questions included in the survey, presented in the order in which they were administered to participants. To ensure a more reliable response pattern, randomization has been implemented in different ways throughout the survey (i.e., randomization of items, randomization of display order of questions; randomization of display order of questions with randomized order of items). Whenever one of these randomization methods has been applied to a question, this is indicated at the respective place in the report of the survey questions presented below.

Informed consent:

In this survey we would like to ask some questions that may be perceived as sensitive. Providing information in response to these questions is entirely voluntary and you may withdraw your consent at any time. The answers that you provide will be used only for market research analysis purposes.

For more information on how your information will be processed and protected, please review the privacy policy here: [Link to privacy policy Info GmbH]

Do you consent to the collection of this information?

Yes, I consent.

No, I do not consent.

Quota screening questions:

Gender:

How do you describe your gender?

Female

Male

Another gender identity

Non-binary

Another gender identity or multiple identifies, please state:

I do not wish to say

Age:

How old are you?

[open text] years

Education background:

What is your highest level of education you have completed?

[country-specific education levels; for more details see translation document: https://osf.io/pb3ar/]

Introduction questions

Introduction:

Values and beliefs are an essential part of our social lives. They serve as guidelines to describe what people consider to be correct, accurate, appropriate, important, or good. These can be religious beliefs as well as specific opinions on political or social issues.

Attention check:

Please briefly describe the hobbies you are most interested in and why you do them.

[open text]

Preceived value congruence (personal environment):

Take a moment to think about your personal environment (e.g. family, friends, and acquaintances). How many of the people in your personal circle have values and beliefs similar to yours?

Please indicate your answer on a scale from 0% (nobody) to 100% (everybody).

Perceived value congruence (society):

This question broadly pertains to the values and beliefs of other people in [country]. How many people in [coountry] have values and beliefs similar to yours?

Please indicate your answer on a scale from 0% (no one) to 100% (everyone).

Attention check:

A boy had seven marbles and lost one of them. How many marbles does he have now?

None more

1

2

3

4

5

6

7

Not sure

None of the above!

Inclusivitiy beliefs [Note: The following items are displayed in randomized order.]

Introduction:

In [country], people differ in terms of various characteristics. Despite this, some people still feel uniquely connected to one another. This may be due to a common faith, a common cultural and/or ethnic background, or even the fact that they share a particular opinion on social or political issues, for example. This means that everyone in [country] is able to feel like a member of different "social groups". The following questions refer to all people living together in [country], i.e., members of different social groups.

Please note that some of the questions may seem repetitive at first glance. So please pay close attention to which people are being asked about in each question.

Personal beliefs:

First, we are interested in your own opinion.

How much do you agree with the following statements about other people in [country], i.e., members of all social groups?

Please indicate your answer on a scale from 1 (strongly disagree) to 7 (strongly agree).

Equality-based respect:

(1) Everyone should always be treated as a human being of equal worth.
(2) All in all, everyone should be treated equally.
(3) Everyone should be recognized as a fellow citizen of equal worth.

Dialogue:

(4) We need to really listen to each other to better understand our differences.
(5) Everyone should be able to contribute so that something new and valuable can develop.
(6) Everyone's arguments should be considered so that we can learn from each other.

Unity:

(7) Despite the differences between the people in the UK, we are all part of a single community.
(8) Despite all our differences, we should feel united, and we have to face societal challenges together.
(9) Despite all the differences between people in the UK, we should feel that we are all part of the same group.

Moral foundations items:

(10)Everyone should show love for our country in their actions.
(11)Everyone should be loyal to our country.
(12)Everyone should abide by standards of purity and decency.

Inclusivitiy norms [Note: The following question sets are displayed in randomized order, including randomization of item order within each question set.]

Introduction – descriptive inclusivity norms:

The following questions are about <u>behaviour</u> towards members of other social groups (for example, people of different faiths, different cultural and ethnic backgrounds, or with varying opinions on social or political issues).

Perceived descriptive inclusivity norms (personal environment):

In the following questions, we are interested in how people in your <u>personal environment</u> (e.g., family, circle of friends, and acquaintances) <u>behave</u>. When answering these questions, please think mainly about the behaviour of the people surrounding you that you discuss important social issues with.

Please indicate on a scale of 1 (does not apply at all) to 7 (applies completely) to what extent the behaviors described apply to <u>most people in your personal environment</u>.

Most people in my personal environment ...

Equality-based respect:

(1) … always treat everyone as a human being of equal worth.
(2) … generally treat everyone equally.
(3) ... recognize everyone as a fellow citizen of equal worth.

Dialogue:

(4) ... really listen to everyone to better understand differences.
(5) ... allow everyone to contribute so that something new and valuable can develop.
(6) ... consider everyone's arguments to learn from each other.

Unity:

(7) ... support the view that despite the differences between the people in the UK, we are all part of a single community.
(8) ... express the feeling that despite all our differences, we are united, and we have to face societal challenges together.
(9) ... express the feeling that despite the differences between people in the UK, we are all part of the same group.

Moral foundations:

(10)... show love for our country in their actions.
(11)… are loyal to our country.
(12)... abide by standards of purity and decency.

Attention check:

(13)...please be sure to answer here with the 5th scale value

Perceived descriptive inclusivity norms (society):

The following questions are about the "current state" of society. This means that they are about how people generally behave in [country].

Please indicate on a scale of 1 (does not apply at all) to 7 (applies completely) to what extent the described behaviors apply to most people in [country].

Most people in [country]...

Equality-based respect:

(1) … always treat everyone as a human being of equal worth.
(2) … generally treat everyone equally.
(3) ... recognize everyone as a fellow citizen of equal worth.

Dialogue:

(4) ... really listen to everyone to better understand differences.
(5) ... allow everyone to contribute so that something new and valuable can develop.
(6) ... consider everyone's arguments to learn from each other.

Unity:

(7) ... support the view that despite the differences between the people in the UK, we are all part of a single community.
(8) ... express the feeling that despite all our differences, we are united, and we have to face societal challenges together.
(9) ... express the feeling that despite the differences between people in the UK, we are all part of the same group.

Moral foundations:

(10)... show love for our country in their actions.
(11)… are loyal to our country.
(12)... abide by standards of purity and decency.

Introduction – injunctive inclusivity norms:

The following questions deal with expectations about the way in which people from different social groups in the UK live together (for example, people of other faiths, of cultural and ethnic backgrounds, or even people with varying opinions on social or political issues).

Perceived injunctive inclusivity norms (personal environment):

The following questions are about expectations of people in your personal environment (e.g., family, circle of friends, and acquaintances). When answering these questions, please think particularly about the opinions of the people surrounding you that you discuss important social issues with.

What do people in your personal environment see as appropriate or correct when it comes to people living together in [country] society?

On a scale from 1 (does not apply at all) to 7 (applies completely), please indicate the extent to which the following statements reflect the opinion of most of the people in your personal environment

Most people in my personal environment believe that we should ...

Equality-based respect:

(1) … always treat everyone as a human being of equal worth.
(2) … generally treat everyone equally.
(3) ... recognize everyone as a fellow citizen of equal worth.
(4) ... really listen to everyone so that differences can be better understood.
(5) ... allow everyone to contribute so that something new and valuable can develop.
(6) ... consider everyone's arguments so that we can learn from each other.
(7) ... support the view that despite the differences between the people in the UK, we are all part of a single community.
(8) ... express the feeling that despite all our differences, we are united, and we have to face societal challenges together.
(9) ... express the feeling that despite the differences between people in the UK, we are all part of the same group.
(10)... show love for our country in our actions.
(11)... be loyal to our country.
(12)... abide by standards of purity and decency.

Perceived injunctive inclusivity norms (society):

In the following questions, we are interested in the <u>expectations</u> of <u>people in [country]</u>.

What do most people in [country] generally see as <u>appropriate or correct</u> when it comes to living together in [country] society?

On a scale from 1 (does not apply at all) to 7 (applies completely), please indicate the extent to which the following statements describe <u>most people's opinions in [country]</u>.

Most people in [country] believe that we should ...

Equality-based respect:

(1) … always treat everyone as a human being of equal worth.
(2) … generally treat everyone equally.
(3) ... recognize everyone as a fellow citizen of equal worth.

Dialogue:

(4) ... really listen to everyone so that differences can be better understood.
(5) ... allow everyone to contribute so that something new and valuable can develop.
(6) ... consider everyone's arguments so that we can learn from each other.

Unity:

(7) ... support the view that despite the differences between the people in the UK, we are all part of a single community.
(8) ... express the feeling that despite all our differences, we are united, and we have to face societal challenges together.

(9) ... express the feeling that despite the differences between people in the UK, we are all part of the same group.

Moral foundations:

(10)... show love for our country in our actions.
(11)... be loyal to our country.
(12)... abide by standards of purity and decency.

Attention check:

(13)...please be sure to answer here with the 3rd scale value.

Normative influence [Note: The following items are displayed in randomized order.]

In these questions, we are interested in how important other people's opinions are to you.

Please indicate on a scale from 1 (not at all important) to 7 (very important) how much you value each opinion.

How important are the following people's opinions to you?

(1) The opinion in your personal environment.
(2) The opinion of people in the UK.

Topic selection

In the UK, people have different opinions and feelings about current social and political issues. Please select the topic that you find easiest to take a clear position on in discussions.

Climate change

Migration and asylum policy

Gender equality

Transgender rights

Mandatory COVID-19 vaccination

Meat consumption

Opinion indication [Note: Participants were only presented with the respective question on the topic they had previously selected.]

Climate change:

The discussion about climate change often revolves around the question of whether climate change is man-made. Some people hold the opinion that the current climate change is mainly caused by

humans. Others believe that humans do not have much influence on the climate. The following question is about your opinion.

What is your opinion on the issue of climate change?

I believe that climate change is man-made.

I do not believe that climate change is man-made.

Migration and asylum policy:

People often use the terms "in favour of" or "against" when discussing migration and asylum matters such as accepting refugees. "In favour of" refers to the opinion that refugees should continue to be accepted into the country, and "against" refers to the opinion that no more refugees should be accepted in [country]. The following is about your opinion.

What is your opinion regarding the issue of accepting more refugees?

I am in favour of accepting more refugees in [country].

I am against accepting more refugees in [country].

Gender equality:

People often use the terms "in favour of" or "against" when discussing gender equality. "In favour of" refers to the opinion that measures should be taken to promote equal rights for women, and "against" refers to the opinion that no measures should be taken because gender equality has long been achieved. The following is about your opinion.

What is your opinion regarding the issue of gender equality?

I am in favour of measures to achieve equality between women and men.

I am against measures to achieve equality between women and men.

Transgender rights:

People often use the terms "in favour of" or "against" when discussing the concept of gender self-determination. "In favour of " refers to the opinion that it should be made easier for trans, intersex, and non-binary people to be able to change their legal gender accordingly to their self-determined gender identity, and "against" refers to the opinion that trans, intersex, and non-binary people should not be granted this right.

What is your opinion regarding the issue of gender self-determination?

I am in favour of gender self-determination.

I am against gender self-determination.

Mandatory Covid-19 vaccination:

People often use the terms "in favour of" or "against" when discussing whether the COVID-19 vaccination should be mandatory. "In favour of" refers to the position that the COVID-19 vaccine should be mandatory, and "against" refers to the position that the COVID-19 vaccine shouldbe an individual choice. The following is about your opinion.

What is your opinion regarding mandatory COVID-19 vaccination?

I am in favour of mandatory COVID-19 vaccination.

I am against mandatory COVID-19 vaccination.

Meat consumption:

People often use the terms "in favour of" or "against" when discussing the issue of reducing meat consumption. "In favour of" refers to the opinion that people should avoid meat (and other animal products), and "against" indicates that meat should be kept as part of a healthy diet. The following is about your opinion.

What is your opinion regarding the issue of reducing meat consumption?

I am in favour of reducing meat consumption.

I am against reducing meat consumption.

Control variables and potential moderators [Note: All of the following items are diaplayed in a randomized order.]

Extremity of the own opinion:

How strongly do you hold this opinion?

Importance of the topic:

How important is the issue of [chosen topic] to you personally?

Identification:

To what extent do you agree with the following statements?

I identify with people who [chosen opinion].

I feel strongly connected to people who [chosen opinion].

Strength of disapproval:

You have indicated that you [chosen opinion].

Some people may have a different opinion on this topic. How strong is your disapproval regarding the opinion that [opposite to chosen opinion]?

Dependent variables [Note: The following question sets are displayed in randomized order, including randomization of item order within each question set.]

Introduction:

The following questions deal with your attitude towards people who think differently from you about the issue of [chosen topic]. Please indicate the extent to which you agree with each statement about people who [opposite to chosen opinion] on a scale from 1 (strongly disagree) to 7 (strongly agree).

Perspective taking:

(1) I can see things from the point of view of these people.
(2) I do not waste my time listening to the arguments of these people.

Tolerance:

(3) These people should be able to stick to their opinion, even if others try to persuade them otherwise.
(4) These people should be allowed to speak their mind freely and openly.
(5) These people shall be allowed to meet in public spaces and give speeches.
(6) These people shall be given the chance to pursue their interests just like others.

Contact willingness:

(7) I am willing to get to know these people better.
(8) I am willing to engage in conversation with these people.
(9) I would rather have nothing to do with these people if I had a choice.
(10) I avoid contact with these people if I can.

Cooperation willingness:

(11) I would cooperate with these people to solve problems that affect all members of society.
(12) I am willing to meet with these people to discuss the concerns that affect all people in the UK.

Attention check:

(13) Please be sure to select the 6th scale value.

Outgroup attitudes:

More generally, how do you feel about people who [opposite to chosen opinion]?

[slider: from negative to positive]

[slider: from cold to warm]

Additional variables [Note: The following question sets are displayed in randomized order, including randomization of item order within each question set.]

Introduction:

The following questions relate to you and the society you live in.

Satisfaction with life:

All things considered, how satisfied are you with your life nowadays?

Social cohesion:

The following statements refer to people living together in [country].

Please indicate how much you agree with each statement on a scale of 1 (strongly disagree) to 7 (strongly agree).

(1) Social cohesion in [country] is at risk.
(2) In reality, most people do not care at all about what is happening to their fellow human beings.

Social trust:

The following questions deal with the topic of trust.

Please indicate your answer on a scale from 1 (do not trust at all) to 7(trust completely).

(1) How much do you trust the people in your personal environment?
(2) How much do you trust people in [country] in general?
(3) How much confidence do you have in the following institutions?
    (a) The British government
    (b) Governmental institutions
    (c) Most political parties
    (d) European institutions

Respect reciprocity [Note: The following question sets are displayed in randomized order, including randomization of item order within each question set.]

Introduction:

The following questions are about how <u>people like you behave</u>. This might be people who share your opinion concerning the issue of [chosen topic], for example.

Ingroup respect generality:

We are interested in how people like you behave towards other people in [country] in general, i.e., members of all social groups (for example, people of different faiths, cultural and ethnic backgrounds, or even people with different opinions on social or political issues).

Please indicate on a scale of 1 (does not apply at all) to 7 (applies completely) to what extent the described behaviors apply to people like you.

People like me ...

(1) … always treat everyone as a human being of equal worth.
(2) ... always communicate with others like they are a person of equal worth.
(3) … generally treat everyone equally.
(4) ... consistently treat everyone as a counterpart to be taken seriously.
(5) ... recognize everyone as a fellow citizen of equal worth.

Ingroup respect towards outgroup:

In this question, we are interested in how people like you behave, i.e., people who have the same opinion on the issue of [topic] as you, and how they behave toward people with a different opinion on the issue of [topic].

On a scale of 1 (does not apply at all) to 7 (applies completely), please indicate the extent to which the behaviors described apply to people like you.

People like me ...

(1) … always treat people who [opposite to chosen opinion] as a human being of equal worth.
(2) ... always communicate with people who [opposite to chosen opinion] like they are a person of equal worth.
(3) … generally treat people who [opposite to chosen opinion] equally.
(4) ... consistently treat people who [opposite to chosen opinion] as a counterpart to be taken seriously.
(5) ... recognize people who [opposite to chosen opinion] as fellow citizens of equal worth.

Outgroup respect generality:

The next questions are about how people who [opposite to chosen opinion] behave.

We are interested in how people who [opposite to chosen opinion] behave towards other people in [country], i.e., members of all social groups (for example, people of different faiths, cultural and ethnic backgrounds, or even people who have a different opinion on social or political issues).

On a scale of 1 (does not apply at all) to 7 (applies completely), please indicate the extent to which the behaviors described apply to people who [opposite to chosen opinion].

People who [opposite to chosen opinion] …

(1) … always treat everyone as a human being of equal worth.
(2) ... always communicate with others like they are a person of equal worth.
(3) … generally treat everyone equally.
(4) ... consistently treat everyone as a counterpart to be taken seriously.
(5) ... recognize everyone as a fellow citizen of equal worth.

Outgroup respect towards ingroup:

The following questions are about how <u>people who [opposite to chosen opinion] behave towards people like you</u> (for example, people who share your opinion regarding the issue of [chosen topic].

On a scale of 1 (does not apply at all) to 7 (applies completely), please indicate the extent to which the behaviors described apply to <u>people who [opposite to chosen opinion].</u>

People who [opposite to chosen opinion] ...

(1) … always treat people like me as a human being of equal worth.
(2) ... always communicate with people like me like they are a person of equal worth.
(3) … generally treat people like me equally.
(4) ... consistently treat people like me as a counterpart to be taken seriously.
(5) ... recognize people like me as fellow citizens of equal worth.

Perceived outgroup threat [Note: All of the following items are diaplayed in a randomized order.]

The following questions once again concern people who have different opinions to you on the issue of [chosen topic].

On a scale of 1 (does not apply at all) to 7 (applies completely), please answer to what extent the following statements apply to people who don't share your opinion on the issue of [chosen topic].

People who [opposite to chosen opinion] ...

(1) ... are dangerous for British society in general.
(2) ... are dangerous for the normal lives of people in [country].
(3) ... reduce my personal political freedom.
(4) ... would affect my personal safety if they gained power.
(5) ... are prepared to use violence against other people to achieve something they consider very important.
(6) ... are prepared to disrupt public order to achieve something they consider very important.

How likely is it that people who [opposite to chosen opinion] will gain a lot of power in [country] society?

How likely is it that people who [opposite to chosen opinion] will affect your or your family's quality of life?

Cultural tightness [Note: All of the following items are diaplayed in a randomized order.]

The following statements relate to [countr] as a whole.

Please indicate the extent to which you agree or disagree with the following statements on a scale from 1 (strongly disagree) to 7 (strongly agree).

Note that the statements sometimes refer to "social norms", i.e., rules of behavior that most people follow but are usually unwritten.

(1) There are many social norms people are supposed to abide by in this country.
(2) In this country, there are very clear expectations for how people should act in most situations.
(3) People agree on what behaviours are appropriate or inappropriate in certain situations in this country.
(4) People in this country have a great deal of freedom in how they want to behave in most situations.
(5) In this country, if someone acts in an inappropriate way, others will strongly disapprove.
(6) People in this country almost always comply with social norms.

Additional variables [Note: All of the following items are diaplayed in a randomized order.]

Introduction:

When answering the following questions, please consider your social position in [country] society.

Please indicate the extent to which the following statements apply to you on a scale of 1 (does not apply at all) to 7 (applies completely).

Self-respect:

(1) In everyday life I always see myself as a person with equal rights.
(2) When I look at myself, I see a person that is equal to others.

Identification with society:

(3) I identify with [country] society.

Spheres of control:

(4) I can usually achieve what I want if I work hard for it.
(5) Almost anything is possible for me if I really want it.
(6) I have no trouble making and keeping friends.
(7) If there is someone I want to meet, I can usually arrange it.
(8) By taking an active part in political and social affairs, we, the people, can influence world events.
(9) The average citizen can influence governmental decisions.

Social dominance orientation:

(10) An ideal society requires some groups to be on the top and others to be on the bottom.
(11) It is unjust to try to make groups equal.

Right wing authoritarianism:

(12) Rules in society should be enforced without compassion.
(13) People should leave important societal decisions to their leaders.
(14) Traditions should definitely be carried on and kept alive.

Attention check:

(15) Please select the 2nd scale value here as your answer.

Network questions

Please think again about the issue of [chosen topic].

Looking back over the last three months, have you talked about the issue of [chosen topic] with any of the following people? Please check all that apply.

My partner or spouse

My mother

My father

(One of) my children

(One of) my other relatives

(One of) my family-in-law

(One of) my friends

(One of) my co-workers

(One of) my neighbours

Person(s) from groups I am a member of (e.g., association, club, or place of worship)

Other acquaintance(s)

Person(s) on social media

With nobody

Looking back over the last three months, have you disagreed about the issue of [chosen topic] with any of the following people? Please check all that apply.

My partner or spouse

My mother

My father

(One of) my children

(One of) my other relatives

(One of) my family-in-law

(One of) my friends

(One of) my co-workers

(One of) my neighbours

Person(s) from groups I am a member of (e.g., association, club, or place of worship)

Other acquaintance(s)

Person(s) on social media

With nobody

Attention check:

If there were a television show or movie about you as a person, who would play the lead role and why?

Demographics

Introduction:

We would like to ask you to provide more details about yourself in order to complete the survey.

Sexual orientation:

What is the primary label you use to describe your sexual orientation?

heterosexual

Homosexual

bisexual

Pansexual

Asexual

Another, please state: [open text]

I do not wish to say

Nationality:

What is your nationality?

[country]

[country] and one other: [open text]

Non-[country], please state: [open text]

Migration history:

Did you immigrate to [country]?

Yes

No

Did one of your parents immigrate to [country]?

Yes

No

Subjective social status:

In our society, there are groups of people that tend to be at the top and those that tend to be at the bottom. We present a scale here that runs from top to bottom. If you think about yourself, where would you place yourself on this scale?

Religious denomination:

Do you feel you belong to a denomination?

Yes

No

I do not know

[if previous question answered with yes:]

Which denomination do you feel you belong to?

Christianity

Islam

Judaism

Hinduism

Buddhism

Other, namely: [open text]

Devoutness:

Regardless of whether you belong to a particular religion, how devout would you consider yourself to be?

Political orientation:

People often talk about "left" (0) and "right" (10) in politics. How would you classify your basic political stance?

Membership of discriminated minority group:

Would you describe yourself as a member of one or more groups that tend to be discriminated against in [country]?

Yes

No

I do not know

[if previous question answered with yes:]

On what grounds is your group discriminated against? Please check all that apply.

Skin color or ethnicity

Nationality

Religion

Language

Age

Gender

Sexuality

Disability

Other reasons, namely: [open text]

Network question/positive ties:

Who do you usually discuss personal matters with?

My partner or spouse

My mother

My father

(One of) my children

(One of) my other relatives

(One of) my family-in-law

(One of) my friends

(One of) my co-workers

(One of) my neighbours

Person(s) from groups I am a member of (e.g., association, club, or place of worship)

Other acquaintance(s)

Person(s) on social media

With nobody

Network question/negative ties:

Do you find any of the following people sometimes demanding or difficult? Please check all that apply.

My partner or spouse

My mother

My father

(One of) my children

(One of) my other relatives

(One of) my family-in-law

(One of) my friends

(One of) my co-workers

(One of) my neighbours

Person(s) from groups I am a member of (e.g., association, club, or place of worship)

Other acquaintance(s)

Person(s) on social media

Nobody

End of survey

We are at the end of the survey. Thank you very much for taking your time until the end and for supporting our study. Your information will of course be evaluated confidentially and anonymously and will be of great help to us. Thank you once again for this!

Figures

Data Validation Process

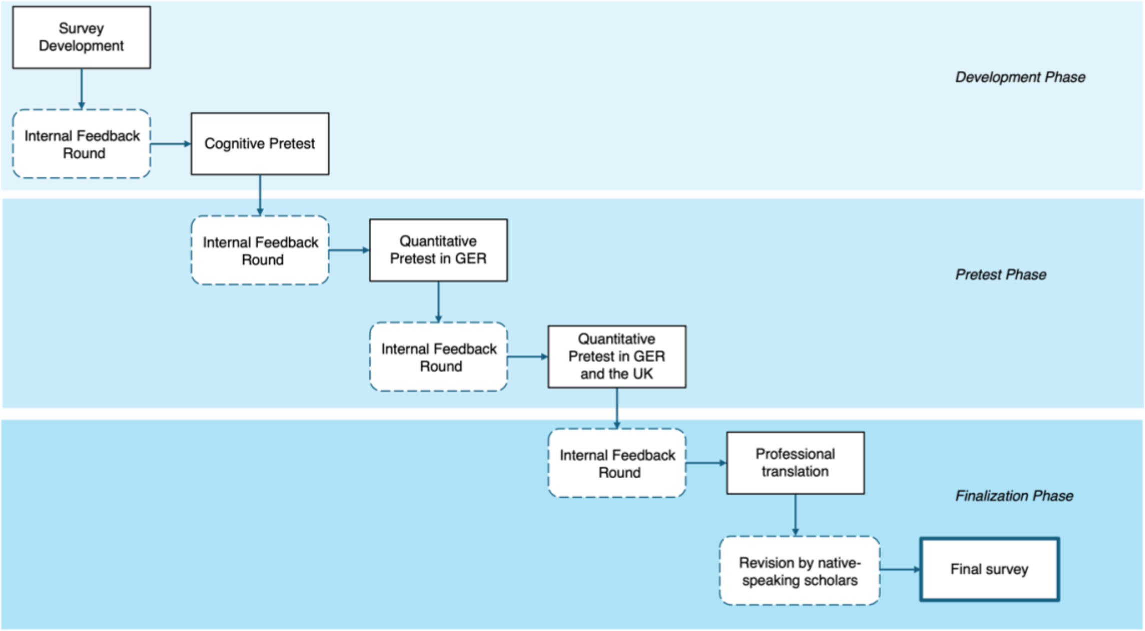

Fig. S1. Data Validation Process

Boundary conditions: Simple slopes

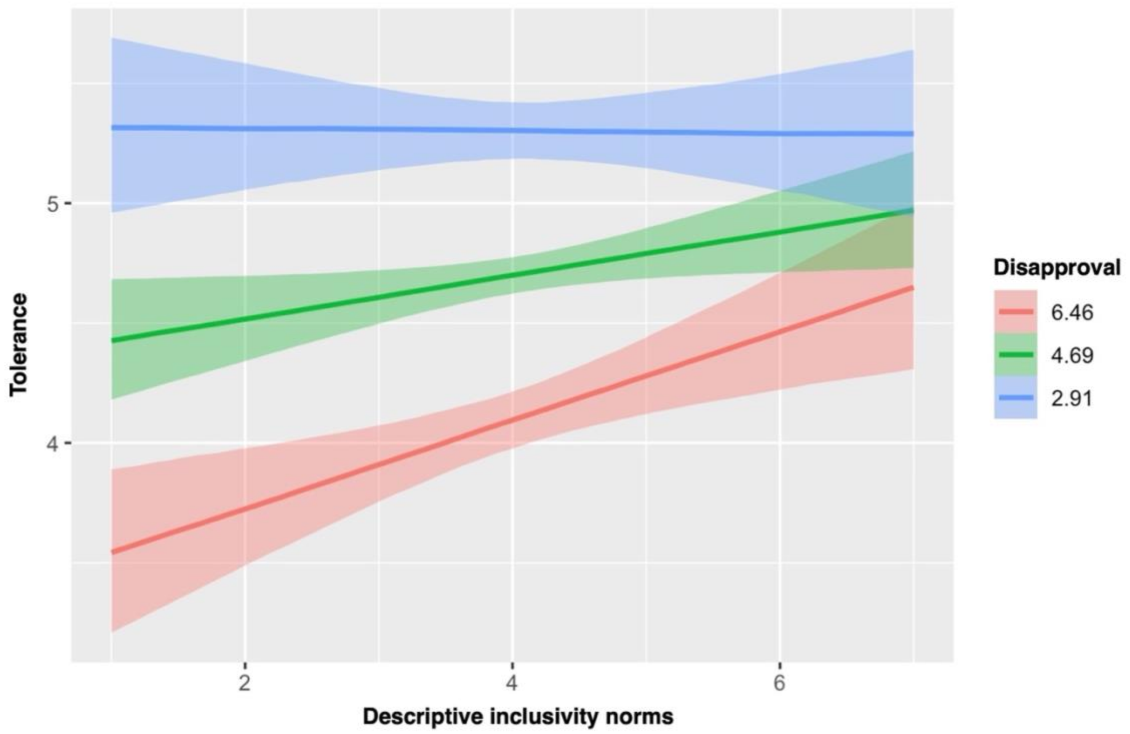

Fig. S2. Bayesian multiple regression slopes for the association between descriptive inclusivity norms and tolerance at the mean for disapproval, one standard deviation above the mean for disapproval, and one standard deviation below the mean for disapproval (Finnish sample).

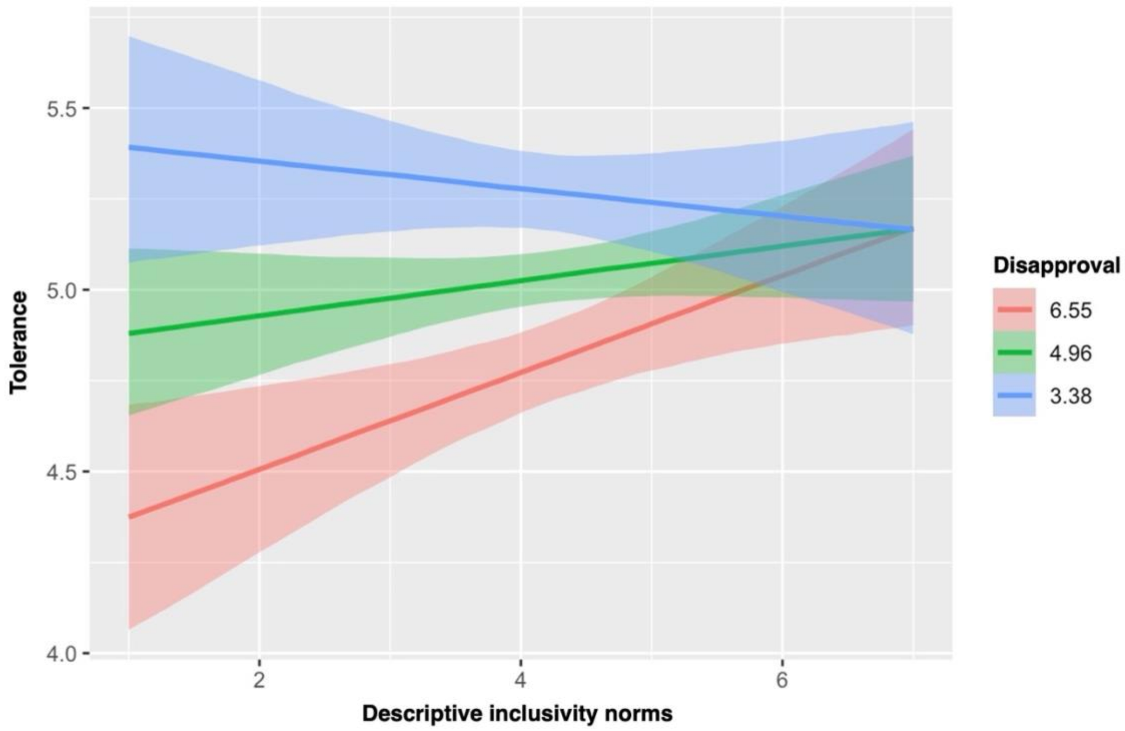

Fig. S3. Bayesian multiple regression slopes for the association between descriptive inclusivity norms and tolerance at the mean for disapproval, one standard deviation above the mean for disapproval, and one standard deviation below the mean for disapproval (German sample).

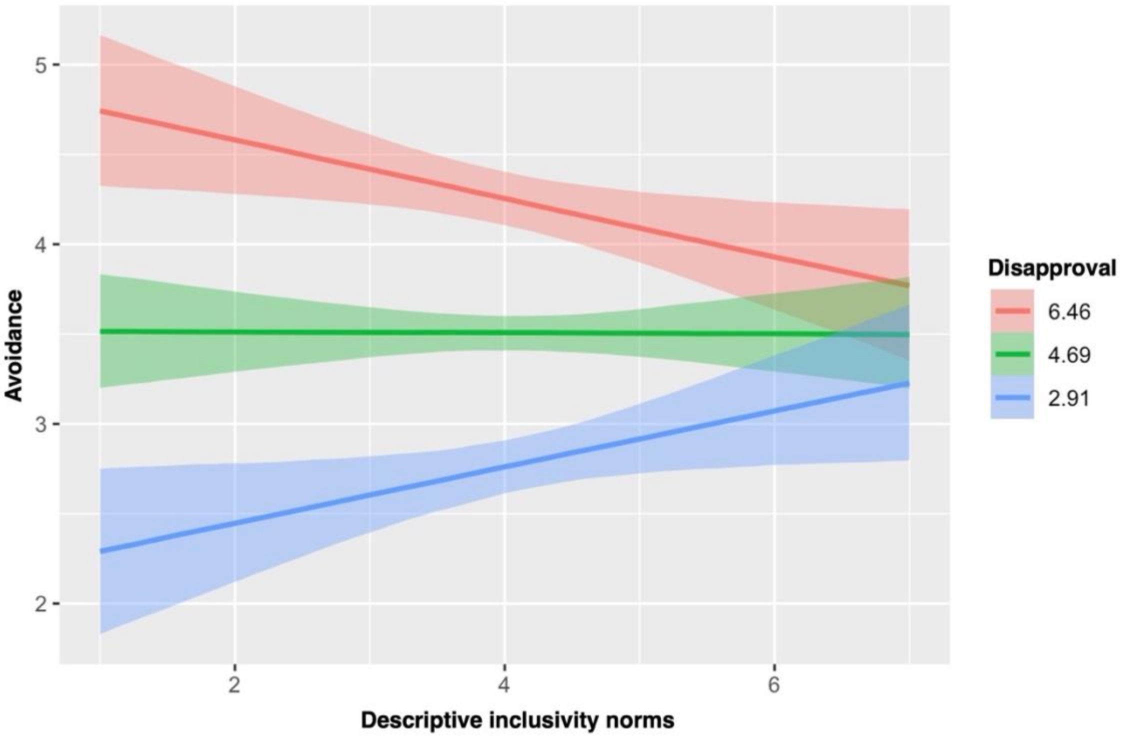

Fig. S4. Bayesian multiple regression slopes for the association between descriptive inclusivity norms and avoidance tendencies at the mean for disapproval, one standard deviation above the mean for disapproval, and one standard deviation below the mean for disapproval (Finnish sample).

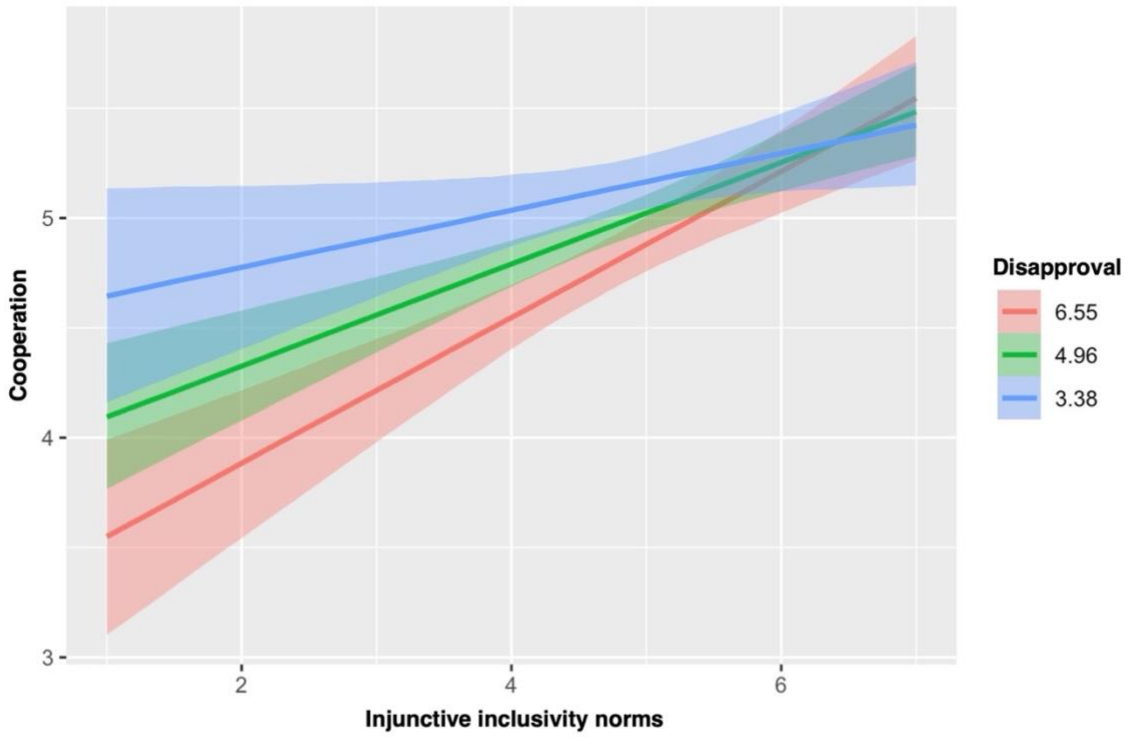

Fig. S5. Bayesian multiple regression slopes for the association between injunctive inclusivity norms and cooperation willingness at the mean for disapproval, one standard deviation above the mean for disapproval, and one standard deviation below the mean for disapproval (German sample).

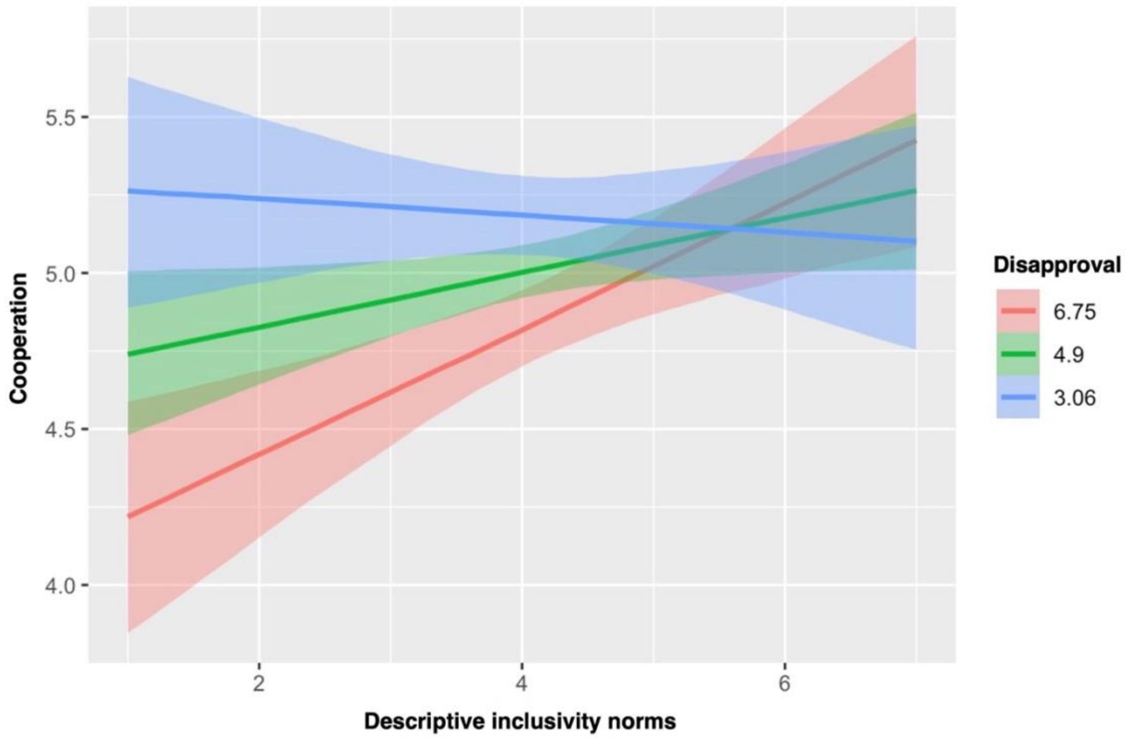

Fig. S6. Bayesian multiple regression slopes for the association between descriptive inclusivity norms and cooperation willingness at the mean for disapproval, one standard deviation above the mean for disapproval, and one standard deviation below the mean for disapproval (Spanish sample).

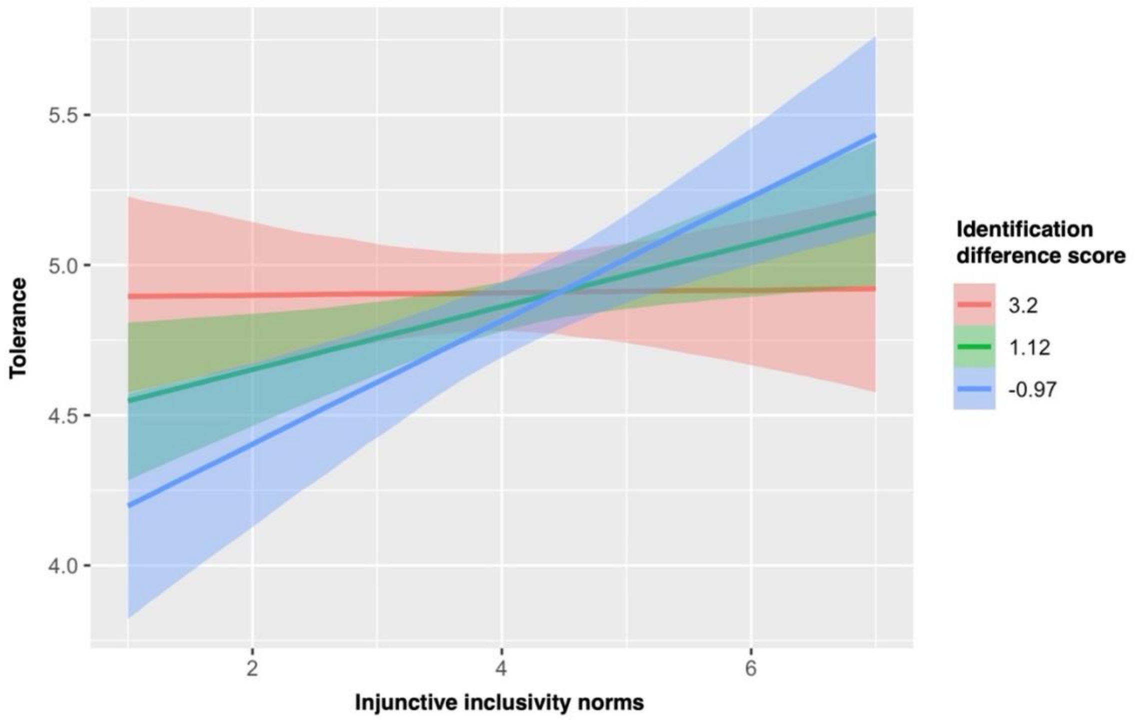

Fig. S7. Bayesian multiple regression slopes for the association between injunctive inclusivity norms and tolerance at the mean for the identification difference score, one standard deviation above the mean for the identification difference score, and one standard deviation below the mean for the identification difference score (Hungarian sample).

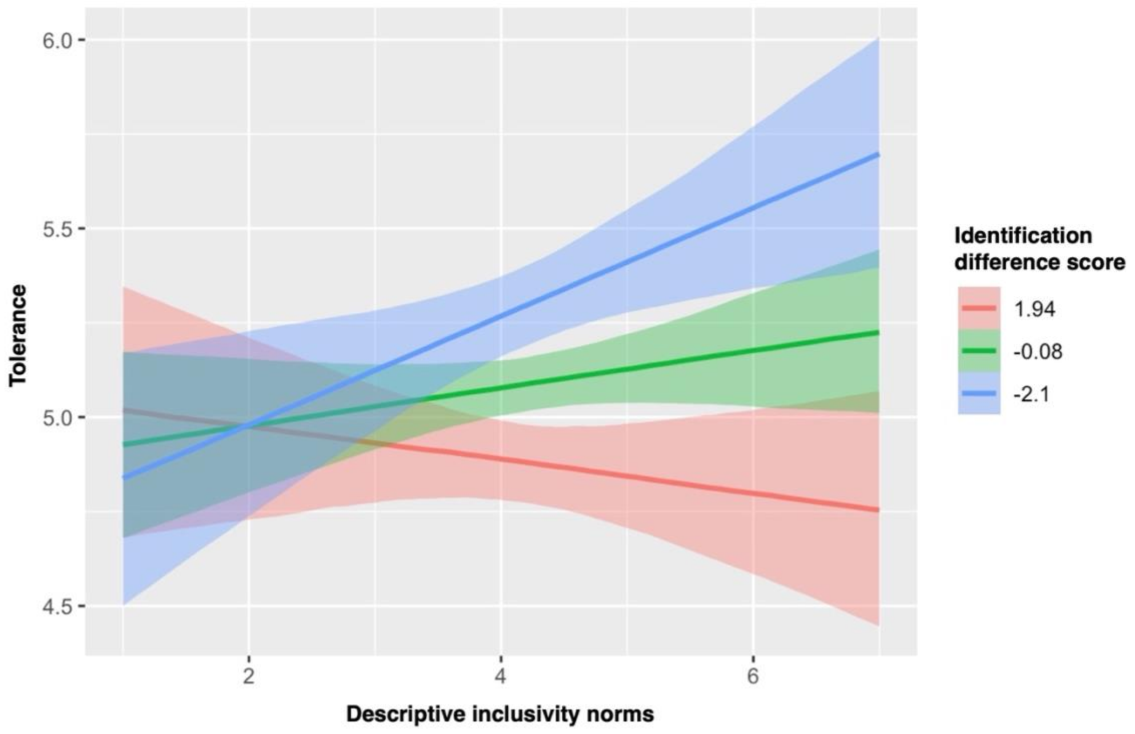

Fig. S8. Bayesian multiple regression slopes for the association between descriptive inclusivity norms and tolerance at the mean for the identification difference score, one standard deviation above the mean for the identification difference score, and one standard deviation below the mean for the identification difference score (Dutch sample).

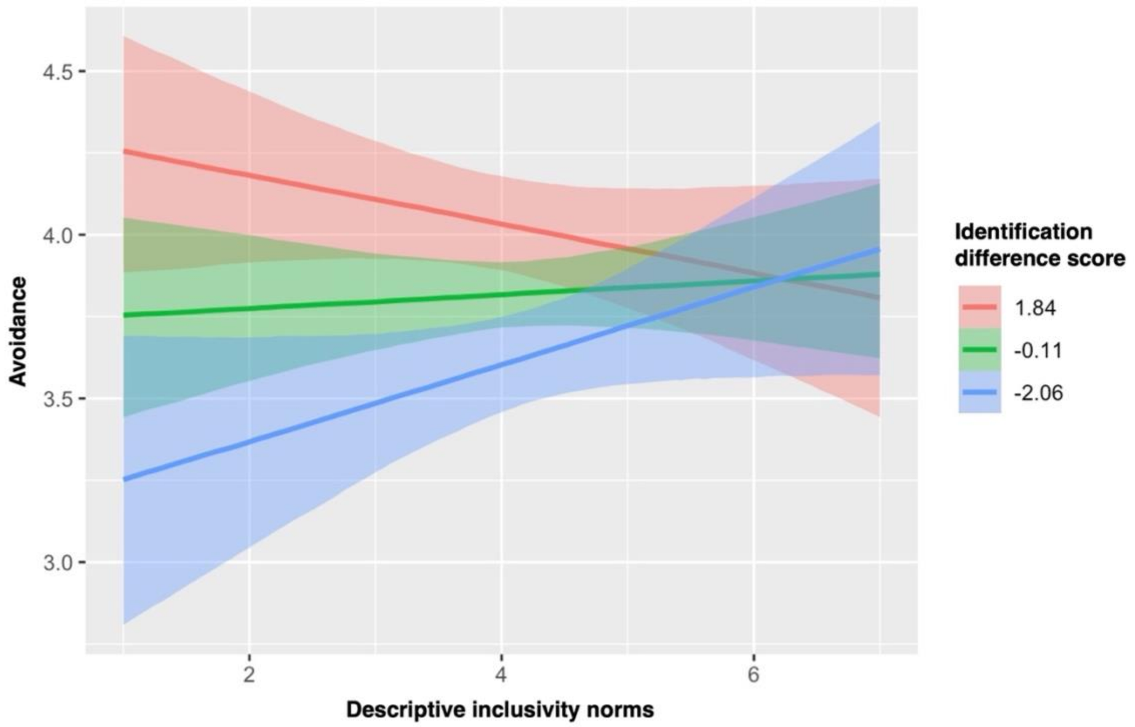

Fig. S9. Bayesian multiple regression slopes for the association between descriptive inclusivity norms and avoidance tendencies at the mean for the identification difference score, one standard deviation above the mean for the identification difference score, and one standard deviation below the mean for the identification difference score (Swedish sample).

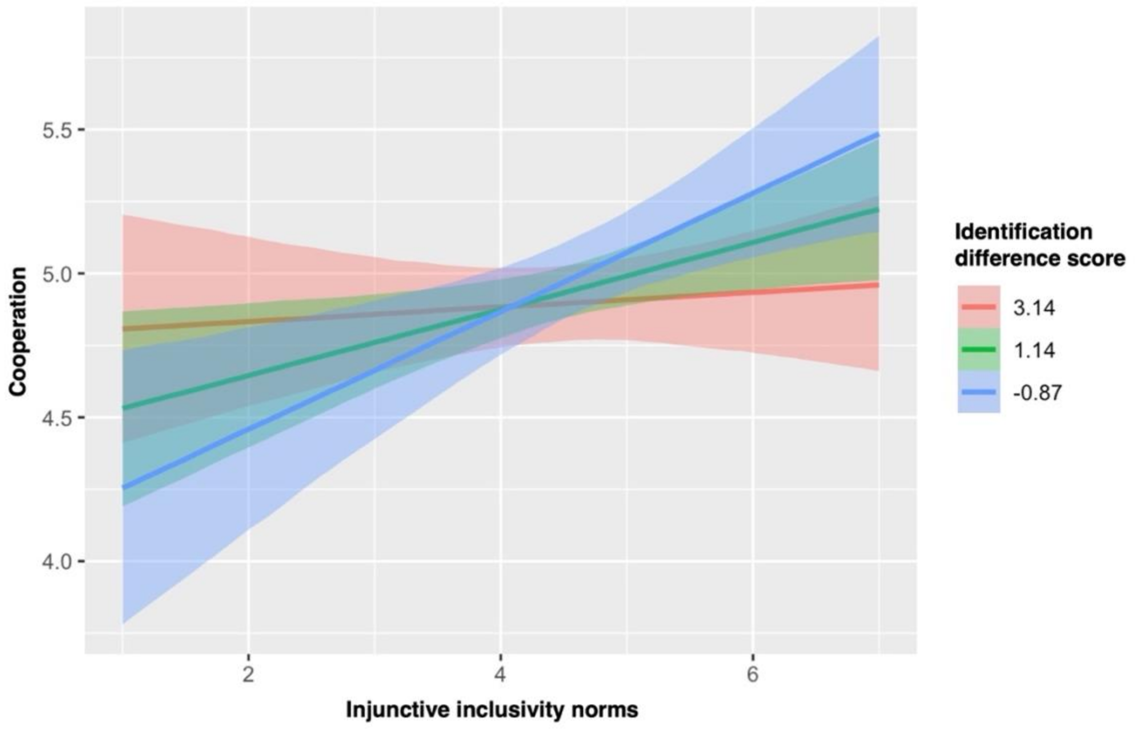

Fig. S10. Bayesian multiple regression slopes for the association between injunctive inclusivity norms and cooperation willingness at the mean for the identification difference score, one standard deviation above the mean for the identification difference score, and one standard deviation below the mean for the identification difference score (Greek sample).

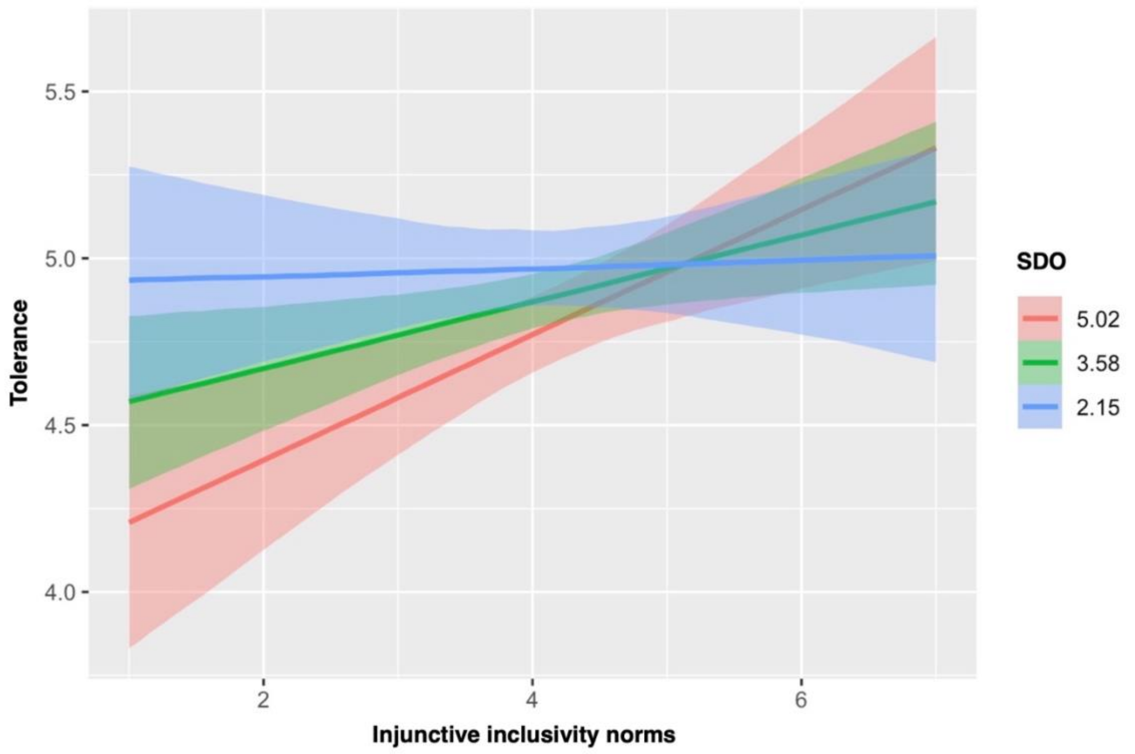

Fig. S11. Bayesian multiple regression slopes for the association between injunctive inclusivity norms and tolerance at the mean for social dominance orientation (SDO), one standard deviation above the mean for SDO, and one standard deviation below the mean for SDO (Hungarian sample).

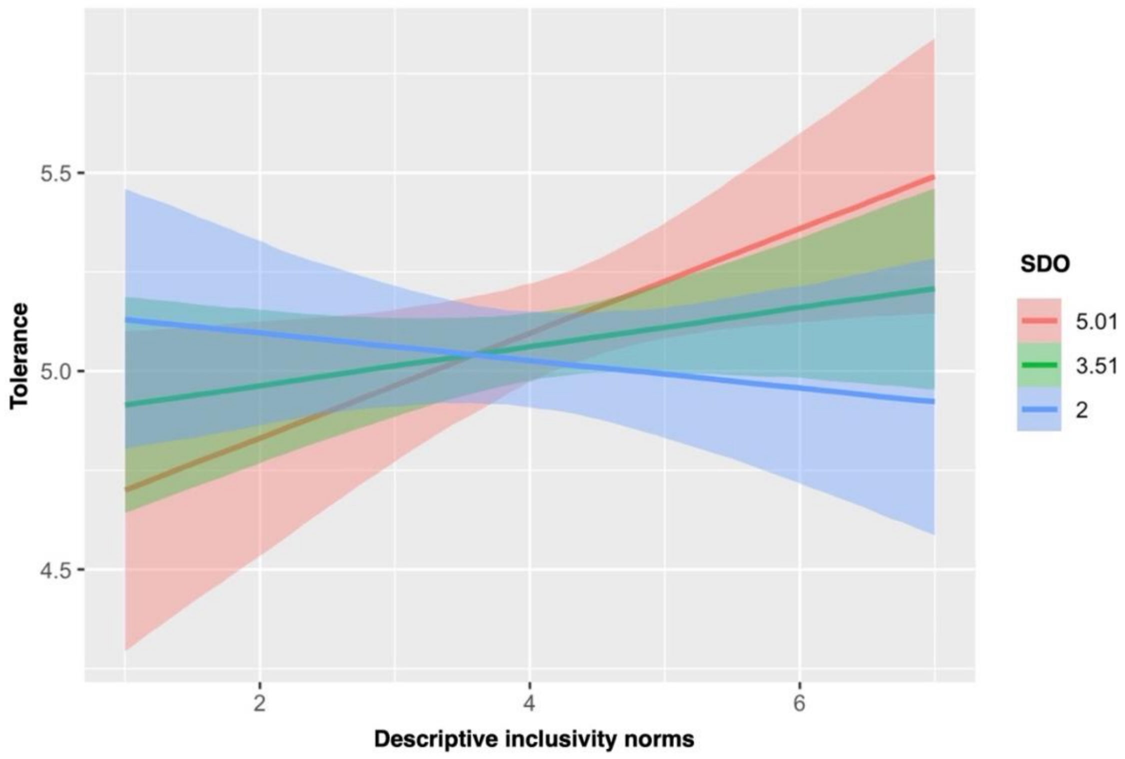

Fig. S12. Bayesian multiple regression slopes for the association between descriptive inclusivity norms and tolerance at the mean for social dominance orientation (SDO), one standard deviation above the mean for SDO, and one standard deviation below the mean for SDO (Spanish sample).

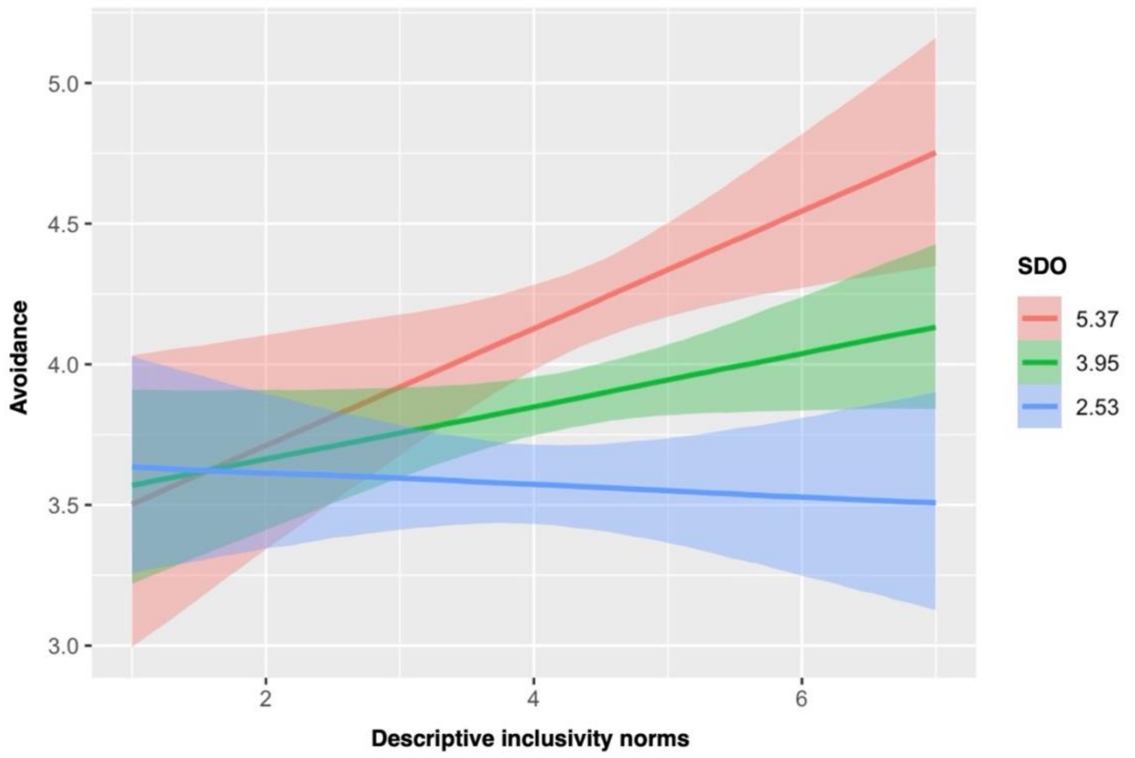

Fig. S13. Bayesian multiple regression slopes for the association between descriptive inclusivity norms and avoidance tendencies at the mean for social dominance orientation (SDO), one standard deviation above the mean for SDO, and one standard deviation below the mean for SDO (German sample).

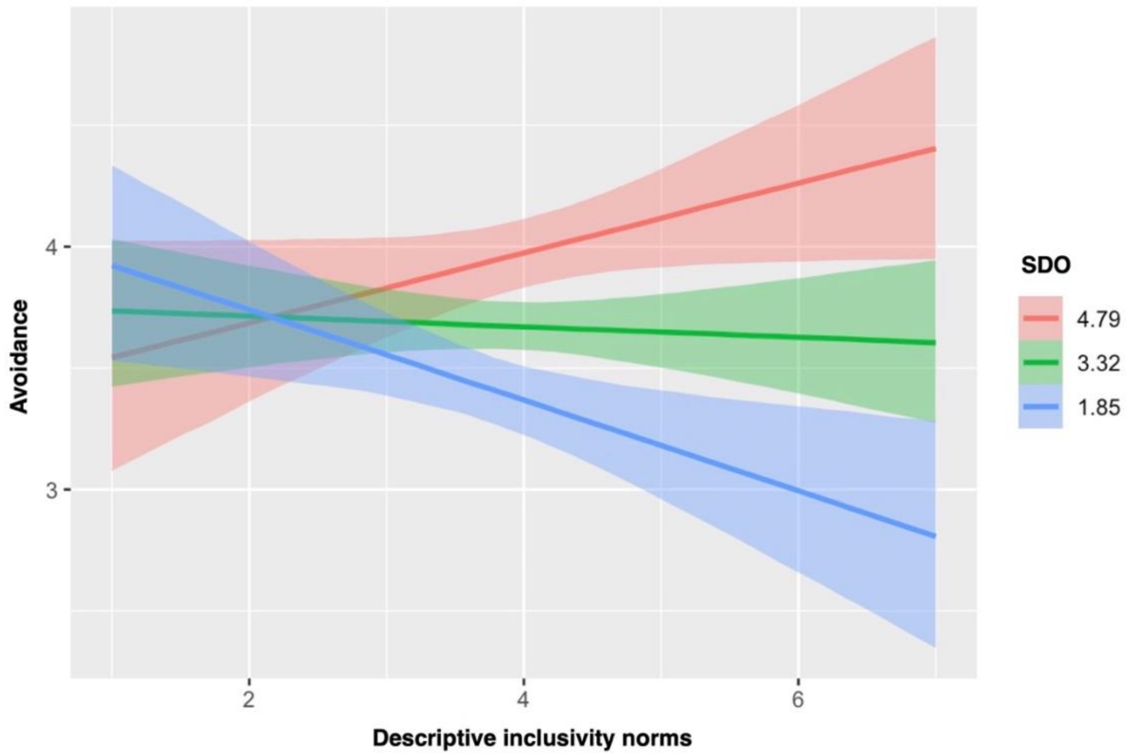

Fig. S14. Bayesian multiple regression slopes for the association between descriptive inclusivity norms and avoidance tendencies at the mean for social dominance orientation (SDO), one standard deviation above the mean for SDO, and one standard deviation below the mean for SDO (Greek sample).

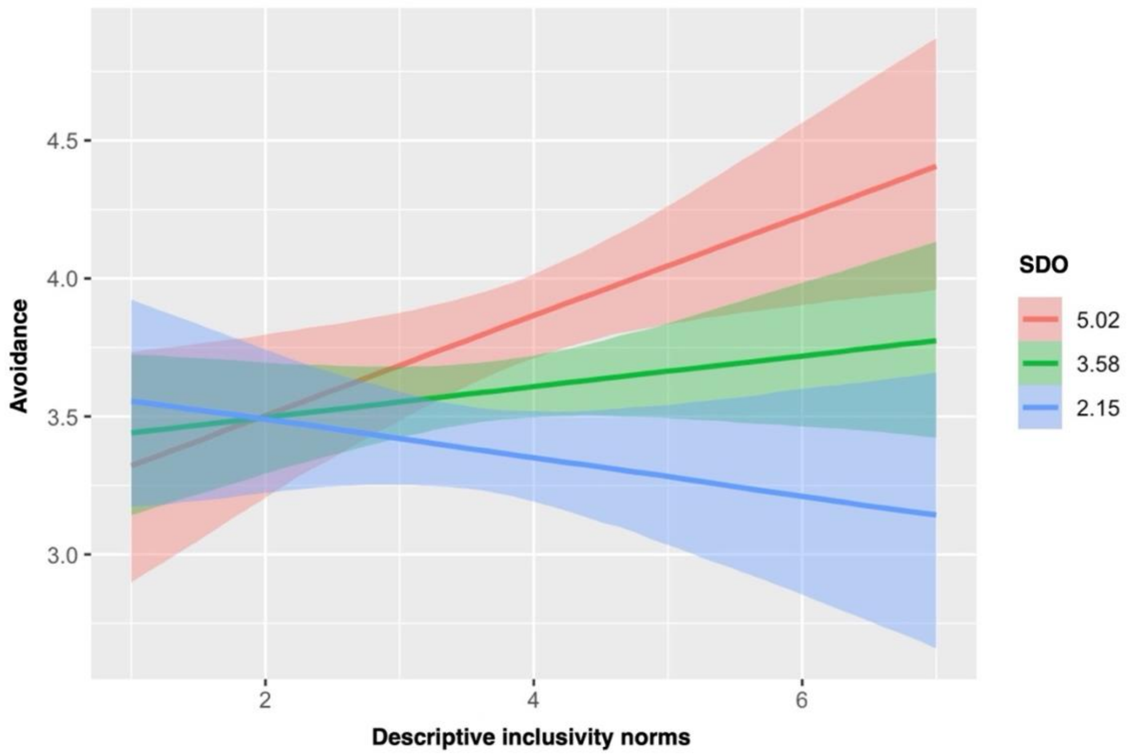

Fig. S15. Bayesian multiple regression slopes for the association between descriptive inclusivity norms and avoidance tendencies at the mean for social dominance orientation (SDO), one standard deviation above the mean for SDO, and one standard deviation below the mean for SDO (Hungarian sample).

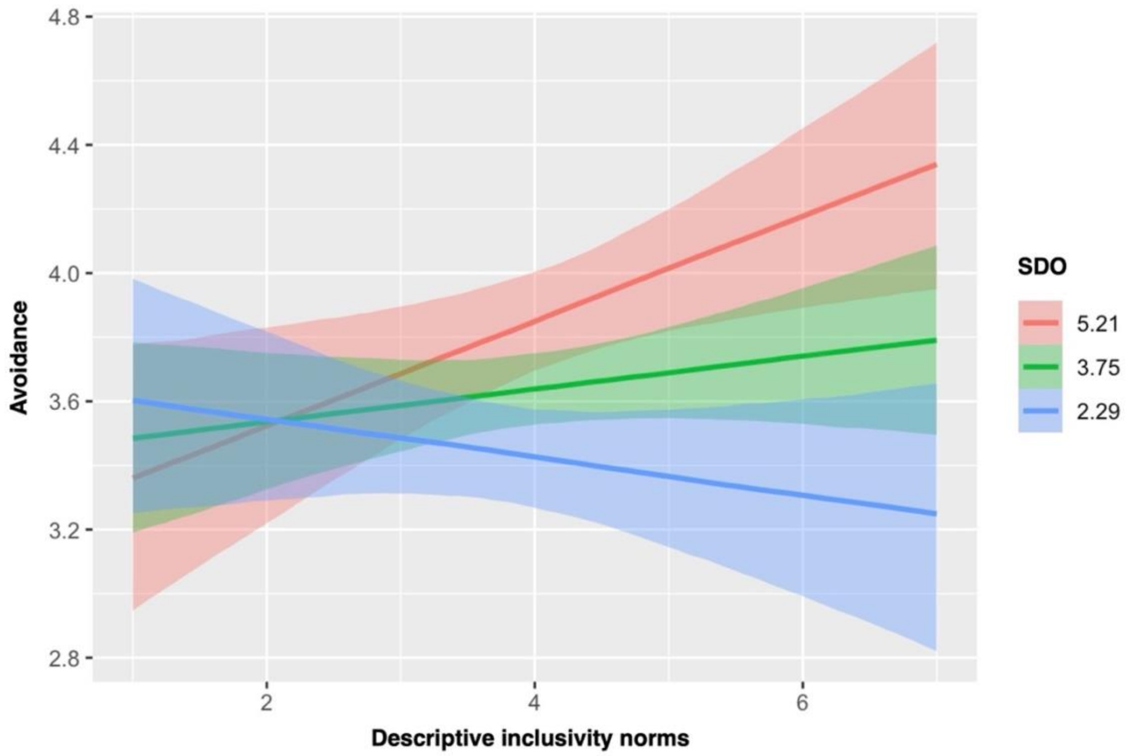

Fig. S16. Bayesian multiple regression slopes for the association between descriptive inclusivity norms and avoidance tendencies at the mean for social dominance orientation (SDO), one standard deviation above the mean for SDO, and one standard deviation below the mean for SDO (Italian sample).

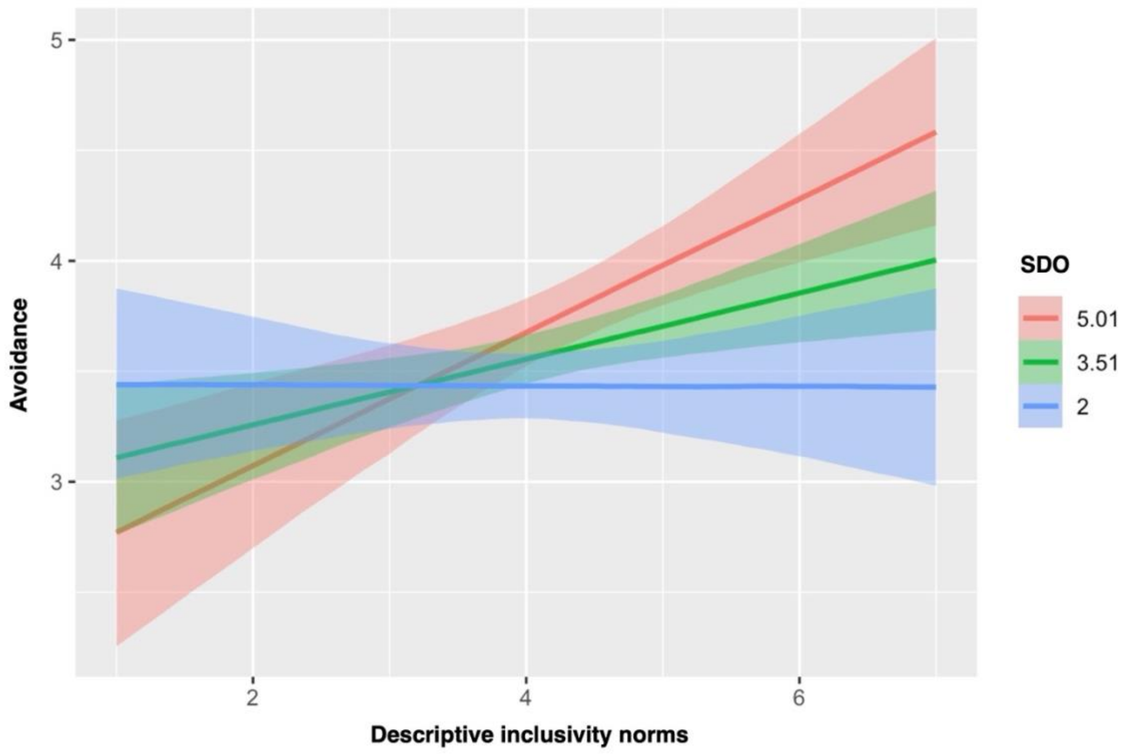

Fig. S17. Bayesian multiple regression slopes for the association between descriptive inclusivity norms and avoidance tendencies at the mean for social dominance orientation (SDO), one standard deviation above the mean for SDO, and one standard deviation below the mean for SDO (Spanish sample).

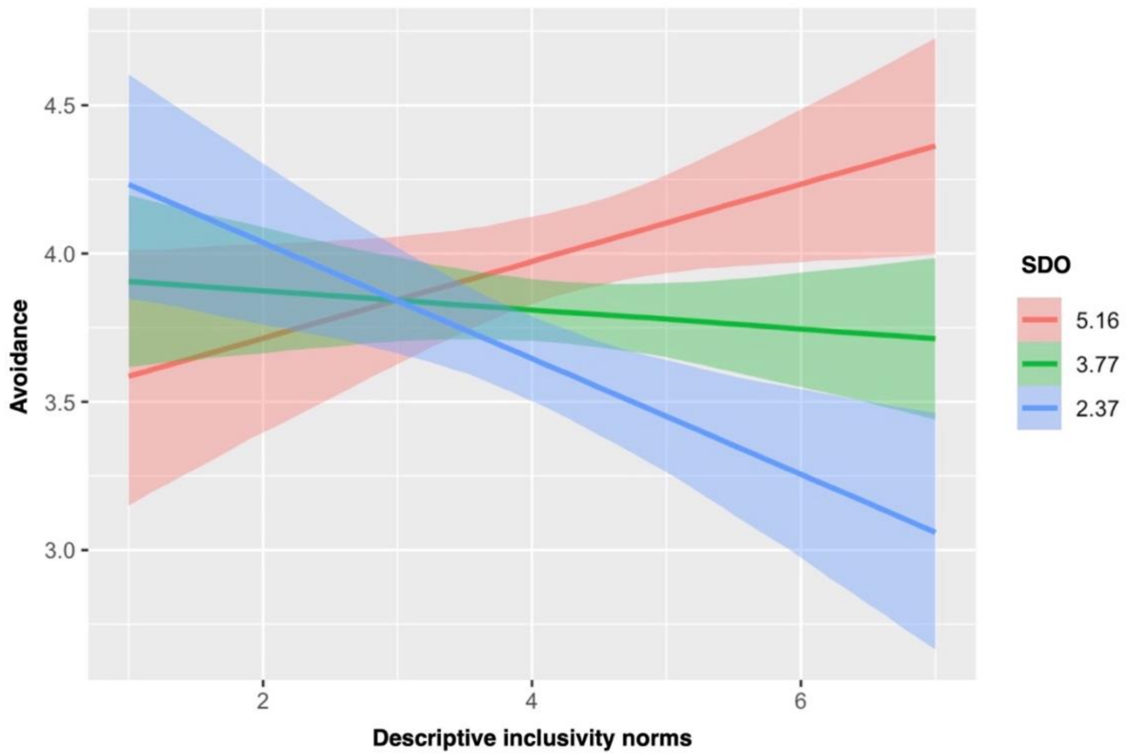

Fig. S18. Bayesian multiple regression slopes for the association between descriptive inclusivity norms and avoidance tendencies at the mean for social dominance orientation (SDO), one standard deviation above the mean for SDO, and one standard deviation below the mean for SDO (Swedish sample).

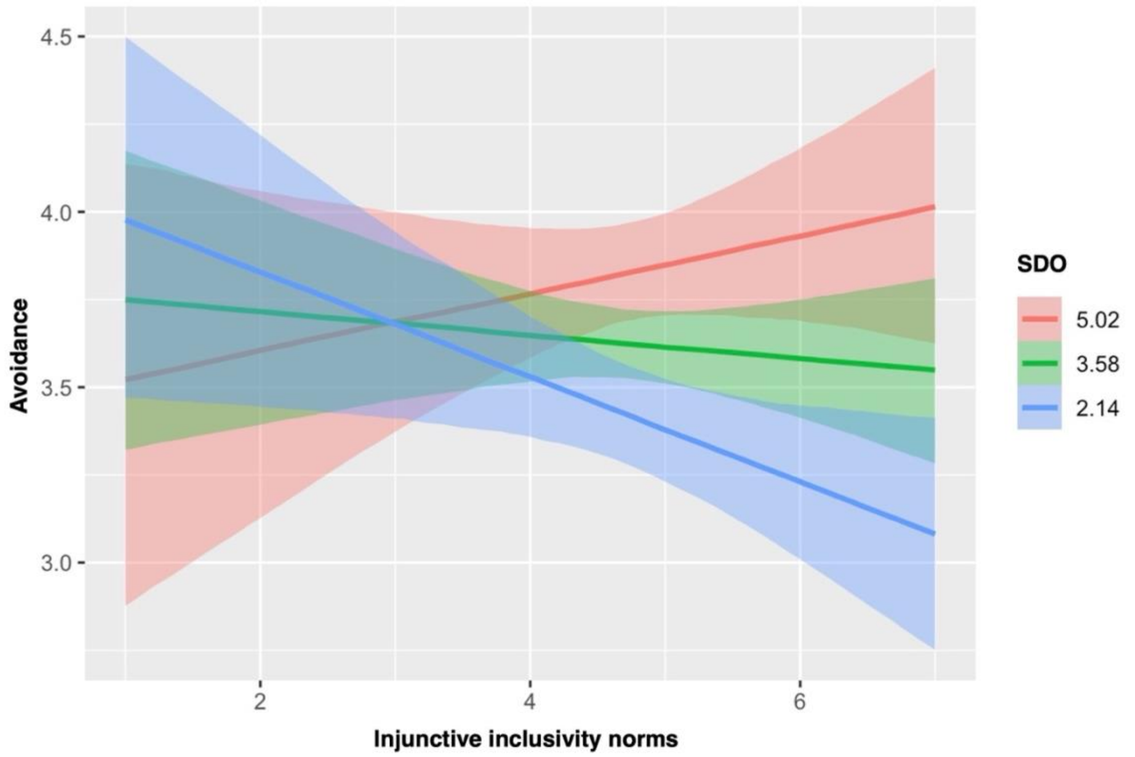

Fig. S19. Bayesian multiple regression slopes for the association between injunctive inclusivity norms and avoidance tendencies at the mean for social dominance orientation (SDO), one standard deviation above the mean for SDO, and one standard deviation below the mean for SDO (British sample).

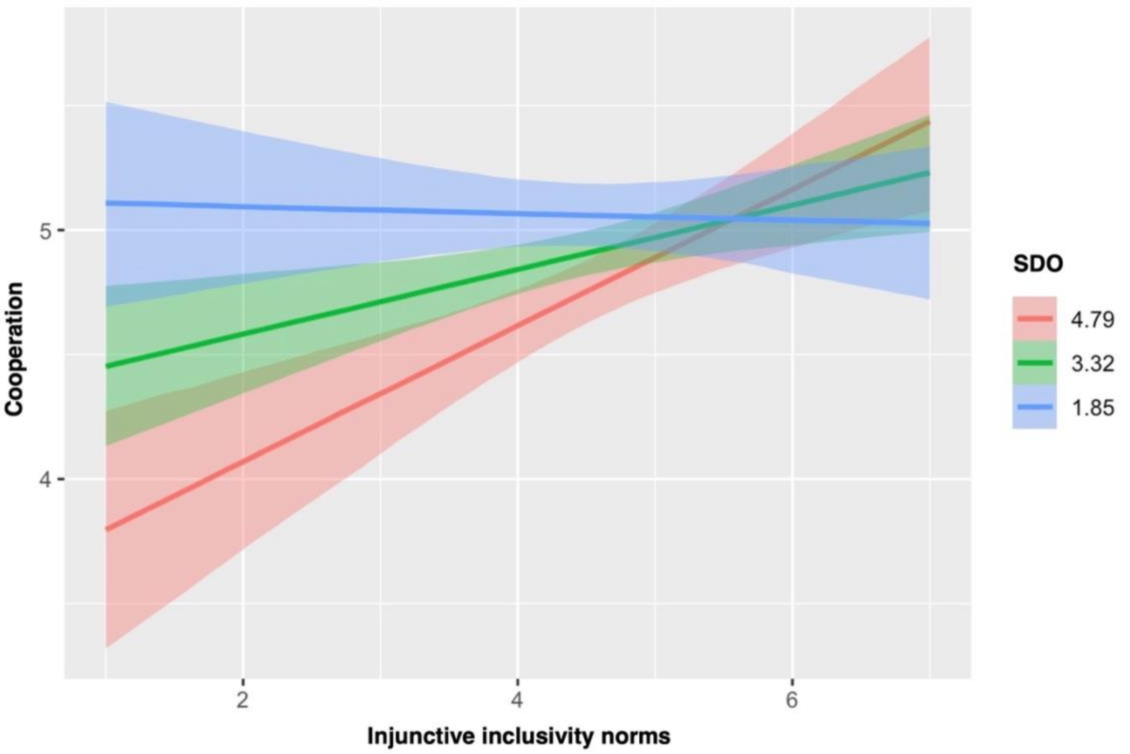

Fig. S20. Bayesian multiple regression slopes for the association between injunctive inclusivity norms and cooperation willingness at the mean for social dominance orientation (SDO), one standard deviation above the mean for SDO, and one standard deviation below the mean for SDO (Greek sample).

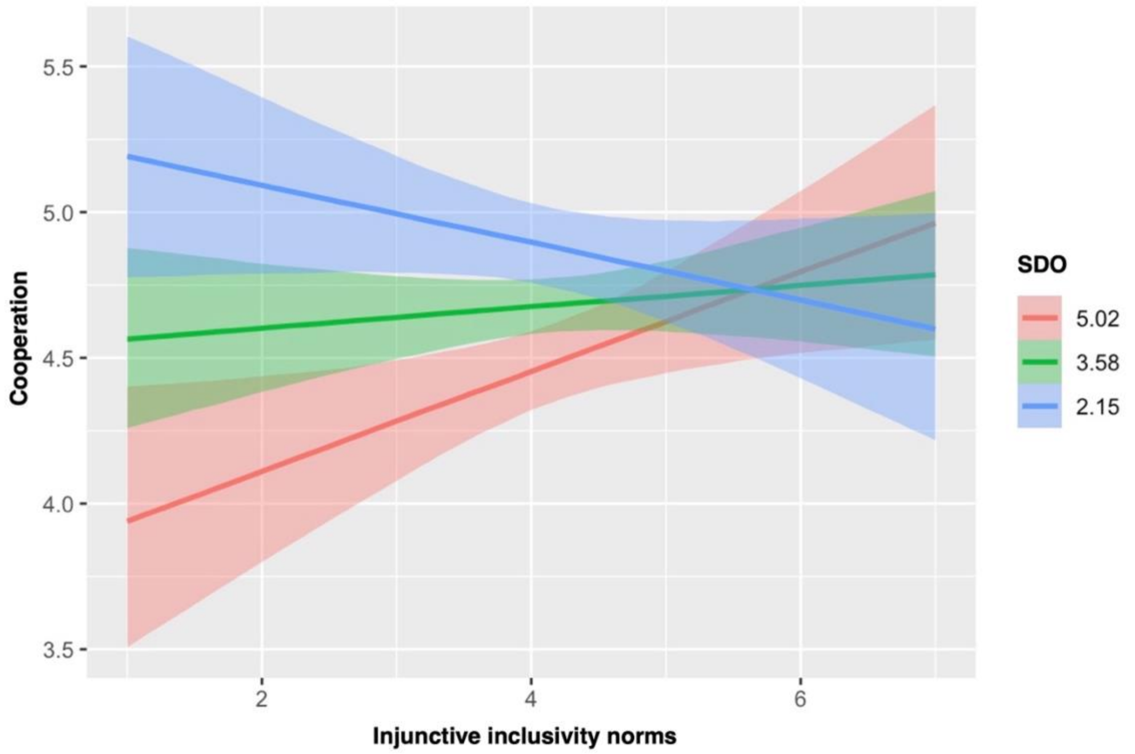

Fig. S21. Bayesian multiple regression slopes for the association between injunctive inclusivity norms and cooperation willingness at the mean for social dominance orientation (SDO), one standard deviation above the mean for SDO, and one standard deviation below the mean for SDO (Hungarian sample).

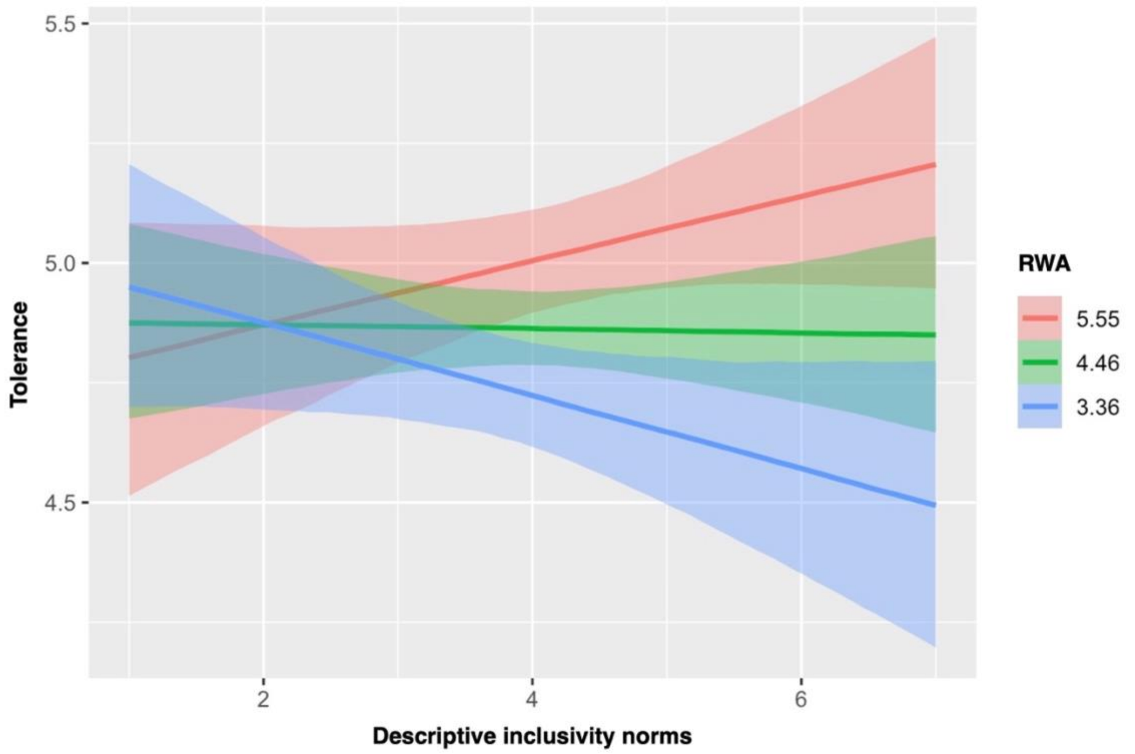

Fig. S22. Bayesian multiple regression slopes for the association between descriptive inclusivity norms and tolerance at the mean for right-wing authoritarianism (RWA), one standard deviation above the mean for RWA, and one standard deviation below the mean for RWA (French sample).

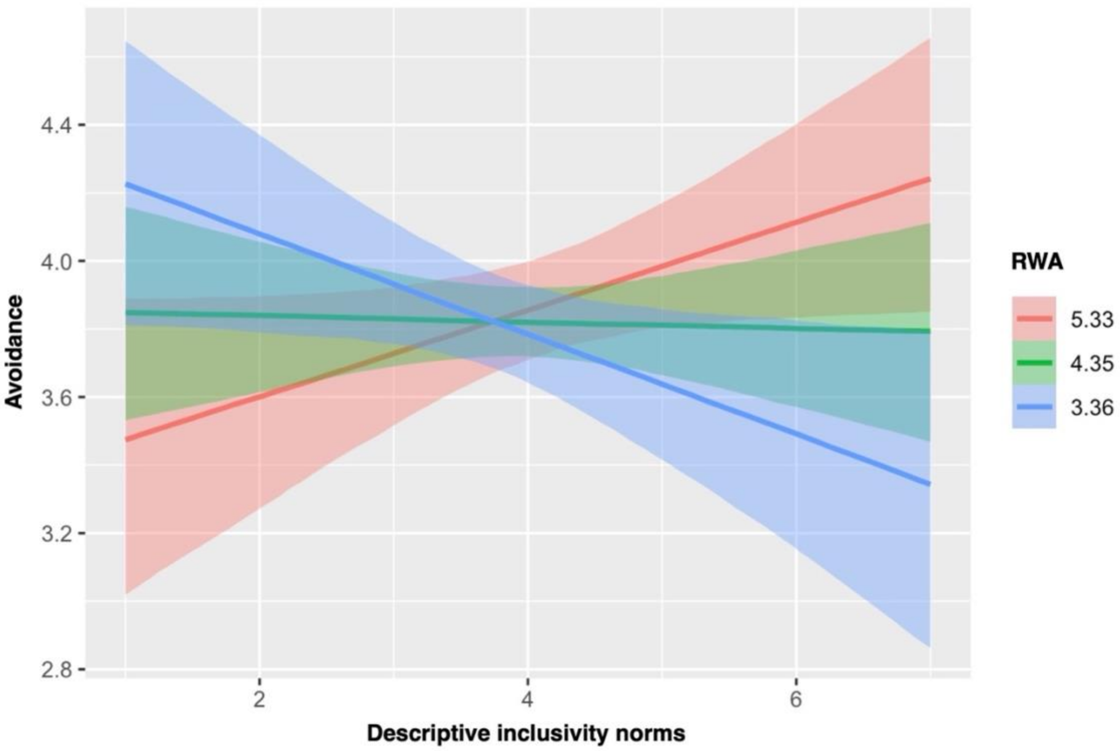

Fig. S23. Bayesian multiple regression slopes for the association between descriptive inclusivity norms and avoidance tendencies at the mean for right-wing authoritarianism (RWA), one standard deviation above the mean for RWA, and one standard deviation below the mean for RWA (Czech sample).

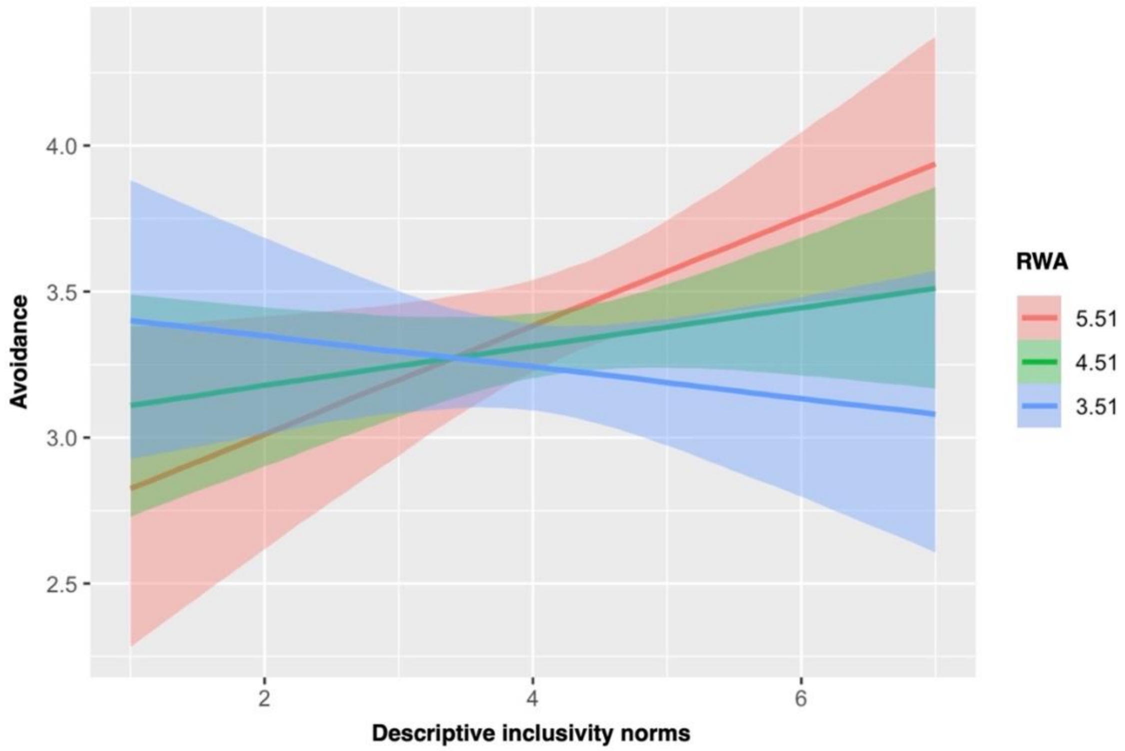

Fig. S24. Bayesian multiple regression slopes for the association between descriptive inclusivity norms and avoidance tendencies at the mean for right-wing authoritarianism (RWA), one standard deviation above the mean for RWA, and one standard deviation below the mean for RWA (Dutch sample).

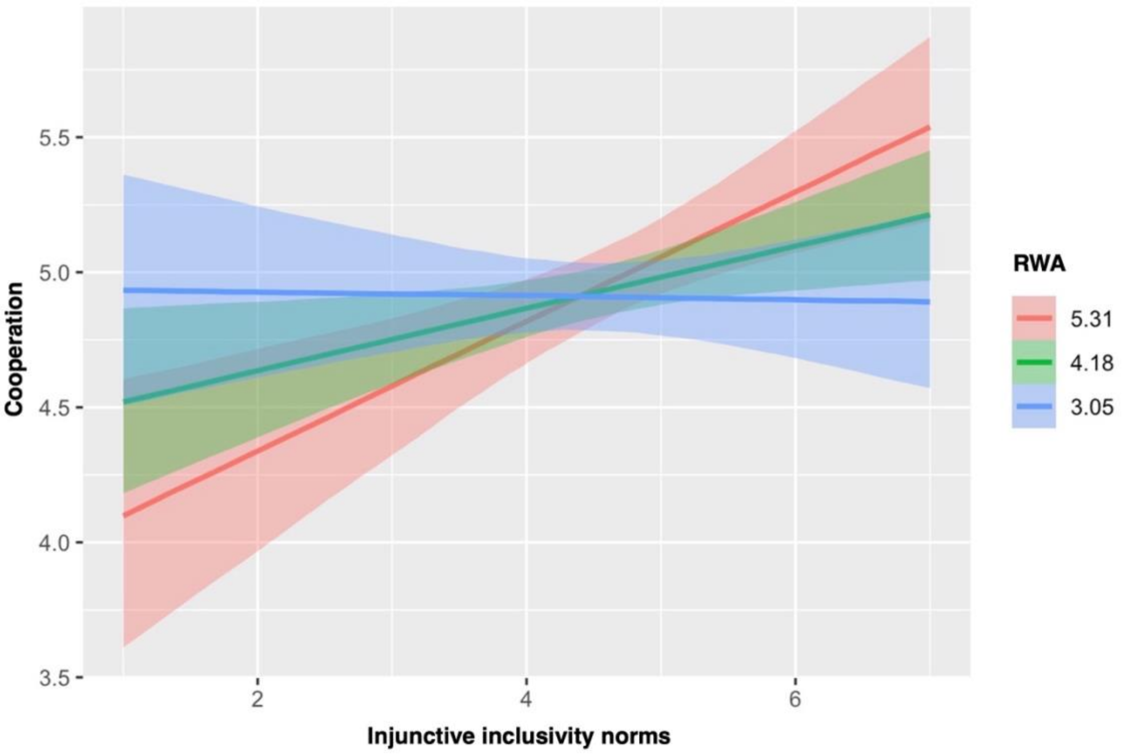

Fig. S25. Bayesian multiple regression slopes for the association between injunctive inclusivity norms and cooperation willingness at the mean for right-wing authoritarianism (RWA), one standard deviation above the mean for RWA, and one standard deviation below the mean for RWA (Greek sample).

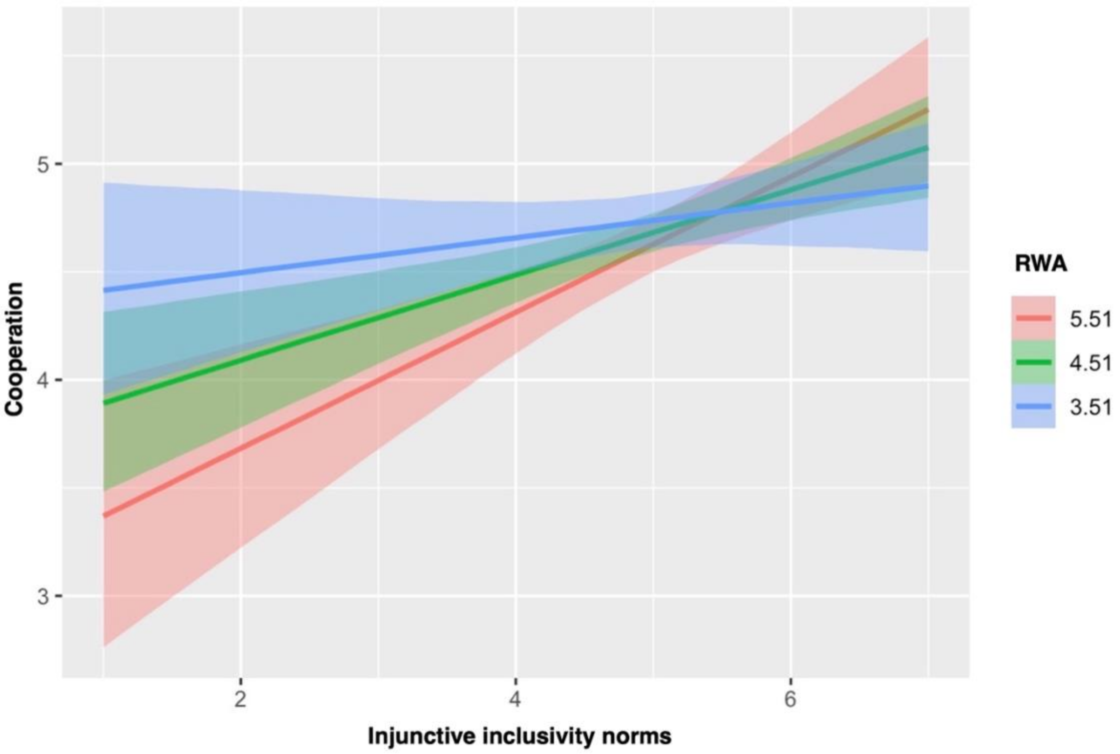

Fig. S26. Bayesian multiple regression slopes for the association between injunctive inclusivity norms and cooperation willingness at the mean for right-wing authoritarianism (RWA), one standard deviation above the mean for RWA, and one standard deviation below the mean for RWA (Dutch sample).

Tables

Sample Description

Table S1.
Sample Characteristics by Country

Variable	**GER** N = 1,009	**CZ** N = 1,002	**GR** N = 1,009	**UK** N = 1,002	**ES** N = 999	**FI** N = 1,001	**FR** N = 1,005	**HU** N = 1,005	**IT** N = 1,003	**NL** N = 1,005	**PL** N = 1,003	**SV** N = 998
**Age** (years)[1]	44.6 (15.1)	43.3 (14.9)	42.2 (13.7)	42.9 (15.1)	43.9 (14.5)	43.9 (15.1)	43.6 (15.4)	43.5 (14.8)	44.7 (14.9)	43.6 (15.6)	42.7 (14.9)	42.4 (15.1)
Gender[2]												
Female	502 (50%)	492 (49%)	496 (49%)	507 (51%)	502 (50%)	494 (49%)	515 (51%)	522 (52%)	505 (50%)	524 (52%)	502 (50%)	500 (50%)
Male	504 (50%)	509 (51%)	505 (50%)	494 (49%)	495 (50%)	507 (51%)	487 (48%)	481 (48%)	496 (49%)	479 (48%)	500 (50%)	495 (50%)
Other	3 (0.3%)	0 (0%)	2 (0.2%)	1 (<0.1%)	2 (0.2%)	0 (0%)	3 (0.3%)	2 (0.2%)	1 (<0.1%)	1 (<0.1%)	0 (0%)	3 (0.3%)
Non-binary	0 (0%)	0 (0%)	0 (0%)	0 (0%)	0 (0%)	0 (0%)	0 (0%)	0 (0%)	0 (0%)	0 (0%)	0 (0%)	0 (0%)
Another gender identity or multiple identities	0 (0%)	0 (0%)	0 (0%)	0 (0%)	0 (0%)	0 (0%)	0 (0%)	0 (0%)	0 (0%)	0 (0%)	0 (0%)	0 (0%)
Do not wish to say	0 (0%)	1 (<0.1%)	6 (0.6%)	0 (0%)	0 (0%)	0 (0%)	0 (0%)	0 (0%)	1 (<0.1%)	1 (<0.1%)	1 (<0.1%)	0 (0%)
**Education** (ISCED Classification)[3]												
< ISCED level 1	0 (0%)	3 (0.3%)	4 (0.4%)	0 (0%)	1 (0.1%)	28 (2.8%)	2 (0.2%)	1 (<0.1%)	4 (0.4%)	4 (0.4%)	1 (<0.1%)	13 (1.3%)
ISCED level 1	0 (0%)	3 (0.3%)	8 (0.8%)	0 (0%)	23 (2.3%)	0 (0%)	75 (7.5%)	3 (0.3%)	27 (2.7%)	20 (2.0%)	36 (3.6%)	10 (1.0%)
ISCED level 2	0 (0%)	119 (12%)	47 (4.7%)	0 (0%)	172 (17%)	159 (16%)	131 (13%)	137 (14%)	347 (35%)	179 (18%)	17 (1.7%)	169 (17%)
ISCED level 3	0 (0%)	485 (48%)	459 (45%)	0 (0%)	443 (44%)	367 (37%)	408 (41%)	502 (50%)	445 (44%)	407 (40%)	572 (57%)	325 (33%)
ISCED level 4	0 (0%)	157 (16%)	97 (9.6%)	0 (0%)	0 (0%)	81 (8.1%)	9 (0.9%)	129 (13%)	0 (0%)	14 (1.4%)	91 (9.1%)	69 (6.9%)
ISCED level 5	0 (0%)	229 (23%)	378 (37%)	0 (0%)	348 (35%)	349 (35%)	369 (37%)	226 (22%)	174 (17%)	374 (37%)	282 (28%)	403 (40%)
ISCED level 6	0 (0%)	6 (0.6%)	16 (1.6%)	14 (1.4%)	10 (1.0%)	17 (1.7%)	11 (1.1%)	7 (0.7%)	6 (0.6%)	7 (0.7%)	4 (0.4%)	9 (0.9%)

Variable	**GER** N = 1,009	**CZ** N = 1,002	**GR** N = 1,009	**UK** N = 1,002	**ES** N = 999	**FI** N = 1,001	**FR** N = 1,005	**HU** N = 1,005	**IT** N = 1,003	**NL** N = 1,005	**PL** N = 1,003	**SV** N = 998
Other	1,009 (100%)	0 (0%)	0 (0%)	988 (99%)	2 (0.2%)	0 (0%)	0 (0%)	0 (0%)	0 (0%)	0 (0%)	0 (0%)	0 (0%)
Religion[2]												
None	0 (0%)	0 (0%)	0 (0%)	0 (0%)	0 (0%)	0 (0%)	0 (0%)	0 (0%)	0 (0%)	0 (0%)	0 (0%)	0 (0%)
Christianity	629 (62%)	905 (90%)	407 (40%)	803 (80%)	700 (70%)	634 (63%)	724 (72%)	800 (80%)	539 (54%)	772 (77%)	522 (52%)	869 (87%)
Islam	330 (33%)	92 (9.2%)	598 (59%)	181 (18%)	292 (29%)	356 (36%)	224 (22%)	200 (20%)	455 (45%)	201 (20%)	478 (48%)	112 (11%)
Judaism	44 (4.4%)	0 (0%)	3 (0.3%)	12 (1.2%)	7 (0.7%)	8 (0.8%)	49 (4.9%)	0 (0%)	4 (0.4%)	28 (2.8%)	0 (0%)	11 (1.1%)
Hinduism	0 (0%)	2 (0.2%)	0 (0%)	3 (0.3%)	0 (0%)	0 (0%)	4 (0.4%)	2 (0.2%)	0 (0%)	0 (0%)	2 (0.2%)	1 (0.1%)
Buddhism	1 (<0.1%)	0 (0%)	0 (0%)	3 (0.3%)	0 (0%)	0 (0%)	1 (<0.1%)	0 (0%)	0 (0%)	1 (<0.1%)	1 (<0.1%)	1 (0.1%)
Other	5 (0.5%)	3 (0.3%)	1 (<0.1%)	0 (0%)	0 (0%)	3 (0.3%)	3 (0.3%)	3 (0.3%)	5 (0.5%)	3 (0.3%)	0 (0%)	4 (0.4%)
**Political Orientation** (1 = left, 10 = right)[1]	5.9 (1.9)	6.1 (2.2)	6.1 (2.3)	5.8 (2.1)	5.6 (2.7)	6.0 (2.4)	6.4 (2.6)	6.2 (2.4)	6.1 (2.7)	6.4 (2.3)	6.0 (2.7)	6.2 (2.6)

Note. [1]Mean (SD); [2]n (%); [3]n (%); Education levels in Germany and the UK were accessed according to national standards. Refer to Table S1A and Table S1B for detailed education levels in Germany and the UK.

Table S1A.

Education levels in Germany

Education levels (Germany)	N = 1,009
Grundschule oder darunter, z.B. kein Schulbesuch	65 (6.5%)
Volks-/Hauptschule, Realschule, Polytechnische Oberschule, integrierte Gesamtschule, Abendschule, Gymnasium (bis zehnte Klasse) oder vergleichbar	201 (20%)
Abgeschlossene Berufsausbildung	341 (34%)
Allgemeinbildender Sekundarbereich II (Abitur, Fachabitur, Abschluss erweiterte Oberschule usw.)	56 (5.6%)
Zweitausbildung, Abendschule (Sekundarbereich II) zusätzlich zu Ausbildung oder vergleichbar	102 (10%)
Abschluss Fachakademie/Berufsakademie, Meisterbrief oder vergleichbare Qualifikation	82 (8.2%)
Bachelor	145 (14%)
Magister Artium, Diplom, Staatsexamen, vergleichbarer Hochschulabschluss, Master	12 (1.2%)
Promotion, Habilitation	0 (0%)
Missing	5

Table S1B.

Education levels in the UK

Education levels (UK)	N = 1,002
Primary school or less	79 (15%)
GCSEs, O Levels, CSE, School Certificate, Scottish Ordinary, Lower Certificate, 3/Foundations S Grade, Scottish Access 1-2, Scottish intermediate 2/Credit S Grade, Foundation GNVQ, Intermediate GNVQ, BTEC first certificate, SVQ/NVQ level 1-2, Level 1-2 vocational awards, IVQ certificate/Technician/Diploma, Level 1-2 International Introductory Awards	270 (51%)
Vocational A-Levels, AVCE, BTEC National, Certificate/Diploma, City and Guilds SVQ/NVQ level 3, Level 3 vocational awards, IVQ Technician Diploma, IVQ Advanced Diploma, Level 3 International Awards	25 (4.8%)
A-levels or Higher Certificate, Scottish Higher Certificate, International Baccalaureate	152 (29%)
Nursing certificate, Teacher training, HE Diploma, Edexcel/B, Full technical certificate, BTEC HND/HNC, City and Guilds Licentiateship (LCGI), Graduateship (GCGI), Associateship (ACGI), Membership (MCGI), Master Professional, Diploma, Fellowship (FCGI), Higher Professional Diploma, SVQ/NVQ level 4-5, Level 4-5 vocational awards, IVQ Advanced Technician Diploma	0 (0%)
5 year University, CNAA first Degree (MB, BDS, BV etc.)	0 (0%)
Masters Degree, M.Phil, PGCE, PGDip, PGDE, PGCert, Post-Graduate Diplomas and Certificates	0 (0%)
Ph.D, D.Phil or equivalent	0 (0%)
Missing	476

Constructs

Table S2.

Overview of Constructs, Measures, and Items

Constructs & Measures	Items	Reference/Comment
Inclusivity Norms		
Descriptive inclusivity norms	Most people in [country] …	
Injunctive inclusivity norms	Most people in [country] believe that we should …	
1. Equality-based respect	1.1. … always treat everyone as a human being of equal worth.<br>1.2. … generally treat everyone equally.<br>1.3. … recognize everyone as a fellow citizen of equal worth.<br>(1 = Does not apply at all to 7 = Applies completely)	Adapted from Renger et al. (2017)<br>Adapted from Renger et al. (2017)<br>Adapted from Simon & Schaefer (2018)
2. Dialogue	2.1. … really listen to everyone to better understand differences.<br>2.2. … allow everyone to contribute so that something new and valuable can develop.<br>2.3. … consider everyone's arguments to learn from each other.<br>(1 = Does not apply at all to 7 = Applies completely)	Adapted from Verkuyten et al. (2020)<br>Adapted from Verkuyten et al. (2020)<br>Theoretically derived from Nagda (2006)
3. Unity	3.1. … really listen to everyone to better understand differences.<br>3.2. … allow everyone to contribute so that something new and valuable can develop.<br>3.3. … consider everyone's arguments to learn from each other.<br>(1 = Does not apply at all to 7 = Applies completely)	Adapted from Verkuyten et al. (2020)<br>Adapted from Verkuyten et al. (2020)<br>Adapted from Gaertner et al. (1994)
Strength of disapproval	Some people may have a different opinion on this topic. How strong is your disapproval regarding the opinion that [opposite opinion]?<br>(1 = Very weak to 7 = Very strong)	Adapted from Simon & Schaefer (2018)
Issue Importance	How important is the issue of [issue] to you personally?<br>(1 = Not at all important to 7 = Very important)	Adapted from Skitka et al. (2013)
Opinion Extremity	How strongly do you hold this opinion?<br>(1 = Very weakly to 7 = Very strongly)	Adapted from Skitka et al. (2013)
Identification with Society	I identify with [country] society.<br>(1 = Does not apply at all to 7 = Applies completely)	Adapted from Postmes et al. (2013)

Identification with the Opinion-Based Group	1. I identify with people who [opinion].<br>2. I feel strongly connected to others who [opinion].<br>(1 = Strongly disagree to 7 = Strongly agree)	All items adapted from Eisner et al. (2022)
Tolerance	1. These people should be able to stick to their opinion, even if others try to persuade them otherwise.<br>2. These people should be allowed to speak their mind freely and openly.<br>3. These people shall be allowed to meet in public spaces and give speeches.<br>4. These people shall be given the chance to pursue their interests just like others.<br>(1 = Strongly disagree to 7 = Strongly agree)	All items adapted from Simon et al. (2019)
Avoidance Tendencies	1. I would rather have nothing to do with these people if I had a choice.<br>2. I avoid contact with these people if I can.<br>(1 = Strongly disagree to 7 = Strongly agree)	All items adapted from Mackie et al. (2000); Tam et al. (2009)
Cooperation Willingness	1. I would cooperate with these people to solve problems that affect all members of society.<br>2. I am willing to meet with these people to discuss the concerns that affect all people in [country].<br>(1 = Strongly disagree to 7 = Strongly agree)	Adapted from Gómez et al. (2013)<br>Adapted from DiBella et al. (2017)
Perceived Social Cohesion	1. Social cohesion in [country] is at risk.<br>2. In reality, most people do not care at all about what is happening to their fellow human beings.<br>(1 = Strongly disagree to 7 = Strongly agree)	Adapted from Brand et al. (2020)<br>Adapted from Brand et al. (2020)
Social Trust	How much do you trust people in [country] in general?<br>(1 = Do not trust at all to 7 = Trust completely)	Adapted from OECD (2017)

Note. The respective country was adapted to participants' residential country. Participants' opinion, issue, and opposed opinion were adapted to the issue chosen and opinion indicated by them. Apart from these placeholders, all measures and items were identical across participants from the twelve countries.

Descriptives and Correlations across Countries

Table S3.
Weighted means, standard deviations, and Pearson correlation coefficients (country-level)

Variable	*M*	*SD*	1	2	3	4
1. Descriptive inclusivity norms	4.09	0.17				
2. Injunctive inclusivity norms	4.79	0.24	.75			
3. Social trust	3.80	0.25	.50	.31		
4. Social cohesion	2.88	0.29	.68	.49	.68	
5. Polarization index	29.45	2.32	-.32	-.10	-.44	-.59

Note. M and *SD* represent mean and standard deviation, respectively.

Descriptives and correlations by country

Table S4.

Means, standard deviations, and Pearson correlations coefficients (Czech sample)

Variable	*M*	*SD*	1	2	3	4	5	6	7	8	9	10	11	12
1. Descriptive inclusivity norms	3.98	1.32												
2. Injunctive inclusivity norms	4.77	1.28	.62**											
3. Tolerance	4.90	1.34	.17**	.16**										
4. Avoidance tendencies	3.85	1.71	-.02	-.02	-.49**									
5. Cooperation willingness	4.68	1.53	.19**	.19**	.63**	-.50**								
6. Strength of disapproval	4.69	1.90	-.01	.00	-.26**	.34**	-.15**							
7. Issue importance	5.31	1.82	.05	.10**	.03	.04	.10**	.20**						
8. Opinion extremity	5.85	1.33	-.01	.03	-.08*	.15**	-.02	.45**	.36**					
9. Identification difference score	0.72	1.96	-.13**	-.11**	-.18**	.17**	-.11**	.31**	.25**	.38**				
10. Identification with society	4.72	1.54	.30**	.30**	.21**	-.10**	.19**	.00	.06*	.00	-.70**			
11. Identification with opinion-based group	5.44	1.41	.14**	.18**	-.02	.13**	.06	.43**	.42**	.52**	.62**	.12**		
12. SDO	3.80	1.33	.10**	.05	-.02	.13**	-.04	.04	-.04	.03	-.07*	.09**	.00	
13. RWA	4.35	0.98	.22**	.20**	.12**	.01	.11**	.02	.07*	.03	-.18**	.31**	.09**	.33**

Note. M and *SD* are used to represent mean and standard deviation, respectively. The identification difference score was calculated by subtracting identification with society from identification with the opinion-based group, resulting in a scale from -6 to 6, with high values suggesting a stronger identification with the opinion-based group relative to society. *indicates $p < .05$. ** indicates $p < .01$.

Table S5.

Means, standard deviations, and Pearson correlations coefficients (Finnish sample)

Variable	*M*	*SD*	1	2	3	4	5	6	7	8	9	10	11	12
1. Descriptive inclusivity norms	4.06	1.32												
2. Injunctive inclusivity norms	4.92	1.28	.56**											
3. Tolerance	4.69	1.34	.17**	.16**										
4. Avoidance tendencies	3.53	1.71	-.11**	-.15**	-.54**									
5. Cooperation willingness	4.74	1.53	.19**	.20**	.58**	-.50**								
6. Strength of disapproval	4.69	1.90	-.10**	-.08*	-.44**	.42**	-.19**							
7. Issue importance	5.04	1.82	.01	.05	-.13**	.11**	.12**	.38**						
8. Opinion extremity	6.18	1.33	-.08*	-.03	-.23**	.20**	-.04	.48**	.35**					
9. Identification difference score	0.01	1.96	-.19**	-.15**	-.31**	.28**	-.12**	.44**	.33**	.39**				
10. Identification with society	5.17	1.54	.33**	.30**	.19**	-.16**	.19**	-.09**	.02	.01	-.65**			
11. Identification with opinion-based group	5.18	1.41	.05	.07*	-.23**	.23**	.01	.49**	.46**	.52**	.72**	.06*		
12. SDO	3.18	1.42	.16**	.04	-.04	.07*	-.11**	-.03	-.14**	-.11**	-.08**	.08**	-.03	
13. RWA	4.20	1.07	.28**	.21**	.06	-.01	-.05	-.05	-.08*	-.05	-.19**	.35**	.07*	.43**

Note. M and *SD* are used to represent mean and standard deviation, respectively. The identification difference score was calculated by subtracting identification with society from identification with the opinion-based group, resulting in a scale from -6 to 6, with high values suggesting a stronger identification with the opinion-based group relative to society. *indicates $p < .05$. ** indicates $p < .01$.

Table S6.

Means, standard deviations, and Pearson correlations coefficients (French sample)

Variable	*M*	*SD*	1	2	3	4	5	6	7	8	9	10	11	12
1. Descriptive inclusivity norms	3.97	1.46												
2. Injunctive inclusivity norms	4.76	1.37	.57**											
3. Tolerance	4.88	1.25	.13**	.19**										
4. Avoidance tendencies	3.82	1.74	.06	.02	-.38**									
5. Cooperation willingness	4.85	1.46	.15**	.20**	.51**	-.32**								
6. Strength of disapproval	5.08	1.81	-.03	-.01	-.18**	.22**	-.05							
7. Issue importance	5.79	1.48	.07*	.06*	.03	.08*	.14**	.20**						
8. Opinion extremity	5.81	1.27	.09**	.09**	-.01	.15**	.10**	.32**	.46**					
9. Identification difference score	0.40	2.19	-.15**	-.11**	-.14**	.22**	.01	.21**	.31**	.28**				
10. Identification with society	4.96	1.61	.28**	.27**	.14**	-.11**	.12**	.00	.01	.05	-.69**			
11. Identification with opinion-based group	5.36	1.59	.07*	.12**	-.06	.18**	.13**	.29**	.44**	.44**	.68**	.06		
12. SDO	3.76	1.46	.15**	.04	.03	.17**	-.09**	-.05	-.09**	-.11**	-.10**	.07*	-.07*	
13. RWA	4.46	1.09	.25**	.18**	.13**	.04	.06	.03	.01	.05	-.21**	.35**	.06*	.34**

Note. M and *SD* are used to represent mean and standard deviation, respectively. The identification difference score was calculated by subtracting identification with society from identification with the opinion-based group, resulting in a scale from -6 to 6, with high values suggesting a stronger identification with the opinion-based group relative to society. *indicates $p < .05$. ** indicates $p < .01$.

Table S7.
Means, standard deviations, and Pearson correlations coefficients (German sample)

Variable	*M*	*SD*	1	2	3	4	5	6	7	8	9	10	11	12
1. Descriptive inclusivity norms	4.23	1.37												
2. Injunctive inclusivity norms	4.81	1.23	.62**											
3. Tolerance	5.04	1.25	.19**	.25**										
4. Avoidance tendencies	3.92	1.75	.07*	-.02	-.34**									
5. Cooperation willingness	4.98	1.44	.17**	.26**	.61**	-.36**								
6. Strength of disapproval	4.96	1.58	.02	-.02	-.16**	.35**	-.07*							
7. Issue importance	5.52	1.62	.08*	.13**	.07*	.06	.22**	.21**						
8. Opinion extremity	6.00	1.16	-.00	.04	-.01	.14**	.04	.39**	.40**					
9. Identification difference score	0.34	1.87	-.11**	-.11**	-.13**	.21**	-.07*	.25**	.20**	.29**				
10. Identification with society	5.05	1.50	.29**	.31**	.18**	-.07*	.22**	.05	.13**	.13**	-.67**			
11. Identification with opinion-based group	5.39	1.41	.16**	.19**	.02	.20**	.14**	.38**	.40**	.52**	.62**	.17**		
12. SDO	3.95	1.42	.25**	.16**	-.03	.20**	-.05	.08*	-.06	-.03	-.05	.14**	.08**	
13. RWA	4.57	1.10	.26**	.29**	.08*	.07*	.10**	.06	.08**	.05	-.20**	.41**	.17**	.43**

Note. M and *SD* are used to represent mean and standard deviation, respectively. The identification difference score was calculated by subtracting identification with society from identification with the opinion-based group, resulting in a scale from -6 to 6, with high values suggesting a stronger identification with the opinion-based group relative to society. *indicates $p < .05$. ** indicates $p < .01$.

Table S8.

Means, standard deviations, and Pearson correlations coefficients (Greek sample)

Variable	*M*	*SD*	1	2	3	4	5	6	7	8	9	10	11	12
1. Descriptive inclusivity norms	3.86	1.36												
2. Injunctive inclusivity norms	4.51	1.33	.67**											
3. Tolerance	4.89	1.32	.18**	.17**										
4. Avoidance tendencies	3.71	1.72	-.00	-.04	-.41**									
5. Cooperation willingness	4.94	1.48	.17**	.17**	.65**	-.47**								
6. Strength of disapproval	4.98	1.72	.07*	.00	-.17**	.30**	-.15**							
7. Issue importance	5.84	1.55	.03	.03	-.00	.07*	.08*	.28**						
8. Opinion extremity	6.02	1.13	.00	.04	-.08*	.14**	-.05	.39**	.38**					
9. Identification difference score	1.14	2.00	-.28**	-.22**	-.14**	.15**	-.09**	.17**	.20**	.27**				
10. Identification with society	4.34	1.72	.42**	.36**	.11**	-.02	.12**	.08**	.10**	.10**	-.74**			
11. Identification with opinion-based group	5.48	1.36	.11**	.14**	-.08*	.19**	.02	.35**	.43**	.52**	.53**	.17**		
12. SDO	3.32	1.47	.23**	.19**	-.06*	.17**	-.07*	.07*	-.06*	-.03	-.20**	.23**	.00	
13. RWA	4.18	1.13	.35**	.31**	.06	.07*	.08*	.07*	.05	.06	-.32**	.45**	.11**	.42**

Note. M and *SD* are used to represent mean and standard deviation, respectively. The identification difference score was calculated by subtracting identification with society from identification with the opinion-based group, resulting in a scale from -6 to 6, with high values suggesting a stronger identification with the opinion-based group relative to society. *indicates $p < .05$. ** indicates $p < .01$.

Table S9.

Means, standard deviations, and Pearson correlations coefficients (Hungarian sample)

Variable	*M*	*SD*	1	2	3	4	5	6	7	8	9	10	11	12
1. Descriptive inclusivity norms	3.71	1.50												
2. Injunctive inclusivity norms	4.14	1.41	.70**											
3. Tolerance	4.89	1.31	.07*	.10**										
4. Avoidance tendencies	3.62	1.78	.05	.01	-.40**									
5. Cooperation willingness	4.69	1.55	.10**	.08*	.63**	-.38**								
6. Strength of disapproval	4.88	1.96	.04	.04	-.17**	.26**	-.08*							
7. Issue importance	5.28	1.97	.05	.03	.02	.10**	.11**	.28**						
8. Opinion extremity	6.18	1.16	.01	.04	-.01	.12**	.00	.39**	.30**					
9. Identification difference score	1.12	2.08	-.31**	-.29**	-.04	.14**	-.02	.26**	.20**	.33**				
10. Identification with society	4.39	1.76	.46**	.44**	.04	-.03	.08*	.04	.05	.02	-.74**			
11. Identification with opinion-based group	5.50	1.43	.11**	.12**	-.01	.17**	.07*	.43**	.35**	.51**	.55**	.15**		
12. SDO	3.58	1.43	.25**	.19**	-.05	.13**	-.10**	.04	-.02	-.01	-.18**	.24**	.04	
13. RWA	4.27	1.21	.44**	.38**	-.00	.09**	.00	.04	.04	.02	-.40**	.59**	.14**	.39**

Note. M and *SD* are used to represent mean and standard deviation, respectively. The identification difference score was calculated by subtracting identification with society from identification with the opinion-based group, resulting in a scale from -6 to 6, with high values suggesting a stronger identification with the opinion-based group relative to society. *indicates $p < .05$. ** indicates $p < .01$.

Table S10.

Means, standard deviations, and Pearson correlations coefficients (Italian sample)

Variable	*M*	*SD*	1	2	3	4	5	6	7	8	9	10	11	12
1. Descriptive inclusivity norms	3.98	1.49												
2. Injunctive inclusivity norms	4.94	1.40	.60**											
3. Tolerance	5.02	1.27	.19**	.20**										
4. Avoidance tendencies	3.66	1.80	.01	-.07*	-.42**									
5. Cooperation willingness	5.05	1.44	.19**	.23**	.59**	-.42**								
6. Strength of disapproval	5.07	1.73	.01	-.03	-.17**	.27**	-.08**							
7. Issue importance	5.85	1.50	.07*	.09**	.00	.07*	.17**	.18**						
8. Opinion extremity	5.94	1.26	-.03	.04	-.08*	.13**	.03	.33**	.35**					
9. Identification difference score	1.32	2.06	-.39**	-.26**	-.17**	.17**	-.14**	.21**	.14**	.31**				
10. Identification with society	4.42	1.73	.49**	.37**	.16**	-.07*	.20**	.01	.10**	.01	-.79**			
11. Identification with opinion-based group	5.74	1.25	.03	.07*	-.06	.18**	.05	.36**	.36**	.52**	.55**	.07*		
12. SDO	3.75	1.46	.19**	.11**	.06*	.10**	.01	-.01	-.05	-.09**	-.22**	.18**	-.11**	
13. RWA	4.62	1.12	.37**	.32**	.16**	-.02	.15**	.04	.04	.01	-.36**	.48**	.07*	.34**

Note. M and *SD* are used to represent mean and standard deviation, respectively. The identification difference score was calculated by subtracting identification with society from identification with the opinion-based group, resulting in a scale from -6 to 6, with high values suggesting a stronger identification with the opinion-based group relative to society. *indicates $p < .05$. ** indicates $p < .01$.

Table S11.
Means, standard deviations, and Pearson correlations coefficients (Dutch sample)

Variable	*M*	*SD*	1	2	3	4	5	6	7	8	9	10	11	12
1. Descriptive inclusivity norms	4.18	1.20												
2. Injunctive inclusivity norms	4.91	1.06	.61**											
3. Tolerance	5.09	1.18	.13**	.15**										
4. Avoidance tendencies	3.34	1.69	-.01	-.07*	-.49**									
5. Cooperation willingness	4.69	1.44	.15**	.18**	.55**	-.43**								
6. Strength of disapproval	4.85	1.93	.08*	.03	.04	.06	.07*							
7. Issue importance	4.53	2.17	.07*	.08**	.13**	-.08*	.28**	-.04						
8. Opinion extremity	6.23	0.97	-.06	-.03	-.02	.12**	.03	.20**	.06					
9. Identification difference score	-0.08	2.02	-.12**	-.15**	-.16**	.22**	-.05	.05	.10**	.34**				
10. Identification with society	5.19	1.37	.26**	.29**	.18**	-.17**	.17**	.07*	.10**	-.04	-.66**			
11. Identification with opinion-based group	5.11	1.51	.08*	.07*	-.05	.13**	.08**	.13**	.23**	.41**	.73**	.02		
12. SDO	3.89	1.30	.18**	.06*	-.05	.11**	-.10**	.08*	-.06*	-.03	-.06	.08*	-.01	
13. RWA	4.51	1.00	.30**	.23**	-.01	.04	.01	.06	-.00	-.06	-.26**	.39**	.01	.39**

Note. M and *SD* are used to represent mean and standard deviation, respectively. The identification difference score was calculated by subtracting identification with society from identification with the opinion-based group, resulting in a scale from -6 to 6, with high values suggesting a stronger identification with the opinion-based group relative to society. *indicates $p < .05$. ** indicates $p < .01$.

Table S12.

Means, standard deviations, and Pearson correlations coefficients (Polish sample)

Variable	*M*	*SD*	1	2	3	4	5	6	7	8	9	10	11	12
1. Descriptive inclusivity norms	4.36	1.39												
2. Injunctive inclusivity norms	5.08	1.30	.64**											
3. Tolerance	5.01	1.28	.13**	.16**										
4. Avoidance tendencies	3.57	1.71	.04	-.04	-.48**									
5. Cooperation willingness	4.98	1.39	.18**	.23**	.61**	-.38**								
6. Strength of disapproval	5.32	1.70	-.05	-.06	-.10**	.13**	-.01							
7. Issue importance	5.55	1.79	.07*	.08**	-.06	.08*	.10**	.14**						
8. Opinion extremity	6.12	1.13	-.03	.03	-.05	.10**	.05	.18**	.34**					
9. Identification difference score	0.29	1.83	-.21**	-.18**	-.12**	.16**	-.06	.15**	.22**	.34**				
10. Identification with society	5.33	1.51	.37**	.32**	.11**	-.07*	.17**	.04	.14**	.07*	-.67**			
11. Identification with opinion-based group	5.62	1.39	.13**	.11**	-.04	.13**	.11**	.23**	.44**	.52**	.59**	.21**		
12. SDO	3.85	1.35	.16**	.12**	-.03	.18**	-.11**	-.01	-.05	-.06*	-.10**	.09**	-.04	
13. RWA	4.38	1.17	.34**	.30**	.03	.06	.06*	-.03	.05	.05	-.26**	.42**	.12**	.33**

Note. M and *SD* are used to represent mean and standard deviation, respectively. The identification difference score was calculated by subtracting identification with society from identification with the opinion-based group, resulting in a scale from -6 to 6, with high values suggesting a stronger identification with the opinion-based group relative to society. *indicates *p* < .05. ** indicates *p* < .01.

Table S13.

Means, standard deviations, and Pearson correlations coefficients (Spanish sample)

Variable	*M*	*SD*	1	2	3	4	5	6	7	8	9	10	11	12
1. Descriptive inclusivity norms	4.09	1.41												
2. Injunctive inclusivity norms	4.76	1.38	.67**											
3. Tolerance	5.09	1.39	.15**	.20**										
4. Avoidance tendencies	3.60	1.76	.06	-.03	-.45**									
5. Cooperation willingness	5.02	1.44	.18**	.19**	.58**	-.37**								
6. Strength of disapproval	4.90	1.84	.03	-.01	-.21**	.26**	-.07*							
7. Issue importance	5.81	1.61	.09**	.11**	-.04	.10**	.17**	.19**						
8. Opinion extremity	6.10	1.12	.01	.01	-.10**	.10**	.07*	.29**	.36**					
9. Identification difference score	1.01	2.03	-.20**	-.21**	-.22**	.20**	-.09**	.24**	.32**	.36**				
10. Identification with society	4.64	1.67	.37**	.35**	.20**	-.09**	.20**	-.05	.04	.01	-.75**			
11. Identification with opinion-based group	5.65	1.36	.15**	.12**	-.08**	.18**	.11**	.30**	.53**	.54**	.58**	.11**		
12. SDO	3.51	1.51	.21**	.12**	.07*	.08*	-.02	-.01	-.12**	-.09**	-.15**	.15**	-.05	
13. RWA	4.23	1.20	.36**	.31**	.18**	.00	.14**	-.01	-.02	-.03	-.30**	.44**	.09**	.42**

Note. M and *SD* are used to represent mean and standard deviation, respectively. The identification difference score was calculated by subtracting identification with society from identification with the opinion-based group, resulting in a scale from -6 to 6, with high values suggesting a stronger identification with the opinion-based group relative to society. *indicates $p < .05$. ** indicates $p < .01$.

Table S14.

Means, standard deviations, and Pearson correlations coefficients (Swedish sample)

Variable	*M*	*SD*	1	2	3	4	5	6	7	8	9	10	11	12
1. Descriptive inclusivity norms	4.14	1.39												
2. Injunctive inclusivity norms	4.95	1.22	.58**											
3. Tolerance	5.11	1.32	.13**	.18**										
4. Avoidance tendencies	3.83	1.63	-.06	-.11**	-.37**									
5. Cooperation willingness	4.90	1.41	.15**	.19**	.53**	-.35**								
6. Strength of disapproval	5.05	1.75	.02	-.06	-.10**	.26**	-.07*							
7. Issue importance	5.48	1.67	.11**	.04	-.07*	.12**	.09**	.30**						
8. Opinion extremity	5.96	1.25	-.02	.01	-.01	.12**	.08*	.33**	.51**					
9. Identification difference score	-0.11	1.95	-.11**	-.06	-.17**	.19**	-.07*	.23**	.25**	.27**				
10. Identification with society	5.41	1.52	.26**	.19**	.13**	-.07*	.11**	-.01	.11**	.09**	-.69**			
11. Identification with opinion-based group	5.31	1.41	.13**	.13**	-.09**	.19**	.02	.31**	.46**	.47**	.63**	.12**		
12. SDO	3.77	1.40	.07*	-.06	-.06*	.10**	-.05	-.04	-.04	-.08*	-.06*	.05	-.03	
13. RWA	4.68	1.11	.20**	.15**	.01	.00	.02	-.07*	.05	.00	-.28**	.39**	.03	.32**

Note. M and *SD* are used to represent mean and standard deviation, respectively. The identification difference score was calculated by subtracting identification with society from identification with the opinion-based group, resulting in a scale from -6 to 6, with high values suggesting a stronger identification with the opinion-based group relative to society. *indicates $p < .05$. ** indicates $p < .01$.

Table S15.

Means, standard deviations, and Pearson correlations coefficients (British sample)

Variable	*M*	*SD*	1	2	3	4	5	6	7	8	9	10	11	12
1. Descriptive inclusivity norms	4.27	1.39												
2. Injunctive inclusivity norms	4.75	1.30	.70**											
3. Tolerance	5.08	1.22	.13**	.12**										
4. Avoidance tendencies	3.65	1.70	-.08**	-.08*	-.45**									
5. Cooperation willingness	5.02	1.37	.20**	.19**	.58**	-.44**								
6. Strength of disapproval	5.13	1.63	-.01	-.04	-.14**	.28**	-.03							
7. Issue importance	5.51	1.64	.04	.05	-.07*	.14**	.11**	.33**						
8. Opinion extremity	6.08	1.11	-.02	.01	-.09**	.19**	.03	.45**	.49**					
9. Identification difference score	0.32	2.07	-.14**	-.14**	-.17**	.24**	-.06*	.27**	.34**	.30**				
10. Identification with society	5.05	1.55	.29**	.27**	.13**	-.15**	.17**	-.01	.05	.03	-.73**			
11. Identification with opinion-based group	5.37	1.42	.11**	.09**	-.11**	.18**	.09**	.38**	.56**	.46**	.67**	.03		
12. SDO	3.58	1.44	.16**	.13**	-.04	.10**	-.11**	.01	-.09**	-.04	-.19**	.18**	-.08*	
13. RWA	4.21	1.19	.26**	.28**	-.03	.07*	-.01	.04	.04	.02	-.26**	.43**	.08**	.51**

Note. M and *SD* are used to represent mean and standard deviation, respectively. The identification difference score was calculated by subtracting identification with society from identification with the opinion-based group, resulting in a scale from -6 to 6, with high values suggesting a stronger identification with the opinion-based group relative to society. *indicates $p < .05$. ** indicates $p < .01$.

Individual-level analyses: Main analyses by country

Table S16.
Hierarchical Bayesian regression results (Czech sample)

	Tolerance			
Predictors	*Estimates*	*CI (95%)*	*Estimates*	*CI (95%)*
Injunctive inclusivity norms	0.08	0.00 – 0.16	-0.07	-0.23 – 0.09
Descriptive inclusivity norms	0.12	0.05 – 0.20	-0.11	-0.33 – 0.11
Topic importance	0.05	0.00 – 0.10	0.05	0.00 – 0.10
Opinion extremity	0.02	-0.05 – 0.09	0.02	-0.05 – 0.09
Strength of disapproval	-0.20	-0.24 – -0.15	-0.20	-0.24 – -0.15
Injunctive inclusivity norms x Descriptive inclusivity norms			0.04	0.00 – 0.09
Observations	1002		1002	
R^2 Bayes	0.108		0.113	
	**Avoidance tendencies**			
Predictors	*Estimates*	*CI (95%)*	*Estimates*	*CI (95%)*
Injunctive inclusivity norms	-0.03	-0.14 – 0.07	0.03	-0.17 – 0.23
Descriptive inclusivity norms	0.01	-0.09 – 0.10	0.11	-0.18 – 0.39
Topic importance	-0.03	-0.09 – 0.03	-0.03	-0.09 – 0.03
Opinion extremity	0.02	-0.07 – 0.11	0.02	-0.07 – 0.11
Strength of disapproval	0.31	0.25 – 0.37	0.31	0.25 – 0.37
Injunctive inclusivity norms x Descriptive inclusivity norms			-0.02	-0.07 – 0.03
Observations	1002		1002	
R^2 Bayes	0.120		0. 121	
	**Cooperation willingness**			
Predictors	*Estimates*	*CI (95%)*	*Estimates*	*CI (95%)*
Injunctive inclusivity norms	0.13	0.04 – 0.23	0.12	-0.07 – 0.30
Descriptive inclusivity norms	0.13	0.04 – 0.22	0.11	-0.15 – 0.37
Topic importance	0.10	0.04 – 0.15	0.10	0.04 – 0.15
Opinion extremity	0.01	-0.07 – 0.09	0.01	-0.07 – 0.09
Strength of disapproval	-0.14	-0.19 – -0.08	-0.14	-0.19 – -0.08
Injunctive inclusivity norms x Descriptive inclusivity norms			0.00	-0.04 – 0.05
Observations	1002		1002	
R^2 Bayes	0.081		0. 082	

Table S17.
Hierarchical Bayesian regression results (Finnish sample)

	Tolerance			
Predictors	*Estimates*	*CI (95%)*	*Estimates*	*CI (95%)*
Injunctive inclusivity norms	0.09	0.01 – 0.17	-0.15	-0.34 – 0.03
Descriptive inclusivity norms	0.10	0.02 – 0.18	-0.27	-0.53 – -0.00
Topic importance	0.03	-0.02 – 0.08	0.03	-0.02 – 0.07
Opinion extremity	-0.04	-0.13 – 0.05	-0.04	-0.13 – 0.04
Strength of disapproval	-0.34	-0.39 – -0.28	-0.34	-0.39 – -0.29
Injunctive inclusivity norms x Descriptive inclusivity norms			0.07	0.02 – 0.12
Observations	1001		1001	
R^2 Bayes	0.216		0.223	
	**Avoidance tendencies**			
Predictors	*Estimates*	*CI (95%)*	*Estimates*	*CI (95%)*
Injunctive inclusivity norms	-0.16	-0.26 – -0.06	-0.06	-0.30 – 0.17
Descriptive inclusivity norms	-0.01	-0.11 – 0.09	0.13	-0.20 – 0.45
Topic importance	-0.04	-0.10 – 0.01	-0.04	-0.10 – 0.02
Opinion extremity	0.02	-0.09 – 0.13	0.02	-0.08 – 0.13
Strength of disapproval	0.41	0.35 – 0.48	0.42	0.35 – 0.48
Injunctive inclusivity norms x Descriptive inclusivity norms			-0.03	-0.09 – 0.04
Observations	1001		1001	
R^2 Bayes	0.193		0. 195	
	**Cooperation willingness**			
Predictors	*Estimates*	*CI (95%)*	*Estimates*	*CI (95%)*
Injunctive inclusivity norms	0.16	0.06 – 0.25	-0.13	-0.34 – 0.08
Descriptive inclusivity norms	0.13	0.03 – 0.22	-0.30	-0.60 – -0.00
Topic importance	0.16	0.11 – 0.22	0.16	0.11 – 0.21
Opinion extremity	0.03	-0.07 – 0.12	0.03	-0.08 – 0.12
Strength of disapproval	-0.22	-0.28 – -0.16	-0.22	-0.28 – -0.16
Injunctive inclusivity norms x Descriptive inclusivity norms			0.08	0.03 – 0.14
Observations	1001		1001	
R^2 Bayes	0.114		0. 123	

Table S18.
Hierarchical Bayesian regression results (French sample)

	Tolerance			
Predictors	*Estimates*	*CI (95%)*	*Estimates*	*CI (95%)*
Injunctive inclusivity norms	0.16	0.10 – 0.23	0.02	-0.10 – 0.14
Descriptive inclusivity norms	0.02	-0.04 – 0.08	-0.23	-0.42 – -0.05
Topic importance	0.04	-0.02 – 0.10	0.04	-0.02 – 0.10
Opinion extremity	0.01	-0.06 – 0.08	0.01	-0.06 – 0.07
Strength of disapproval	-0.13	-0.18 – -0.09	-0.14	-0.18 – -0.09
Injunctive inclusivity norms x Descriptive inclusivity norms			0.05	0.01 – 0.08
Observations	1005		1005	
R^2 Bayes	0.067		0.083	

	Avoidance tendencies			
Predictors	*Estimates*	*CI (95%)*	*Estimates*	*CI (95%)*
Injunctive inclusivity norms	-0.04	-0.13 – 0.06	0.07	-0.10 – 0.24
Descriptive inclusivity norms	0.09	0.00 – 0.17	0.27	-0.00 – 0.53
Topic importance	-0.01	-0.09 – 0.07	-0.01	-0.09 – 0.08
Opinion extremity	0.12	0.02 – 0.21	0.12	0.02 – 0.22
Strength of disapproval	0.19	0.12 – 0.25	0.19	0.13 – 0.25
Injunctive inclusivity norms x Descriptive inclusivity norms			-0.03	-0.08 – 0.01
Observations	1005		1005	
R^2 Bayes	0.062		0.065	

	Cooperation willingness			
Predictors	*Estimates*	*CI (95%)*	*Estimates*	*CI (95%)*
Injunctive inclusivity norms	0.17	0.09 – 0.25	-0.02	-0.16 – 0.12
Descriptive inclusivity norms	0.04	-0.03 – 0.12	-0.28	-0.50 – -0.06
Topic importance	0.12	0.05 – 0.18	0.11	0.05 – 0.18
Opinion extremity	0.07	-0.02 – 0.15	0.06	-0.02 – 0.14
Strength of disapproval	-0.07	-0.13 – -0.02	-0.08	-0.12 – -0.03
Injunctive inclusivity norms x Descriptive inclusivity norms			0.06	0.02 – 0.10
Observations	1005		1005	
R^2 Bayes	0.070		0.078	

Table S19.
Hierarchical Bayesian regression results (German sample)

	Tolerance			
Predictors	*Estimates*	*CI (95%)*	*Estimates*	*CI (95%)*
Injunctive inclusivity norms	0.19	0.11 – 0.27	0.08	-0.08 – 0.22
Descriptive inclusivity norms	0.06	-0.00 – 0.13	-0.10	-0.29 – 0.09
Topic importance	0.05	0.00 – 0.10	0.05	0.00 – 0.10
Opinion extremity	0.03	-0.04 – 0.10	0.02	-0.05 – 0.09
Strength of disapproval	-0.15	-0.20 – -0.10	-0.15	-0.20 – -0.10
Injunctive inclusivity norms x Descriptive inclusivity norms			0.03	-0.00 – 0.07
Observations	1009		1009	
R^2 Bayes	0.098		0.100	

	Avoidance tendencies			
Predictors	*Estimates*	*CI (95%)*	*Estimates*	*CI (95%)*
Injunctive inclusivity norms	-0.12	-0.22 – -0.01	-0.07	-0.29 – 0.13
Descriptive inclusivity norms	0.15	0.05 – 0.24	0.20	-0.08 – 0.47
Topic importance	-0.02	-0.09 – 0.05	-0.02	-0.09 – 0.05
Opinion extremity	0.03	-0.07 – 0.13	0.03	-0.07 – 0.13
Strength of disapproval	0.38	0.31 – 0.45	0.38	0.31 – 0.45
Injunctive inclusivity norms x Descriptive inclusivity norms			-0.01	-0.06 – 0.04
Observations	1009		1009	
R^2 Bayes	0.135		0.135	

	Cooperation willingness			
Predictors	*Estimates*	*CI (95%)*	*Estimates*	*CI (95%)*
Injunctive inclusivity norms	0.25	0.17 – 0.34	0.11	-0.07 – 0.28
Descriptive inclusivity norms	0.02	-0.06 – 0.10	-0.18	-0.41 – 0.03
Topic importance	0.20	0.14 – 0.26	0.20	0.14 – 0.25
Opinion extremity	-0.01	-0.10 – 0.07	-0.02	-0.10 – 0.06
Strength of disapproval	-0.10	-0.16 – -0.05	-0.11	-0.16 – -0.05
Injunctive inclusivity norms x Descriptive inclusivity norms			0.04	0.00 – 0.08
Observations	1009		1009	
R^2 Bayes	0.121		0.125	

Table S20.
Hierarchical Bayesian regression results (Greek sample)

	**Tolerance**			
Predictors	*Estimates*	*CI (95%)*	*Estimates*	*CI (95%)*
Injunctive inclusivity norms	0.08	-0.00 – 0.16	0.03	-0.12 – 0.18
Descriptive inclusivity norms	0.13	0.05 – 0.21	0.06	-0.15 – 0.27
Topic importance	0.05	-0.01 – 0.10	0.05	-0.01 – 0.10
Opinion extremity	-0.04	-0.12 – 0.04	-0.04	-0.12 – 0.04
Strength of disapproval	-0.14	-0.19 – -0.09	-0.15	-0.20 – -0.09
Injunctive inclusivity norms x Descriptive inclusivity norms			0.02	-0.02 – 0.05
Observations	1009		1009	
R^2 Bayes	0.075		0.077	
	**Avoidance tendencies**			
Predictors	*Estimates*	*CI (95%)*	*Estimates*	*CI (95%)*
Injunctive inclusivity norms	-0.05	-0.16 – 0.05	-0.28	-0.47 – -0.09
Descriptive inclusivity norms	0.00	-0.10 – 0.11	-0.33	-0.60 – -0.07
Topic importance	-0.03	-0.10 – 0.04	-0.03	-0.10 – 0.04
Opinion extremity	0.06	-0.04 – 0.16	0.04	-0.06 – 0.14
Strength of disapproval	0.29	0.22 – 0.35	0.28	0.21 – 0.35
Injunctive inclusivity norms x Descriptive inclusivity norms			0.07	0.02 – 0.12
Observations	1009		1009	
R^2 Bayes	0.094		0.101	
	**Cooperation willingness**			
Predictors	*Estimates*	*CI (95%)*	*Estimates*	*CI (95%)*
Injunctive inclusivity norms	0.10	0.01 – 0.19	0.03	-0.15 – 0.20
Descriptive inclusivity norms	0.13	0.05 – 0.22	0.02	-0.22 – 0.26
Topic importance	0.13	0.07 – 0.19	0.13	0.07 – 0.19
Opinion extremity	-0.05	-0.14 – 0.04	-0.05	-0.15 – 0.04
Strength of disapproval	-0.15	-0.21 – -0.10	-0.16	-0.21 – -0.10
Injunctive inclusivity norms x Descriptive inclusivity norms			0.02	-0.02 – 0.07
Observations	1009		1009	
R^2 Bayes	0.080		0.081	

Table S21.
Hierarchical Bayesian regression results (Hungarian sample)

		**Tolerance**		
Predictors	*Estimates*	*CI (95%)*	*Estimates*	*CI (95%)*
Injunctive inclusivity norms	0.09	0.01 – 0.17	-0.06	-0.19 – 0.08
Descriptive inclusivity norms	0.01	-0.07 – 0.08	-0.18	-0.35 – -0.02
Topic importance	0.04	-0.00 – 0.08	0.04	-0.00 – 0.08
Opinion extremity	0.06	-0.02 – 0.13	0.05	-0.02 – 0.13
Strength of disapproval	-0.14	-0.18 – -0.09	-0.14	-0.18 – -0.10
Injunctive inclusivity norms x Descriptive inclusivity norms			0.04	0.01 – 0.08
Observations	1005		1005	
R^2 Bayes	0.050		0.056	
		**Avoidance tendencies**		
Predictors	*Estimates*	*CI (95%)*	*Estimates*	*CI (95%)*
Injunctive inclusivity norms	-0.08	-0.18 – 0.03	-0.20	-0.39 – -0.01
Descriptive inclusivity norms	0.09	-0.00 – 0.19	-0.06	-0.29 – 0.15
Topic importance	0.02	-0.04 – 0.08	0.02	-0.04 – 0.08
Opinion extremity	0.02	-0.08 – 0.13	0.02	-0.09 – 0.12
Strength of disapproval	0.22	0.16 – 0.28	0.22	0.16 – 0.28
Injunctive inclusivity norms x Descriptive inclusivity norms			0.04	-0.01 – 0.08
Observations	1005		1005	
R^2 Bayes	0.073		0.077	
		**Cooperation willingness**		
Predictors	*Estimates*	*CI (95%)*	*Estimates*	*CI (95%)*
Injunctive inclusivity norms	0.02	-0.07 – 0.11	-0.11	-0.27 – 0.05
Descriptive inclusivity norms	0.09	0.00 – 0.17	-0.08	-0.28 – 0.11
Topic importance	0.11	0.06 – 0.16	0.11	0.06 – 0.16
Opinion extremity	0.01	-0.08 – 0.10	0.01	-0.09 – 0.10
Strength of disapproval	-0.10	-0.15 – -0.04	-0.10	-0.15 – -0.04
Injunctive inclusivity norms x Descriptive inclusivity norms			0.04	0.00 – 0.08
Observations	1005		1005	
R^2 Bayes	0.038		0.043	

Table S22.
Hierarchical Bayesian regression results (Italian sample)

	Tolerance			
Predictors	*Estimates*	*CI (95%)*	*Estimates*	*CI (95%)*
Injunctive inclusivity norms	0.12	0.05 – 0.19	-0.05	-0.18 – 0.08
Descriptive inclusivity norms	0.09	0.03 – 0.16	-0.22	-0.44 – -0.01
Topic importance	0.02	-0.03 – 0.08	0.02	-0.04 – 0.07
Opinion extremity	-0.04	-0.11 – 0.03	-0.04	-0.11 – 0.02
Strength of disapproval	-0.12	-0.17 – -0.07	-0.12	-0.17 – -0.07
Injunctive inclusivity norms x Descriptive inclusivity norms			0.06	0.02 – 0.09
Observations	1003		1003	
R^2 Bayes	0.081		0.091	
	**Avoidance tendencies**			
Predictors	*Estimates*	*CI (95%)*	*Estimates*	*CI (95%)*
Injunctive inclusivity norms	-0.13	-0.22 – -0.03	0.00	-0.18 – 0.18
Descriptive inclusivity norms	0.07	-0.02 – 0.17	0.31	0.01 – 0.61
Topic importance	0.02	-0.06 – 0.10	0.02	-0.06 – 0.10
Opinion extremity	0.07	-0.03 – 0.16	0.07	-0.03 – 0.16
Strength of disapproval	0.26	0.19 – 0.33	0.26	0.20 – 0.33
Injunctive inclusivity norms x Descriptive inclusivity norms			-0.04	-0.09 – 0.01
Observations	1003		1003	
R^2 Bayes	0.085		0.087	
	**Cooperation willingness**			
Predictors	*Estimates*	*CI (95%)*	*Estimates*	*CI (95%)*
Injunctive inclusivity norms	0.18	0.10 – 0.26	0.05	-0.08 – 0.20
Descriptive inclusivity norms	0.07	-0.00 – 0.14	-0.16	-0.40 – 0.09
Topic importance	0.16	0.10 – 0.22	0.16	0.09 – 0.22
Opinion extremity	0.00	-0.07 – 0.08	0.00	-0.07 – 0.08
Strength of disapproval	-0.09	-0.15 – -0.04	-0.10	-0.15 – -0.04
Injunctive inclusivity norms x Descriptive inclusivity norms			0.04	-0.00 – 0.08
Observations	1003		1003	
R^2 Bayes	0.095		0.100	

Table S23.
Hierarchical Bayesian regression results (Dutch sample)

	Tolerance			
Predictors	*Estimates*	*CI (95%)*	*Estimates*	*CI (95%)*
Injunctive inclusivity norms	0.11	0.02 – 0.20	-0.03	-0.21 – 0.15
Descriptive inclusivity norms	0.06	-0.02 – 0.13	-0.15	-0.40 – 0.09
Topic importance	0.07	0.03 – 0.10	0.06	0.03 – 0.10
Opinion extremity	-0.04	-0.11 – 0.04	-0.04	-0.12 – 0.03
Strength of disapproval	0.02	-0.01 – 0.06	0.02	-0.02 – 0.06
Injunctive inclusivity norms x Descriptive inclusivity norms			0.04	-0.01 – 0.09
Observations	1005		1005	
R^2 Bayes	0.044		0.048	

	Avoidance tendencies			
Predictors	*Estimates*	*CI (95%)*	*Estimates*	*CI (95%)*
Injunctive inclusivity norms	-0.15	-0.27 – -0.02	-0.06	-0.33 – 0.21
Descriptive inclusivity norms	0.07	-0.03 – 0.19	0.19	-0.16 – 0.55
Topic importance	-0.06	-0.11 – -0.01	-0.06	-0.11 – -0.01
Opinion extremity	0.21	0.11 – 0.33	0.22	0.11 – 0.33
Strength of disapproval	0.03	-0.03 – 0.08	0.03	-0.03 – 0.08
Injunctive inclusivity norms x Descriptive inclusivity norms			-0.02	-0.09 – 0.04
Observations	1005		1005	
R^2 Bayes	0.032		0.034	

	Cooperation willingness			
Predictors	*Estimates*	*CI (95%)*	*Estimates*	*CI (95%)*
Injunctive inclusivity norms	0.17	0.07 – 0.27	-0.30	-0.51 – -0.09
Descriptive inclusivity norms	0.07	-0.02 – 0.15	-0.61	-0.88 – -0.32
Topic importance	0.17	0.14 – 0.21	0.17	0.13 – 0.21
Opinion extremity	0.02	-0.07 – 0.11	-0.01	-0.09 – 0.08
Strength of disapproval	0.05	0.01 – 0.10	0.05	0.00 – 0.09
Injunctive inclusivity norms x Descriptive inclusivity norms			0.13	0.08 – 0.18
Observations	1005		1005	
R^2 Bayes	0.111		0.132	

Table S24.
Hierarchical Bayesian regression results (Polish sample)

	Tolerance			
Predictors	*Estimates*	*CI (95%)*	*Estimates*	*CI (95%)*
Injunctive inclusivity norms	0.12	0.04 – 0.20	0.02	-0.14 – 0.17
Descriptive inclusivity norms	0.04	-0.03 – 0.12	-0.12	-0.34 – 0.10
Topic importance	-0.04	-0.08 – 0.01	-0.04	-0.09 – 0.01
Opinion extremity	-0.03	-0.10 – 0.04	-0.03	-0.10 – 0.05
Strength of disapproval	-0.06	-0.10 – -0.01	-0.06	-0.10 – -0.01
Injunctive inclusivity norms x Descriptive inclusivity norms			0.03	-0.01 – 0.07
Observations	1003		1003	
R^2 Bayes	0.041		0.045	
	**Avoidance tendencies**			
Predictors	*Estimates*	*CI (95%)*	*Estimates*	*CI (95%)*
Injunctive inclusivity norms	-0.17	-0.27 – -0.06	-0.22	-0.43 – 0.00
Descriptive inclusivity norms	0.16	0.06 – 0.25	0.08	-0.22 – 0.38
Topic importance	0.04	-0.03 – 0.10	0.04	-0.03 – 0.10
Opinion extremity	0.12	0.02 – 0.22	0.12	0.02 – 0.22
Strength of disapproval	0.11	0.04 – 0.17	0.11	0.04 – 0.17
Injunctive inclusivity norms x Descriptive inclusivity norms			0.01	-0.04 – 0.07
Observations	1003		1003	
R^2 Bayes	0.039		0.040	
	**Cooperation willingness**			
Predictors	*Estimates*	*CI (95%)*	*Estimates*	*CI (95%)*
Injunctive inclusivity norms	0.21	0.12 – 0.29	0.02	-0.15 – 0.19
Descriptive inclusivity norms	0.05	-0.03 – 0.13	-0.23	-0.47 – 0.01
Topic importance	0.06	0.01 – 0.11	0.06	0.01 – 0.10
Opinion extremity	0.02	-0.06 – 0.10	0.02	-0.06 – 0.10
Strength of disapproval	-0.01	-0.06 – 0.04	-0.01	-0.06 – 0.04
Injunctive inclusivity norms x Descriptive inclusivity norms			0.05	0.01 – 0.09
Observations	1003		1003	
R^2 Bayes	0.066		0.072	

Table S25.
Hierarchical Bayesian regression results (Spanish sample)

	Tolerance			
Predictors	*Estimates*	*CI (95%)*	*Estimates*	*CI (95%)*
Injunctive inclusivity norms	0.17	0.09 – 0.24	0.18	0.02 – 0.33
Descriptive inclusivity norms	0.05	-0.03 – 0.13	0.07	-0.16 – 0.29
Topic importance	-0.01	-0.07 – 0.05	-0.01	-0.07 – 0.05
Opinion extremity	-0.05	-0.13 – 0.03	-0.05	-0.13 – 0.04
Strength of disapproval	-0.15	-0.19 – -0.10	-0.14	-0.19 – -0.10
Injunctive inclusivity norms x Descriptive inclusivity norms			-0.00	-0.04 – 0.04
Observations	999		999	
R^2 Bayes	0.088		0.089	

	Avoidance tendencies			
Predictors	*Estimates*	*CI (95%)*	*Estimates*	*CI (95%)*
Injunctive inclusivity norms	-0.15	-0.26 – -0.05	-0.31	-0.49 – -0.11
Descriptive inclusivity norms	0.16	0.06 – 0.26	-0.08	-0.35 – 0.21
Topic importance	0.05	-0.02 – 0.12	0.05	-0.02 – 0.12
Opinion extremity	0.02	-0.08 – 0.12	0.01	-0.10 – 0.12
Strength of disapproval	0.23	0.17 – 0.29	0.23	0.17 – 0.29
Injunctive inclusivity norms x Descriptive inclusivity norms			0.05	-0.01 – 0.09
Observations	999		999	
R^2 Bayes	0.082		0.086	

	Cooperation willingness			
Predictors	*Estimates*	*CI (95%)*	*Estimates*	*CI (95%)*
Injunctive inclusivity norms	0.11	0.03 – 0.20	0.16	-0.01 – 0.32
Descriptive inclusivity norms	0.09	0.01 – 0.17	0.16	-0.07 – 0.39
Topic importance	0.13	0.08 – 0.19	0.14	0.08 – 0.19
Opinion extremity	0.07	-0.02 – 0.15	0.07	-0.01 – 0.16
Strength of disapproval	-0.10	-0.14 – -0.05	-0.09	-0.14 – -0.05
Injunctive inclusivity norms x Descriptive inclusivity norms			-0.01	-0.05 – 0.03
Observations	999		999	
R^2 Bayes	0.078		0.079	

Table S26.
Hierarchical Bayesian regression results (Swedish sample)

	Tolerance			
Predictors	*Estimates*	*CI (95%)*	*Estimates*	*CI (95%)*
Injunctive inclusivity norms	0.16	0.09 – 0.25	0.06	-0.10 – 0.22
Descriptive inclusivity norms	0.04	-0.02 – 0.11	-0.12	-0.35 – 0.12
Topic importance	-0.06	-0.12 – -0.00	-0.06	-0.12 – -0.01
Opinion extremity	0.06	-0.02 – 0.14	0.06	-0.02 – 0.14
Strength of disapproval	-0.07	-0.12 – -0.02	-0.07	-0.12 – -0.02
Injunctive inclusivity norms x Descriptive inclusivity norms			0.03	-0.01 – 0.07
Observations	998		998	
R^2 Bayes	0.051		0.055	

	Avoidance tendencies			
Predictors	*Estimates*	*CI (95%)*	*Estimates*	*CI (95%)*
Injunctive inclusivity norms	-0.12	-0.22 – -0.02	-0.10	-0.30 – 0.10
Descriptive inclusivity norms	-0.01	-0.10 – 0.08	0.02	-0.27 – 0.29
Topic importance	0.05	-0.02 – 0.12	0.05	-0.02 – 0.12
Opinion extremity	0.02	-0.08 – 0.11	0.02	-0.08 – 0.11
Strength of disapproval	0.22	0.16 – 0.28	0.22	0.16 – 0.28
Injunctive inclusivity norms x Descriptive inclusivity norms			-0.01	-0.05 – 0.05
Observations	998		998	
R^2 Bayes	0.085		0.085	

	Cooperation willingness			
Predictors	*Estimates*	*CI (95%)*	*Estimates*	*CI (95%)*
Injunctive inclusivity norms	0.17	0.08 – 0.26	0.09	-0.08 – 0.28
Descriptive inclusivity norms	0.07	-0.01 – 0.14	-0.05	-0.30 – 0.21
Topic importance	0.06	0.01 – 0.12	0.06	0.00 – 0.12
Opinion extremity	0.08	-0.00 – 0.16	0.08	-0.00 – 0.16
Strength of disapproval	-0.08	-0.14 – -0.03	-0.09	-0.14 – -0.03
Injunctive inclusivity norms x Descriptive inclusivity norms			0.02	-0.02 – 0.07
Observations	998		998	
R^2 Bayes	0.061		0.063	

Table S27.
Hierarchical Bayesian regression results (British sample)

	**Tolerance**			
Predictors	*Estimates*	*CI (95%)*	*Estimates*	*CI (95%)*
Injunctive inclusivity norms	0.06	-0.03 – 0.14	-0.08	-0.23 – 0.07
Descriptive inclusivity norms	0.07	-0.00 – 0.15	-0.12	-0.31 – 0.07
Topic importance	-0.01	-0.06 – 0.04	-0.02	-0.07 – 0.04
Opinion extremity	-0.02	-0.11 – 0.06	-0.02	-0.11 – 0.06
Strength of disapproval	-0.09	-0.15 – -0.04	-0.10	-0.15 – -0.04
Injunctive inclusivity norms x Descriptive inclusivity norms			0.04	0.00 – 0.07
Observations	1002		1002	
R^2 Bayes	0.043		0.048	
	**Avoidance tendencies**			
Predictors	*Estimates*	*CI (95%)*	*Estimates*	*CI (95%)*
Injunctive inclusivity norms	-0.05	-0.16 – 0.06	-0.13	-0.33 – 0.07
Descriptive inclusivity norms	-0.06	-0.17 – 0.04	-0.19	-0.44 – 0.07
Topic importance	0.03	-0.04 – 0.11	0.03	-0.04 – 0.10
Opinion extremity	0.11	-0.01 – 0.22	0.10	-0.01 – 0.22
Strength of disapproval	0.25	0.17 – 0.32	0.25	0.18 – 0.32
Injunctive inclusivity norms x Descriptive inclusivity norms			0.02	-0.02 – 0.07
Observations	1002		1002	
R^2 Bayes	0.095		0.097	
	**Cooperation willingness**			
Predictors	*Estimates*	*CI (95%)*	*Estimates*	*CI (95%)*
Injunctive inclusivity norms	0.09	0.00 – 0.18	0.01	-0.16 – 0.18
Descriptive inclusivity norms	0.13	0.05 – 0.22	0.02	-0.20 – 0.22
Topic importance	0.11	0.05 – 0.17	0.10	0.05 – 0.17
Opinion extremity	-0.00	-0.10 – 0.09	-0.00	-0.10 – 0.09
Strength of disapproval	-0.05	-0.11 – 0.00	-0.05	-0.11 – 0.00
Injunctive inclusivity norms x Descriptive inclusivity norms			0.02	-0.02 – 0.06
Observations	1002		1002	
R^2 Bayes	0.063		0.065	

Table S28.
Test of differences in the strength of effects between injunctive inclusivity norms and descriptive inclusivity norms on the outcomes tolerance, avoidance tendencies, and cooperation willingness

**Czechia**		
Outcome	*Estimate*	*CI (95%)*
Tolerance	-0.04	-0.18 – 0.1
Avoidance tendencies	-0.04	-0.22 – 0.13
Cooperation willingness	0	-0.17 – 0.17
**Finland**		
Outcome	*Estimate*	*CI (95%)*
Tolerance	0	-0.15 – 0.14
Avoidance tendencies	-0.15	-0.33 – 0.03
Cooperation willingness	0.03	-0.14 – 0.19
**France**		
Outcome	*Estimate*	*CI (95%)*
Tolerance	0.14	0.03 – 0.26
Avoidance tendencies	-0.12	-0.28 – 0.04
Cooperation willingness	0.13	-0.01 – 0.25
**Germany**		
Outcome	*Estimate*	*CI (95%)*
Tolerance	0.13	0 – 0.26
Avoidance tendencies	-0.26	-0.44 – -0.08
Cooperation willingness	0.23	0.08 – 0.38
**Greece**		
Outcome	*Estimate*	*CI (95%)*
Tolerance	-0.05	-0.02 – 0.09
Avoidance tendencies	-0.06	-0.24 – 0.13
Cooperation willingness	-0.03	-0.19 – 0.13
**Hungary**		
Outcome	*Estimate*	*CI (95%)*
Tolerance	0.08	0.03 – 0.26
Avoidance tendencies	-0.17	-0.37 – 0.03
Cooperation willingness	-0.06	-0.23 – 0.1
**Italy**		
Outcome	*Estimate*	*CI (95%)*
Tolerance	0.03	-0.09 – 0.14
Avoidance tendencies	-0.2	-0.37 – -0.03
Cooperation willingness	0.11	-0.03 – 0.25
**The Netherlands**		
Outcome	*Estimate*	*CI (95%)*
Tolerance	0.05	-0.1 – 0.2
Avoidance tendencies	-0.22	-0.44 – -0.01
Cooperation willingness	0.1	-0.07 – 0.27
**Poland**		
Outcome	*Estimate*	*CI (95%)*
Tolerance	0.08	-0.06 – 0.21
Avoidance tendencies	-0.32	-0.51 – -0.13
Cooperation willingness	0.16	0.01 – 0.3

**Spain**		
Outcome	*Estimate*	*CI (95%)*
Tolerance	0.11	-0.03 – 0.26
Avoidance tendencies	-0.31	-0.5 – -0.13
Cooperation willingness	0.02	-0.13 – 0.17
**Sweden**		
Outcome	*Estimate*	*CI (95%)*
Tolerance	0.12	-0.01 – 0.25
Avoidance tendencies	-0.1	0.27 – 0.06
Cooperation willingness	0.11	-0.04 – 0.26
**UK**		
Outcome	*Estimate*	*CI (95%)*
Tolerance	-0.02	-0.16 – 0.13
Avoidance tendencies	0.02	-0.17 – 0.21
Cooperation willingness	-0.03	-0.19 – 0.12

Boundary Conditions

Strength of Disapproval

Table S29.
Bayesian moderated multiple regression results (Czech sample)

	Tolerance	
Predictors	*Estimates*	*CI (95%)*
Injunctive inclusivity norms	0.01	-0.19 – 0.22
Strength of disapproval	-0.32	-0.48 – -0.17
Descriptive inclusivity norms	0.05	-0.16 – 0.26
Topic importance	0.05	0.00 – 0.10
Opinion extremity	0.03	-0.05 – 0.09
Injunctive inclusivity norms x Strength of disapproval	0.01	-0.03 – 0.05
Descriptive inclusivity norms x Strength of disapproval	0.01	-0.03 – 0.06
Observations	1002	
R^2 Bayes	0.112	
BF_{01}	127.09	
	**Avoidance tendencies**	
Predictors	*Estimates*	*CI (95%)*
Injunctive inclusivity norms	0.01	-0.26 – 0.28
Strength of disapproval	0.37	0.17 – 0.56
Descriptive inclusivity norms	0.02	-0.24 – 0.29
Topic importance	-0.03	-0.09 – 0.03
Opinion extremity	0.02	-0.07 – 0.11
Injunctive inclusivity norms x Strength of disapproval	-0.01	-0.06 – 0.04
Descriptive inclusivity norms x Strength of disapproval	-0.00	-0.06 – 0.05
Observations	1002	
R^2 Bayes	0.122	
BF_{01}	255.20	
	**Cooperation willingness**	
Predictors	*Estimates*	*CI (95%)*
Injunctive inclusivity norms	0.09	-0.14 – 0.32

Strength of disapproval	-0.16	-0.35 – 0.02
Descriptive inclusivity norms	0.15	-0.10 – 0.39
Topic importance	0.09	0.04 – 0.15
Opinion extremity	0.01	-0.07 – 0.09
Injunctive inclusivity norms x Strength of disapproval	0.01	-0.04 – 0.05
Descriptive inclusivity norms x Strength of disapproval	-0.00	-0.05 – 0.04
Observations	1002	
R^2 Bayes	0.082	
BF_{01}	341.31	

Table S30.
Bayesian moderated multiple regression results (Finnish sample)

	**Tolerance**	
Predictors	*Estimates*	*CI (95%)*
Injunctive inclusivity norms	-0.03	-0.24 – 0.20
Strength of disapproval	-0.67	-0.85 – -0.48
Descriptive inclusivity norms	-0.16	-0.37 – 0.06
Topic importance	0.03	-0.02 – 0.07
Opinion extremity	-0.04	-0.12 – 0.05
Injunctive inclusivity norms x Strength of disapproval	0.02	-0.02 – 0.06
Descriptive inclusivity norms x Strength of disapproval	0.05	0.01 – 0.10
Observations	1001	
R^2 Bayes	0.229	
BF_{01}	0.14	
	**Avoidance tendencies**	
Predictors	*Estimates*	*CI (95%)*
Injunctive inclusivity norms	-0.05	-0.33 – 0.23
Strength of disapproval	0.88	0.65 – 1.11
Descriptive inclusivity norms	0.42	0.14 – 0.68
Topic importance	-0.04	-0.10 – 0.02
Opinion extremity	0.01	-0.10 – 0.12
Injunctive inclusivity norms x Strength of disapproval	-0.02	-0.08 – 0.03
Descriptive inclusivity norms x Strength of disapproval	-0.09	-0.14 – -0.04
Observations	1001	
R^2 Bayes	0.213	
BF_{01}	0.01	
	**Cooperation willingness**	
Predictors	*Estimates*	*CI (95%)*
Injunctive inclusivity norms	0.08	-0.18 – 0.35
Strength of disapproval	-0.39	-0.60 – -0.18
Descriptive inclusivity norms	0.01	-0.25 – 0.26
Topic importance	0.16	0.11 – 0.21
Opinion extremity	0.03	-0.07 – 0.13

Injunctive inclusivity norms x Strength of disapproval	0.01	-0.04 – 0.07
Descriptive inclusivity norms x Strength of disapproval	0.02	-0.03 – 0.08
Observations	1001	
R^2 Bayes	0.118	
BF_{01}	69.85	

Table S31.
Bayesian moderated multiple regression results (French sample)

	Tolerance	
Predictors	*Estimates*	*CI (95%)*
Injunctive inclusivity norms	0.19	0.01 – 0.38
Strength of disapproval	-0.17	-0.32 – -0.02
Descriptive inclusivity norms	-0.07	-0.24 – 0.10
Topic importance	0.04	-0.01 – 0.10
Opinion extremity	0.01	-0.05 – 0.08
Injunctive inclusivity norms x Strength of disapproval	-0.01	-0.04 – 0.03
Descriptive inclusivity norms x Strength of disapproval	0.02	-0.01 – 0.05
Observations	1005	
R^2 Bayes	0.079	
BF_{01}	340.83	
	**Avoidance tendencies**	
Predictors	*Estimates*	*CI (95%)*
Injunctive inclusivity norms	-0.16	-0.41 – 0.09
Strength of disapproval	0.20	-0.01 – 0.41
Descriptive inclusivity norms	0.25	0.02 – 0.48
Topic importance	-0.01	-0.09 – 0.07
Opinion extremity	0.11	0.02 – 0.21
Injunctive inclusivity norms x Strength of disapproval	0.03	-0.02 – 0.07
Descriptive inclusivity norms x Strength of disapproval	-0.03	-0.08 – 0.01
Observations	1005	
R^2 Bayes	0.066	
BF_{01}	108.27	
	**Cooperation willingness**	
Predictors	*Estimates*	*CI (95%)*
Injunctive inclusivity norms	0.27	0.07 – 0.48
Strength of disapproval	-0.10	-0.27 – 0.07
Descriptive inclusivity norms	-0.12	-0.30 – 0.07
Topic importance	0.12	0.05 – 0.18
Opinion extremity	0.07	-0.01 – 0.15

Injunctive inclusivity norms x Strength of disapproval	-0.02	-0.06 – 0.02
Descriptive inclusivity norms x Strength of disapproval	0.03	-0.00 – 0.07
Observations	1005	
R^2 Bayes	0.073	
BF_{01}	105.09	

Table S32.
Bayesian moderated multiple regression results (German sample)

	**Tolerance**	
Predictors	*Estimates*	*CI (95%)*
Injunctive inclusivity norms	-0.04	-0.28 – 0.20
Strength of disapproval	-0.58	-0.76 – -0.40
Descriptive inclusivity norms	-0.22	-0.43 – -0.01
Topic importance	0.05	0.00 – 0.10
Opinion extremity	0.04	-0.03 – 0.11
Injunctive inclusivity norms x Strength of disapproval	0.04	-0.00 – 0.09
Descriptive inclusivity norms x Strength of disapproval	0.05	0.01 – 0.09
Observations	1009	
R^2 Bayes	0.122	
BF_{01}	0.00	

	**Avoidance tendencies**	
Predictors	*Estimates*	*CI (95%)*
Injunctive inclusivity norms	0.22	-0.11 – 0.55
Strength of disapproval	0.91	0.66 – 1.16
Descriptive inclusivity norms	0.43	0.15 – 0.72
Topic importance	-0.02	-0.09 – 0.05
Opinion extremity	0.01	-0.08 – 0.11
Injunctive inclusivity norms x Strength of disapproval	-0.06	-0.12 – -0.00
Descriptive inclusivity norms x Strength of disapproval	-0.05	-0.11 – -0.00
Observations	1009	
R^2 Bayes	0.153	
BF_{01}	0.01	

	**Cooperation willingness**	
Predictors	*Estimates*	*CI (95%)*
Injunctive inclusivity norms	-0.09	-0.36 – 0.18
Strength of disapproval	-0.61	-0.82 – -0.41
Descriptive inclusivity norms	-0.23	-0.47 – 0.01
Topic importance	0.19	0.14 – 0.25
Opinion extremity	0.00	-0.08 – 0.08

Injunctive inclusivity norms x Strength of disapproval	0.06	0.01 – 0.11
Descriptive inclusivity norms x Strength of disapproval	0.05	0.00 – 0.09
Observations	1009	
R^2 Bayes	0.146	
BF_{01}	0.00	

Table S33.
Bayesian moderated multiple regression results (Greek sample)

	**Tolerance**	
Predictors	*Estimates*	*CI (95%)*
Injunctive inclusivity norms	0.09	-0.12 – 0.31
Strength of disapproval	-0.26	-0.41 – -0.10
Descriptive inclusivity norms	-0.05	-0.26 – 0.17
Topic importance	0.05	-0.01 – 0.10
Opinion extremity	-0.04	-0.11 – 0.04
Injunctive inclusivity norms x Strength of disapproval	-0.00	-0.05 – 0.04
Descriptive inclusivity norms x Strength of disapproval	0.04	-0.00 – 0.08
Observations	1009	
R^2 Bayes	0.081	
BF_{01}	52.85	

	**Avoidance tendencies**	
Predictors	*Estimates*	*CI (95%)*
Injunctive inclusivity norms	-0.05	-0.31 – 0.21
Strength of disapproval	0.21	0.00 – 0.41
Descriptive inclusivity norms	-0.11	-0.38 – 0.16
Topic importance	-0.03	-0.11 – 0.04
Opinion extremity	0.06	-0.05 – 0.17
Injunctive inclusivity norms x Strength of disapproval	-0.00	-0.05 – 0.05
Descriptive inclusivity norms x Strength of disapproval	0.02	-0.03 – 0.07
Observations	1009	
R^2 Bayes	0.097	
BF_{01}	158.24	

	**Cooperation willingness**	
Predictors	*Estimates*	*CI (95%)*
Injunctive inclusivity norms	0.12	-0.11 – 0.35
Strength of disapproval	-0.21	-0.38 – -0.04
Descriptive inclusivity norms	0.02	-0.21 – 0.26
Topic importance	0.13	0.07 – 0.19
Opinion extremity	-0.05	-0.14 – 0.04

Injunctive inclusivity norms x Strength of disapproval	-0.01	-0.05 – 0.04
Descriptive inclusivity norms x Strength of disapproval	0.02	-0.02 – 0.07
Observations	1009	
R^2 Bayes	0.082	
BF_{01}	213.83	

Table S34.
Bayesian moderated multiple regression results (Hungarian sample)

	**Tolerance**	
Predictors	*Estimates*	*CI (95%)*
Injunctive inclusivity norms	0.01	-0.19 – 0.22
Strength of disapproval	-0.21	-0.33 – -0.09
Descriptive inclusivity norms	-0.00	-0.20 – 0.19
Topic importance	0.04	-0.01 – 0.08
Opinion extremity	0.06	-0.01 – 0.14
Injunctive inclusivity norms x Strength of disapproval	0.02	-0.02 – 0.06
Descriptive inclusivity norms x Strength of disapproval	0.00	-0.03 – 0.04
Observations	1005	
R^2 Bayes	0.053	
BF_{01}	274.46	
	**Avoidance tendencies**	
Predictors	*Estimates*	*CI (95%)*
Injunctive inclusivity norms	0.22	-0.05 – 0.51
Strength of disapproval	0.39	0.23 – 0.56
Descriptive inclusivity norms	-0.01	-0.27 – 0.25
Topic importance	0.03	-0.03 – 0.08
Opinion extremity	0.02	-0.08 – 0.12
Injunctive inclusivity norms x Strength of disapproval	-0.06	-0.11 – -0.01
Descriptive inclusivity norms x Strength of disapproval	0.02	-0.03 – 0.07
Observations	1005	
R^2 Bayes	0.081	
BF_{01}	15.17	
	**Cooperation willingness**	
Predictors	*Estimates*	*CI (95%)*
Injunctive inclusivity norms	-0.15	-0.38 – 0.09
Strength of disapproval	-0.18	-0.32 – -0.03
Descriptive inclusivity norms	0.17	-0.06 – 0.39
Topic importance	0.10	0.05 – 0.15
Opinion extremity	0.02	-0.07 – 0.11

Injunctive inclusivity norms x Strength of disapproval	0.04	-0.01 – 0.08
Descriptive inclusivity norms x Strength of disapproval	-0.02	-0.06 – 0.03
Observations	1005	
R^2 Bayes	0.042	
BF_{01}	142.12	

Table S35.
Bayesian moderated multiple regression results (Italian sample)

	Tolerance	
Predictors	*Estimates*	*CI (95%)*
Injunctive inclusivity norms	0.10	-0.09 – 0.30
Strength of disapproval	-0.18	-0.33 – -0.02
Descriptive inclusivity norms	0.04	-0.15 – 0.22
Topic importance	0.02	-0.03 – 0.08
Opinion extremity	-0.04	-0.11 – 0.03
Injunctive inclusivity norms x Strength of disapproval	0.00	-0.03 – 0.04
Descriptive inclusivity norms x Strength of disapproval	0.01	-0.02 – 0.04
Observations	1003	
R^2 Bayes	0.083	
BF_{01}	393.45	

	Avoidance tendencies	
Predictors	*Estimates*	*CI (95%)*
Injunctive inclusivity norms	-0.09	-0.37 – 0.20
Strength of disapproval	0.41	0.19 – 0.63
Descriptive inclusivity norms	0.23	-0.04 – 0.50
Topic importance	0.02	-0.06 – 0.09
Opinion extremity	0.07	-0.03 – 0.16
Injunctive inclusivity norms x Strength of disapproval	-0.01	-0.06 – 0.05
Descriptive inclusivity norms x Strength of disapproval	-0.03	-0.08 – 0.02
Observations	1003	
R^2 Bayes	0.088	
BF_{01}	67.27	

	Cooperation willingness	
Predictors	*Estimates*	*CI (95%)*
Injunctive inclusivity norms	0.33	0.09 – 0.55
Strength of disapproval	-0.09	-0.27 – 0.09
Descriptive inclusivity norms	-0.12	-0.33 – 0.10
Topic importance	0.16	0.10 – 0.22
Opinion extremity	0.00	-0.07 – 0.08

Injunctive inclusivity norms x Strength of disapproval	-0.03	-0.07 – 0.01
Descriptive inclusivity norms x Strength of disapproval	0.04	-0.00 – 0.08
Observations	1003	
R^2 Bayes	0.100	
BF_{01}	81.06	

Table S36.
Bayesian moderated multiple regression results (Dutch sample)

	Tolerance	
Predictors	*Estimates*	*CI (95%)*
Injunctive inclusivity norms	0.08	-0.15 – 0.30
Strength of disapproval	0.02	-0.14 – 0.19
Descriptive inclusivity norms	0.09	-0.11 – 0.30
Topic importance	0.07	0.03 – 0.10
Opinion extremity	-0.04	-0.12 – 0.04
Injunctive inclusivity norms x Strength of disapproval	0.01	-0.03 – 0.05
Descriptive inclusivity norms x Strength of disapproval	-0.01	-0.05 – 0.03
Observations	1005	
R^2 Bayes	0.046	
BF_{01}	395.91	
	**Avoidance tendencies**	
Predictors	*Estimates*	*CI (95%)*
Injunctive inclusivity norms	-0.32	-0.64 – 0.00
Strength of disapproval	-0.16	-0.40 – 0.08
Descriptive inclusivity norms	0.05	-0.25 – 0.34
Topic importance	-0.06	-0.11 – -0.01
Opinion extremity	0.21	0.11 – 0.32
Injunctive inclusivity norms x Strength of disapproval	0.04	-0.02 – 0.10
Descriptive inclusivity norms x Strength of disapproval	0.00	-0.05 – 0.06
Observations	1005	
R^2 Bayes	0.037	
BF_{01}	295.22	
	**Cooperation willingness**	
Predictors	*Estimates*	*CI (95%)*
Injunctive inclusivity norms	0.18	-0.09 – 0.43
Strength of disapproval	0.08	-0.12 – 0.27
Descriptive inclusivity norms	0.10	-0.15 – 0.33
Topic importance	0.17	0.13 – 0.21
Opinion extremity	0.02	-0.07 – 0.11

Injunctive inclusivity norms x Strength of disapproval	-0.00	-0.05 – 0.05
Descriptive inclusivity norms x Strength of disapproval	-0.01	-0.05 – 0.04
Observations	1005	
R^2 Bayes	0.113	
BF_{01}	334.25	

Table S37.
Bayesian moderated multiple regression results (Polish sample)

	**Tolerance**	
Predictors	*Estimates*	*CI (95%)*
Injunctive inclusivity norms	-0.09	-0.35 – 0.17
Strength of disapproval	-0.38	-0.58 – -0.19
Descriptive inclusivity norms	-0.11	-0.33 – 0.13
Topic importance	-0.03	-0.08 – 0.02
Opinion extremity	-0.02	-0.10 – 0.06
Injunctive inclusivity norms x Strength of disapproval	0.04	-0.01 – 0.08
Descriptive inclusivity norms x Strength of disapproval	0.03	-0.01 – 0.07
Observations	1003	
R^2 Bayes	0.055	
BF_{01}	1.13	

	**Avoidance tendencies**	
Predictors	*Estimates*	*CI (95%)*
Injunctive inclusivity norms	-0.10	-0.45 – 0.24
Strength of disapproval	0.32	0.07 – 0.58
Descriptive inclusivity norms	0.34	0.02 – 0.65
Topic importance	0.03	-0.03 – 0.09
Opinion extremity	0.12	0.02 – 0.22
Injunctive inclusivity norms x Strength of disapproval	-0.01	-0.07 – 0.05
Descriptive inclusivity norms x Strength of disapproval	-0.03	-0.09 – 0.02
Observations	1003	
R^2 Bayes	0.044	
BF_{01}	41.22	

	**Cooperation willingness**	
Predictors	*Estimates*	*CI (95%)*
Injunctive inclusivity norms	0.08	-0.20 – 0.37
Strength of disapproval	-0.17	-0.37 – 0.04
Descriptive inclusivity norms	0.00	-0.25 – 0.26
Topic importance	0.06	0.02 – 0.11
Opinion extremity	0.03	-0.05 – 0.11

Injunctive inclusivity norms x Strength of disapproval	0.02	-0.03 – 0.07
Descriptive inclusivity norms x Strength of disapproval	0.01	-0.03 – 0.05
Observations	1003	
R^2 Bayes	0.070	
BF_{01}	113.44	

Table S38.
Bayesian moderated multiple regression results (Spanish sample)

	**Tolerance**	
Predictors	*Estimates*	*CI (95%)*
Injunctive inclusivity norms	0.20	-0.02 – 0.41
Strength of disapproval	-0.43	-0.59 – -0.28
Descriptive inclusivity norms	-0.34	-0.56 – -0.13
Topic importance	0.00	-0.05 – 0.06
Opinion extremity	-0.04	-0.12 – 0.04
Injunctive inclusivity norms x Strength of disapproval	-0.01	-0.05 – 0.03
Descriptive inclusivity norms x Strength of disapproval	0.08	0.04 – 0.12
Observations	999	
R^2 Bayes	0.110	
BF_{01}	0.00	

	**Avoidance tendencies**	
Predictors	*Estimates*	*CI (95%)*
Injunctive inclusivity norms	-0.16	-0.43 – 0.11
Strength of disapproval	0.41	0.21 – 0.61
Descriptive inclusivity norms	0.39	0.11 – 0.67
Topic importance	0.05	-0.03 – 0.12
Opinion extremity	0.02	-0.09 – 0.11
Injunctive inclusivity norms x Strength of disapproval	0.00	-0.05 – 0.05
Descriptive inclusivity norms x Strength of disapproval	-0.05	-0.10 – 0.01
Observations	999	
R^2 Bayes	0.089	
BF_{01}	21.99	

	**Cooperation willingness**	
Predictors	*Estimates*	*CI (95%)*
Injunctive inclusivity norms	0.17	-0.05 – 0.39
Strength of disapproval	-0.29	-0.45 – -0.12
Descriptive inclusivity norms	-0.22	-0.43 – 0.01
Topic importance	0.15	0.09 – 0.20
Opinion extremity	0.07	-0.01 – 0.15

Injunctive inclusivity norms x Strength of disapproval	-0.01	-0.05 – 0.03
Descriptive inclusivity norms x Strength of disapproval	0.06	0.02 – 0.10
Observations	999	
R^2 Bayes	0.089	
BF_{01}	1.33	

Table S39.
Bayesian moderated multiple regression results (Swedish sample)

	Tolerance	
Predictors	*Estimates*	*CI (95%)*
Injunctive inclusivity norms	0.26	0.03 – 0.50
Strength of disapproval	-0.03	-0.21 – 0.15
Descriptive inclusivity norms	-0.02	-0.22 – 0.18
Topic importance	-0.06	-0.12 – -0.00
Opinion extremity	0.06	-0.02 – 0.13
Injunctive inclusivity norms x Strength of disapproval	-0.02	-0.06 – 0.02
Descriptive inclusivity norms x Strength of disapproval	0.01	-0.02 – 0.05
Observations	998	
R^2 Bayes	0.055	
BF_{01}	333.48	

	Avoidance tendencies	
Predictors	*Estimates*	*CI (95%)*
Injunctive inclusivity norms	-0.20	-0.48 – 0.07
Strength of disapproval	0.15	-0.08 – 0.38
Descriptive inclusivity norms	-0.01	-0.25 – 0.22
Topic importance	0.05	-0.02 – 0.12
Opinion extremity	0.02	-0.08 – 0.11
Injunctive inclusivity norms x Strength of disapproval	0.02	-0.04 – 0.07
Descriptive inclusivity norms x Strength of disapproval	-0.00	-0.04 – 0.04
Observations	998	
R^2 Bayes	0.087	
BF_{01}	250.46	

	Cooperation willingness	
Predictors	*Estimates*	*CI (95%)*
Injunctive inclusivity norms	0.23	-0.02 – 0.47
Strength of disapproval	-0.02	-0.23 – 0.17
Descriptive inclusivity norms	0.07	-0.16 – 0.28
Topic importance	0.06	0.00 – 0.12
Opinion extremity	0.08	-0.00 – 0.16

Injunctive inclusivity norms x Strength of disapproval	-0.01	-0.05 – 0.03
Descriptive inclusivity norms x Strength of disapproval	-0.00	-0.04 – 0.04
Observations	998	
R^2 Bayes	0.063	
BF_{01}	343.87	

Table S40.
Bayesian moderated multiple regression results (British sample)

	Tolerance	
Predictors	*Estimates*	*CI (95%)*
Injunctive inclusivity norms	-0.02	-0.28 – 0.25
Strength of disapproval	-0.26	-0.44 – -0.09
Descriptive inclusivity norms	-0.05	-0.29 – 0.18
Topic importance	-0.01	-0.07 – 0.04
Opinion extremity	-0.02	-0.10 – 0.06
Injunctive inclusivity norms x Strength of disapproval	0.01	-0.04 – 0.06
Descriptive inclusivity norms x Strength of disapproval	0.02	-0.02 – 0.07
Observations	1002	
R^2 Bayes	0.049	
BF_{01}	37.58	
	**Avoidance tendencies**	
Predictors	*Estimates*	*CI (95%)*
Injunctive inclusivity norms	-0.02	-0.38 – 0.33
Strength of disapproval	0.48	0.24 – 0.71
Descriptive inclusivity norms	0.18	-0.14 – 0.51
Topic importance	0.03	-0.04 – 0.11
Opinion extremity	0.10	-0.01 – 0.21
Injunctive inclusivity norms x Strength of disapproval	-0.00	-0.07 – 0.07
Descriptive inclusivity norms x Strength of disapproval	-0.05	-0.11 – 0.01
Observations	1002	
R^2 Bayes	0.101	
BF_{01}	12.67	
	**Cooperation willingness**	
Predictors	*Estimates*	*CI (95%)*
Injunctive inclusivity norms	-0.11	-0.39 – 0.19
Strength of disapproval	-0.38	-0.57 – -0.19
Descriptive inclusivity norms	-0.05	-0.30 – 0.22
Topic importance	0.11	0.05 – 0.17
Opinion extremity	0.01	-0.09 – 0.10

Injunctive inclusivity norms x Strength of disapproval	0.04	-0.02 – 0.09
Descriptive inclusivity norms x Strength of disapproval	0.03	-0.01 – 0.08
Observations	1002	
R^2 Bayes	0.077	
BF_{01}	0.42	

Identification Difference Score

Table S41.
Bayesian moderated multiple regression results (Czech sample)

	**Tolerance**	
Predictors	*Estimates*	*CI (95%)*
Injunctive inclusivity norms	0.08	-0.01 – 0.16
Identification difference score	-0.06	-0.20 – 0.09
Descriptive inclusivity norms	0.11	0.03 – 0.19
Topic importance	0.06	0.01 – 0.11
Opinion extremity	0.05	-0.02 – 0.13
Strength of disapproval	-0.18	-0.23 – -0.13
Injunctive inclusivity norms x Identification difference score	-0.00	-0.04 – 0.03
Descriptive inclusivity norms x Identification difference score	-0.00	-0.04 – 0.03
Observations	1002	
R^2 Bayes	0.122	
BF_{01}	533.23	
	**Avoidance tendencies**	
Predictors	*Estimates*	*CI (95%)*
Injunctive inclusivity norms	0.01	-0.10 – 0.11
Identification difference score	0.26	0.08 – 0.46
Descriptive inclusivity norms	0.02	-0.08 – 0.12
Topic importance	-0.04	-0.10 – 0.02
Opinion extremity	-0.01	-0.10 – 0.08
Strength of disapproval	0.29	0.23 – 0.36
Injunctive inclusivity norms x Identification difference score	-0.04	-0.09 – 0.01
Descriptive inclusivity norms x Identification difference score	-0.00	-0.05 – 0.04
Observations	1002	
R^2 Bayes	0.132	
BF_{01}	35.47	
	**Cooperation willingness**	
Predictors	*Estimates*	*CI (95%)*
Injunctive inclusivity norms	0.15	0.05 – 0.24

Identification difference score	-0.03	-0.20 – 0.14
Descriptive inclusivity norms	0.10	0.00 – 0.19
Topic importance	0.10	0.05 – 0.16
Opinion extremity	0.03	-0.05 – 0.11
Strength of disapproval	-0.13	-0.18 – -0.07
Injunctive inclusivity norms x Identification difference score	-0.02	-0.07 – 0.02
Descriptive inclusivity norms x Identification difference score	0.02	-0.02 – 0.06
Observations	1002	
R^2 Bayes	0.089	
BF_{01}	220.40	

Table S42.
Bayesian moderated multiple regression results (Finnish sample)

	Tolerance	
Predictors	*Estimates*	*CI (95%)*
Injunctive inclusivity norms	0.08	0.01 – 0.17
Identification difference score	-0.04	-0.19 – 0.11
Descriptive inclusivity norms	0.08	-0.00 – 0.15
Topic importance	0.05	0.00 – 0.09
Opinion extremity	-0.00	-0.09 – 0.08
Strength of disapproval	-0.31	-0.36 – -0.25
Injunctive inclusivity norms x Identification difference score	-0.02	-0.06 – 0.01
Descriptive inclusivity norms x Identification difference score	0.01	-0.02 – 0.05
Observations	1001	
R^2 Bayes	0.232	
BF_{01}	258.75	

	Avoidance tendencies	
Predictors	*Estimates*	*CI (95%)*
Injunctive inclusivity norms	-0.14	-0.25 – -0.04
Identification difference score	0.18	-0.01 – 0.36
Descriptive inclusivity norms	0.01	-0.09 – 0.11
Topic importance	-0.06	-0.12 – -0.01
Opinion extremity	-0.02	-0.13 – 0.09
Strength of disapproval	0.38	0.32 – 0.45
Injunctive inclusivity norms x Identification difference score	-0.01	-0.05 – 0.03
Descriptive inclusivity norms x Identification difference score	-0.01	-0.06 – 0.04
Observations	1001	
R^2 Bayes	0.206	
BF_{01}	270.57	

	Cooperation willingness	
Predictors	*Estimates*	*CI (95%)*
Injunctive inclusivity norms	0.16	0.06 – 0.25
Identification difference score	0.11	-0.06 – 0.29
Descriptive inclusivity norms	0.12	0.03 – 0.22

Topic importance	0.17	0.12 – 0.23
Opinion extremity	0.05	-0.05 – 0.15
Strength of disapproval	-0.20	-0.26 – -0.14
Injunctive inclusivity norms x Identification difference score	-0.02	-0.06 – 0.02
Descriptive inclusivity norms x Identification difference score	-0.01	-0.06 – 0.03
Observations	1001	
R^2 Bayes	0.123	
BF_{01}	78.95	

Table S43.
Bayesian moderated multiple regression results (French sample)

	Tolerance	
Predictors	*Estimates*	*CI (95%)*
Injunctive inclusivity norms	0.16	0.09 – 0.23
Identification difference score	0.01	-0.11 – 0.12
Descriptive inclusivity norms	0.01	-0.06 – 0.07
Topic importance	0.07	0.01 – 0.13
Opinion extremity	0.03	-0.04 – 0.10
Strength of disapproval	-0.12	-0.17 – -0.08
Injunctive inclusivity norms x Identification difference score	-0.01	-0.03 – 0.02
Descriptive inclusivity norms x Identification difference score	-0.01	-0.04 – 0.01
Observations	1005	
R^2 Bayes	0.092	
BF_{01}	405.58	

	Avoidance tendencies	
Predictors	*Estimates*	*CI (95%)*
Injunctive inclusivity norms	-0.02	-0.11 – 0.07
Identification difference score	0.13	-0.03 – 0.29
Descriptive inclusivity norms	0.12	0.03 – 0.21
Topic importance	-0.06	-0.14 – 0.02
Opinion extremity	0.08	-0.02 – 0.17
Strength of disapproval	0.17	0.11 – 0.23
Injunctive inclusivity norms x Identification difference score	0.00	-0.03 – 0.04
Descriptive inclusivity norms x Identification difference score	0.01	-0.03 – 0.04
Observations	1005	
R^2 Bayes	0.096	
BF_{01}	544.63	

	Cooperation willingness	
Predictors	*Estimates*	*CI (95%)*
Injunctive inclusivity norms	0.19	0.11 – 0.27
Identification difference score	0.08	-0.05 – 0.21
Descriptive inclusivity norms	0.04	-0.03 – 0.11

Topic importance	0.12	0.04 – 0.19
Opinion extremity	0.07	-0.02 – 0.15
Strength of disapproval	-0.07	-0.12 – -0.02
Injunctive inclusivity norms x Identification difference score	-0.02	-0.05 – 0.01
Descriptive inclusivity norms x Identification difference score	0.01	-0.02 – 0.04
Observations	1005	
R^2 Bayes	0.074	
BF_{01}	257.55	

Table S44.
Bayesian moderated multiple regression results (German sample)

	**Tolerance**	
Predictors	*Estimates*	*CI (95%)*
Injunctive inclusivity norms	0.18	0.10 – 0.26
Identification difference score	-0.03	-0.17 – 0.11
Descriptive inclusivity norms	0.06	-0.01 – 0.14
Topic importance	0.06	0.01 – 0.11
Opinion extremity	0.05	-0.03 – 0.12
Strength of disapproval	-0.14	-0.19 – -0.08
Injunctive inclusivity norms x Identification difference score	0.01	-0.03 – 0.04
Descriptive inclusivity norms x Identification difference score	-0.02	-0.05 – 0.02
Observations	1009	
R^2 Bayes	0.108	
BF_{01}	389.75	
	**Avoidance tendencies**	
Predictors	*Estimates*	*CI (95%)*
Injunctive inclusivity norms	-0.11	-0.22 – -0.00
Identification difference score	0.11	-0.08 – 0.31
Descriptive inclusivity norms	0.17	0.06 – 0.27
Topic importance	-0.03	-0.10 – 0.03
Opinion extremity	-0.01	-0.11 – 0.09
Strength of disapproval	0.36	0.29 – 0.43
Injunctive inclusivity norms x Identification difference score	0.01	-0.04 – 0.06
Descriptive inclusivity norms x Identification difference score	-0.01	-0.06 – 0.04
Observations	1009	
R^2 Bayes	0.154	
BF_{01}	299.84	
	**Cooperation willingness**	
Predictors	*Estimates*	*CI (95%)*
Injunctive inclusivity norms	0.22	0.13 – 0.32
Identification difference score	-0.11	-0.26 – 0.04
Descriptive inclusivity norms	0.04	-0.04 – 0.12

Topic importance	0.20	0.15 – 0.27
Opinion extremity	0.00	-0.08 – 0.08
Strength of disapproval	-0.09	-0.15 – -0.04
Injunctive inclusivity norms x Identification difference score	0.04	-0.00 – 0.08
Descriptive inclusivity norms x Identification difference score	-0.03	-0.07 – 0.01
Observations	1009	
R^2 Bayes	0.130	
BF_{01}	80.04	

Table S45.
Bayesian moderated multiple regression results (Greek sample)

	Tolerance	
Predictors	*Estimates*	*CI (95%)*
Injunctive inclusivity norms	0.09	-0.00 – 0.18
Identification difference score	0.02	-0.12 – 0.15
Descriptive inclusivity norms	0.12	0.03 – 0.22
Topic importance	0.05	-0.00 – 0.11
Opinion extremity	-0.02	-0.10 – 0.06
Strength of disapproval	-0.14	-0.19 – -0.09
Injunctive inclusivity norms x Identification difference score	-0.01	-0.04 – 0.02
Descriptive inclusivity norms x Identification difference score	-0.00	-0.04 – 0.03
Observations	1009	
R^2 Bayes	0.083	
BF_{01}	404.35	

	Avoidance tendencies	
Predictors	*Estimates*	*CI (95%)*
Injunctive inclusivity norms	-0.06	-0.18 – 0.06
Identification difference score	0.13	-0.05 – 0.32
Descriptive inclusivity norms	0.07	-0.05 – 0.19
Topic importance	-0.05	-0.12 – 0.03
Opinion extremity	0.03	-0.07 – 0.14
Strength of disapproval	0.28	0.21 – 0.34
Injunctive inclusivity norms x Identification difference score	0.01	-0.04 – 0.05
Descriptive inclusivity norms x Identification difference score	-0.02	-0.07 – 0.02
Observations	1009	
R^2 Bayes	0.107	
BF_{01}	261.26	

	Cooperation willingness	
Predictors	*Estimates*	*CI (95%)*
Injunctive inclusivity norms	0.17	0.06 – 0.28
Identification difference score	0.10	-0.06 – 0.25
Descriptive inclusivity norms	0.09	-0.02 – 0.20

Topic importance	0.13	0.07 – 0.20
Opinion extremity	-0.05	-0.14 – 0.04
Strength of disapproval	-0.15	-0.21 – -0.09
Injunctive inclusivity norms x Identification difference score	-0.04	-0.08 – -0.01
Descriptive inclusivity norms x Identification difference score	0.02	-0.01 – 0.06
Observations	1009	
R^2 Bayes	0.087	
BF_{01}	30.58	

Table S46.
Bayesian moderated multiple regression results (Hungarian sample)

	**Tolerance**	
Predictors	*Estimates*	*CI (95%)*
Injunctive inclusivity norms	0.16	0.07 – 0.25
Identification difference score	0.11	0.01 – 0.22
Descriptive inclusivity norms	-0.02	-0.11 – 0.06
Topic importance	0.04	-0.01 – 0.08
Opinion extremity	0.04	-0.03 – 0.12
Strength of disapproval	-0.14	-0.19 – -0.10
Injunctive inclusivity norms x Identification difference score	-0.05	-0.08 – -0.02
Descriptive inclusivity norms x Identification difference score	0.03	-0.00 – 0.06
Observations	1005	
R^2 Bayes	0.060	
BF_{01}	13.92	
	**Avoidance tendencies**	
Predictors	*Estimates*	*CI (95%)*
Injunctive inclusivity norms	-0.08	-0.21 – 0.03
Identification difference score	0.16	0.01 – 0.31
Descriptive inclusivity norms	0.17	0.06 – 0.28
Topic importance	0.01	-0.04 – 0.07
Opinion extremity	-0.01	-0.12 – 0.09
Strength of disapproval	0.20	0.14 – 0.27
Injunctive inclusivity norms x Identification difference score	0.02	-0.02 – 0.07
Descriptive inclusivity norms x Identification difference score	-0.05	-0.09 – -0.00
Observations	1005	
R^2 Bayes	0.087	
BF_{01}	40.18	
	**Cooperation willingness**	
Predictors	*Estimates*	*CI (95%)*
Injunctive inclusivity norms	0.07	-0.04 – 0.18
Identification difference score	0.13	-0.00 – 0.26
Descriptive inclusivity norms	0.08	-0.01 – 0.19

Topic importance	0.10	0.05 – 0.16
Opinion extremity	-0.00	-0.09 – 0.09
Strength of disapproval	-0.10	-0.15 – -0.05
Injunctive inclusivity norms x Identification difference score	-0.03	-0.07 – 0.01
Descriptive inclusivity norms x Identification difference score	0.00	-0.03 – 0.04
Observations	1005	
R^2 Bayes	0.045	
BF_{01}	65.33	

Table S47.
Bayesian moderated multiple regression results (Italian sample)

	Tolerance	
Predictors	*Estimates*	*CI (95%)*
Injunctive inclusivity norms	0.15	0.06 – 0.24
Identification difference score	0.11	-0.01 – 0.24
Descriptive inclusivity norms	0.10	0.02 – 0.18
Topic importance	0.02	-0.03 – 0.08
Opinion extremity	-0.02	-0.09 – 0.05
Strength of disapproval	-0.12	-0.17 – -0.07
Injunctive inclusivity norms x Identification difference score	-0.02	-0.05 – 0.01
Descriptive inclusivity norms x Identification difference score	-0.02	-0.05 – 0.01
Observations	1003	
R^2 Bayes	0.093	
BF_{01}	29.73	
	**Avoidance tendencies**	
Predictors	*Estimates*	*CI (95%)*
Injunctive inclusivity norms	-0.15	-0.27 – -0.03
Identification difference score	0.07	-0.11 – 0.25
Descriptive inclusivity norms	0.15	0.03 – 0.27
Topic importance	0.01	-0.07 – 0.08
Opinion extremity	0.02	-0.08 – 0.12
Strength of disapproval	0.25	0.18 – 0.31
Injunctive inclusivity norms x Identification difference score	0.02	-0.02 – 0.06
Descriptive inclusivity norms x Identification difference score	-0.01	-0.05 – 0.03
Observations	1003	
R^2 Bayes	0.100	
BF_{01}	286.77	
	**Cooperation willingness**	
Predictors	*Estimates*	*CI (95%)*
Injunctive inclusivity norms	0.20	0.10 – 0.30
Identification difference score	0.01	-0.13 – 0.15
Descriptive inclusivity norms	0.04	-0.05 – 0.13

Topic importance	0.16	0.10 – 0.23
Opinion extremity	0.03	-0.05 – 0.11
Strength of disapproval	-0.09	-0.14 – -0.03
Injunctive inclusivity norms x Identification difference score	-0.01	-0.05 – 0.02
Descriptive inclusivity norms x Identification difference score	-0.00	-0.04 – 0.03
Observations	1003	
R^2 Bayes	0.104	
BF_{01}	384.02	

Table S48.
Bayesian moderated multiple regression results (Dutch sample)

	**Tolerance**	
Predictors	*Estimates*	*CI (95%)*
Injunctive inclusivity norms	0.09	0.01 – 0.18
Identification difference score	-0.04	-0.19 – 0.12
Descriptive inclusivity norms	0.05	-0.03 – 0.12
Topic importance	0.08	0.04 – 0.11
Opinion extremity	0.03	-0.05 – 0.11
Strength of disapproval	0.03	-0.01 – 0.06
Injunctive inclusivity norms x Identification difference score	0.03	-0.01 – 0.07
Descriptive inclusivity norms x Identification difference score	-0.05	-0.08 – -0.01
Observations	1005	
R^2 Bayes	0.076	
BF_{01}	20.41	
	**Avoidance tendencies**	
Predictors	*Estimates*	*CI (95%)*
Injunctive inclusivity norms	-0.10	-0.22 – 0.03
Identification difference score	0.25	0.02 – 0.49
Descriptive inclusivity norms	0.08	-0.03 – 0.19
Topic importance	-0.08	-0.13 – -0.03
Opinion extremity	0.10	-0.02 – 0.21
Strength of disapproval	0.03	-0.02 – 0.09
Injunctive inclusivity norms x Identification difference score	-0.01	-0.07 – 0.05
Descriptive inclusivity norms x Identification difference score	-0.01	-0.06 – 0.04
Observations	1005	
R^2 Bayes	0.070	
BF_{01}	198.58	
	**Cooperation willingness**	
Predictors	*Estimates*	*CI (95%)*
Injunctive inclusivity norms	0.16	0.06 – 0.27
Identification difference score	0.15	-0.03 – 0.34
Descriptive inclusivity norms	0.06	-0.03 – 0.15

Topic importance	0.18	0.14 – 0.22
Opinion extremity	0.05	-0.04 – 0.14
Strength of disapproval	0.05	0.01 – 0.10
Injunctive inclusivity norms x Identification difference score	-0.02	-0.07 – 0.02
Descriptive inclusivity norms x Identification difference score	-0.02	-0.07 – 0.02
Observations	1005	
R^2 Bayes	0.123	
BF_{01}	22.38	

Table S49.
Bayesian moderated multiple regression results (Polish sample)

	**Tolerance**	
Predictors	*Estimates*	*CI (95%)*
Injunctive inclusivity norms	0.12	0.03 – 0.20
Identification difference score	0.01	-0.15 – 0.16
Descriptive inclusivity norms	0.04	-0.03 – 0.12
Topic importance	-0.03	-0.08 – 0.02
Opinion extremity	-0.01	-0.08 – 0.07
Strength of disapproval	-0.05	-0.10 – -0.01
Injunctive inclusivity norms x Identification difference score	0.01	-0.04 – 0.05
Descriptive inclusivity norms x Identification difference score	-0.02	-0.06 – 0.02
Observations	1003	
R^2 Bayes	0.049	
BF_{01}	273.28	
	**Avoidance tendencies**	
Predictors	*Estimates*	*CI (95%)*
Injunctive inclusivity norms	-0.14	-0.25 – -0.04
Identification difference score	0.23	0.02 – 0.44
Descriptive inclusivity norms	0.19	0.09 – 0.30
Topic importance	0.02	-0.04 – 0.08
Opinion extremity	0.07	-0.04 – 0.17
Strength of disapproval	0.10	0.04 – 0.16
Injunctive inclusivity norms x Identification difference score	-0.00	-0.05 – 0.06
Descriptive inclusivity norms x Identification difference score	-0.03	-0.08 – 0.03
Observations	1003	
R^2 Bayes	0.058	
BF_{01}	116.39	
	**Cooperation willingness**	
Predictors	*Estimates*	*CI (95%)*
Injunctive inclusivity norms	0.19	0.10 – 0.29
Identification difference score	-0.03	-0.20 – 0.13
Descriptive inclusivity norms	0.05	-0.03 – 0.14

Topic importance	0.07	0.01 – 0.12
Opinion extremity	0.04	-0.04 – 0.12
Strength of disapproval	-0.00	-0.05 – 0.05
Injunctive inclusivity norms x Identification difference score	0.01	-0.03 – 0.06
Descriptive inclusivity norms x Identification difference score	-0.02	-0.06 – 0.03
Observations	1003	
R^2 Bayes	0.071	
BF_{01}	326.94	

Table S50.
Bayesian moderated multiple regression results (Spanish sample)

	Tolerance	
Predictors	*Estimates*	*CI (95%)*
Injunctive inclusivity norms	0.13	0.04 – 0.21
Identification difference score	-0.14	-0.27 – 0.00
Descriptive inclusivity norms	0.04	-0.04 – 0.13
Topic importance	0.02	-0.04 – 0.08
Opinion extremity	-0.01	-0.09 – 0.08
Strength of disapproval	-0.13	-0.18 – -0.08
Injunctive inclusivity norms x Identification difference score	0.02	-0.02 – 0.05
Descriptive inclusivity norms x Identification difference score	-0.01	-0.04 – 0.03
Observations	999	
R^2 Bayes	0.106	
BF_{01}	416.49	
	**Avoidance tendencies**	
Predictors	*Estimates*	*CI (95%)*
Injunctive inclusivity norms	-0.11	-0.21 – 0.01
Identification difference score	0.37	0.21 – 0.54
Descriptive inclusivity norms	0.23	0.12 – 0.33
Topic importance	0.00	-0.07 – 0.08
Opinion extremity	-0.04	-0.15 – 0.07
Strength of disapproval	0.20	0.14 – 0.26
Injunctive inclusivity norms x Identification difference score	-0.01	-0.05 – 0.04
Descriptive inclusivity norms x Identification difference score	-0.05	-0.10 – -0.00
Observations	999	
R^2 Bayes	0.114	
BF_{01}	2.42	
	**Cooperation willingness**	
Predictors	*Estimates*	*CI (95%)*
Injunctive inclusivity norms	0.08	-0.02 – 0.17
Identification difference score	-0.13	-0.27 – 0.01
Descriptive inclusivity norms	0.09	-0.01 – 0.17

Topic importance	0.16	0.10 – 0.22
Opinion extremity	0.10	0.01 – 0.19
Strength of disapproval	-0.08	-0.13 – -0.03
Injunctive inclusivity norms x Identification difference score	0.02	-0.02 – 0.06
Descriptive inclusivity norms x Identification difference score	-0.01	-0.05 – 0.03
Observations	999	
R^2 Bayes	0.091	
BF_{01}	334.95	

Table S51.
Bayesian moderated multiple regression results (Swedish sample)

	Tolerance	
Predictors	*Estimates*	*CI (95%)*
Injunctive inclusivity norms	0.17	0.09 – 0.25
Identification difference score	-0.10	-0.25 – 0.03
Descriptive inclusivity norms	0.02	-0.05 – 0.10
Topic importance	-0.04	-0.10 – 0.01
Opinion extremity	0.08	0.01 – 0.16
Strength of disapproval	-0.05	-0.10 – -0.00
Injunctive inclusivity norms x Identification difference score	-0.01	-0.05 – 0.03
Descriptive inclusivity norms x Identification difference score	0.02	-0.02 – 0.05
Observations	998	
R^2 Bayes	0.075	
BF_{01}	404.07	
	**Avoidance tendencies**	
Predictors	*Estimates*	*CI (95%)*
Injunctive inclusivity norms	-0.13	-0.23 – -0.03
Identification difference score	0.07	-0.10 – 0.24
Descriptive inclusivity norms	0.02	-0.07 – 0.11
Topic importance	0.03	-0.04 – 0.10
Opinion extremity	-0.00	-0.10 – 0.09
Strength of disapproval	0.21	0.15 – 0.27
Injunctive inclusivity norms x Identification difference score	0.05	0.00 – 0.10
Descriptive inclusivity norms x Identification difference score	-0.05	-0.09 – -0.01
Observations	998	
R^2 Bayes	0.107	
BF_{01}	20.00	
	**Cooperation willingness**	
Predictors	*Estimates*	*CI (95%)*
Injunctive inclusivity norms	0.17	0.08 – 0.26
Identification difference score	0.03	-0.12 – 0.18
Descriptive inclusivity norms	0.06	-0.02 – 0.13

Topic importance	0.08	0.01 – 0.14
Opinion extremity	0.09	0.01 – 0.18
Strength of disapproval	-0.08	-0.13 – -0.02
Injunctive inclusivity norms x Identification difference score	-0.00	-0.04 – 0.04
Descriptive inclusivity norms x Identification difference score	-0.02	-0.06 – 0.01
Observations	998	
R^2 Bayes	0.072	
BF_{01}	130.64	

Table S52.
Bayesian moderated multiple regression results (British sample)

	**Tolerance**	
Predictors	*Estimates*	*CI (95%)*
Injunctive inclusivity norms	0.04	-0.04 – 0.13
Identification difference score	-0.13	-0.25 – -0.01
Descriptive inclusivity norms	0.06	-0.01 – 0.13
Topic importance	0.01	-0.04 – 0.06
Opinion extremity	-0.01	-0.09 – 0.07
Strength of disapproval	-0.08	-0.13 – -0.03
Injunctive inclusivity norms x Identification difference score	0.00	-0.03 – 0.04
Descriptive inclusivity norms x Identification difference score	0.01	-0.02 – 0.05
Observations	1002	
R^2 Bayes	0.060	
BF_{01}	448.64	
	**Avoidance tendencies**	
Predictors	*Estimates*	*CI (95%)*
Injunctive inclusivity norms	-0.03	-0.14 – 0.08
Identification difference score	0.22	0.06 – 0.38
Descriptive inclusivity norms	-0.04	-0.14 – 0.07
Topic importance	-0.01	-0.08 – 0.07
Opinion extremity	0.08	-0.03 – 0.19
Strength of disapproval	0.23	0.15 – 0.30
Injunctive inclusivity norms x Identification difference score	0.01	-0.04 – 0.06
Descriptive inclusivity norms x Identification difference score	-0.03	-0.08 – 0.01
Observations	1002	
R^2 Bayes	0.120	
BF_{01}	88.97	
	**Cooperation willingness**	
Predictors	*Estimates*	*CI (95%)*
Injunctive inclusivity norms	0.12	0.02 – 0.21
Identification difference score	0.06	-0.08 – 0.19
Descriptive inclusivity norms	0.11	0.03 – 0.20

Topic importance	0.12	0.06 – 0.18
Opinion extremity	0.01	-0.08 – 0.10
Strength of disapproval	-0.05	-0.11 – 0.01
Injunctive inclusivity norms x Identification difference score	-0.04	-0.08 – -0.00
Descriptive inclusivity norms x Identification difference score	0.02	-0.02 – 0.06
Observations	1002	
R^2 Bayes	0.074	
BF_{01}	54.18	

Social Dominance Orientation

Table S53.
Bayesian moderated multiple regression results (Czech sample)

	Tolerance	
Predictors	*Estimates*	*CI (95%)*
Injunctive inclusivity norms	-0.09	-0.31 – 0.12
SDO	-0.20	-0.42 – 0.00
Descriptive inclusivity norms	0.16	-0.07 – 0.38
Topic importance	0.05	0.00 – 0.10
Opinion extremity	0.03	-0.04 – 0.10
Strength of disapproval	-0.20	-0.25 – -0.15
Injunctive inclusivity norms x SDO	0.05	-0.01 – 0.10
Descriptive inclusivity norms x SDO	-0.01	-0.06 – 0.04
Observations	1002	
R2 Bayes	0.113	
BF_{01}	42.26	
	**Avoidance tendencies**	
Predictors	*Estimates*	*CI (95%)*
Injunctive inclusivity norms	-0.00	-0.29 – 0.27
SDO	0.01	-0.26 – 0.28
Descriptive inclusivity norms	-0.18	-0.46 – 0.11
Topic importance	-0.02	-0.08 – 0.04
Opinion extremity	0.02	-0.07 – 0.11
Strength of disapproval	0.30	0.24 – 0.36
Injunctive inclusivity norms x SDO	-0.01	-0.08 – 0.06
Descriptive inclusivity norms x SDO	0.04	-0.03 – 0.11
Observations	1002	
R^2 Bayes	0.138	
BF_{01}	53.27	
	**Cooperation willingness**	
Predictors	*Estimates*	*CI (95%)*
Injunctive inclusivity norms	0.08	-0.17 – 0.33
SDO	-0.01	-0.25 – 0.23
Descriptive inclusivity norms	0.24	-0.01 – 0.50

Topic importance	0.09	0.04 – 0.15
Opinion extremity	0.01	-0.07 – 0.09
Strength of disapproval	-0.14	-0.19 – -0.08
Injunctive inclusivity norms x SDO	0.01	-0.05 – 0.08
Descriptive inclusivity norms x SDO	-0.03	-0.09 – 0.03
Observations	1002	
R^2 Bayes	0.086	
BF_{01}	132.29	

Table S54.
Bayesian moderated multiple regression results (Finnish sample)

	Tolerance	
Predictors	*Estimates*	*CI (95%)*
Injunctive inclusivity norms	-0.01	-0.19 – 0.17
SDO	-0.17	-0.40 – 0.05
Descriptive inclusivity norms	0.15	-0.03 – 0.33
Topic importance	0.02	-0.02 – 0.07
Opinion extremity	-0.05	-0.13 – 0.04
Strength of disapproval	-0.33	-0.38 – -0.28
Injunctive inclusivity norms x SDO	0.03	-0.02 – 0.08
Descriptive inclusivity norms x SDO	-0.01	-0.06 – 0.04
Observations	1001	
R2 Bayes	0.223	
BF_{01}	138.75	

	Avoidance tendencies	
Predictors	*Estimates*	*CI (95%)*
Injunctive inclusivity norms	-0.20	-0.44 – 0.03
SDO	-0.06	-0.35 – 0.23
Descriptive inclusivity norms	-0.12	-0.35 – 0.13
Topic importance	-0.04	-0.09 – 0.02
Opinion extremity	0.03	-0.08 – 0.14
Strength of disapproval	0.41	0.34 – 0.47
Injunctive inclusivity norms x SDO	0.02	-0.05 – 0.09
Descriptive inclusivity norms x SDO	0.02	-0.05 – 0.09
Observations	1001	
R^2 Bayes	0.205	
BF_{01}	67.83	

	Cooperation willingness	
Predictors	*Estimates*	*CI (95%)*
Injunctive inclusivity norms	-0.04	-0.27 – 0.17
SDO	-0.58	-0.85 – -0.31
Descriptive inclusivity norms	0.03	-0.19 – 0.25
Topic importance	0.15	0.09 – 0.20
Opinion extremity	0.02	-0.08 – 0.12

Strength of disapproval	-0.21	-0.27 – -0.16
Injunctive inclusivity norms x SDO	0.06	-0.00 – 0.13
Descriptive inclusivity norms x SDO	0.04	-0.03 – 0.10
Observations	1001	
R^2 Bayes	0.139	
BF_{01}	0.40	

Table S55.
Bayesian moderated multiple regression results (French sample)

	Tolerance	
Predictors	*Estimates*	*CI (95%)*
Injunctive inclusivity norms	0.14	-0.03 – 0.30
SDO	0.11	-0.06 – 0.29
Descriptive inclusivity norms	0.13	-0.02 – 0.29
Topic importance	0.04	-0.02 – 0.10
Opinion extremity	0.01	-0.06 – 0.09
Strength of disapproval	-0.13	-0.18 – -0.09
Injunctive inclusivity norms x SDO	0.01	-0.03 – 0.05
Descriptive inclusivity norms x SDO	-0.03	-0.07 – 0.01
Observations	1005	
R2 Bayes	0.082	
BF_{01}	84.12	
	**Avoidance tendencies**	
Predictors	*Estimates*	*CI (95%)*
Injunctive inclusivity norms	-0.20	-0.43 – 0.05
SDO	-0.05	-0.28 – 0.19
Descriptive inclusivity norms	0.00	-0.22 – 0.22
Topic importance	0.01	-0.07 – 0.09
Opinion extremity	0.14	0.04 – 0.23
Strength of disapproval	0.19	0.12 – 0.25
Injunctive inclusivity norms x SDO	0.05	-0.01 – 0.11
Descriptive inclusivity norms x SDO	0.01	-0.05 – 0.07
Observations	1005	
R^2 Bayes	0.103	
BF_{01}	14.46	
	**Cooperation willingness**	
Predictors	*Estimates*	*CI (95%)*
Injunctive inclusivity norms	0.20	0.00 – 0.39
SDO	-0.08	-0.29 – 0.11
Descriptive inclusivity norms	0.03	-0.15 – 0.21
Topic importance	0.11	0.05 – 0.18
Opinion extremity	0.05	-0.03 – 0.14

Strength of disapproval	-0.07	-0.12 – -0.02
Injunctive inclusivity norms x SDO	-0.01	-0.06 – 0.04
Descriptive inclusivity norms x SDO	0.01	-0.04 – 0.06
Observations	1005	
R^2 Bayes	0.080	
BF_{01}	295.31	

Table S56.
Bayesian moderated multiple regression results (German sample)

	Tolerance	
Predictors	*Estimates*	*CI (95%)*
Injunctive inclusivity norms	0.09	-0.13 – 0.30
SDO	-0.21	-0.43 – -0.02
Descriptive inclusivity norms	0.04	-0.16 – 0.22
Topic importance	0.05	-0.00 – 0.10
Opinion extremity	0.03	-0.05 – 0.10
Strength of disapproval	-0.15	-0.20 – -0.10
Injunctive inclusivity norms x SDO	0.03	-0.03 – 0.08
Descriptive inclusivity norms x SDO	0.01	-0.04 – 0.06
Observations	1009	
R2 Bayes	0.106	
BF_{01}	73.84	

	Avoidance tendencies	
Predictors	*Estimates*	*CI (95%)*
Injunctive inclusivity norms	-0.21	-0.49 – 0.06
SDO	-0.22	-0.47 – 0.04
Descriptive inclusivity norms	-0.23	-0.48 – 0.02
Topic importance	-0.00	-0.07 – 0.06
Opinion extremity	0.04	-0.05 – 0.14
Strength of disapproval	0.35	0.28 – 0.42
Injunctive inclusivity norms x SDO	0.02	-0.05 – 0.09
Descriptive inclusivity norms x SDO	0.08	0.02 – 0.14
Observations	1009	
R^2 Bayes	0.176	
BF_{01}	0.09	

	Cooperation willingness	
Predictors	*Estimates*	*CI (95%)*
Injunctive inclusivity norms	0.24	0.01 – 0.48
SDO	-0.29	-0.52 – -0.08
Descriptive inclusivity norms	-0.16	-0.37 – 0.06
Topic importance	0.19	0.13 – 0.25
Opinion extremity	-0.02	-0.10 – 0.07

Strength of disapproval	-0.10	-0.16 – -0.04
Injunctive inclusivity norms x SDO	0.00	-0.06 – 0.06
Descriptive inclusivity norms x SDO	0.05	-0.00 – 0.10
Observations	1009	
R^2 Bayes	0.134	
BF_{01}	9.51	

Table S57.
Bayesian moderated multiple regression results (Greek sample)

	Tolerance	
Predictors	*Estimates*	*CI (95%)*
Injunctive inclusivity norms	-0.07	-0.26 – 0.12
SDO	-0.31	-0.49 – -0.13
Descriptive inclusivity norms	0.16	-0.04 – 0.34
Topic importance	0.04	-0.02 – 0.09
Opinion extremity	-0.04	-0.12 – 0.04
Strength of disapproval	-0.14	-0.19 – -0.09
Injunctive inclusivity norms x SDO	0.05	-0.00 – 0.11
Descriptive inclusivity norms x SDO	-0.00	-0.06 – 0.05
Observations	1009	
R2 Bayes	0.094	
BF_{01}	11.78	
	**Avoidance tendencies**	
Predictors	*Estimates*	*CI (95%)*
Injunctive inclusivity norms	0.10	-0.14 – 0.35
SDO	0.00	-0.23 – 0.23
Descriptive inclusivity norms	-0.39	-0.63 – -0.15
Topic importance	-0.02	-0.09 – 0.05
Opinion extremity	0.08	-0.02 – 0.19
Strength of disapproval	0.27	0.21 – 0.33
Injunctive inclusivity norms x SDO	-0.05	-0.12 – 0.01
Descriptive inclusivity norms x SDO	0.11	0.04 – 0.18
Observations	1009	
R^2 Bayes	0.132	
BF_{01}	0.53	
	**Cooperation willingness**	
Predictors	*Estimates*	*CI (95%)*
Injunctive inclusivity norms	-0.20	-0.39 – 0.01
SDO	-0.54	-0.74 – -0.34
Descriptive inclusivity norms	0.14	-0.06 – 0.35
Topic importance	0.12	0.06 – 0.18
Opinion extremity	-0.04	-0.13 – 0.04

Strength of disapproval	-0.15	-0.21 – -0.10
Injunctive inclusivity norms x SDO	0.10	0.04 – 0.15
Descriptive inclusivity norms x SDO	-0.00	-0.06 – 0.06
Observations	1009	
R^2 Bayes	0.109	
BF_{01}	0.01	

Table S58.
Bayesian moderated multiple regression results (Hungarian sample)

	**Tolerance**	
Predictors	*Estimates*	*CI (95%)*
Injunctive inclusivity norms	-0.12	-0.32 – 0.09
SDO	-0.18	-0.34 – -0.03
Descriptive inclusivity norms	0.15	-0.04 – 0.33
Topic importance	0.04	-0.01 – 0.08
Opinion extremity	0.06	-0.02 – 0.13
Strength of disapproval	-0.14	-0.18 – -0.09
Injunctive inclusivity norms x SDO	0.06	0.01 – 0.11
Descriptive inclusivity norms x SDO	-0.04	-0.08 – 0.01
Observations	1005	
R2 Bayes	0.060	
BF_{01}	24.99	
	**Avoidance tendencies**	
Predictors	*Estimates*	*CI (95%)*
Injunctive inclusivity norms	0.08	-0.19 – 0.34
SDO	0.00	-0.20 – 0.23
Descriptive inclusivity norms	-0.26	-0.51 – -0.00
Topic importance	0.03	-0.03 – 0.08
Opinion extremity	0.02	-0.07 – 0.13
Strength of disapproval	0.22	0.16 – 0.28
Injunctive inclusivity norms x SDO	-0.04	-0.11 – 0.03
Descriptive inclusivity norms x SDO	0.09	0.02 – 0.15
Observations	1005	
R^2 Bayes	0.097	
BF_{01}	3.12	
	**Cooperation willingness**	
Predictors	*Estimates*	*CI (95%)*
Injunctive inclusivity norms	-0.30	-0.54 – -0.05
SDO	-0.37	-0.55 – -0.18
Descriptive inclusivity norms	0.27	0.04 – 0.50
Topic importance	0.10	0.05 – 0.15
Opinion extremity	0.00	-0.09 – 0.09

Strength of disapproval	-0.09	-0.14 – -0.04
Injunctive inclusivity norms x SDO	0.09	0.03 – 0.16
Descriptive inclusivity norms x SDO	-0.04	-0.10 – 0.02
Observations	1005	
R^2 Bayes	0.064	
BF_{01}	1.86	

Table S59.
Bayesian moderated multiple regression results (Italian sample)

	Tolerance	
Predictors	*Estimates*	*CI (95%)*
Injunctive inclusivity norms	0.06	-0.11 – 0.23
SDO	-0.09	-0.27 – 0.10
Descriptive inclusivity norms	0.06	-0.10 – 0.22
Topic importance	0.02	-0.03 – 0.07
Opinion extremity	-0.04	-0.11 – 0.03
Strength of disapproval	-0.12	-0.16 – -0.07
Injunctive inclusivity norms x SDO	0.02	-0.03 – 0.06
Descriptive inclusivity norms x SDO	0.01	-0.03 – 0.05
Observations	1003	
R2 Bayes	0.085	
BF_{01}	203.93	
	**Avoidance tendencies**	
Predictors	*Estimates*	*CI (95%)*
Injunctive inclusivity norms	-0.04	-0.29 – 0.19
SDO	-0.05	-0.30 – 0.20
Descriptive inclusivity norms	-0.23	-0.47 – -0.02
Topic importance	0.01	-0.06 – 0.09
Opinion extremity	0.08	-0.02 – 0.18
Strength of disapproval	0.26	0.20 – 0.32
Injunctive inclusivity norms x SDO	-0.02	-0.08 – 0.04
Descriptive inclusivity norms x SDO	0.08	0.02 – 0.13
Observations	1003	
R^2 Bayes	0.106	
BF_{01}	3.37	
	**Cooperation willingness**	
Predictors	*Estimates*	*CI (95%)*
Injunctive inclusivity norms	0.11	-0.07 – 0.30
SDO	-0.12	-0.33 – 0.09
Descriptive inclusivity norms	0.07	-0.11 – 0.25
Topic importance	0.16	0.10 – 0.22
Opinion extremity	0.00	-0.07 – 0.08

Strength of disapproval	-0.09	-0.15 – -0.04
Injunctive inclusivity norms x SDO	0.02	-0.03 – 0.07
Descriptive inclusivity norms x SDO	0.00	-0.04 – 0.05
Observations	1003	
R^2 Bayes	0.098	
BF_{01}	212.94	

Table S60.
Bayesian moderated multiple regression results (Dutch sample)

	Tolerance	
Predictors	*Estimates*	*CI (95%)*
Injunctive inclusivity norms	-0.07	-0.30 – 0.16
SDO	-0.42	-0.65 – -0.22
Descriptive inclusivity norms	-0.06	-0.27 – 0.15
Topic importance	0.06	0.03 – 0.09
Opinion extremity	-0.05	-0.12 – 0.03
Strength of disapproval	0.02	-0.02 – 0.06
Injunctive inclusivity norms x SDO	0.05	-0.01 – 0.11
Descriptive inclusivity norms x SDO	0.03	-0.02 – 0.08
Observations	1005	
R2 Bayes	0.063	
BF_{01}	0.56	

	Avoidance tendencies	
Predictors	*Estimates*	*CI (95%)*
Injunctive inclusivity norms	0.12	-0.21 – 0.45
SDO	0.17	-0.16 – 0.49
Descriptive inclusivity norms	-0.24	-0.56 – 0.06
Topic importance	-0.05	-0.10 – -0.01
Opinion extremity	0.22	0.11 – 0.33
Strength of disapproval	0.02	-0.04 – 0.07
Injunctive inclusivity norms x SDO	-0.07	-0.15 – 0.01
Descriptive inclusivity norms x SDO	0.07	0.00 – 0.15
Observations	1005	
R^2 Bayes	0.050	
BF_{01}	15.59	

	Cooperation willingness	
Predictors	*Estimates*	*CI (95%)*
Injunctive inclusivity norms	-0.05	-0.32 – 0.21
SDO	-0.53	-0.80 – -0.28
Descriptive inclusivity norms	-0.03	-0.27 – 0.22
Topic importance	0.17	0.13 – 0.20
Opinion extremity	0.01	-0.08 – 0.09

Strength of disapproval	0.05	0.01 – 0.10
Injunctive inclusivity norms x SDO	0.06	-0.01 – 0.13
Descriptive inclusivity norms x SDO	0.03	-0.03 – 0.09
Observations	1005	
R^2 Bayes	0.134	
BF_{01}	0.98	

Table S61.
Bayesian moderated multiple regression results (Polish sample)

	**Tolerance**	
Predictors	*Estimates*	*CI (95%)*
Injunctive inclusivity norms	0.10	-0.11 – 0.31
SDO	-0.11	-0.32 – 0.10
Descriptive inclusivity norms	0.04	-0.16 – 0.25
Topic importance	-0.04	-0.09 – 0.01
Opinion extremity	-0.03	-0.11 – 0.04
Strength of disapproval	-0.06	-0.10 – -0.01
Injunctive inclusivity norms x SDO	0.01	-0.04 – 0.06
Descriptive inclusivity norms x SDO	0.00	-0.05 – 0.05
Observations	1003	
R^2 Bayes	0.048	
BF_{01}	277.84	
	**Avoidance tendencies**	
Predictors	*Estimates*	*CI (95%)*
Injunctive inclusivity norms	-0.08	-0.37 – 0.20
SDO	0.15	-0.13 – 0.43
Descriptive inclusivity norms	-0.06	-0.33 – 0.21
Topic importance	0.04	-0.02 – 0.11
Opinion extremity	0.13	0.03 – 0.23
Strength of disapproval	0.10	0.04 – 0.17
Injunctive inclusivity norms x SDO	-0.02	-0.09 – 0.05
Descriptive inclusivity norms x SDO	0.05	-0.02 – 0.11
Observations	1003	
R^2 Bayes	0.076	
BF_{01}	55.84	
	**Cooperation willingness**	
Predictors	*Estimates*	*CI (95%)*
Injunctive inclusivity norms	0.15	-0.09 – 0.37
SDO	-0.38	-0.61 – -0.16
Descriptive inclusivity norms	-0.06	-0.28 – 0.15
Topic importance	0.05	0.00 – 0.10
Opinion extremity	0.01	-0.06 – 0.09

Strength of disapproval	-0.00	-0.05 – 0.05
Injunctive inclusivity norms x SDO	0.02	-0.04 – 0.07
Descriptive inclusivity norms x SDO	0.03	-0.02 – 0.09
Observations	1003	
R^2 Bayes	0.093	
BF_{01}	18.42	

Table S62.
Bayesian moderated multiple regression results (Spanish sample)

	Tolerance	
Predictors	*Estimates*	*CI (95%)*
Injunctive inclusivity norms	0.23	0.05 – 0.41
SDO	-0.10	-0.28 – 0.07
Descriptive inclusivity norms	-0.14	-0.32 – 0.04
Topic importance	-0.01	-0.06 – 0.05
Opinion extremity	-0.05	-0.13 – 0.04
Strength of disapproval	-0.15	-0.19 – -0.10
Injunctive inclusivity norms x SDO	-0.02	-0.07 – 0.03
Descriptive inclusivity norms x SDO	0.06	0.01 – 0.10
Observations	999	
R2 Bayes	0.097	
BF_{01}	18.54	

	Avoidance tendencies	
Predictors	*Estimates*	*CI (95%)*
Injunctive inclusivity norms	0.01	-0.22 – 0.24
SDO	-0.08	-0.31 – 0.14
Descriptive inclusivity norms	-0.20	-0.46 – 0.04
Topic importance	0.06	-0.00 – 0.13
Opinion extremity	0.03	-0.08 – 0.13
Strength of disapproval	0.22	0.16 – 0.28
Injunctive inclusivity norms x SDO	-0.05	-0.12 – 0.01
Descriptive inclusivity norms x SDO	0.10	0.04 – 0.17
Observations	999	
R^2 Bayes	0.100	
BF_{01}	0.90	

	Cooperation willingness	
Predictors	*Estimates*	*CI (95%)*
Injunctive inclusivity norms	-0.04	-0.23 – 0.15
SDO	-0.23	-0.41 – -0.06
Descriptive inclusivity norms	0.11	-0.09 – 0.30
Topic importance	0.13	0.07 – 0.19
Opinion extremity	0.07	-0.02 – 0.15

Strength of disapproval	-0.09	-0.14 – -0.05
Injunctive inclusivity norms x SDO	0.05	-0.01 – 0.10
Descriptive inclusivity norms x SDO	-0.00	-0.06 – 0.05
Observations	999	
R^2 Bayes	0.086	
BF_{01}	19.68	

Table S63.
Bayesian moderated multiple regression results (Swedish sample)

	**Tolerance**	
Predictors	*Estimates*	*CI (95%)*
Injunctive inclusivity norms	0.01	-0.21 – 0.24
SDO	-0.16	-0.37 – 0.04
Descriptive inclusivity norms	0.13	-0.07 – 0.34
Topic importance	-0.06	-0.12 – -0.01
Opinion extremity	0.06	-0.02 – 0.13
Strength of disapproval	-0.07	-0.12 – -0.02
Injunctive inclusivity norms x SDO	0.04	-0.02 – 0.09
Descriptive inclusivity norms x SDO	-0.02	-0.07 – 0.03
Observations	998	
R2 Bayes	0.060	
BF_{01}	117.68	
	**Avoidance tendencies**	
Predictors	*Estimates*	*CI (95%)*
Injunctive inclusivity norms	0.18	-0.11 – 0.46
SDO	0.04	-0.21 – 0.28
Descriptive inclusivity norms	-0.47	-0.71 – -0.22
Topic importance	0.05	-0.02 – 0.12
Opinion extremity	0.03	-0.06 – 0.12
Strength of disapproval	0.23	0.17 – 0.29
Injunctive inclusivity norms x SDO	-0.08	-0.15 – -0.00
Descriptive inclusivity norms x SDO	0.12	0.05 – 0.18
Observations	998	
R^2 Bayes	0.112	
BF_{01}	0.21	
	**Cooperation willingness**	
Predictors	*Estimates*	*CI (95%)*
Injunctive inclusivity norms	0.01	-0.24 – 0.25
SDO	-0.21	-0.42 – 0.01
Descriptive inclusivity norms	0.09	-0.13 – 0.33
Topic importance	0.06	0.00 – 0.12
Opinion extremity	0.08	-0.00 – 0.16

Strength of disapproval	-0.09	-0.14 – -0.03
Injunctive inclusivity norms x SDO	0.04	-0.02 – 0.10
Descriptive inclusivity norms x SDO	-0.01	-0.07 – 0.05
Observations	998	
R^2 Bayes	0.068	
BF_{01}	72.08	

Table S64.
Bayesian moderated multiple regression results (British sample)

	Tolerance	
Predictors	*Estimates*	*CI (95%)*
Injunctive inclusivity norms	0.08	-0.10 – 0.27
SDO	-0.05	-0.23 – 0.14
Descriptive inclusivity norms	0.06	-0.12 – 0.24
Topic importance	-0.02	-0.07 – 0.03
Opinion extremity	-0.02	-0.11 – 0.06
Strength of disapproval	-0.09	-0.14 – -0.04
Injunctive inclusivity norms x SDO	-0.01	-0.06 – 0.04
Descriptive inclusivity norms x SDO	0.01	-0.04 – 0.05
Observations	1002	
R2 Bayes	0.050	
BF_{01}	340.37	
	**Avoidance tendencies**	
Predictors	*Estimates*	*CI (95%)*
Injunctive inclusivity norms	-0.32	-0.58 – -0.07
SDO	-0.39	-0.62 – -0.15
Descriptive inclusivity norms	-0.22	-0.46 – 0.02
Topic importance	0.04	-0.03 – 0.12
Opinion extremity	0.12	0.01 – 0.24
Strength of disapproval	0.23	0.16 – 0.30
Injunctive inclusivity norms x SDO	0.08	0.01 – 0.15
Descriptive inclusivity norms x SDO	0.03	-0.03 – 0.10
Observations	1002	
R^2 Bayes	0.129	
BF_{01}	0.01	
	**Cooperation willingness**	
Predictors	*Estimates*	*CI (95%)*
Injunctive inclusivity norms	0.07	-0.13 – 0.27
SDO	-0.23	-0.43 – -0.03
Descriptive inclusivity norms	0.11	-0.09 – 0.31
Topic importance	0.09	0.03 – 0.15
Opinion extremity	-0.00	-0.09 – 0.09

Strength of disapproval	-0.05	-0.11 – 0.01
Injunctive inclusivity norms x SDO	0.01	-0.05 – 0.07
Descriptive inclusivity norms x SDO	0.01	-0.04 – 0.06
Observations	1002	
R^2 Bayes	0.085	
BF_{01}	184.20	

Right-Wing Authoritarianism

Table S65.
Bayesian moderated multiple regression results (Czech sample)

	**Tolerance**	
Predictors	*Estimates*	*CI (95%)*
Injunctive inclusivity norms	-0.02	-0.33 – 0.30
RWA	-0.11	-0.38 – 0.18
Descriptive inclusivity norms	-0.02	-0.36 – 0.31
Topic importance	0.05	0.00 – 0.09
Opinion extremity	0.02	-0.05 – 0.09
Strength of disapproval	-0.20	-0.24 – -0.15
Injunctive inclusivity norms x RWA	0.02	-0.05 – 0.09
Descriptive inclusivity norms x RWA	0.03	-0.05 – 0.10
Observations	1001	
R2 Bayes	0.119	
BF_{01}	40.46	
	**Avoidance tendencies**	
Predictors	*Estimates*	*CI (95%)*
Injunctive inclusivity norms	0.15	-0.25 – 0.56
RWA	-0.32	-0.67 – 0.02
Descriptive inclusivity norms	-0.62	-1.07 – -0.19
Topic importance	-0.03	-0.09 – 0.02
Opinion extremity	0.02	-0.06 – 0.11
Strength of disapproval	0.31	0.25 – 0.37
Injunctive inclusivity norms x RWA	-0.04	-0.14 – 0.05
Descriptive inclusivity norms x RWA	0.14	0.05 – 0.24
Observations	1001	
R^2 Bayes	0.131	
BF_{01}	0.53	
	**Cooperation willingness**	
Predictors	*Estimates*	*CI (95%)*
Injunctive inclusivity norms	0.23	-0.15 – 0.60
RWA	0.18	-0.15 – 0.50
Descriptive inclusivity norms	0.07	-0.32 – 0.48

Topic importance	0.09	0.04 – 0.15
Opinion extremity	0.01	-0.07 – 0.09
Strength of disapproval	-0.14	-0.19 – -0.08
Injunctive inclusivity norms x RWA	-0.03	-0.11 – 0.06
Descriptive inclusivity norms x RWA	0.01	-0.08 – 0.10
Observations	1001	
R^2 Bayes	0.087	
BF_{01}	91.34	

Table S66.
Bayesian moderated multiple regression results (Finnish sample)

	**Tolerance**	
Predictors	*Estimates*	*CI (95%)*
Injunctive inclusivity norms	-0.12	-0.42 – 0.17
RWA	-0.32	-0.61 – -0.04
Descriptive inclusivity norms	0.03	-0.27 – 0.35
Topic importance	0.03	-0.02 – 0.07
Opinion extremity	-0.03	-0.12 – 0.05
Strength of disapproval	-0.34	-0.39 – -0.29
Injunctive inclusivity norms x RWA	0.05	-0.02 – 0.12
Descriptive inclusivity norms x RWA	0.02	-0.06 – 0.08
Observations	1001	
R2 Bayes	0.220	
BF_{01}	12.64	
	**Avoidance tendencies**	
Predictors	*Estimates*	*CI (95%)*
Injunctive inclusivity norms	-0.32	-0.69 – 0.06
RWA	-0.24	-0.62 – 0.13
Descriptive inclusivity norms	-0.14	-0.54 – 0.25
Topic importance	-0.04	-0.10 – 0.01
Opinion extremity	0.02	-0.08 – 0.13
Strength of disapproval	0.41	0.34 – 0.47
Injunctive inclusivity norms x RWA	0.04	-0.05 – 0.13
Descriptive inclusivity norms x RWA	0.03	-0.06 – 0.12
Observations	1001	
R^2 Bayes	0.199	
BF_{01}	22.48	
	**Cooperation willingness**	
Predictors	*Estimates*	*CI (95%)*
Injunctive inclusivity norms	0.24	-0.09 – 0.58
RWA	-0.33	-0.66 – -0.00
Descriptive inclusivity norms	-0.13	-0.49 – 0.22
Topic importance	0.15	0.10 – 0.21
Opinion extremity	0.03	-0.07 – 0.13

Strength of disapproval	-0.22	-0.28 – -0.16
Injunctive inclusivity norms x RWA	-0.02	-0.09 – 0.06
Descriptive inclusivity norms x RWA	0.07	-0.01 – 0.15
Observations	1001	
R^2 Bayes	0.129	
BF_{01}	24.07	

Table S67.
Bayesian moderated multiple regression results (French sample)

	Tolerance	
Predictors	*Estimates*	*CI (95%)*
Injunctive inclusivity norms	0.37	0.13 – 0.62
RWA	0.09	-0.13 – 0.32
Descriptive inclusivity norms	-0.30	-0.54 – -0.06
Topic importance	0.05	-0.01 – 0.10
Opinion extremity	0.00	-0.07 – 0.07
Strength of disapproval	-0.13	-0.17 – -0.09
Injunctive inclusivity norms x RWA	-0.05	-0.10 – 0.01
Descriptive inclusivity norms x RWA	0.07	0.02 – 0.12
Observations	1005	
R2 Bayes	0.093	
BF_{01}	9.67	

	Avoidance tendencies	
Predictors	*Estimates*	*CI (95%)*
Injunctive inclusivity norms	-0.19	-0.52 – 0.16
RWA	-0.02	-0.33 – 0.30
Descriptive inclusivity norms	0.22	-0.12 – 0.55
Topic importance	-0.01	-0.09 – 0.07
Opinion extremity	0.12	0.03 – 0.22
Strength of disapproval	0.18	0.13 – 0.24
Injunctive inclusivity norms x RWA	0.04	-0.04 – 0.11
Descriptive inclusivity norms x RWA	-0.03	-0.10 – 0.04
Observations	1005	
R^2 Bayes	0.066	
BF_{01}	85.59	

	Cooperation willingness	
Predictors	*Estimates*	*CI (95%)*
Injunctive inclusivity norms	0.14	-0.14 – 0.44
RWA	-0.23	-0.49 – 0.03
Descriptive inclusivity norms	-0.22	-0.51 – 0.06
Topic importance	0.12	0.05 – 0.19
Opinion extremity	0.06	-0.02 – 0.14

Strength of disapproval	-0.08	-0.13 – -0.02
Injunctive inclusivity norms x RWA	0.01	-0.06 – 0.07
Descriptive inclusivity norms x RWA	0.06	-0.00 – 0.12
Observations	1005	
R^2 Bayes	0.078	
BF_{01}	9.52	

Table S68.
Bayesian moderated multiple regression results (German sample)

	Tolerance	
Predictors	*Estimates*	*CI (95%)*
Injunctive inclusivity norms	0.18	-0.10 – 0.48
RWA	-0.12	-0.37 – 0.15
Descriptive inclusivity norms	-0.08	-0.33 – 0.17
Topic importance	0.05	0.00 – 0.10
Opinion extremity	0.03	-0.04 – 0.10
Strength of disapproval	-0.15	-0.20 – -0.10
Injunctive inclusivity norms x RWA	0.00	-0.06 – 0.06
Descriptive inclusivity norms x RWA	0.03	-0.02 – 0.08
Observations	1009	
R2 Bayes	0.102	
BF_{01}	84.27	
	**Avoidance tendencies**	
Predictors	*Estimates*	*CI (95%)*
Injunctive inclusivity norms	-0.15	-0.52 – 0.25
RWA	-0.14	-0.49 – 0.22
Descriptive inclusivity norms	-0.09	-0.42 – 0.26
Topic importance	-0.02	-0.09 – 0.05
Opinion extremity	0.03	-0.07 – 0.13
Strength of disapproval	0.37	0.30 – 0.44
Injunctive inclusivity norms x RWA	0.00	-0.08 – 0.08
Descriptive inclusivity norms x RWA	0.05	-0.03 – 0.12
Observations	1009	
R^2 Bayes	0.141	
BF_{01}	32.68	
	**Cooperation willingness**	
Predictors	*Estimates*	*CI (95%)*
Injunctive inclusivity norms	0.34	0.03 – 0.67
RWA	-0.09	-0.38 – 0.20
Descriptive inclusivity norms	-0.24	-0.52 – 0.05
Topic importance	0.19	0.14 – 0.25
Opinion extremity	-0.02	-0.10 – 0.07

Strength of disapproval	-0.10	-0.16 – -0.05
Injunctive inclusivity norms x RWA	-0.02	-0.09 – 0.05
Descriptive inclusivity norms x RWA	0.06	-0.00 – 0.12
Observations	1009	
R^2 Bayes	0.126	
BF_{01}	30.54	

Table S69.
Bayesian moderated multiple regression results (Greek sample)

	**Tolerance**	
Predictors	*Estimates*	*CI (95%)*
Injunctive inclusivity norms	-0.14	-0.42 – 0.14
RWA	-0.23	-0.45 – -0.00
Descriptive inclusivity norms	0.15	-0.14 – 0.43
Topic importance	0.04	-0.01 – 0.10
Opinion extremity	-0.03	-0.11 – 0.05
Strength of disapproval	-0.14	-0.19 – -0.09
Injunctive inclusivity norms x RWA	0.05	-0.01 – 0.12
Descriptive inclusivity norms x RWA	-0.01	-0.07 – 0.06
Observations	1009	
R2 Bayes	0.082	
BF_{01}	22.61	
	**Avoidance tendencies**	
Predictors	*Estimates*	*CI (95%)*
Injunctive inclusivity norms	-0.05	-0.41 – 0.31
RWA	-0.23	-0.51 – 0.06
Descriptive inclusivity norms	-0.43	-0.80 – -0.03
Topic importance	-0.04	-0.11 – 0.03
Opinion extremity	0.06	-0.04 – 0.17
Strength of disapproval	0.28	0.21 – 0.35
Injunctive inclusivity norms x RWA	-0.00	-0.09 – 0.08
Descriptive inclusivity norms x RWA	0.09	0.00 – 0.18
Observations	1009	
R^2 Bayes	0.109	
BF_{01}	1.37	
	**Cooperation willingness**	
Predictors	*Estimates*	*CI (95%)*
Injunctive inclusivity norms	-0.34	-0.66 – -0.02
RWA	-0.24	-0.49 – 0.02
Descriptive inclusivity norms	0.38	0.04 – 0.71
Topic importance	0.12	0.06 – 0.19
Opinion extremity	-0.05	-0.14 – 0.04

Strength of disapproval	-0.15	-0.21 – -0.10
Injunctive inclusivity norms x RWA	0.11	0.03 – 0.19
Descriptive inclusivity norms x RWA	-0.06	-0.14 – 0.02
Observations	1009	
R^2 Bayes	0.089	
BF_{01}	2.88	

Table S70.
Bayesian moderated multiple regression results (Hungarian sample)

	Tolerance	
Predictors	*Estimates*	*CI (95%)*
Injunctive inclusivity norms	-0.03	-0.30 – 0.22
RWA	-0.25	-0.43 – -0.06
Descriptive inclusivity norms	-0.06	-0.32 – 0.18
Topic importance	0.04	-0.00 – 0.08
Opinion extremity	0.05	-0.02 – 0.13
Strength of disapproval	-0.14	-0.18 – -0.09
Injunctive inclusivity norms x RWA	0.03	-0.03 – 0.09
Descriptive inclusivity norms x RWA	0.02	-0.04 – 0.08
Observations	1005	
R2 Bayes	0.058	
BF_{01}	21.73	
	**Avoidance tendencies**	
Predictors	*Estimates*	*CI (95%)*
Injunctive inclusivity norms	-0.06	-0.40 – 0.28
RWA	0.04	-0.21 – 0.30
Descriptive inclusivity norms	-0.08	-0.39 – 0.26
Topic importance	0.02	-0.04 – 0.08
Opinion extremity	0.02	-0.08 – 0.13
Strength of disapproval	0.22	0.16 – 0.28
Injunctive inclusivity norms x RWA	-0.01	-0.09 – 0.07
Descriptive inclusivity norms x RWA	0.03	-0.05 – 0.10
Observations	1005	
R^2 Bayes	0.082	
BF_{01}	88.20	
	**Cooperation willingness**	
Predictors	*Estimates*	*CI (95%)*
Injunctive inclusivity norms	0.10	-0.22 – 0.41
RWA	-0.16	-0.39 – 0.07
Descriptive inclusivity norms	-0.07	-0.36 – 0.22
Topic importance	0.11	0.06 – 0.16
Opinion extremity	0.01	-0.08 – 0.10

Strength of disapproval	-0.09	-0.15 – -0.04
Injunctive inclusivity norms x RWA	-0.02	-0.09 – 0.06
Descriptive inclusivity norms x RWA	0.04	-0.03 – 0.11
Observations	1005	
R^2 Bayes	0.044	
BF_{01}	71.56	

Table S71.
Bayesian moderated multiple regression results (Italian sample)

	**Tolerance**	
Predictors	*Estimates*	*CI (95%)*
Injunctive inclusivity norms	0.07	-0.20 – 0.32
RWA	-0.13	-0.35 – 0.11
Descriptive inclusivity norms	-0.16	-0.43 – 0.10
Topic importance	0.02	-0.03 – 0.07
Opinion extremity	-0.05	-0.11 – 0.02
Strength of disapproval	-0.12	-0.17 – -0.08
Injunctive inclusivity norms x RWA	0.01	-0.05 – 0.07
Descriptive inclusivity norms x RWA	0.05	-0.01 – 0.10
Observations	1003	
R2 Bayes	0.096	
BF_{01}	9.90	
	**Avoidance tendencies**	
Predictors	*Estimates*	*CI (95%)*
Injunctive inclusivity norms	-0.05	-0.44 – 0.32
RWA	0.07	-0.28 – 0.38
Descriptive inclusivity norms	0.11	-0.25 – 0.52
Topic importance	0.02	-0.06 – 0.10
Opinion extremity	0.07	-0.03 – 0.17
Strength of disapproval	0.26	0.20 – 0.33
Injunctive inclusivity norms x RWA	-0.02	-0.10 – 0.07
Descriptive inclusivity norms x RWA	-0.01	-0.09 – 0.07
Observations	1003	
R^2 Bayes	0.088	
BF_{01}	99.77	
	**Cooperation willingness**	
Predictors	*Estimates*	*CI (95%)*
Injunctive inclusivity norms	0.06	-0.24 – 0.36
RWA	-0.12	-0.38 – 0.14
Descriptive inclusivity norms	-0.08	-0.37 – 0.22
Topic importance	0.16	0.10 – 0.22
Opinion extremity	0.00	-0.08 – 0.08

Strength of disapproval	-0.10	-0.15 – -0.05
Injunctive inclusivity norms x RWA	0.02	-0.04 – 0.09
Descriptive inclusivity norms x RWA	0.03	-0.03 – 0.09
Observations	1003	
R^2 Bayes	0.104	
BF_{01}	41.05	

Table S72.
Bayesian moderated multiple regression results (Dutch sample)

	Tolerance	
Predictors	*Estimates*	*CI (95%)*
Injunctive inclusivity norms	-0.03	-0.36 – 0.32
RWA	-0.52	-0.81 – -0.21
Descriptive inclusivity norms	-0.23	-0.54 – 0.08
Topic importance	0.06	0.03 – 0.09
Opinion extremity	-0.05	-0.13 – 0.02
Strength of disapproval	0.02	-0.01 – 0.06
Injunctive inclusivity norms x RWA	0.03	-0.04 – 0.11
Descriptive inclusivity norms x RWA	0.07	-0.00 – 0.13
Observations	1005	
R2 Bayes	0.061	
BF_{01}	0.60	
	**Avoidance tendencies**	
Predictors	*Estimates*	*CI (95%)*
Injunctive inclusivity norms	0.34	-0.18 – 0.83
RWA	0.15	-0.26 – 0.58
Descriptive inclusivity norms	-0.46	-0.94 – 0.01
Topic importance	-0.06	-0.11 – -0.01
Opinion extremity	0.22	0.11 – 0.33
Strength of disapproval	0.02	-0.03 – 0.08
Injunctive inclusivity norms x RWA	-0.11	-0.22 – 0.00
Descriptive inclusivity norms x RWA	0.12	0.02 – 0.22
Observations	1005	
R^2 Bayes	0.042	
BF_{01}	4.39	
	**Cooperation willingness**	
Predictors	*Estimates*	*CI (95%)*
Injunctive inclusivity norms	-0.33	-0.74 – 0.06
RWA	-0.62	-0.97 – -0.28
Descriptive inclusivity norms	0.08	-0.31 – 0.48
Topic importance	0.17	0.13 – 0.21
Opinion extremity	0.00	-0.09 – 0.10

Strength of disapproval	0.05	0.01 – 0.10
Injunctive inclusivity norms x RWA	0.12	0.02 – 0.21
Descriptive inclusivity norms x RWA	-0.00	-0.09 – 0.08
Observations	1005	
R^2 Bayes	0.125	
BF_{01}	0.55	

Table S73.
Bayesian moderated multiple regression results (Polish sample)

	Tolerance	
Predictors	*Estimates*	*CI (95%)*
Injunctive inclusivity norms	0.21	-0.07 – 0.51
RWA	-0.12	-0.35 – 0.11
Descriptive inclusivity norms	-0.13	-0.43 – 0.14
Topic importance	-0.04	-0.09 – 0.01
Opinion extremity	-0.03	-0.11 – 0.04
Strength of disapproval	-0.06	-0.10 – -0.01
Injunctive inclusivity norms x RWA	-0.02	-0.08 – 0.05
Descriptive inclusivity norms x RWA	0.04	-0.02 – 0.11
Observations	1003	
R2 Bayes	0.047	
BF_{01}	73.96	
	**Avoidance tendencies**	
Predictors	*Estimates*	*CI (95%)*
Injunctive inclusivity norms	-0.21	-0.58 – 0.17
RWA	-0.04	-0.35 – 0.29
Descriptive inclusivity norms	0.06	-0.32 – 0.45
Topic importance	0.03	-0.03 – 0.10
Opinion extremity	0.11	0.02 – 0.21
Strength of disapproval	0.11	0.05 – 0.17
Injunctive inclusivity norms x RWA	0.01	-0.08 – 0.09
Descriptive inclusivity norms x RWA	0.02	-0.06 – 0.10
Observations	1003	
R^2 Bayes	0.045	
BF_{01}	87.23	
	**Cooperation willingness**	
Predictors	*Estimates*	*CI (95%)*
Injunctive inclusivity norms	0.19	-0.11 – 0.50
RWA	-0.17	-0.43 – 0.09
Descriptive inclusivity norms	-0.08	-0.39 – 0.22
Topic importance	0.06	0.01 – 0.11
Opinion extremity	0.02	-0.06 – 0.10

Strength of disapproval	-0.01	-0.06 – 0.04
Injunctive inclusivity norms x RWA	0.01	-0.06 – 0.07
Descriptive inclusivity norms x RWA	0.03	-0.04 – 0.10
Observations	1003	
R^2 Bayes	0.071	
BF_{01}	71.56	

Table S74.
Bayesian moderated multiple regression results (Spanish sample)

	Tolerance	
Predictors	*Estimates*	*CI (95%)*
Injunctive inclusivity norms	0.22	-0.07 – 0.48
RWA	-0.04	-0.28 – 0.20
Descriptive inclusivity norms	-0.25	-0.53 – 0.02
Topic importance	-0.01	-0.07 – 0.05
Opinion extremity	-0.05	-0.13 – 0.04
Strength of disapproval	-0.15	-0.19 – -0.10
Injunctive inclusivity norms x RWA	-0.02	-0.08 – 0.05
Descriptive inclusivity norms x RWA	0.06	0.00 – 0.12
Observations	999	
R2 Bayes	0.109	
BF_{01}	10.91	

	Avoidance tendencies	
Predictors	*Estimates*	*CI (95%)*
Injunctive inclusivity norms	-0.12	-0.48 – 0.21
RWA	-0.18	-0.47 – 0.11
Descriptive inclusivity norms	-0.05	-0.39 – 0.29
Topic importance	0.05	-0.02 – 0.12
Opinion extremity	0.02	-0.08 – 0.13
Strength of disapproval	0.23	0.17 – 0.29
Injunctive inclusivity norms x RWA	-0.01	-0.08 – 0.08
Descriptive inclusivity norms x RWA	0.05	-0.03 – 0.13
Observations	999	
R^2 Bayes	0.087	
BF_{01}	35.97	

	Cooperation willingness	
Predictors	*Estimates*	*CI (95%)*
Injunctive inclusivity norms	0.12	-0.18 – 0.41
RWA	-0.00	-0.26 – 0.24
Descriptive inclusivity norms	-0.06	-0.35 – 0.24
Topic importance	0.14	0.08 – 0.20
Opinion extremity	0.07	-0.02 – 0.15

Strength of disapproval	-0.10	-0.15 – -0.05
Injunctive inclusivity norms x RWA	-0.00	-0.07 – 0.07
Descriptive inclusivity norms x RWA	0.03	-0.04 – 0.10
Observations	999	
R^2 Bayes	0.088	
BF_{01}	101.12	

Table S75.
Bayesian moderated multiple regression results (Swedish sample)

	**Tolerance**	
Predictors	*Estimates*	*CI (95%)*
Injunctive inclusivity norms	0.05	-0.29 – 0.36
RWA	-0.34	-0.62 – -0.08
Descriptive inclusivity norms	-0.18	-0.47 – 0.11
Topic importance	-0.06	-0.12 – -0.00
Opinion extremity	0.06	-0.02 – 0.14
Strength of disapproval	-0.07	-0.12 – -0.03
Injunctive inclusivity norms x RWA	0.02	-0.04 – 0.09
Descriptive inclusivity norms x RWA	0.05	-0.01 – 0.11
Observations	998	
R2 Bayes	0.062	
BF_{01}	4.99	

	**Avoidance tendencies**	
Predictors	*Estimates*	*CI (95%)*
Injunctive inclusivity norms	0.07	-0.33 – 0.46
RWA	0.04	-0.30 – 0.37
Descriptive inclusivity norms	-0.27	-0.64 – 0.09
Topic importance	0.05	-0.02 – 0.12
Opinion extremity	0.02	-0.08 – 0.11
Strength of disapproval	0.23	0.16 – 0.29
Injunctive inclusivity norms x RWA	-0.04	-0.12 – 0.04
Descriptive inclusivity norms x RWA	0.05	-0.02 – 0.13
Observations	998	
R^2 Bayes	0.090	
BF_{01}	49.66	

	**Cooperation willingness**	
Predictors	*Estimates*	*CI (95%)*
Injunctive inclusivity norms	0.18	-0.15 – 0.54
RWA	-0.14	-0.43 – 0.14
Descriptive inclusivity norms	-0.08	-0.41 – 0.24
Topic importance	0.06	0.00 – 0.13
Opinion extremity	0.08	-0.00 – 0.16

Strength of disapproval	-0.09	-0.14 – -0.03
Injunctive inclusivity norms x RWA	-0.00	-0.08 – 0.07
Descriptive inclusivity norms x RWA	0.03	-0.03 – 0.10
Observations	998	
R^2 Bayes	0.066	
BF_{01}	81.11	

Table S76.
Bayesian moderated multiple regression results (British sample)

	Tolerance	
Predictors	*Estimates*	*CI (95%)*
Injunctive inclusivity norms	0.07	-0.21 – 0.35
RWA	-0.10	-0.30 – 0.11
Descriptive inclusivity norms	0.06	-0.21 – 0.34
Topic importance	-0.01	-0.07 – 0.04
Opinion extremity	-0.02	-0.11 – 0.06
Strength of disapproval	-0.09	-0.15 – -0.04
Injunctive inclusivity norms x RWA	0.00	-0.07 – 0.07
Descriptive inclusivity norms x RWA	0.00	-0.06 – 0.07
Observations	1002	
R2 Bayes	0.051	
BF_{01}	233.02	

	Avoidance tendencies	
Predictors	*Estimates*	*CI (95%)*
Injunctive inclusivity norms	-0.23	-0.59 – 0.13
RWA	-0.31	-0.58 – -0.04
Descriptive inclusivity norms	-0.34	-0.69 – 0.02
Topic importance	0.03	-0.04 – 0.10
Opinion extremity	0.11	-0.01 – 0.22
Strength of disapproval	0.24	0.17 – 0.31
Injunctive inclusivity norms x RWA	0.04	-0.05 – 0.13
Descriptive inclusivity norms x RWA	0.06	-0.03 – 0.14
Observations	1002	
R^2 Bayes	0.115	
BF_{01}	0.38	

	Cooperation willingness	
Predictors	*Estimates*	*CI (95%)*
Injunctive inclusivity norms	-0.15	-0.46 – 0.15
RWA	-0.32	-0.54 – -0.08
Descriptive inclusivity norms	0.22	-0.07 – 0.52
Topic importance	0.11	0.05 – 0.17
Opinion extremity	-0.01	-0.10 – 0.08

Strength of disapproval	-0.05	-0.11 – 0.01
Injunctive inclusivity norms x RWA	0.07	-0.01 – 0.14
Descriptive inclusivity norms x RWA	-0.02	-0.09 – 0.04
Observations	1002	
R^2 Bayes	0.077	
BF_{01}	16.56	

Opinion on Polarized Issue

Table S76.
Bayesian moderated multiple regression results by topic including opinion on the chosen topic as moderator (topic: migration policies)

	**Tolerance**	
Predictors	*Estimates*	*CI (95%)*
Injunctive inclusivity norms	0.24	0.01 – 0.47
Opinion on topic	0.06	-0.42– 0.54
Descriptive inclusivity norms	0.12	-0.11 – 0.34
Topic importance	0.04	0.01 –0.07
Opinion extremity	-0.12	-0.17 – -0.07
Strength of disapproval	-0.14	-0.18 – -0.11
Injunctive inclusivity norms x Opinion on topic	-0.02	-0.15 – 0.10
Descriptive inclusivity norms x Opinion on topic	-0.07	-0.19 – 0.05
Observations	2616	
R^2 Bayes	0.11	
BF_{01}	11.22	
	**Avoidance tendencies**	
Predictors	*Estimates*	*CI (95%)*
Injunctive inclusivity norms	-0.39	-0.67 – -0.11
Opinion on topic	-0.13	-0.73 – 0.49
Descriptive inclusivity norms	0.25	-0.03 – 0.54
Topic importance	-0.02	-0.05 – 0.02
Opinion extremity	0.21	0.15 – 0.27
Strength of disapproval	0.24	0.20 – 0.28
Injunctive inclusivity norms x Opinion on topic	0.13	-0.02 – 0.29
Descriptive inclusivity norms x Opinion on topic	-0.10	-0.25 – 0.06
Observations	2616	
R^2 Bayes	0.12	
BF_{01}	9.05	
	**Cooperation willingness**	
Predictors	*Estimates*	*CI (95%)*
Injunctive inclusivity norms	0.19	-0.07 – 0.46

Opinion on topic	-0.23	-0.78 – 0.35
Descriptive inclusivity norms	0.17	-0.10 – 0.43
Topic importance	0.11	0.08 – 0.15
Opinion extremity	-0.14	-0.19 – -0.08
Strength of disapproval	-0.13	-0.16 – -0.09
Injunctive inclusivity norms x Opinion on topic	0.02	-0.13 – 0.16
Descriptive inclusivity norms x Opinion on topic	-0.08	-0.22 – 0.07
Observations	2616	
R^2 Bayes	0.12	
BF_{01}	18.31	

Note. Opinion on topic was coded as 1 = progressive opinion and 2 = conservative opinion.

Table S77.
Bayesian moderated multiple regression results by topic including opinion on the chosen topic as moderator (topic: gender equality)

	Tolerance	
Predictors	*Estimates*	*CI (95%)*
Injunctive inclusivity norms	0.10	-0.11 – 0.32
Opinion on topic	0.90	0.07 – 1.70
Descriptive inclusivity norms	0.32	0.11 – 0.52
Topic importance	0.01	-0.03 – 0.05
Opinion extremity	-0.00	-0.06 – 0.04
Strength of disapproval	-0.09	-0.13 – -0.06
Injunctive inclusivity norms x Opinion on topic	-0.01	-0.21 – 0.19
Descriptive inclusivity norms x Opinion on topic	-0.18	-0.36 – 0.01
Observations	2876	
R^2 Bayes	0.06	
BF_{01}	1.69	
	**Avoidance tendencies**	
Predictors	*Estimates*	*CI (95%)*
Injunctive inclusivity norms	-0.11	-0.39 – 0.16
Opinion on topic	-0.75	-1.76 – 0.31
Descriptive inclusivity norms	-0.15	-0.41 – 0.12
Topic importance	0.08	0.02 – 0.13
Opinion extremity	-0.00	-0.07 – 0.06
Strength of disapproval	0.20	0.16 – 0.24
Injunctive inclusivity norms x Opinion on topic	-0.00	-0.25 – 0.26
Descriptive inclusivity norms x Opinion on topic	0.17	-0.08 – 0.41
Observations	2876	
R^2 Bayes	0.06	
BF_{01}	3.16	
	**Cooperation willingness**	
Predictors	*Estimates*	*CI (95%)*
Injunctive inclusivity norms	0.01	-0.22 – 0.24
Opinion on topic	0.06	-0.81 – 0.91
Descriptive inclusivity norms	0.26	0.04 – 0.48

Topic importance	0.09	0.04 – 0.13
Opinion extremity	0.12	0.07 – 0.17
Strength of disapproval	-0.04	-0.07 – -0.01
Injunctive inclusivity norms x Opinion on topic	0.09	-0.12 – 0.31
Descriptive inclusivity norms x Opinion on topic	-0.13	-0.33 – 0.08
Observations	2876	
R^2 Bayes	0.08	
BF_{01}	8.04	

Note. Opinion on topic was coded as 1 = progressive opinion and 2 = conservative opinion.

Table S78.
Bayesian moderated multiple regression results by topic including opinion on the chosen topic as moderator (topic: transgender rights)

	**Tolerance**	
Predictors	*Estimates*	*CI (95%)*
Injunctive inclusivity norms	-0.11	-0.49 – 0.25
Opinion on topic	-1.35	-2.39 – -0.32
Descriptive inclusivity norms	0.23	-0.11 – 0.58
Topic importance	0.11	-0.06 – 0.08
Opinion extremity	-0.01	-0.13 – 0.10
Strength of disapproval	-0.15	-0.21 – -0.08
Injunctive inclusivity norms x Opinion on topic	0.15	-0.09 – 0.42
Descriptive inclusivity norms x Opinion on topic	-0.10	-0.36 – 0.15
Observations	581	
R^2 Bayes	0.16	
BF_{01}	6.00	
	**Avoidance tendencies**	
Predictors	*Estimates*	*CI (95%)*
Injunctive inclusivity norms	-0.08	-0.50 – 0.36
Opinion on topic	0.37	-0.87 – 1.66
Descriptive inclusivity norms	-0.19	-0.61 – 0.22
Topic importance	0.09	0.01 – 0.17
Opinion extremity	0.13	-0.00 – 0.27
Strength of disapproval	0.22	0.14 – 0.30
Injunctive inclusivity norms x Opinion on topic	0.07	-0.25 – 0.37
Descriptive inclusivity norms x Opinion on topic	0.12	-0.17 – 0.42
Observations	581	
R^2 Bayes	0.16	
BF_{01}	3.09	
	**Cooperation willingness**	
Predictors	*Estimates*	*CI (95%)*
Injunctive inclusivity norms	-0.05	-0.45 – 0.36
Opinion on topic	-1.45	-2.53 – -0.36
Descriptive inclusivity norms	0.03	-0.36 – 0.44

Topic importance	0.06	-0.02 – 0.13
Opinion extremity	-0.03	-0.15 – 0.09
Strength of disapproval	-0.09	-0.16 – -0.02
Injunctive inclusivity norms x Opinion on topic	0.13	-0.16 – 0.42
Descriptive inclusivity norms x Opinion on topic	0.01	-0.27 – 0.29
Observations	581	
R^2 Bayes	0.10	
BF_{01}	4.52	

Note. Opinion on topic was coded as 1 = progressive opinion and 2 = conservative opinion.

Additional Analyses

Injunctive Inclusivity Norms

Table S79.

Bayesian multiple regression results including only injunctive inclusivity norms (Czech sample)

	**Tolerance**	
Predictors	*Estimates*	*CI (95%)*
Injunctive inclusivity norms	0.16	0.10 – 0.22
Topic importance	0.05	0.00 – 0.10
Opinion extremity	0.02	-0.05 – 0.09
Strength of disapproval	-0.20	-0.24 – -0.15
Observations	1002	
R^2 Bayes	0.098	
	**Avoidance tendencies**	
Predictors	*Estimates*	*CI (95%)*
Injunctive inclusivity norms	-0.03	-0.11 – 0.05
Topic importance	-0.03	-0.09 – 0.03
Opinion extremity	0.02	-0.07 – 0.11
Strength of disapproval	0.31	0.25 – 0.37
Observations	1002	
R^2 Bayes	0.119	
	**Cooperation willingness**	
Predictors	*Estimates*	*CI (95%)*
Injunctive inclusivity norms	0.21	0.14 – 0.28
Topic importance	0.10	0.04 – 0.15
Opinion extremity	0.01	-0.07 – 0.08
Strength of disapproval	-0.14	-0.19 – -0.08
Observations	1002	
R^2 Bayes	0.072	

Table S80.

Bayesian multiple regression results including only injunctive inclusivity norms (Finnish sample)

	**Tolerance**	
Predictors	*Estimates*	*CI (95%)*
Injunctive inclusivity norms	0.15	0.08 – 0.21
Topic importance	0.03	-0.01 – 0.08
Opinion extremity	-0.04	-0.13 – 0.04
Strength of disapproval	-0.34	-0.39 – -0.29
Observations	1001	
R^2 Bayes	0.211	
	**Avoidance tendencies**	
Predictors	*Estimates*	*CI (95%)*
Injunctive inclusivity norms	-0.17	-0.25 – -0.08
Topic importance	-0.05	-0.10 – 0.01
Opinion extremity	0.02	-0.09 – 0.12
Strength of disapproval	0.41	0.35 – 0.48
Observations	1001	
R^2 Bayes	0.193	
	**Cooperation willingness**	
Predictors	*Estimates*	*CI (95%)*
Injunctive inclusivity norms	0.23	0.15 – 0.31
Topic importance	0.16	0.11 – 0.22
Opinion extremity	0.02	-0.07 – 0.13
Strength of disapproval	-0.22	-0.28 – -0.16
Observations	1001	
R^2 Bayes	0.107	

Table S81.

Bayesian multiple regression results including only injunctive inclusivity norms (French sample)

	**Tolerance**	
Predictors	*Estimates*	*CI (95%)*
Injunctive inclusivity norms	0.17	0.12 – 0.23
Topic importance	0.04	-0.01 – 0.10
Opinion extremity	0.01	-0.06 – 0.08
Strength of disapproval	-0.13	-0.18 – -0.09
Observations	1005	
R^2 Bayes	0.074	
	**Avoidance tendencies**	
Predictors	*Estimates*	*CI (95%)*
Injunctive inclusivity norms	0.02	-0.06 – 0.09
Topic importance	-0.01	-0.08 – 0.07
Opinion extremity	0.12	0.03 – 0.22
Strength of disapproval	0.18	0.12 – 0.24
Observations	1005	
R^2 Bayes	0.058	
	**Cooperation willingness**	
Predictors	*Estimates*	*CI (95%)*
Injunctive inclusivity norms	0.20	0.14 – 0.26
Topic importance	0.12	0.05 – 0.18
Opinion extremity	0.07	-0.01 – 0.15
Strength of disapproval	-0.08	-0.13 – -0.02
Observations	1005	
R^2 Bayes	0.067	

Table S82.

Bayesian multiple regression results including only injunctive inclusivity norms (German sample)

	Tolerance	
Predictors	*Estimates*	*CI (95%)*
Injunctive inclusivity norms	0.24	0.17 – 0.30
Topic importance	0.05	0.00 – 0.10
Opinion extremity	0.03	-0.05 – 0.10
Strength of disapproval	-0.14	-0.19 – -0.09
Observations	1009	
R^2 Bayes	0.094	
	**Avoidance tendencies**	
Predictors	*Estimates*	*CI (95%)*
Injunctive inclusivity norms	-0.01	-0.10 – 0.07
Topic importance	-0.02	-0.09 – 0.05
Opinion extremity	0.02	-0.08 – 0.12
Strength of disapproval	0.39	0.32 – 0.45
Observations	1009	
R^2 Bayes	0.126	
	**Cooperation willingness**	
Predictors	*Estimates*	*CI (95%)*
Injunctive inclusivity norms	0.27	0.20 – 0.34
Topic importance	0.20	0.14 – 0.25
Opinion extremity	-0.02	-0.10 – 0.06
Strength of disapproval	-0.10	-0.15 – -0.04
Observations	1009	
R^2 Bayes	0.120	

Table S83.

Bayesian multiple regression results including only injunctive inclusivity norms (Greek sample)

	Tolerance	
Predictors	*Estimates*	*CI (95%)*
Injunctive inclusivity norms	0.17	0.11 – 0.23
Topic importance	0.05	-0.01 – 0.10
Opinion extremity	-0.05	-0.13 – 0.04
Strength of disapproval	-0.13	-0.19 – -0.08
Observations	1009	
R^2 Bayes	0.065	

	Avoidance tendencies	
Predictors	*Estimates*	*CI (95%)*
Injunctive inclusivity norms	-0.05	-0.13 – 0.03
Topic importance	-0.03	-0.10 – 0.04
Opinion extremity	0.06	-0.04 – 0.17
Strength of disapproval	0.29	0.22 – 0.35
Observations	1009	
R^2 Bayes	0.094	

	Cooperation willingness	
Predictors	*Estimates*	*CI (95%)*
Injunctive inclusivity norms	0.19	0.12 – 0.26
Topic importance	0.13	0.07 – 0.19
Opinion extremity	-0.06	-0.15 – 0.03
Strength of disapproval	-0.15	-0.20 – -0.09
Observations	1009	
R^2 Bayes	0.070	

Table S84.

Bayesian multiple regression results including only injunctive inclusivity norms (Hungarian sample)

	**Tolerance**	
Predictors	*Estimates*	*CI (95%)*
Injunctive inclusivity norms	0.10	0.04 – 0.15
Topic importance	0.04	-0.00 – 0.08
Opinion extremity	0.06	-0.02 – 0.14
Strength of disapproval	-0.14	-0.18 – -0.09
Observations	1005	
R^2 Bayes	0.049	
	**Avoidance tendencies**	
Predictors	*Estimates*	*CI (95%)*
Injunctive inclusivity norms	-0.01	-0.08 – 0.07
Topic importance	0.02	-0.04 – 0.08
Opinion extremity	0.02	-0.08 – 0.12
Strength of disapproval	0.22	0.16 – 0.28
Observations	1005	
R^2 Bayes	0.069	
	**Cooperation willingness**	
Predictors	*Estimates*	*CI (95%)*
Injunctive inclusivity norms	0.09	0.02 – 0.16
Topic importance	0.11	0.06 – 0.16
Opinion extremity	0.01	-0.09 – 0.10
Strength of disapproval	-0.10	-0.15 – -0.04
Observations	1005	
R^2 Bayes	0.034	

Table S85.

Bayesian multiple regression results including only injunctive inclusivity norms (Italian sample)

	Tolerance	
Predictors	*Estimates*	*CI (95%)*
Injunctive inclusivity norms	0.18	0.12 – 0.23
Topic importance	0.03	-0.03 – 0.08
Opinion extremity	-0.05	-0.11 – 0.02
Strength of disapproval	-0.11	-0.16 – -0.07
Observations	1003	
R^2 Bayes	0.073	

	Avoidance tendencies	
Predictors	*Estimates*	*CI (95%)*
Injunctive inclusivity norms	-0.08	-0.16 – -0.00
Topic importance	0.02	-0.05 – 0.10
Opinion extremity	0.06	-0.03 – 0.15
Strength of disapproval	0.26	0.20 – 0.33
Observations	1003	
R^2 Bayes	0.081	

	Cooperation willingness	
Predictors	*Estimates*	*CI (95%)*
Injunctive inclusivity norms	0.22	0.16 – 0.28
Topic importance	0.16	0.10 – 0.23
Opinion extremity	-0.00	-0.08 – 0.07
Strength of disapproval	-0.09	-0.14 – -0.04
Observations	1003	
R^2 Bayes	0.091	

Table S86.

Bayesian multiple regression results including only injunctive inclusivity norms (Dutch sample)

	**Tolerance**	
Predictors	*Estimates*	*CI (95%)*
Injunctive inclusivity norms	0.15	0.08 – 0.22
Topic importance	0.07	0.04 – 0.10
Opinion extremity	-0.04	-0.12 – 0.03
Strength of disapproval	0.03	-0.01 – 0.07
Observations	1005	
R^2 Bayes	0.041	
	**Avoidance tendencies**	
Predictors	*Estimates*	*CI (95%)*
Injunctive inclusivity norms	-0.09	-0.19 – 0.00
Topic importance	-0.06	-0.11 – -0.01
Opinion extremity	0.21	0.10 – 0.32
Strength of disapproval	0.03	-0.02 – 0.09
Observations	1005	
R^2 Bayes	0.029	
	**Cooperation willingness**	
Predictors	*Estimates*	*CI (95%)*
Injunctive inclusivity norms	0.24	0.18 – 0.31
Topic importance	0.06	0.01 – 0.11
Opinion extremity	0.02	-0.06 – 0.10
Strength of disapproval	-0.01	-0.06 – 0.04
Observations	1003	
R^2 Bayes	0.064	

Table S87.

Bayesian multiple regression results including only injunctive inclusivity norms (Polish sample)

	**Tolerance**	
Predictors	*Estimates*	*CI (95%)*
Injunctive inclusivity norms	0.15	0.09 – 0.22
Topic importance	-0.04	-0.08 – 0.01
Opinion extremity	-0.03	-0.11 – 0.04
Strength of disapproval	-0.06	-0.10 – -0.01
Observations	1003	
R^2 Bayes	0.039	
	**Avoidance tendencies**	
Predictors	*Estimates*	*CI (95%)*
Injunctive inclusivity norms	-0.06	-0.14 – 0.02
Topic importance	0.04	-0.02 – 0.10
Opinion extremity	0.11	0.01 – 0.21
Strength of disapproval	0.11	0.04 – 0.17
Observations	1003	
R^2 Bayes	0.028	
	**Cooperation willingness**	
Predictors	*Estimates*	*CI (95%)*
Injunctive inclusivity norms	0.24	0.18 – 0.31
Topic importance	0.06	0.01 – 0.11
Opinion extremity	0.02	-0.06 – 0.10
Strength of disapproval	-0.01	-0.06 – 0.04
Observations	1003	
R^2 Bayes	0.064	

Table S88.

Bayesian multiple regression results including only injunctive inclusivity norms (Spanish sample)

	**Tolerance**	
Predictors	*Estimates*	*CI (95%)*
Injunctive inclusivity norms	0.20	0.14 – 0.26
Topic importance	-0.01	-0.07 – 0.04
Opinion extremity	-0.05	-0.13 – 0.03
Strength of disapproval	-0.14	-0.19 – -0.10
Observations	999	
R^2 Bayes	0.086	
	**Avoidance tendencies**	
Predictors	*Estimates*	*CI (95%)*
Injunctive inclusivity norms	-0.05	-0.13 – 0.03
Topic importance	0.05	-0.02 – 0.13
Opinion extremity	0.02	-0.08 – 0.12
Strength of disapproval	0.23	0.17 – 0.29
Observations	999	
R^2 Bayes	0.072	
	**Cooperation willingness**	
Predictors	*Estimates*	*CI (95%)*
Injunctive inclusivity norms	0.18	0.12 – 0.24
Topic importance	0.14	0.08 – 0.19
Opinion extremity	0.07	-0.02 – 0.15
Strength of disapproval	-0.09	-0.14 – -0.04
Observations	999	
R^2 Bayes	0.072	

Table S89.

Bayesian multiple regression results including only injunctive inclusivity norms (Swedish sample)

	**Tolerance**	
Predictors	*Estimates*	*CI (95%)*
Injunctive inclusivity norms	0.20	0.13 – 0.26
Topic importance	-0.06	-0.11 – -0.00
Opinion extremity	0.06	-0.02 – 0.13
Strength of disapproval	-0.07	-0.12 – -0.02
Observations	998	
R^2 Bayes	0.050	
	**Avoidance tendencies**	
Predictors	*Estimates*	*CI (95%)*
Injunctive inclusivity norms	-0.13	-0.21 – -0.05
Topic importance	0.05	-0.02 – 0.12
Opinion extremity	0.02	-0.08 – 0.11
Strength of disapproval	0.22	0.16 – 0.28
Observations	998	
R^2 Bayes	0.084	
	**Cooperation willingness**	
Predictors	*Estimates*	*CI (95%)*
Injunctive inclusivity norms	0.21	0.14 – 0.28
Topic importance	0.07	0.01 – 0.13
Opinion extremity	0.07	-0.01 – 0.15
Strength of disapproval	-0.08	-0.14 – -0.03
Observations	998	
R^2 Bayes	0.057	

Table S90.

Bayesian multiple regression results including only injunctive inclusivity norms (British sample)

	**Tolerance**	
Predictors	*Estimates*	*CI (95%)*
Injunctive inclusivity norms	0.11	0.05 – 0.17
Topic importance	-0.01	-0.07 – 0.04
Opinion extremity	-0.03	-0.11 – 0.06
Strength of disapproval	-0.09	-0.15 – -0.04
Observations	1002	
R^2 Bayes	0.039	
	**Avoidance tendencies**	
Predictors	*Estimates*	*CI (95%)*
Injunctive inclusivity norms	-0.10	-0.18 – -0.02
Topic importance	0.04	-0.03 – 0.11
Opinion extremity	0.11	0.00 – 0.22
Strength of disapproval	0.24	0.17 – 0.32
Observations	1002	
R^2 Bayes	0.092	
	**Cooperation willingness**	
Predictors	*Estimates*	*CI (95%)*
Injunctive inclusivity norms	0.19	0.13 – 0.26
Topic importance	0.11	0.05 – 0.17
Opinion extremity	-0.01	-0.10 – 0.08
Strength of disapproval	-0.05	-0.11 – 0.01
Observations	1002	
R^2 Bayes	0.053	

Descriptive Inclusivity Norms

Table S91.

Bayesian multiple regression results including only descriptive inclusivity norms (Czech sample)

	**Tolerance**	
Predictors	*Estimates*	*CI (95%)*
Descriptive inclusivity norms	0.17	0.11 – 0.23
Topic importance	0.05	0.01 – 0.10
Opinion extremity	0.02	-0.05 – 0.09
Strength of disapproval	-0.20	-0.25 – -0.15
Observations	1002	
R^2 Bayes	0.103	
	**Avoidance tendencies**	
Predictors	*Estimates*	*CI (95%)*
Descriptive inclusivity norms	-0.01	-0.09 – 0.06
Topic importance	-0.03	-0.09 – 0.03
Opinion extremity	0.02	-0.07 – 0.11
Strength of disapproval	0.31	0.25 – 0.37
Observations	1002	
R^2 Bayes	0.118	
	**Cooperation willingness**	
Predictors	*Estimates*	*CI (95%)*
Descriptive inclusivity norms	0.21	0.14 – 0.28
Topic importance	0.10	0.05 – 0.15
Opinion extremity	0.01	-0.07 – 0.09
Strength of disapproval	-0.14	-0.19 – -0.09
Observations	1002	
R^2 Bayes	0.073	

Table S92.

Bayesian multiple regression results including only descriptive inclusivity norms (Finnish sample)

	**Tolerance**	
Predictors	*Estimates*	*CI (95%)*
Descriptive inclusivity norms	0.15	0.08 – 0.21
Topic importance	0.03	-0.01 – 0.08
Opinion extremity	-0.04	-0.12 – 0.05
Strength of disapproval	-0.34	-0.39 – -0.29
Observations	1001	
R^2 Bayes	0.211	
	**Avoidance tendencies**	
Predictors	*Estimates*	*CI (95%)*
Descriptive inclusivity norms	-0.10	-0.18 – -0.02
Topic importance	-0.05	-0.11 – 0.01
Opinion extremity	0.02	-0.10 – 0.12
Strength of disapproval	0.42	0.36 – 0.49
Observations	1001	
R^2 Bayes	0.185	
	**Cooperation willingness**	
Predictors	*Estimates*	*CI (95%)*
Descriptive inclusivity norms	0.21	0.14 – 0.29
Topic importance	0.17	0.12 – 0.22
Opinion extremity	0.03	-0.07 – 0.14
Strength of disapproval	-0.22	-0.28 – -0.16
Observations	1001	
R^2 Bayes	0.104	

Table S93.

Bayesian multiple regression results including only descriptive inclusivity norms (French sample)

	**Tolerance**	
Predictors	*Estimates*	*CI (95%)*
Descriptive inclusivity norms	0.10	0.05 – 0.15
Topic importance	0.04	-0.01 – 0.10
Opinion extremity	0.02	-0.05 – 0.09
Strength of disapproval	-0.13	-0.18 – -0.09
Observations	1005	
R^2 Bayes	0.054	
	**Avoidance tendencies**	
Predictors	*Estimates*	*CI (95%)*
Descriptive inclusivity norms	0.07	-0.01 – 0.14
Topic importance	-0.01	-0.09 – 0.07
Opinion extremity	0.12	0.02 – 0.21
Strength of disapproval	0.19	0.12 – 0.25
Observations	1005	
R^2 Bayes	0.061	
	**Cooperation willingness**	
Predictors	*Estimates*	*CI (95%)*
Descriptive inclusivity norms	0.13	0.07 – 0.20
Topic importance	0.12	0.05 – 0.18
Opinion extremity	0.07	-0.01 – 0.15
Strength of disapproval	-0.07	-0.13 – -0.02
Observations	1005	
R^2 Bayes	0.051	

Table S94.

Bayesian multiple regression results including only descriptive inclusivity norms (German sample)

	Tolerance	
Predictors	*Estimates*	*CI (95%)*
Descriptive inclusivity norms	0.17	0.12 – 0.22
Topic importance	0.07	0.02 – 0.12
Opinion extremity	0.04	-0.04 – 0.11
Strength of disapproval	-0.16	-0.21 – -0.11
Observations	1009	
R^2 Bayes	0.075	
	**Avoidance tendencies**	
Predictors	*Estimates*	*CI (95%)*
Descriptive inclusivity norms	0.08	0.01 – 0.16
Topic importance	-0.03	-0.10 – 0.04
Opinion extremity	0.03	-0.07 – 0.13
Strength of disapproval	0.39	0.31 – 0.45
Observations	1009	
R^2 Bayes	0.130	
	**Cooperation willingness**	
Predictors	*Estimates*	*CI (95%)*
Descriptive inclusivity norms	0.16	0.10 – 0.22
Topic importance	0.21	0.16 – 0.27
Opinion extremity	-0.01	-0.09 – 0.07
Strength of disapproval	-0.11	-0.17 – -0.05
Observations	1009	
R^2 Bayes	0.091	

Table S95.

Bayesian multiple regression results including only descriptive inclusivity norms (Greek sample)

	**Tolerance**	
Predictors	*Estimates*	*CI (95%)*
Descriptive inclusivity norms	0.18	0.13 – 0.24
Topic importance	0.05	-0.01 – 0.10
Opinion extremity	-0.03	-0.11 – 0.05
Strength of disapproval	-0.15	-0.20 – -0.10
Observations	1009	
R^2 Bayes	0.072	
	**Avoidance tendencies**	
Predictors	*Estimates*	*CI (95%)*
Descriptive inclusivity norms	-0.03	-0.10 – 0.04
Topic importance	-0.03	-0.11 – 0.04
Opinion extremity	0.06	-0.04 – 0.16
Strength of disapproval	0.29	0.23 – 0.35
Observations	1009	
R^2 Bayes	0.092	
	**Cooperation willingness**	
Predictors	*Estimates*	*CI (95%)*
Descriptive inclusivity norms	0.20	0.13 – 0.26
Topic importance	0.13	0.07 – 0.19
Opinion extremity	-0.04	-0.13 – 0.04
Strength of disapproval	-0.16	-0.22 – -0.11
Observations	1009	
R^2 Bayes	0.074	

Table S96.

Bayesian multiple regression results including only descriptive inclusivity norms (Hungarian sample)

	**Tolerance**	
Predictors	*Estimates*	*CI (95%)*
Descriptive inclusivity norms	0.07	0.02 – 0.12
Topic importance	0.04	-0.00 – 0.08
Opinion extremity	0.06	-0.01 – 0.14
Strength of disapproval	-0.14	-0.18 – -0.10
Observations	1005	
R^2 Bayes	0.044	

	**Avoidance tendencies**	
Predictors	*Estimates*	*CI (95%)*
Descriptive inclusivity norms	0.04	-0.03 – 0.11
Topic importance	0.02	-0.04 – 0.08
Opinion extremity	0.02	-0.08 – 0.13
Strength of disapproval	0.22	0.16 – 0.28
Observations	1005	
R^2 Bayes	0.071	

	**Cooperation willingness**	
Predictors	*Estimates*	*CI (95%)*
Descriptive inclusivity norms	0.10	0.04 – 0.17
Topic importance	0.11	0.06 – 0.16
Opinion extremity	0.01	-0.08 – 0.10
Strength of disapproval	-0.10	-0.15 – -0.04
Observations	1005	
R^2 Bayes	0.037	

Table S97.

Bayesian multiple regression results including only descriptive inclusivity norms (Italian sample)

	**Tolerance**	
Predictors	*Estimates*	*CI (95%)*
Descriptive inclusivity norms	0.16	0.11 – 0.21
Topic importance	0.03	-0.03 – 0.08
Opinion extremity	-0.03	-0.10 – 0.04
Strength of disapproval	-0.12	-0.17 – -0.08
Observations	1003	
R^2 Bayes	0.069	
	**Avoidance tendencies**	
Predictors	*Estimates*	*CI (95%)*
Descriptive inclusivity norms	0.00	-0.07 – 0.07
Topic importance	0.01	-0.07 – 0.09
Opinion extremity	0.06	-0.04 – 0.15
Strength of disapproval	0.27	0.20 – 0.34
Observations	1003	
R^2 Bayes	0.077	
	**Cooperation willingness**	
Predictors	*Estimates*	*CI (95%)*
Descriptive inclusivity norms	0.17	0.11 – 0.23
Topic importance	0.17	0.11 – 0.23
Opinion extremity	0.02	-0.06 – 0.10
Strength of disapproval	-0.10	-0.16 – -0.05
Observations	1003	
R^2 Bayes	0.075	

Table S98.

Bayesian multiple regression results including only descriptive inclusivity norms (Dutch sample)

	**Tolerance**	
Predictors	*Estimates*	*CI (95%)*
Descriptive inclusivity norms	0.12	0.05 – 0.18
Topic importance	0.07	0.03 – 0.10
Opinion extremity	-0.04	-0.11 – 0.04
Strength of disapproval	0.02	-0.02 – 0.06
Observations	1005	
R^2 Bayes	0.037	
	**Avoidance tendencies**	
Predictors	*Estimates*	*CI (95%)*
Descriptive inclusivity norms	-0.00	-0.09 – 0.08
Topic importance	-0.06	-0.11 – -0.02
Opinion extremity	0.21	0.10 – 0.32
Strength of disapproval	0.03	-0.03 – 0.08
Observations	1005	
R^2 Bayes	0.026	
	**Cooperation willingness**	
Predictors	*Estimates*	*CI (95%)*
Descriptive inclusivity norms	0.15	0.08 – 0.22
Topic importance	0.18	0.14 – 0.22
Opinion extremity	0.02	-0.07 – 0.10
Strength of disapproval	0.05	0.00 – 0.10
Observations	1005	
R^2 Bayes	0.101	

Table S99.

Bayesian multiple regression results including only descriptive inclusivity norms (Polish sample)

	**Tolerance**	
Predictors	*Estimates*	*CI (95%)*
Injunctive inclusivity norms	0.12	0.06 – 0.18
Topic importance	-0.04	-0.08 – 0.01
Opinion extremity	-0.02	-0.09 – 0.05
Strength of disapproval	-0.06	-0.11 – -0.01
Observations	1003	
R^2 Bayes	0.031	
	**Avoidance tendencies**	
Predictors	*Estimates*	*CI (95%)*
Descriptive inclusivity norms	0.06	-0.01 – 0.13
Topic importance	0.03	-0.03 – 0.10
Opinion extremity	0.11	0.01 – 0.21
Strength of disapproval	0.11	0.05 – 0.17
Observations	1003	
R^2 Bayes	0.029	
	**Cooperation willingness**	
Predictors	*Estimates*	*CI (95%)*
Descriptive inclusivity norms	0.17	0.11 – 0.23
Topic importance	0.07	0.01 – 0.12
Opinion extremity	0.03	-0.05 – 0.11
Strength of disapproval	-0.01	-0.06 – 0.04
Observations	1003	
R^2 Bayes	0.044	

Table S100.

Bayesian multiple regression results including only descriptive inclusivity norms (Spanish sample)

	**Tolerance**	
Predictors	*Estimates*	*CI (95%)*
Descriptive inclusivity norms	0.16	0.10 – 0.22
Topic importance	-0.00	-0.06 – 0.05
Opinion extremity	-0.05	-0.14 – 0.03
Strength of disapproval	-0.15	-0.20 – -0.10
Observations	999	
R^2 Bayes	0.072	

	**Avoidance tendencies**	
Predictors	*Estimates*	*CI (95%)*
Descriptive inclusivity norms	0.06	-0.02 – 0.13
Topic importance	0.05	-0.03 – 0.11
Opinion extremity	0.02	-0.09 – 0.13
Strength of disapproval	0.23	0.17 – 0.29
Observations	999	
R^2 Bayes	0.073	

	**Cooperation willingness**	
Predictors	*Estimates*	*CI (95%)*
Descriptive inclusivity norms	0.17	0.11 – 0.23
Topic importance	0.14	0.08 – 0.20
Opinion extremity	0.07	-0.02 – 0.15
Strength of disapproval	-0.10	-0.15 – -0.05
Observations	999	
R^2 Bayes	0.070	

Table S101.

Bayesian multiple regression results including only descriptive inclusivity norms (Swedish sample)

	**Tolerance**	
Predictors	*Estimates*	*CI (95%)*
Descriptive inclusivity norms	0.13	0.07 – 0.19
Topic importance	-0.07	-0.12 – -0.01
Opinion extremity	0.07	-0.00 – 0.15
Strength of disapproval	-0.08	-0.13 – -0.03
Observations	998	
R^2 Bayes	0.036	
	**Avoidance tendencies**	
Predictors	*Estimates*	*CI (95%)*
Descriptive inclusivity norms	-0.08	-0.15 – -0.00
Topic importance	0.05	-0.02 – 0.13
Opinion extremity	0.01	-0.09 – 0.10
Strength of disapproval	0.23	0.17 – 0.29
Observations	998	
R^2 Bayes	0.078	
	**Cooperation willingness**	
Predictors	*Estimates*	*CI (95%)*
Descriptive inclusivity norms	0.15	0.09 – 0.22
Topic importance	0.06	-0.00 – 0.12
Opinion extremity	0.09	0.01 – 0.17
Strength of disapproval	-0.09	-0.15 – -0.04
Observations	998	
R^2 Bayes	0.046	

Table S102.

Bayesian multiple regression results including only descriptive inclusivity norms (British sample)

	Tolerance	
Predictors	*Estimates*	*CI (95%)*
Descriptive inclusivity norms	0.11	0.06 – 0.16
Topic importance	-0.01	-0.07 – 0.04
Opinion extremity	-0.02	-0.11 – 0.06
Strength of disapproval	-0.10	-0.15 – -0.04
Observations	1002	
R^2 Bayes	0.040	

	Avoidance tendencies	
Predictors	*Estimates*	*CI (95%)*
Descriptive inclusivity norms	-0.10	-0.17 – -0.03
Topic importance	0.03	-0.04 – 0.11
Opinion extremity	0.10	-0.00 – 0.22
Strength of disapproval	0.25	0.18 – 0.32
Observations	1002	
R^2 Bayes	0.093	

	Cooperation willingness	
Predictors	*Estimates*	*CI (95%)*
Descriptive inclusivity norms	0.19	0.13 – 0.25
Topic importance	0.11	0.05 – 0.17
Opinion extremity	-0.00	-0.10 – 0.09
Strength of disapproval	-0.06	-0.12 – 0.00
Observations	1002	
R^2 Bayes	0.058	

Identification with Society

Table S103.

Bayesian moderated multiple regression results including identification with society as moderator (Czech sample)

	**Tolerance**	
Predictors	*Estimates*	*CI (95%)*
Injunctive inclusivity norms	0.15	-0.07 – 0.37
Identification with Society	0.11	-0.07 – 0.29
Descriptive inclusivity norms	-0.07	-0.30 – 0.15
Topic importance	0.04	0.00 – 0.09
Opinion extremity	0.03	-0.04 – 0.09
Strength of disapproval	-0.20	-0.24 – -0.15
Injunctive inclusivity norms x Identification with Society	-0.02	-0.07 – 0.02
Descriptive inclusivity norms x Identification with Society	0.03	-0.01 – 0.08
Observations	1002	
R^2 Bayes	0.136	

	**Avoidance tendencies**	
Predictors	*Estimates*	*CI (95%)*
Injunctive inclusivity norms	-0.14	-0.41 – 0.15
Identification with Society	-0.27	-0.50 – -0.05
Descriptive inclusivity norms	-0.00	-0.28 – 0.28
Topic importance	-0.03	-0.08 – 0.03
Opinion extremity	0.01	-0.08 – 0.10
Strength of disapproval	0.31	0.25 – 0.37

Injunctive inclusivity norms x Identification with Society	0.03	-0.03 – 0.09
Descriptive inclusivity norms x Identification with Society	0.01	-0.05 – 0.06
Observations	1002	
R^2 Bayes	0.133	

	**Cooperation willingness**	
Predictors	*Estimates*	*CI (95%)*
Injunctive inclusivity norms	0.18	-0.09 – 0.45
Identification with Society	0.18	-0.03 – 0.41
Descriptive inclusivity norms	0.06	-0.19 – 0.33
Topic importance	0.09	0.04 – 0.15
Opinion extremity	0.01	-0.07 – 0.10
Strength of disapproval	-0.14	-0.19 – -0.09
Injunctive inclusivity norms x Identification with Society	-0.02	-0.07 – 0.04
Descriptive inclusivity norms x Identification with Society	0.01	-0.05 – 0.06
Observations	1002	
R^2 Bayes	0.100	

Table S104.

Bayesian moderated multiple regression results including identification with society as moderator (Finnish sample)

	Tolerance	
Predictors	*Estimates*	*CI (95%)*
Injunctive inclusivity norms	-0.19	-0.44 – 0.07
Identification with Society	-0.18	-0.38 – 0.01
Descriptive inclusivity norms	-0.02	-0.31 – 0.25
Topic importance	0.03	-0.02 – 0.07
Opinion extremity	-0.05	-0.14 – 0.04
Strength of disapproval	-0.33	-0.38 – -0.27
Injunctive inclusivity norms x Identification with Society	0.05	0.00 – 0.10
Descriptive inclusivity norms x Identification with Society	0.02	-0.03 – 0.07
Observations	1001	
R^2 Bayes	0.237	

	Avoidance tendencies	
Predictors	*Estimates*	*CI (95%)*
Injunctive inclusivity norms	-0.05	-0.37 – 0.26
Identification with Society	0.03	-0.22 – 0.28
Descriptive inclusivity norms	0.13	-0.22 – 0.48
Topic importance	-0.04	-0.10 – 0.02
Opinion extremity	0.03	-0.08 – 0.14
Strength of disapproval	0.40	0.34 – 0.47
Injunctive inclusivity norms x Identification with Society	-0.02	-0.08 – 0.04
Descriptive inclusivity norms x Identification with Society	-0.02	-0.08 – 0.04
Observations	1001	
R^2 Bayes	0.205	

	Cooperation willingness	
Predictors	*Estimates*	*CI (95%)*
Injunctive inclusivity norms	-0.14	-0.43 – 0.16
Identification with Society	-0.16	-0.39 – 0.06
Descriptive inclusivity norms	0.05	-0.26 – 0.39
Topic importance	0.16	0.11 – 0.21

Opinion extremity	0.02	-0.08 – 0.12
Strength of disapproval	-0.21	-0.27 – -0.15
Injunctive inclusivity norms x Identification with Society	0.05	-0.00 – 0.11
Descriptive inclusivity norms x Identification with Society	0.01	-0.05 – 0.07
Observations	1001	
R^2 Bayes	0.132	

Table S105.

Bayesian moderated multiple regression results including identification with society as moderator (French sample)

	Tolerance	
Predictors	*Estimates*	*CI (95%)*
Injunctive inclusivity norms	0.18	-0.01 – 0.36
Identification with Society	-0.13	-0.27 – 0.02
Descriptive inclusivity norms	-0.33	-0.51 – -0.14
Topic importance	0.04	-0.02 – 0.10
Opinion extremity	0.00	-0.06 – 0.07
Strength of disapproval	-0.13	-0.17 – -0.08
Injunctive inclusivity norms x Identification with Society	-0.01	-0.04 – 0.03
Descriptive inclusivity norms x Identification with Society	0.07	0.03 – 0.10
Observations	1005	
R^2 Bayes	0.102	

	Avoidance tendencies	
Predictors	*Estimates*	*CI (95%)*
Injunctive inclusivity norms	-0.02	-0.27 – 0.23
Identification with Society	-0.09	-0.30 – 0.11
Descriptive inclusivity norms	0.23	-0.03 – 0.49
Topic importance	-0.01	-0.09 – 0.07
Opinion extremity	0.13	0.03 – 0.22
Strength of disapproval	0.19	0.13 – 0.24
Injunctive inclusivity norms x Identification with Society	0.00	-0.05 – 0.05
Descriptive inclusivity norms x Identification with Society	-0.02	-0.07 – 0.03
Observations	1005	
R^2 Bayes	0.084	

	Cooperation willingness	
Predictors	*Estimates*	*CI (95%)*
Injunctive inclusivity norms	0.06	-0.16 – 0.27
Identification with Society	-0.11	-0.27 – 0.06
Descriptive inclusivity norms	-0.07	-0.29 – 0.16
Topic importance	0.12	0.05 – 0.18

Opinion extremity	0.06	-0.02 – 0.14
Strength of disapproval	-0.07	-0.13 – -0.02
Injunctive inclusivity norms x Identification with Society	0.02	-0.02 – 0.06
Descriptive inclusivity norms x Identification with Society	0.02	-0.02 – 0.06
Observations	1005	
R^2 Bayes	0.080	

Table S106.

Bayesian moderated multiple regression results including identification with society as moderator (German sample)

	**Tolerance**	
Predictors	*Estimates*	*CI (95%)*
Injunctive inclusivity norms	0.17	-0.04 – 0.39
Identification with Society	-0.15	-0.31 – 0.02
Descriptive inclusivity norms	-0.30	-0.51 – -0.09
Topic importance	0.05	-0.01 – 0.10
Opinion extremity	0.01	-0.07 – 0.08
Strength of disapproval	-0.15	-0.20 – -0.10
Injunctive inclusivity norms x Identification with Society	-0.00	-0.05 – 0.04
Descriptive inclusivity norms x Identification with Society	0.07	0.03 – 0.11
Observations	1009	
R^2 Bayes	0.124	
	**Avoidance tendencies**	
Predictors	*Estimates*	*CI (95%)*
Injunctive inclusivity norms	0.01	-0.29 – 0.32
Identification with Society	0.10	-0.15 – 0.35
Descriptive inclusivity norms	0.38	0.09 – 0.67
Topic importance	-0.01	-0.08 – 0.06
Opinion extremity	0.06	-0.05 – 0.16
Strength of disapproval	0.38	0.31 – 0.45
Injunctive inclusivity norms x Identification with Society	-0.02	-0.08 – 0.04
Descriptive inclusivity norms x Identification with Society	-0.04	-0.10 – 0.01
Observations	1009	
R^2 Bayes	0.152	
	**Cooperation willingness**	
Predictors	*Estimates*	*CI (95%)*
Injunctive inclusivity norms	0.44	0.20 – 0.67
Identification with Society	-0.02	-0.21 – 0.17
Descriptive inclusivity norms	-0.49	-0.73 – -0.25
Topic importance	0.19	0.13 – 0.25

Opinion extremity	-0.04	-0.13 – 0.04
Strength of disapproval	-0.10	-0.16 – -0.04
Injunctive inclusivity norms x Identification with Society	-0.05	-0.09 – -0.00
Descriptive inclusivity norms x Identification with Society	0.10	0.05 – 0.14
Observations	1009	
R^2 Bayes	0.156	

Table S107.

Bayesian moderated multiple regression results including identification with society as moderator (Greek sample)

	**Tolerance**	
Predictors	*Estimates*	*CI (95%)*
Injunctive inclusivity norms	-0.08	-0.26 – 0.10
Identification with Society	-0.16	-0.31 – -0.01
Descriptive inclusivity norms	0.09	-0.10 – 0.27
Topic importance	0.04	-0.02 – 0.09
Opinion extremity	-0.04	-0.11 – 0.04
Strength of disapproval	-0.15	-0.20 – -0.10
Injunctive inclusivity norms x Identification with Society	0.04	-0.00 – 0.08
Descriptive inclusivity norms x Identification with Society	0.01	-0.04 – 0.05
Observations	1009	
R^2 Bayes	0.086	

	**Avoidance tendencies**	
Predictors	*Estimates*	*CI (95%)*
Injunctive inclusivity norms	0.11	-0.12 – 0.34
Identification with Society	0.01	-0.18 – 0.21
Descriptive inclusivity norms	-0.11	-0.34 – 0.13
Topic importance	-0.03	-0.10 – 0.04
Opinion extremity	0.07	-0.03 – 0.17
Strength of disapproval	0.29	0.22 – 0.35
Injunctive inclusivity norms x Identification with Society	-0.04	-0.09 – 0.01
Descriptive inclusivity norms x Identification with Society	0.03	-0.02 – 0.08
Observations	1009	
R^2 Bayes	0.100	

	**Cooperation willingness**	
Predictors	*Estimates*	*CI (95%)*
Injunctive inclusivity norms	-0.17	-0.37 – 0.03
Identification with Society	-0.16	-0.33 – -0.00
Descriptive inclusivity norms	0.19	-0.02 – 0.41
Topic importance	0.12	0.06 – 0.18

Opinion extremity	-0.05	-0.15 – 0.04
Strength of disapproval	-0.16	-0.21 – -0.10
Injunctive inclusivity norms x Identification with Society	0.07	0.02 – 0.11
Descriptive inclusivity norms x Identification with Society	-0.02	-0.07 – 0.02
Observations	1009	
R^2 Bayes	0.091	

Table S108.

Bayesian moderated multiple regression results including identification with society as moderator (Hungarian sample)

	Tolerance	
Predictors	*Estimates*	*CI (95%)*
Injunctive inclusivity norms	-0.16	-0.36 – 0.04
Identification with Society	-0.16	-0.30 – -0.03
Descriptive inclusivity norms	0.10	-0.10 – 0.29
Topic importance	0.04	-0.00 – 0.08
Opinion extremity	0.06	-0.02 – 0.13
Strength of disapproval	-0.14	-0.19 – -0.10
Injunctive inclusivity norms x Identification with Society	0.06	0.02 – 0.10
Descriptive inclusivity norms x Identification with Society	-0.02	-0.06 – 0.02
Observations	1005	
R^2 Bayes	0.061	

	Avoidance tendencies	
Predictors	*Estimates*	*CI (95%)*
Injunctive inclusivity norms	0.03	-0.24 – 0.29
Identification with Society	-0.20	-0.37 – -0.02
Descriptive inclusivity norms	-0.16	-0.42 – 0.10
Topic importance	0.02	-0.03 – 0.08
Opinion extremity	0.01	-0.09 – 0.12
Strength of disapproval	0.22	0.16 – 0.28
Injunctive inclusivity norms x Identification with Society	-0.02	-0.07 – 0.04
Descriptive inclusivity norms x Identification with Society	0.06	0.01 – 0.11
Observations	1005	
R^2 Bayes	0.084	

	Cooperation willingness	
Predictors	*Estimates*	*CI (95%)*
Injunctive inclusivity norms	-0.12	-0.35 – 0.11
Identification with Society	-0.12	-0.28 – 0.04
Descriptive inclusivity norms	0.03	-0.19 – 0.26
Topic importance	0.11	0.06 – 0.16

Opinion extremity	0.00	-0.09 – 0.10
Strength of disapproval	-0.10	-0.15 – -0.05
Injunctive inclusivity norms x Identification with Society	0.03	-0.02 – 0.08
Descriptive inclusivity norms x Identification with Society	0.01	-0.04 – 0.05
Observations	1005	
R^2 Bayes	0.046	

Table S109.

Bayesian moderated multiple regression results including identification with society as moderator (Italian sample)

	Tolerance	
Predictors	*Estimates*	*CI (95%)*
Injunctive inclusivity norms	-0.04	-0.20 – 0.11
Identification with Society	-0.22	-0.37 – -0.08
Descriptive inclusivity norms	-0.05	-0.22 – 0.11
Topic importance	0.02	-0.04 – 0.07
Opinion extremity	-0.05	-0.11 – 0.02
Strength of disapproval	-0.13	-0.17 – -0.08
Injunctive inclusivity norms x Identification with Society	0.04	0.00 – 0.08
Descriptive inclusivity norms x Identification with Society	0.03	-0.01 – 0.06
Observations	1003	
R^2 Bayes	0.102	

	Avoidance tendencies	
Predictors	*Estimates*	*CI (95%)*
Injunctive inclusivity norms	-0.00	-0.21 – 0.22
Identification with Society	-0.05	-0.26 – 0.16
Descriptive inclusivity norms	0.01	-0.21 – 0.24
Topic importance	0.02	-0.06 – 0.10
Opinion extremity	0.06	-0.03 – 0.16
Strength of disapproval	0.26	0.20 – 0.33
Injunctive inclusivity norms x Identification with Society	-0.03	-0.08 – 0.02
Descriptive inclusivity norms x Identification with Society	0.03	-0.02 – 0.07
Observations	1003	
R^2 Bayes	0.094	

	Cooperation willingness	
Predictors	*Estimates*	*CI (95%)*
Injunctive inclusivity norms	0.12	-0.06 – 0.29
Identification with Society	0.01	-0.16 – 0.17
Descriptive inclusivity norms	-0.01	-0.20 – 0.17
Topic importance	0.15	0.10 – 0.21

Opinion extremity	0.01	-0.07 – 0.08
Strength of disapproval	-0.10	-0.15 – -0.04
Injunctive inclusivity norms x Identification with Society	0.01	-0.03 – 0.05
Descriptive inclusivity norms x Identification with Society	0.01	-0.03 – 0.05
Observations	1003	
R^2 Bayes	0.108	

Table S110.

Bayesian moderated multiple regression results including identification with society as moderator (Dutch sample)

	Tolerance	
Predictors	*Estimates*	*CI (95%)*
Injunctive inclusivity norms	0.35	0.09 – 0.62
Identification with Society	0.02	-0.18 – 0.23
Descriptive inclusivity norms	-0.44	-0.70 – -0.19
Topic importance	0.06	0.03 – 0.09
Opinion extremity	-0.04	-0.11 – 0.04
Strength of disapproval	0.02	-0.02 – 0.06
Injunctive inclusivity norms x Identification with Society	-0.06	-0.11 – -0.01
Descriptive inclusivity norms x Identification with Society	0.09	0.05 – 0.14
Observations	1005	
R^2 Bayes	0.078	

	Avoidance tendencies	
Predictors	*Estimates*	*CI (95%)*
Injunctive inclusivity norms	-0.31	-0.72 – 0.06
Identification with Society	-0.20	-0.49 – 0.09
Descriptive inclusivity norms	0.41	0.04 – 0.78
Topic importance	-0.05	-0.10 – -0.00
Opinion extremity	0.20	0.09 – 0.31
Strength of disapproval	0.04	-0.02 – 0.09
Injunctive inclusivity norms x Identification with Society	0.05	-0.02 – 0.12
Descriptive inclusivity norms x Identification with Society	-0.06	-0.13 – 0.01
Observations	1005	
R^2 Bayes	0.062	

	Cooperation willingness	
Predictors	*Estimates*	*CI (95%)*
Injunctive inclusivity norms	-0.01	-0.32 – 0.30
Identification with Society	-0.21	-0.46 – 0.03
Descriptive inclusivity norms	-0.21	-0.52 – 0.10
Topic importance	0.16	0.12 – 0.20

Opinion extremity	0.02	-0.08 – 0.10
Strength of disapproval	0.05	0.00 – 0.09
Injunctive inclusivity norms x Identification with Society	0.03	-0.03 – 0.09
Descriptive inclusivity norms x Identification with Society	0.05	-0.01 – 0.10
Observations	1005	
R^2 Bayes	0.129	

Table S111.

Bayesian moderated multiple regression results including identification with society as moderator (Polish sample)

	**Tolerance**	
Predictors	*Estimates*	*CI (95%)*
Injunctive inclusivity norms	0.26	0.00 – 0.51
Identification with Society	-0.16	-0.34 – 0.02
Descriptive inclusivity norms	-0.46	-0.71 – -0.19
Topic importance	-0.05	-0.09 – -0.00
Opinion extremity	-0.04	-0.11 – 0.03
Strength of disapproval	-0.06	-0.11 – -0.01
Injunctive inclusivity norms x Identification with Society	-0.03	-0.07 – 0.02
Descriptive inclusivity norms x Identification with Society	0.09	0.04 – 0.14
Observations	1003	
R^2 Bayes	0.064	
	**Avoidance tendencies**	
Predictors	*Estimates*	*CI (95%)*
Injunctive inclusivity norms	-0.13	-0.48 – 0.22
Identification with Society	-0.15	-0.40 – 0.10
Descriptive inclusivity norms	0.14	-0.21 – 0.49
Topic importance	0.05	-0.02 – 0.11
Opinion extremity	0.12	0.02 – 0.23
Strength of disapproval	0.11	0.05 – 0.17
Injunctive inclusivity norms x Identification with Society	-0.00	-0.07 – 0.06
Descriptive inclusivity norms x Identification with Society	0.01	-0.05 – 0.07
Observations	1003	
R^2 Bayes	0.052	
	**Cooperation willingness**	
Predictors	*Estimates*	*CI (95%)*
Injunctive inclusivity norms	0.31	0.05 – 0.59
Identification with Society	-0.02	-0.22 – 0.17
Descriptive inclusivity norms	-0.29	-0.56 – -0.01
Topic importance	0.05	0.00 – 0.10

Opinion extremity	0.01	-0.06 – 0.09
Strength of disapproval	-0.01	-0.06 – 0.04
Injunctive inclusivity norms x Identification with Society	-0.02	-0.07 – 0.03
Descriptive inclusivity norms x Identification with Society	0.06	0.01 – 0.11
Observations	1003	
R^2 Bayes	0.081	

Table S112.

Bayesian moderated multiple regression results including identification with society as moderator (Spanish sample)

	Tolerance	
Predictors	*Estimates*	*CI (95%)*
Injunctive inclusivity norms	0.23	0.01 – 0.44
Identification with Society	0.02	-0.14 – 0.18
Descriptive inclusivity norms	-0.20	-0.42 – 0.03
Topic importance	-0.01	-0.07 – 0.04
Opinion extremity	-0.06	-0.14 – 0.03
Strength of disapproval	-0.14	-0.19 – -0.09
Injunctive inclusivity norms x Identification with Society	-0.02	-0.06 – 0.03
Descriptive inclusivity norms x Identification with Society	0.05	0.00 – 0.09
Observations	999	
R^2 Bayes	0.109	

	Avoidance tendencies	
Predictors	*Estimates*	*CI (95%)*
Injunctive inclusivity norms	-0.17	-0.44 – 0.09
Identification with Society	-0.23	-0.42 – -0.02
Descriptive inclusivity norms	0.10	-0.19 – 0.39
Topic importance	0.05	-0.01 – 0.12
Opinion extremity	0.02	-0.09 – 0.13
Strength of disapproval	0.22	0.16 – 0.28
Injunctive inclusivity norms x Identification with Society	0.01	-0.04 – 0.06
Descriptive inclusivity norms x Identification with Society	0.02	-0.04 – 0.08
Observations	999	
R^2 Bayes	0.095	

	Cooperation willingness	
Predictors	*Estimates*	*CI (95%)*
Injunctive inclusivity norms	0.38	0.16 – 0.60
Identification with Society	0.17	0.00 – 0.34
Descriptive inclusivity norms	-0.24	-0.47 – -0.01
Topic importance	0.13	0.08 – 0.19

Opinion extremity	0.07	-0.02 – 0.15
Strength of disapproval	-0.09	-0.13 – -0.04
Injunctive inclusivity norms x Identification with Society	-0.06	-0.11 – -0.02
Descriptive inclusivity norms x Identification with Society	0.06	0.02 – 0.11
Observations	999	
R^2 Bayes	0.104	

Table S113.

Bayesian moderated multiple regression results including identification with society as moderator (Swedish sample)

	Tolerance	
Predictors	*Estimates*	*CI (95%)*
Injunctive inclusivity norms	0.23	-0.02 – 0.49
Identification with Society	0.10	-0.09 – 0.28
Descriptive inclusivity norms	-0.05	-0.28 – 0.18
Topic importance	-0.07	-0.12 – -0.01
Opinion extremity	0.05	-0.02 – 0.13
Strength of disapproval	-0.07	-0.11 – -0.02
Injunctive inclusivity norms x Identification with Society	-0.01	-0.06 – 0.03
Descriptive inclusivity norms x Identification with Society	0.01	-0.03 – 0.06
Observations	998	
R^2 Bayes	0.064	

	Avoidance tendencies	
Predictors	*Estimates*	*CI (95%)*
Injunctive inclusivity norms	0.07	-0.25 – 0.38
Identification with Society	-0.03	-0.26 – 0.20
Descriptive inclusivity norms	-0.19	-0.45 – 0.09
Topic importance	0.05	-0.02 – 0.12
Opinion extremity	0.02	-0.07 – 0.12
Strength of disapproval	0.22	0.16 – 0.28
Injunctive inclusivity norms x Identification with Society	-0.04	-0.09 – 0.02
Descriptive inclusivity norms x Identification with Society	0.04	-0.01 – 0.08
Observations	998	
R^2 Bayes	0.091	

	Cooperation willingness	
Predictors	*Estimates*	*CI (95%)*
Injunctive inclusivity norms	0.33	0.05 – 0.60
Identification with Society	0.10	-0.11 – 0.29
Descriptive inclusivity norms	-0.09	-0.33 – 0.15
Topic importance	0.06	-0.00 – 0.12

Opinion extremity	0.07	-0.01 – 0.16
Strength of disapproval	-0.08	-0.14 – -0.03
Injunctive inclusivity norms x Identification with Society	-0.03	-0.08 – 0.02
Descriptive inclusivity norms x Identification with Society	0.03	-0.02 – 0.07
Observations	998	
R^2 Bayes	0.068	

Table S114.

Bayesian moderated multiple regression results including identification with society as moderator (British sample)

	Tolerance	
Predictors	*Estimates*	*CI (95%)*
Injunctive inclusivity norms	0.19	-0.04 – 0.43
Identification with Society	0.06	-0.09 – 0.21
Descriptive inclusivity norms	-0.15	-0.39 – 0.09
Topic importance	-0.02	-0.07 – 0.03
Opinion extremity	-0.02	-0.11 – 0.06
Strength of disapproval	-0.09	-0.15 – -0.04
Injunctive inclusivity norms x Identification with Society	-0.03	-0.08 – 0.02
Descriptive inclusivity norms x Identification with Society	0.04	-0.00 – 0.09
Observations	1002	
R^2 Bayes	0.058	

	Avoidance tendencies	
Predictors	*Estimates*	*CI (95%)*
Injunctive inclusivity norms	-0.09	-0.40 – 0.24
Identification with Society	-0.26	-0.46 – -0.05
Descriptive inclusivity norms	-0.09	-0.42 – 0.23
Topic importance	0.04	-0.03 – 0.11
Opinion extremity	0.11	0.00 – 0.22
Strength of disapproval	0.24	0.17 – 0.31
Injunctive inclusivity norms x Identification with Society	0.01	-0.05 – 0.08
Descriptive inclusivity norms x Identification with Society	0.01	-0.05 – 0.07
Observations	1002	
R^2 Bayes	0.116	

	Cooperation willingness	
Predictors	*Estimates*	*CI (95%)*
Injunctive inclusivity norms	-0.10	-0.36 – 0.15
Identification with Society	-0.13	-0.30 – 0.04
Descriptive inclusivity norms	0.05	-0.21 – 0.33
Topic importance	0.10	0.04 – 0.16

Opinion extremity	-0.01	-0.09 – 0.08
Strength of disapproval	-0.05	-0.11 – 0.01
Injunctive inclusivity norms x Identification with Society	0.04	-0.01 – 0.09
Descriptive inclusivity norms x Identification with Society	0.01	-0.04 – 0.06
Observations	1002	
R^2 Bayes	0.082	

Identification with the Opinion-Based Group

Table S115.

Bayesian moderated multiple regression results including identification with the opinion-based group as moderator (Czech sample)

	**Tolerance**	
Predictors	*Estimates*	*CI (95%)*
Injunctive inclusivity norms	0.29	-0.02 – 0.60
Identification with the opinion-based group	0.01	-0.19 – 0.21
Descriptive inclusivity norms	-0.19	-0.49 – 0.12
Topic importance	0.04	-0.01 – 0.09
Opinion extremity	0.01	-0.07 – 0.08
Strength of disapproval	-0.20	-0.25 – -0.16
Injunctive inclusivity norms x Identification with the opinion-based group	-0.04	-0.09 – 0.02
Descriptive inclusivity norms x Identification with the opinion-based group	0.06	0.00 – 0.11
Observations	1002	
R^2 Bayes	0.115	
	**Avoidance tendencies**	
Predictors	*Estimates*	*CI (95%)*
Injunctive inclusivity norms	0.21	-0.17 – 0.60
Identification with Society	0.15	-0.10 – 0.40
Descriptive inclusivity norms	-0.03	-0.42 – 0.36
Topic importance	-0.03	-0.08 – 0.03
Opinion extremity	0.03	-0.07 – 0.12
Strength of disapproval	0.31	0.25 – 0.37
Injunctive inclusivity norms x Identification with the opinion-based group	-0.04	-0.11 – 0.02
Descriptive inclusivity norms x Identification with the opinion-based group	0.01	-0.06 – 0.08
Observations	1002	
R^2 Bayes	0.124	
	**Cooperation willingness**	
Predictors	*Estimates*	*CI (95%)*
Injunctive inclusivity norms	0.52	0.16 – 0.87
Identification with the opinion-based group	0.15	-0.08 – 0.39

Descriptive inclusivity norms	-0.25	-0.61 – 0.13
Topic importance	0.08	0.02 – 0.14
Opinion extremity	-0.02	-0.11 – 0.07
Strength of disapproval	-0.15	-0.21 – -0.10
Injunctive inclusivity norms x Identification with the opinion-based group	-0.07	-0.13 – -0.01
Descriptive inclusivity norms x Identification with the opinion-based group	0.07	0.00 – 0.13
Observations	1002	
R^2 Bayes	0.091	

Table S116.

Bayesian moderated multiple regression results including identification with the opinion-based group as moderator (Finnish sample)

	Tolerance	
Predictors	*Estimates*	*CI (95%)*
Injunctive inclusivity norms	0.12	-0.16 – 0.40
Identification with the opinion-based group	-0.24	-0.44 – -0.04
Descriptive inclusivity norms	-0.18	-0.48 – 0.11
Topic importance	0.04	-0.01 – 0.09
Opinion extremity	-0.02	-0.11 – 0.07
Strength of disapproval	-0.33	-0.38 – -0.27
Injunctive inclusivity norms x Identification with the opinion-based group	-0.00	-0.05 – 0.05
Descriptive inclusivity norms x Identification with the opinion-based group	0.05	0.00 – 0.11
Observations	1001	
R^2 Bayes	0.223	
	**Avoidance tendencies**	
Predictors	*Estimates*	*CI (95%)*
Injunctive inclusivity norms	-0.04	-0.38 – 0.30
Identification with the opinion-based group	0.34	0.08 – 0.60
Descriptive inclusivity norms	0.19	-0.19 – 0.56
Topic importance	-0.06	-0.12 – 0.00
Opinion extremity	-0.01	-0.13 – 0.10
Strength of disapproval	0.40	0.33 – 0.47
Injunctive inclusivity norms x Identification with the opinion-based group	-0.02	-0.09 – 0.04
Descriptive inclusivity norms x Identification with the opinion-based group	-0.04	-0.11 – 0.03
Observations	1001	
R^2 Bayes	0.202	
	**Cooperation willingness**	
Predictors	*Estimates*	*CI (95%)*
Injunctive inclusivity norms	0.12	-0.19 – 0.44
Identification with the opinion-based group	0.07	-0.17 – 0.31
Descriptive inclusivity norms	0.21	-0.14 – 0.55
Topic importance	0.15	0.10 – 0.21

Opinion extremity	0.01	-0.09 – 0.12
Strength of disapproval	-0.23	-0.29 – -0.16
Injunctive inclusivity norms x Identification with the opinion-based group	0.01	-0.05 – 0.06
Descriptive inclusivity norms x Identification with the opinion-based group	-0.02	-0.08 – 0.05
Observations	1001	
R^2 Bayes	0.118	

Table S117.

Bayesian moderated multiple regression results including identification with the opinion-based group as moderator (French sample)

	Tolerance	
Predictors	*Estimates*	*CI (95%)*
Injunctive inclusivity norms	0.30	0.09 – 0.52
Identification with the opinion-based group	-0.13	-0.29 – 0.03
Descriptive inclusivity norms	-0.26	-0.46 – -0.05
Topic importance	0.06	-0.00 – 0.11
Opinion extremity	0.03	-0.04 – 0.10
Strength of disapproval	-0.13	-0.17 – -0.08
Injunctive inclusivity norms x Identification with the opinion-based group	-0.02	-0.06 – 0.01
Descriptive inclusivity norms x Identification with the opinion-based group	0.05	0.01 – 0.09
Observations	1005	
R^2 Bayes	0.088	

	Avoidance tendencies	
Predictors	*Estimates*	*CI (95%)*
Injunctive inclusivity norms	-0.18	-0.48 – 0.12
Identification with the opinion-based group	0.11	-0.11 – 0.33
Descriptive inclusivity norms	0.21	-0.08 – 0.50
Topic importance	-0.05	-0.13 – 0.03
Opinion extremity	0.07	-0.03 – 0.17
Strength of disapproval	0.17	0.11 – 0.23
Injunctive inclusivity norms x Identification with the opinion-based group	0.02	-0.03 – 0.07
Descriptive inclusivity norms x Identification with the opinion-based group	-0.02	-0.07 – 0.03
Observations	1005	
R^2 Bayes	0.077	

	Cooperation willingness	
Predictors	*Estimates*	*CI (95%)*
Injunctive inclusivity norms	0.27	0.03 – 0.53
Identification with the opinion-based group	0.04	-0.14 – 0.23
Descriptive inclusivity norms	-0.13	-0.38 – 0.11
Topic importance	0.09	0.02 – 0.16

Opinion extremity	0.05	-0.04 – 0.13
Strength of disapproval	-0.08	-0.14 – -0.03
Injunctive inclusivity norms x Identification with the opinion-based group	-0.02	-0.07 – 0.02
Descriptive inclusivity norms x Identification with the opinion-based group	0.03	-0.01 – 0.08
Observations	1005	
R^2 Bayes	0.078	

Table S118.

Bayesian moderated multiple regression results including identification with the opinion-based group as moderator (German sample)

	Tolerance	
Predictors	*Estimates*	*CI (95%)*
Injunctive inclusivity norms	0.20	-0.06 – 0.48
Identification with the opinion-based group	-0.18	-0.35 – -0.01
Descriptive inclusivity norms	-0.21	-0.48 – 0.05
Topic importance	0.05	-0.00 – 0.10
Opinion extremity	0.02	-0.06 – 0.10
Strength of disapproval	-0.15	-0.20 – -0.10
Injunctive inclusivity norms x Identification with the opinion-based group	-0.00	-0.05 – 0.05
Descriptive inclusivity norms x Identification with the opinion-based group	0.05	0.00 – 0.10
Observations	1009	
R^2 Bayes	0.106	

	Avoidance tendencies	
Predictors	*Estimates*	*CI (95%)*
Injunctive inclusivity norms	-0.17	-0.54 – 0.22
Identification with the opinion-based group	0.29	0.05 – 0.53
Descriptive inclusivity norms	0.44	0.07 – 0.78
Topic importance	-0.04	-0.11 – 0.03
Opinion extremity	-0.01	-0.12 – 0.09
Strength of disapproval	0.36	0.29 – 0.43
Injunctive inclusivity norms x Identification with the opinion-based group	0.01	-0.06 – 0.07
Descriptive inclusivity norms x Identification with the opinion-based group	-0.06	-0.11 – 0.01
Observations	1009	
R^2 Bayes	0.145	

	Cooperation willingness	
Predictors	*Estimates*	*CI (95%)*
Injunctive inclusivity norms	0.29	-0.01 – 0.63
Identification with the opinion-based group	-0.13	-0.32 – 0.07
Descriptive inclusivity norms	-0.36	-0.67 – -0.06
Topic importance	0.18	0.12 – 0.24

Opinion extremity	-0.06	-0.15 – 0.02
Strength of disapproval	-0.12	-0.18 – -0.06
Injunctive inclusivity norms x Identification with the opinion-based group	-0.01	-0.07 – 0.04
Descriptive inclusivity norms x Identification with the opinion-based group	0.07	0.02 – 0.12
Observations	1009	
R^2 Bayes	0.136	

Table S119.

Bayesian moderated multiple regression results including identification with the opinion-based group as moderator (Greek sample)

	Tolerance	
Predictors	*Estimates*	*CI (95%)*
Injunctive inclusivity norms	-0.11	-0.42 – 0.20
Identification with the opinion-based group	-0.28	-0.48 – -0.06
Descriptive inclusivity norms	0.05	-0.29 – 0.39
Topic importance	0.06	-0.00 – 0.11
Opinion extremity	-0.01	-0.09 – 0.08
Strength of disapproval	-0.14	-0.19 – -0.08
Injunctive inclusivity norms x Identification with the opinion-based group	0.04	-0.02 – 0.09
Descriptive inclusivity norms x Identification with the opinion-based group	0.01	-0.04 – 0.07
Observations	1009	
R^2 Bayes	0.085	

	Avoidance tendencies	
Predictors	*Estimates*	*CI (95%)*
Injunctive inclusivity norms	0.11	-0.28 – 0.53
Identification with the opinion-based group	0.33	0.06 – 0.61
Descriptive inclusivity norms	0.03	-0.40 – 0.44
Topic importance	-0.06	-0.14 – 0.01
Opinion extremity	-0.02	-0.12 – 0.09
Strength of disapproval	0.27	0.20 – 0.33
Injunctive inclusivity norms x Identification with the opinion-based group	-0.03	-0.11 – 0.04
Descriptive inclusivity norms x Identification with the opinion-based group	-0.01	-0.08 – 0.07
Observations	1009	
R^2 Bayes	0.109	

	Cooperation willingness	
Predictors	*Estimates*	*CI (95%)*
Injunctive inclusivity norms	0.09	-0.26 – 0.45
Identification with the opinion-based group	-0.09	-0.33 – 0.14
Descriptive inclusivity norms	-0.05	-0.42 – 0.33
Topic importance	0.12	0.06 – 0.19

Opinion extremity	-0.07	-0.16 – 0.03
Strength of disapproval	-0.16	-0.21 – -0.10
Injunctive inclusivity norms x Identification with the opinion-based group	0.00	-0.06 – 0.06
Descriptive inclusivity norms x Identification with the opinion-based group	0.03	-0.04 – 0.09
Observations	1009	
R^2 Bayes	0.084	

Table S120.

Bayesian moderated multiple regression results including identification with the opinion-based group as moderator (Hungarian sample)

	**Tolerance**	
Predictors	*Estimates*	*CI (95%)*
Injunctive inclusivity norms	0.20	-0.10 – 0.49
Identification with the opinion-based group	-0.02	-0.19 – 0.14
Descriptive inclusivity norms	-0.19	-0.47 – 0.10
Topic importance	0.04	-0.01 – 0.08
Opinion extremity	0.04	-0.04 – 0.12
Strength of disapproval	-0.14	-0.19 – -0.09
Injunctive inclusivity norms x Identification with the opinion-based group	-0.02	-0.07 – 0.03
Descriptive inclusivity norms x Identification with the opinion-based group	0.03	-0.01 – 0.08
Observations	1005	
R^2 Bayes	0.054	
	**Avoidance tendencies**	
Predictors	*Estimates*	*CI (95%)*
Injunctive inclusivity norms	-0.26	-0.65 – 0.15
Identification with the opinion-based group	0.06	-0.17 – 0.29
Descriptive inclusivity norms	0.23	-0.16 – 0.61
Topic importance	0.01	-0.05 – 0.07
Opinion extremity	-0.01	-0.12 – 0.11
Strength of disapproval	0.20	0.14 – 0.27
Injunctive inclusivity norms x Identification with the opinion-based group	0.03	-0.04 – 0.10
Descriptive inclusivity norms x Identification with the opinion-based group	-0.03	-0.09 – 0.04
Observations	1005	
R^2 Bayes	0.080	
	**Cooperation willingness**	
Predictors	*Estimates*	*CI (95%)*
Injunctive inclusivity norms	0.18	-0.17 – 0.52
Identification with the opinion-based group	0.11	-0.09 – 0.32
Descriptive inclusivity norms	-0.08	-0.40 – 0.26
Topic importance	0.09	0.04 – 0.15

Opinion extremity	-0.04	-0.14 – 0.06
Strength of disapproval	-0.11	-0.17 – -0.06
Injunctive inclusivity norms x Identification with the opinion-based group	-0.03	-0.09 – 0.03
Descriptive inclusivity norms x Identification with the opinion-based group	0.03	-0.03 – 0.08
Observations	1005	
R^2 Bayes	0.047	

Table S121.

Bayesian moderated multiple regression results including identification with the opinion-based group as moderator (Italian sample)

	Tolerance	
Predictors	*Estimates*	*CI (95%)*
Injunctive inclusivity norms	-0.09	-0.39 – 0.22
Identification with the opinion-based group	-0.17	-0.40 – 0.05
Descriptive inclusivity norms	0.10	-0.16 – 0.37
Topic importance	0.03	-0.03 – 0.08
Opinion extremity	-0.04	-0.11 – 0.03
Strength of disapproval	-0.11	-0.17 – -0.07
Injunctive inclusivity norms x Identification with the opinion-based group	0.03	-0.02 – 0.08
Descriptive inclusivity norms x Identification with the opinion-based group	-0.00	-0.05 – 0.04
Observations	1003	
R^2 Bayes	0.086	

	Avoidance tendencies	
Predictors	*Estimates*	*CI (95%)*
Injunctive inclusivity norms	-0.11	-0.54 – 0.30
Identification with the opinion-based group	0.15	-0.16 – 0.46
Descriptive inclusivity norms	0.04	-0.34 – 0.44
Topic importance	-0.01	-0.08 – 0.07
Opinion extremity	0.01	-0.10 – 0.11
Strength of disapproval	0.24	0.17 – 0.31
Injunctive inclusivity norms x Identification with the opinion-based group	-0.00	-0.07 – 0.07
Descriptive inclusivity norms x Identification with the opinion-based group	0.01	-0.06 – 0.07
Observations	1003	
R^2 Bayes	0.094	

	Cooperation willingness	
Predictors	*Estimates*	*CI (95%)*
Injunctive inclusivity norms	0.23	-0.09 – 0.57
Identification with the opinion-based group	0.07	-0.18 – 0.32
Descriptive inclusivity norms	0.07	-0.24 – 0.40
Topic importance	0.16	0.09 – 0.22

Opinion extremity	-0.00	-0.08 – 0.08
Strength of disapproval	-0.10	-0.15 – -0.04
Injunctive inclusivity norms x Identification with the opinion-based group	-0.01	-0.07 – 0.05
Descriptive inclusivity norms x Identification with the opinion-based group	-0.00	-0.05 – 0.05
Observations	1003	
R^2 Bayes	0.097	

Table S122.

Bayesian moderated multiple regression results including identification with the opinion-based group as moderator (Dutch sample)

	**Tolerance**	
Predictors	*Estimates*	*CI (95%)*
Injunctive inclusivity norms	0.16	-0.11 – 0.43
Identification with the opinion-based group	-0.03	-0.25 – 0.20
Descriptive inclusivity norms	0.07	-0.16 – 0.30
Topic importance	0.08	0.04 – 0.11
Opinion extremity	0.01	-0.07 – 0.10
Strength of disapproval	0.03	-0.01 – 0.07
Injunctive inclusivity norms x Identification with the opinion-based group	-0.01	-0.06 – 0.04
Descriptive inclusivity norms x Identification with the opinion-based group	-0.00	-0.05 – 0.04
Observations	1005	
R^2 Bayes	0.055	
	**Avoidance tendencies**	
Predictors	*Estimates*	*CI (95%)*
Injunctive inclusivity norms	-0.22	-0.62 – 0.16
Identification with the opinion-based group	0.26	-0.07 – 0.58
Descriptive inclusivity norms	0.32	-0.02 – 0.66
Topic importance	-0.08	-0.13 – -0.03
Opinion extremity	0.13	0.01 – 0.25
Strength of disapproval	0.02	-0.03 – 0.08
Injunctive inclusivity norms x Identification with the opinion-based group	0.02	-0.06 – 0.09
Descriptive inclusivity norms x Identification with the opinion-based group	-0.05	-0.12 – 0.02
Observations	1005	
R^2 Bayes	0.050	
	**Cooperation willingness**	
Predictors	*Estimates*	*CI (95%)*
Injunctive inclusivity norms	0.21	-0.11 – 0.53
Identification with the opinion-based group	0.06	-0.20 – 0.33
Descriptive inclusivity norms	0.10	-0.19 – 0.38
Topic importance	0.17	0.14 – 0.22

Opinion extremity	0.02	-0.08 – 0.12
Strength of disapproval	0.05	0.01 – 0.10
Injunctive inclusivity norms x Identification with the opinion-based group	-0.01	-0.07 – 0.05
Descriptive inclusivity norms x Identification with the opinion-based group	-0.01	-0.06 – 0.05
Observations	1005	
R^2 Bayes	0.112	

Table S123.

Bayesian moderated multiple regression results including identification with the opinion-based group as moderator (Polish sample)

	Tolerance	
Predictors	*Estimates*	*CI (95%)*
Injunctive inclusivity norms	0.27	-0.04 – 0.59
Identification with the opinion-based group	-0.20	-0.42 – 0.02
Descriptive inclusivity norms	-0.39	-0.71 – -0.07
Topic importance	-0.03	-0.08 – 0.02
Opinion extremity	-0.03	-0.11 – 0.06
Strength of disapproval	-0.05	-0.10 – -0.01
Injunctive inclusivity norms x Identification with the opinion-based group	-0.03	-0.08 – 0.03
Descriptive inclusivity norms x Identification with the opinion-based group	0.08	0.02 – 0.13
Observations	1003	
R^2 Bayes	0.053	

	Avoidance tendencies	
Predictors	*Estimates*	*CI (95%)*
Injunctive inclusivity norms	-0.09	-0.55 – 0.36
Identification with the opinion-based group	0.25	-0.06 – 0.54
Descriptive inclusivity norms	0.27	-0.15 – 0.70
Topic importance	0.02	-0.05 – 0.08
Opinion extremity	0.08	-0.04 – 0.19
Strength of disapproval	0.10	0.03 – 0.16
Injunctive inclusivity norms x Identification with the opinion-based group	-0.01	-0.09 – 0.06

Descriptive inclusivity norms x Identification with the opinion-based group	-0.02	-0.09 – 0.05
Observations	1003	
R^2 Bayes	0.046	

	Cooperation willingness	
Predictors	*Estimates*	*CI (95%)*
Injunctive inclusivity norms	0.26	-0.06 – 0.63
Identification with the opinion-based group	-0.05	-0.28 – 0.19
Descriptive inclusivity norms	-0.17	-0.52 – 0.15
Topic importance	0.05	-0.00 – 0.10
Opinion extremity	-0.01	-0.10 – 0.08
Strength of disapproval	-0.01	-0.06 – 0.04
Injunctive inclusivity norms x Identification with the opinion-based group	-0.01	-0.07 – 0.05
Descriptive inclusivity norms x Identification with the opinion-based group	0.04	-0.02 – 0.09
Observations	1003	
R^2 Bayes	0.073	

Table S124.

Bayesian moderated multiple regression results including identification with the opinion-based group as moderator (Spanish sample)

	**Tolerance**	
Predictors	*Estimates*	*CI (95%)*
Injunctive inclusivity norms	0.06	-0.24 – 0.37
Identification with the opinion-based group	-0.28	-0.49 – -0.07
Descriptive inclusivity norms	-0.19	-0.48 – 0.11
Topic importance	0.00	-0.06 – 0.06
Opinion extremity	-0.04	-0.14 – 0.04
Strength of disapproval	-0.14	-0.18 – -0.09
Injunctive inclusivity norms x Identification with the opinion-based group	0.02	-0.04 – 0.07
Descriptive inclusivity norms x Identification with the opinion-based group	0.04	-0.01 – 0.09
Observations	999	
R^2 Bayes	0.097	

	**Avoidance tendencies**	
Predictors	*Estimates*	*CI (95%)*
Injunctive inclusivity norms	-0.09	-0.51 – 0.34
Identification with the opinion-based group	0.48	0.22 – 0.75
Descriptive inclusivity norms	0.53	0.13 – 0.93
Topic importance	-0.00	-0.08 – 0.07
Opinion extremity	-0.04	-0.16 – 0.07
Strength of disapproval	0.21	0.15 – 0.27
Injunctive inclusivity norms x Identification with the opinion-based group	-0.01	-0.08 – 0.06
Descriptive inclusivity norms x Identification with the opinion-based group	-0.07	-0.14 – 0.00
Observations	999	
R^2 Bayes	0.102	

	**Cooperation willingness**	
Predictors	*Estimates*	*CI (95%)*
Injunctive inclusivity norms	0.37	0.04 – 0.71
Identification with the opinion-based group	-0.01	-0.23 – 0.22
Descriptive inclusivity norms	-0.28	-0.61 – 0.04
Topic importance	0.13	0.06 – 0.19

Opinion extremity	0.05	-0.05 – 0.15
Strength of disapproval	-0.10	-0.15 – -0.05
Injunctive inclusivity norms x Identification with the opinion-based group	-0.05	-0.11 – 0.01
Descriptive inclusivity norms x Identification with the opinion-based group	0.07	0.01 – 0.12
Observations	999	
R^2 Bayes	0.086	

Table S125.

Bayesian moderated multiple regression results including identification with the opinion-based group as moderator (Swedish sample)

	Tolerance	
Predictors	*Estimates*	*CI (95%)*
Injunctive inclusivity norms	0.40	0.12 – 0.71
Identification with the opinion-based group	-0.12	-0.33 – 0.10
Descriptive inclusivity norms	-0.24	-0.51 – 0.02
Topic importance	-0.03	-0.09 – 0.03
Opinion extremity	0.09	0.02 – 0.17
Strength of disapproval	-0.05	-0.11 – -0.01
Injunctive inclusivity norms x Identification with the opinion-based group	-0.04	-0.10 – 0.01
Descriptive inclusivity norms x Identification with the opinion-based group	0.05	0.01 – 0.10
Observations	998	
R^2 Bayes	0.068	
	**Avoidance tendencies**	
Predictors	*Estimates*	*CI (95%)*
Injunctive inclusivity norms	-0.41	-0.77 – -0.04
Identification with the opinion-based group	0.16	-0.10 – 0.42
Descriptive inclusivity norms	0.28	-0.04 – 0.60
Topic importance	0.01	-0.06 – 0.08
Opinion extremity	-0.04	-0.14 – 0.06
Strength of disapproval	0.20	0.14 – 0.26
Injunctive inclusivity norms x Identification with the opinion-based group	0.05	-0.02 – 0.11
Descriptive inclusivity norms x Identification with the opinion-based group	-0.06	-0.11 – 0.00
Observations	998	
R^2 Bayes	0.106	
	**Cooperation willingness**	
Predictors	*Estimates*	*CI (95%)*
Injunctive inclusivity norms	0.41	0.09 – 0.73
Identification with the opinion-based group	0.19	-0.05 – 0.42
Descriptive inclusivity norms	0.14	-0.14 – 0.43
Topic importance	0.08	0.01 – 0.14

Opinion extremity	0.10	0.02 – 0.19
Strength of disapproval	-0.08	-0.13 – -0.02
Injunctive inclusivity norms x Identification with the opinion-based group	-0.04	-0.10 – 0.01
Descriptive inclusivity norms x Identification with the opinion-based group	-0.01	-0.06 – 0.04
Observations	998	
R^2 Bayes	0.072	

Table S126.

Bayesian moderated multiple regression results including identification with the opinion-based group as moderator (British sample)

	Tolerance	
Predictors	*Estimates*	*CI (95%)*
Injunctive inclusivity norms	0.16	-0.13 – 0.46
Identification with the opinion-based group	-0.26	-0.46 – -0.07
Descriptive inclusivity norms	-0.29	-0.56 – -0.03
Topic importance	0.01	-0.04 – 0.07
Opinion extremity	-0.01	-0.10 – 0.07
Strength of disapproval	-0.08	-0.14 – -0.03
Injunctive inclusivity norms x Identification with the opinion-based group	-0.02	-0.07 – 0.03
Descriptive inclusivity norms x Identification with the opinion-based group	0.07	0.02 – 0.12
Observations	1002	
R^2 Bayes	0.060	

	Avoidance tendencies	
Predictors	*Estimates*	*CI (95%)*
Injunctive inclusivity norms	-0.18	-0.57 – 0.19
Identification with the opinion-based group	0.20	-0.06 – 0.46
Descriptive inclusivity norms	0.22	-0.14 – 0.58
Topic importance	0.00	-0.07 – 0.08
Opinion extremity	0.09	-0.02 – 0.20
Strength of disapproval	0.23	0.16 – 0.31
Injunctive inclusivity norms x Identification with the opinion-based group	0.03	-0.04 – 0.10
Descriptive inclusivity norms x Identification with the opinion-based group	-0.06	-0.12 – 0.01
Observations	1002	
R^2 Bayes	0.104	

	Cooperation willingness	
Predictors	*Estimates*	*CI (95%)*
Injunctive inclusivity norms	0.27	-0.05 – 0.58
Identification with the opinion-based group	-0.03	-0.25 – 0.18
Descriptive inclusivity norms	-0.17	-0.45 – 0.12
Topic importance	0.10	0.03 – 0.16

Opinion extremity	-0.02	-0.11 – 0.08
Strength of disapproval	-0.06	-0.12 – 0.00
Injunctive inclusivity norms x Identification with the opinion-based group	-0.03	-0.09 – 0.02
Descriptive inclusivity norms x Identification with the opinion-based group	0.06	0.00 – 0.11
Observations	1002	
R^2 Bayes	0.070	

Network Norms

Table S127.

Range of means, standard deviations, and Pearson correlations coefficients including personal network norms across countries

Variable	*M*	*SD*	1	2	3	4	5	6	7	8	9	10	11	12
1. Descriptive societal norms	3.71 – 4.36	1.17 – 1.50												
2. Injunctive societal norms	4.14 – 5.08	1.06 – 1.41	.56** – .70**											
3. Descriptive network norms	4.83 – 5.37	1.04 – 1.34	.45** – .55**	.43** – .54**										
4. Injunctive network norms	5.08 – 5.49	1.00 – 1.29	.40** – .46**	.51** – .64**	.64** – .78**									
5. Tolerance	4.69 – 5.11	1.18 – 1.40	.07* – .19**	.10** – .25**	.10** – .29**	.19** – .38**								
6. Avoidance tendencies	3.34 – 3.92	1.69 – 1.80	-.11** – .07*	-.15** – .02	-.18** – .03	-.20** – -.04	-.54** – -.37**							
7. Cooperation willingness	4.68 – 5.05	1.37 – 1.55	.10** – .20**	.08* – .26**	.16** – .31**	.22** – .38**	.51** – .63**	-.50** – -.32**						
8. Strength of disapproval	4.69 – 5.32	1.58 – 1.93	-.10** – .08*	-.08* – .04	-.09** – .12**	-.08* – .11**	-.44** – -.10**	.06 – .42**	-.19** – -.01					
9. Issue importance	4.53 – 5.85	1.48 – 2.17	.01 – .11**	.03 – .13**	.06** – .17**	.08** – .20**	-.13** – .13**	-.08* – .14**	.09** – .28**	-.04 – .38**				
10. Opinion extremity	5.81 – 6.23	0.97 – 1.33	-.08* – .09**	-.03 – .09**	.00 – .17**	-.02 – .13**	-.23** – -.01	.10** – .20**	-.04 – .10**	.18** – .48**	.06 – .51**			
11. Identification diff. score	-0.11 – 1.32	1.83 – 2.19	-.39** – -.11**	-.29** – -.06	-.24** – -.06	-.17** – -.06	-.31** – -.04	.14** – .28**	-.14** – .01	.05 – .44**	.10** – .34**	.27** – .39**		
12. SDO	3.18 – 3.89	1.30 – 1.51	.07* – .25**	-.06 – .19**	-.11** – .06*	-.15** – .02	-.06* – .07*	.07* – .20**	-.11** – .01	-.05 – .08*	-.14** – -.02	-.11** – .03	-.22** – -.05	
13. RWA	4.20 – 4.68	0.98 – 1.21	.20** – .44**	.15** – .38**	.09** – .27**	.07* – .26**	-.03 – .18**	-.02 – .09**	-.05 – .15**	-.07* – .06	-.08* – .08**	-.06 – .05	-.40** – -.18**	.32** – .51**

Note. M and *SD* are used to represent mean and standard deviation, respectively. The identification difference score was calculated by subtracting identification with society from identification with the opinion-based group, resulting in a scale from -6 to 6, with high values suggesting a stronger identification with the opinion-based group relative to society. *indicates $p < .05$. ** indicates $p < .01$.

Table S128.

Hierarchical Bayesian regression results for personal network norms across countries

Tolerance				
Predictors	*Estimates*	*CI (95%)*	*Estimates*	*CI (95%)*
Injunctive network norms	0.12 – 0.40	[00.05 – 0.21] – [0.06 – 0.28]	-0.21 – 0.48	[-0.17 – 0.18] – [-0.09 – 0.43]
Descriptive network norms	0.01 – 0.22	[-0.14 – 0.02]– [-0.08 – 0.15]	-0.32 – 0.23	[-0.28 – 0.11] – [-0.37 – 0.20]
Topic importance	-0.08 – 0.05	[-0.04 – 0.07] – [0.01 – 0.08]	-0.07 – 0.05	[0.01 – 0.08] – [-0.04 – 0.07]
Opinion extremity	-0.06 – 0.05	[0.13 – 0.30] – [0.13 – 0.30]	-0.07 – 0.05	[-0.12 – 0.01] – [-0.13 – 0.04]
Strength of disapproval	-0.33 – 0.02	[-0.02 – 0.06] – [-0.14 – -0.03]	-0.32 – 0.02	[-0.02 – 0.06] – [-0.14 – -0.03]
Injunctive network norms x Descriptive network norms			-0.02 – 0.08	[-0.06 – 0.02] – [-0.02 – 0.09]
Observations	998 – 1009		998 – 1009	
R^2 Bayes	0.068 – 0.246		0.070 – 0.247	

Avoidance tendencies				
Predictors	*Estimates*	*CI (95%)*	*Estimates*	*CI (95%)*
Injunctive network norms	-0.34 – -0.05	[-0.18 – 0.04] – [-0.37 – -0.08]	-0.29 – 0.42	[-0.23 – 0.26] – [-0.61 – 0.04]
Descriptive network norms	-0.17 – 0.10	[-0.27 – -0.06] – [-0.27 – 0.02]	-0.32 – 0.65	[-0.21 – 0.37] – [0.32 – 0.98]
Strength of disapproval	-0.04 – 0.06	[-0.09 – 0.01] – [-0.07 – 0.09]	-0.04 – 0.06	[-0.08 – 0.04] – [-0.01 – 0.14]
Topic importance	0.02 – 0.21	[-0.06 – 0.12] – [0.01 – 0.24]	0.02 – 0.22	[-0.06 – 0.12] – [0.11 – 0.33]
Opinion extremity	0.03 – 0.40	[0.17 – 0.28] – [0.30 – 0.44]	0.04 – 0.41	[-0.02 – 0.09] – [0.08 – 0.72]
Injunctive network norms x Descriptive network norms			-0.13 – 0.04	[-0.12 – -0.01] – [-0.01 – 0.10]
Observations	998 – 1009		998 – 1009	
R^2 Bayes	0.044 – 0.207		0.045 – 0.208	

Cooperation willingness				
Predictors	*Estimates*	*CI (95%)*	*Estimates*	*CI (95%)*

Injunctive network norms	0.12 – 0.44	[0.15 – 0.33] – [0.28 – 0.54]	-0.04 – 0.34	[-0.23 – 0.18] – [0.00 – 0.60]
Descriptive network norms	0.00 – 0.24	[0.01 – 0.18] – [-0.13 – 0.13]	-0.24 – 0.43	[-0.35 – 0.12] – [-0.45 – 0.19]
Strength of disapproval	0.05 – 0.18	[0.11 – 0.19] – [0.04 – 0.17]	0.05 – 0.18	[0.11 – 0.19] – [0.03 – 0.16]
Topic importance	-0.08 – 0.06	[-0.13 – 0.02] – [-0.08 – 0.12]	-0.08 – 0.06	[-0.08 – 0.07] – [-0.07 – 0.12]
Opinion extremity	-0.21 – 0.05	[0.00 – 0.09] – [-0.26 – -0.15]	-0.21 – 0.05	[0.00 – 0.09] – [-0.27 – -0.15]
Injunctive network norms x Descriptive network norms			-0.04 – 0.06	[0.02 – 0.10] – [-0.03 – 0.08]
Observations	998 – 1009		998 – 1009	
R^2 Bayes	0.072 – 0.186		0.076 – 0.187	
